# Chemistry and Tumor Cell Growth Inhibitory Activity of 11,20-Epoxy-3*Z*,5(6)*E*-diene Briaranes from the South China Sea Gorgonian *Dichotella gemmacea*

**DOI:** 10.3390/md11051565

**Published:** 2013-05-15

**Authors:** Cui Li, Mei Jiang, Ming-Ping La, Tie-Jun Li, Hua Tang, Peng Sun, Bao-Shu Liu, Yang-Hua Yi, Zhiyong Liu, Wen Zhang

**Affiliations:** 1 Research Center for Marine Drugs, and Department of Pharmacology, School of Pharmacy, Second Military Medical University, 325 Guo-He Road, Shanghai 200433, China; E-Mails: licuiwan@163.com (C.L.); jiangmei988@yahoo.cn (M.J.); lmp12@163.com (M.-P.L.); ltj204@163.com (T.-J.L.); tanghua0309@126.com (H.T.); sunp918@hotmail.com (P.S.); liubaoshu@126.com (B.-S.L.); yiyanghua@126.com (Y.-H.Y.); 2 Department of Urology, Changhai Hospital, Second Military Medical University, 168 Chang-Hai Road, Shanghai 200003, China

**Keywords:** structure activity relationship, briarane diterpenoids, biological activity, *Dichotella gemmacea*, gorgonian

## Abstract

Eighteen new 11,20-epoxy-3*Z*,5*E*-dien briaranes, gemmacolides AA–AR (**1**–**18**), were isolated together with three known analogs, dichotellides F (**19**) and I (**20**), and juncenolide C (**21**), from the South China Sea gorgonian *Dichotella gemmacea*. The structures of the compounds were elucidated by detailed spectroscopic analysis and comparison with reported data. The absolute configuration was determined based on the ECD experiment. In the *in vitro* bioassay, compounds **1**–**3**, **5**, **6**, **8**–**12**, and **14**–**19** exhibited different levels of growth inhibition activity against A549 and MG63 cell lines. Preliminary structure-activity analysis suggests that 12-*O*-isovalerate may increase the activity whereas 13- or 14-*O*-isovalerate may decrease the activity. Contribution of substitutions at C-2 and C-16 remains uncertain.

## 1. Introduction

Briarane-type diterpenoids are a group of highly oxidized secondary metabolites reported from marine organisms, particularly from octocorals [1]. These metabolites are reported to have a wide spectrum of interesting biological activities, including cytotoxic, anti-inflammatory, antiviral, antifouling, insecticidal and immunomodulatory effects [1,2,3,4,5]. 

**Chart 1:**
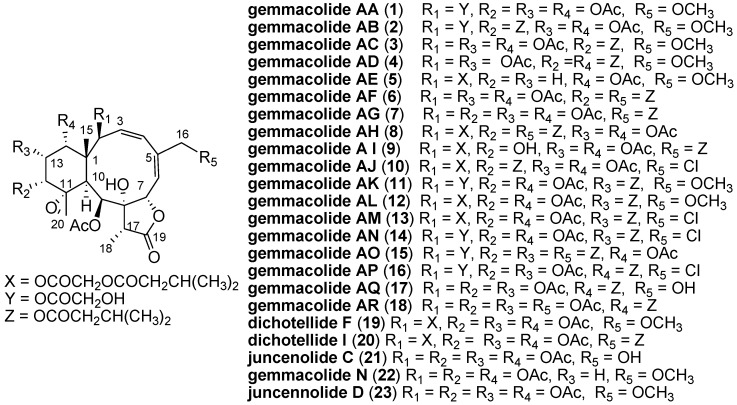
Structures of compounds **1**–**23**.

In the course of our ongoing screening for biologically active secondary metabolites from marine sources [6,7,8,9,10,11,12], we made several collections of the gorgonian *Dichotella gemmacea* off the coast of Beihai, China. Chemical investigation on the species led to the isolation and structure elucidation of nineteen new briaranes, namely gemmacolides G–Y, together with eight known analogues, praelolide, juncin O, junceellolide C, juncenolide D and J, and juncins R, S and U [10,11,12]. In the *in vitro* bioassays, these compounds exhibited different levels of growth inhibition activity against A549 and MG63 cells. In particular, gemmacolides J, V and Y were more active than the positive control adriamycin against A549 cells [10,12], demonstrating a potent activity in tumor cell growth inhibition. The interesting result encourages a systematic study on briarane diterpenoids regarding their chemistry and bioactivities. However, the structural complexity greatly challenged the total synthesis of such metabolites. Until now, only three papers reported the preliminary synthetic research on fragments of briarane diterpenoids [13,14,15,16]. Isolation of the compounds from natural sources is therefore a better choice for the chemical and biological study of such cluster of compounds. This promotes repeated collections of gorgonian *D. gemmacea*, a promising source of briarane diterpenoids. Our continuous investigation on the title animals led to the isolation and structure elucidation of eighteen new briaranes, namely gemmacolides AA–AR (**1**–**18**), and three known analogs, dichotellides F (**19**) and I (**20**) [5], and juncenolide C (**21**) [17] (Chart 1). The structures of the compounds were elucidated by detailed analysis of spectroscopic data and comparisons with reported data. The isolates were tested *in vitro* for their tumor cell growth inhibitory activities. A preliminary analysis was attempted on the structure-activity relationship. We herein report on the isolation, structure elucidation, and bioactivities of these compounds.

## 2. Results and Discussion

Freshly collected specimens of *D. gemmacea* were immediately frozen to −20 °C and stored at this temperature before extraction. The workup for the extraction and isolation of cembrane diterpenoids was basically performed as previously reported [10,11,12]. This common procedure yielded twenty-one pure compounds (**1**–**21**). 

Gemmacolide AA (**1**), a white amorphous powder, had the molecular formula of C_31_H_40_O_16_ based on its HRESI-MS. The IR spectrum showed absorption bands of hydroxyl (3470 cm^−1^), a γ-lactone (1775 cm^−1^), and ester (1741 cm^−1^) functionalities. This observation was in agreement with the signals in the ^13^C NMR and DEPT spectra (Table 1) for ten *sp*^2^ carbon atoms (6 × OC = O, CH = CH, CH = C) at lower field and twenty one *sp*^3^ carbon atoms at higher field (1 × C, 2 × CH, 6 × CH_3_, 2 × OC, 6 × OCH, 3 × OCH_2_, 1 × OCH_3_), accounting for eight double bond equivalents (Table 1, Table 2). The remaining double bond equivalents were due to the presence of four rings in the molecule.

**Table 1:** ^13^C NMR data for gemmacolides AA–AI (**1**–**9**) ^a^.

Position	1	2	3	4	5	6	7	8	9
1	46.6, C	46.6, C	46.5, C	46.5, C	47.3, C	46.4, C	46.4, C	46.4, C	46.5, C
2	75.4, CH	75.4, CH	74.1, CH	74.1, CH	76.1, CH	74.2, CH	74.2, CH	75.5, CH	75.5, CH
3	130.6, CH	130.6, CH	131.3, CH	131.3, CH	131.0, CH	132.1, CH	132.1, CH	131.3, CH	131.5, CH
4	129.5, CH	129.5, CH	128.7, CH	128.6,CH	128.9, CH	127.8, CH	127.8, CH	128.5, CH	128.3, CH
5	141.3, C	141.3, C	140.3, C	141.6, C	141.3, C	139.8, C	139.8, C	139.7, C	138.7, C
6	123.1, CH	123.2, CH	122.8, CH	122.7, CH	122.9, CH	122.5, CH	122.4, CH	122.5, CH	122.2, CH
7	78.9, CH	78.9, CH	78.9, CH	79.0, CH	79.0, CH	78.7, CH	78.7, CH	78.7, CH	78.3, CH
8	81.1, C	81.0, C	81.0, C	80.9, C	80.9, C	81.0, C	81.1, C	81.0, C	81.0, C
9	63.8, CH	63.9, CH	63.9, CH	63.9, CH	64.7, CH	63.9, CH	63.8, CH	63.8, CH	63.9, CH
10	32.7, CH	32.8, CH	32.8, CH	32.7, CH	37.8, CH	32.8, CH	32.7, CH	32.7, CH	31.4, CH
11	58.1, C	58.2, C	58.3, C	58.2, C	60.0, C	58.4, C	58.4, C	58.3, C	60.4, C
12	73.2, CH	72.7, CH	72.9, CH	72.9, CH	29.2, CH_2_	72.8, CH	73.3, CH	72.8, CH	75.2, CH
13	66.5, CH	66.4, CH	66.5, CH	66.5, CH	25.1, CH_2_	66.5, CH	66.7, CH	66.4, CH	67.4, CH
14	73.9, CH	73.9, CH	73.8, CH	73.4, CH	74.6, CH	73.6, CH	73.7, CH	73.6, CH	75.9, CH
15	14.3, CH_3_	14.4, CH_3_	14.5, CH_3_	14.5, CH_3_	14.4, CH_3_	14.5, CH_3_	14.5, CH_3_	14.5, CH_3_	14.6, CH_3_
16	72.2, CH_2_	72.2, CH_2_	72.2, CH_2_	72.1, CH_2_	72.1, CH_2_	62.8, CH_2_	62.8, CH_2_	62.7, CH_2_	62.7, CH_2_
17	44.2, CH	44.2, CH	44.2, CH	44.2, CH	44.1, CH	44.1, CH	44.1, CH	44.1, CH	44.0, CH
18	6.3, CH_3_	6.3, CH_3_	6.3, CH_3_	6.3, CH_3_	6.4, CH_3_	6.3, CH_3_	6.3, CH_3_	6.3, CH_3_	6.3, CH_3_
19	175.2, C	175.3, C	175.3, C	175.3, C	175.7, C	175.3, C	175.2, C	175.2, C	175.3, C
20	48.9, CH_2_	49.1, CH_2_	49.1, CH_2_	49.2, CH_2_	50.4, CH_2_	49.1, CH_2_	48.8, CH_2_	49.0, CH_2_	48.1, CH_2_
9-OAc	170.2, C	170.2, C	170.2, C	170.2, C	170.3, C	170.2, C	170.2, C	170.2, C	170.2, C
	21.5, CH_3_	21.5, CH_3_	21.5, CH_3_	21.6, CH_3_	21.6, CH_3_	21.5, CH_3_	21.5, CH_3_	21.5, CH_3_	21.5, CH_3_
R_1_	171.9, C	171.9, C	169.7, C	169.7, C	166.5, C	169.6, C	169.5, C	166.6, C	166.8, C
	61.1, CH_2_	61.1, CH_2_	21.2, CH_3_	20.6, CH_3_	60.6, CH_2_	21.3, CH_3_	20.5, CH_3_	60.9, CH_2_	60.9, CH_2_
					172.2, C			172.4, C	172.4, C
					42.8, CH_2_			44.5, CH_2_	42.7, CH_2_
					25.7, CH			25.6, CH	25.7, CH
					22.4, 2 × CH_3_			22.3, 2 × CH_3_	22.4, 2 × CH_3_
R_2_	169.7, C	171.8, C	171.8, C	171.9, C		171.9, C	169.8, C	171.9, C	
	20.9, CH_3_	43.5, CH_2_	43.5, CH_2_	43.5, CH_2_		43.4, CH_2_	20.6, CH_3_	43.3, CH_2_	
		25.7, CH	25.7, CH	24.9, CH		25.7, CH		25.6, CH	
		22.3, CH_3_	22.3, CH_3_	22.5, 2 × CH_3_		22.4, 2 × CH_3_		22.3, 2 × CH_3_	
		22.4, CH_3_	22.4, CH_3_						
R_3_	169.7, C	169.7, C	169.7, C	169.7, C		169.8, C	170.5, C	169.7, C	169.9, C
	20.5, CH_3_	20.5, CH_3_	20.9, CH_3_	21.3, CH_3_		20.5, CH_3_	20.7, CH_3_	20.5, CH_3_	20.6, CH_3_
R_4_	170.6, C	170.6, C	170.1, C	172.3, C	170.1, C	170.5, C	170.5, C	170.2, C	170.2, C
	20.9, CH_3_	21.0, CH_3_	21.4, CH_3_	43.5, CH_2_	21.2, CH_3_	20.9, CH_3_	20.9, CH_3_	20.8, CH_3_	20.8, CH_3_
				25.1, CH					
				22.3, 2 × CH_3_					
R_5_	58.5, CH_3_	58.5, CH_3_	58.5, CH_3_	58.5, CH_3_	58.5, CH_3_	172.1, C	172.0, C	172.0, C	172.6, C
						43.3, CH_2_	43.3, CH_2_	43.5, CH_2_	43.2, CH_2_
						25.7, CH	25.7, CH	25.7, CH	25.8, CH
						22.5, 2 × CH_3_	22.4, 2 × CH_3_	22.5, 2 × CH_3_	22.4, 2 × CH_3_

^a^ 100 MHz, in CDCl_3_, assignments made by DEPT, ^1^H-^1^H COSY, HSQC, and HMBC.

**Table 2:** ^1^H NMR data for gemmacolides AA–AF (**1**–**6**) ^a^.

Position	1	2	3	4	5	6
2	5.70, d (9.5)	5.70, d (9.5)	5.57, ov	5.52, ov	5.68, d (9.6)	5.61, ov
3	5.60, dd (10.4, 9.7)	5.60, dd (10.2, 9.5)	5.57, ov	5.53, ov	5.56, dd (10.6, 9.6)	5.61, ov
4	6.33, d (10.4)	6.33, d (10.2)	6.29, d (10.7)	6.28, d (10.1)	6.30, d (10.6)	6.29, ov
6	5.91, d (8.7)	5.90, d (8.8)	5.89, d (8.8)	5.88, d (8.6)	5.88, d (8.7)	5.71, d (8.7)
7	4.99, d (8.7)	4.98, d (8.8)	4.99, d (8.8)	5.01, d (8.6)	4.97, d (8.7)	4.97, d (8.7)
9	4.76, d (4.5)	4.76, d (4.5)	4.75, d (4.6)	4.74, d (4.6)	4.75, d (5.0)	4.74, d (4.7)
10	3.63, d (4.5)	3.62, d (4.5)	3.61, d (4.6)	3.61, ov	3.14, d (5.0)	3.61, ov
12	4.88, d (3.2)	4.92, d (3.3)	4.91, d (3.0)	4.92, d (3.1)	2.19, m	4.91, d (3.3)
					1.11, m	
13β	5.06, dd (3.2, 3.2)	5.08, dd (3.3, 3.5)	5.09, dd (3.0, 3.0)	5.1, dd (3.1, 3.2)	1.95, m	5.08, dd (3.3, 3.2)
13α					1.74, m	
14	5.17, d (3.2)	5.17, d (3.5)	5.21, d (3.0)	5.26, d (3.2)	4.86, br s	5.22, d (3.2)
15	1.14, s	1.14, s	1.13, s	1.13, s	1.13, s	1.14, s
16a	4.51, d (15.1)	4.51, d (14.8)	4.49, d (14.7)	4.5, d (14.4)	4.47, d (14.9)	5.42, d (15.7)
16b	4.23, d (15.1)	4.23, d (14.8)	4.23, d (14.7)	4.23, d (14.4)	4.16, d (14.9)	4.64, d (15.7)
17	2.31, q (7.1)	2.30, ov	2.30, q (7.1)	2.31, q (6.9)	2.27, q (7.1)	2.30, q (7.0)
18	1.16, d (7.1)	1.13, ov	1.14, d (7.1)	1.14, d (6.9)	1.14, d (7.1)	1.13, d (7.0)
20a	3.60, d (2.4)	3.60, d (2.0)	3.61, ov	3.60, ov	3.49, br s	3.61, ov
20b	2.92, d (2.4)	2.92, d (2.0)	2.92, d (2.0)	2.94, br s	2.64, d (2.3)	2.92, br s
8-OH		2.72, s				
9-OAc	2.19, s	2.19, s	2.19, s	2.19, s	2.16, s	2.19, s
R_1_	4.13, d (16.9)	4.13, d (16.8)	1.94, s	1.95, s	4.53, d (15.7)	1.95, s
	4.00, d (16.9)	4.00, d (16.8)			4.48, d (15.7)	
					2.32, ov	
					2.29, ov	
					2.13, m	
					0.98, d (6.5) (×2)	
R_2_	2.16, s	2.22, ov	2.32, ov	2.27, ov		2.30, ov (×2)
		2.32, ov	2.23, ov	2.15, ov		
		2.17, m	2.15, m	2.04, m		2.16, m
		1.01, d (6.5)	1.01, d (6.6)	0.99, d (6.5) (×2)		0.99, d (6.4) (×2)
		0.99, d (6.5)	0.99, d (6.6)			
R_3_	1.94, s	1.93, s	1.95, s	1.95, s		1.94, s
R_4_	2.08, s	2.08, s	2.06, s	2.32, ov (×2)	2.07, s	2.10, s
				2.15, m		
				0.99, d (6.6) (×2)		
R_5_	3.46, s	3.45, s	3.45, s	3.45, s	3.42, s	2.28, ov (×2)
						2.13, m
						0.99, d (6.4) (×2)

^a^ 400 MHz, in CDCl_3_, assignments made by DEPT, ^1^H-^1^H COSY, HSQC, and HMBC; “ov” means overlapped signals.

**Figure 1:**
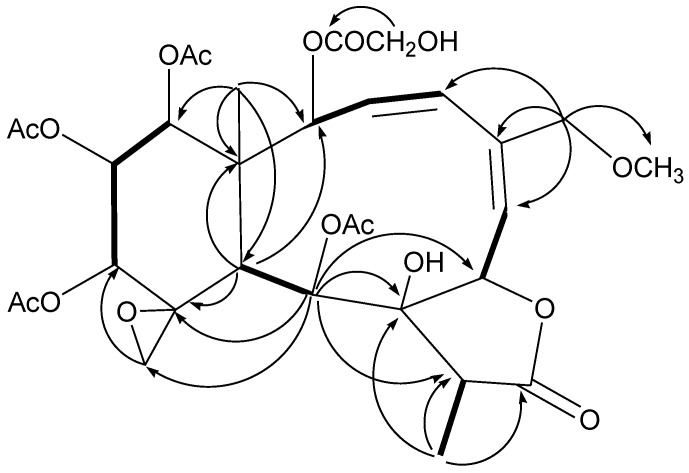
Key HMBC (arrow) and COSY (bond) correlations for compound **1**.

**Figure 2:**
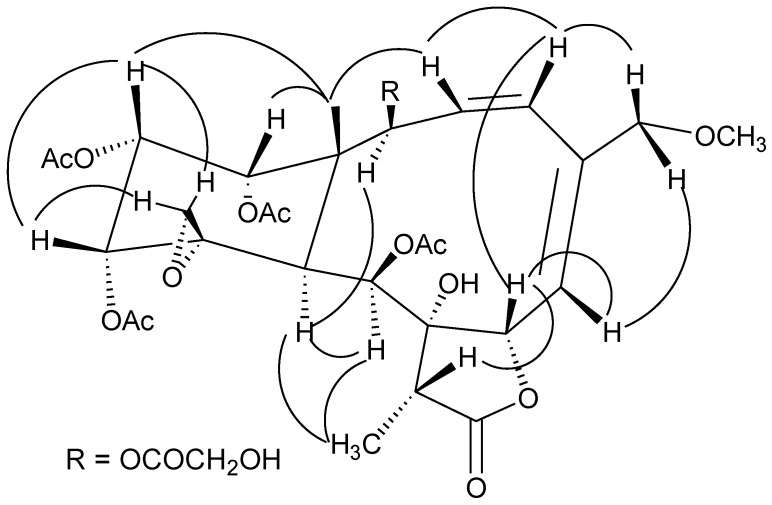
Key NOESY correlations for compound **1**.

Analysis of the ^1^H and ^13^C NMR spectra of **1** (Table 1, Table 2) revealed a great similarity to those of gemmacolide N (**22**) [11]. An additional glycolyl group was observed. The location of the glycolyl group at C-2 was indicated by the distinct HMBC correlations of both H-2 and H-2′ with C-1′. The four acetyl groups were assigned at C-9, C-12, C-13 and C-14 due to the obvious HMBC correlations from the secondary alcohol protons to the respective ester carbonyl groups. The established planar structure of **1** was further supported by the COSY and HMBC spectra as shown in Figure 1. The relative configuration of **1** at the chiral centers was proved the same as that of juncenolide D (**23**) by a NOESY experiment (Figure 2), showing a β configuration of H-7, H-12, H-13, H-14, Me-15, H-17, and CH_2_-20, and an α configuration of H-2, H-9, H-10, and Me-18. The geometry of the Δ^3^ double bond was assigned as *Z* based on the proton coupling constant between H-3 and H-4 (*J* = 10.4 Hz) while that of Δ^5^ was determined as *E* due to the NOESY correlation between H-6 and H_2_-16. The relative configuration of **1** was thus determined as (1S*,2S*,7S*,8S*,9S*,10S*,11R*,12R*,13R*,14R*,17R*). 

As gemmacolide AA (**1**) contained the same lactone and diene chromophores as gemmacolide N (**22**) and they differed only in the nature of the ester group at C-2 and the R_3_, the ECD spectrum of gemmacolide N could therefore be used as an ECD reference for the configurational assignment of gemmacolide AA (**1**) and analogues. Since the absolute configuration of gemmacolide N had been unambiguously determined by a TDDFT calculation of its solution ECD spectrum [11], the absolute configuration of **1** was then suggested as (1*S*,2*S*,7*S*,8*S*,9*S*,10*S*,11*R*,12*R*,13*R*,14*R*,17*R*) due to the congruent ECD curves for **1** and that of gemmacolide N. The assignment of the absolute configuration was in agreement with that of dichotellide T, an analogue recently isolated from the same species of animals with its absolute stereochemistry being determined by X-ray single crystal diffraction analysis [5]. 

Gemmacolide AB (**2**) was obtained as a white amorphous powder with the molecular formula of C_34_H_46_O_16_ being established by HRESI-MS. The structure of **2** differed from that of **1** by the presence of an isovaleryl group instead of an acetyl group at C-12 (Table 1, Table 2). The assignment of the isovalerate ester at C-12 was indicated by the HMBC correlations from both H-12 and H-2″ to the isovaleryl carbonyl carbon. The structure of **2** was thus determined. Its absolute configuration was proved the same as that of **1** on the basis of their similar ECD spectrum.

Gemmacolide AC (**3**) was isolated as a white amorphous powder, had a molecular formula of C_34_H_46_O_15_ as deduced from its HRESI-MS. Its ^1^H and ^13^C NMR spectra data (Table 1, Table 2) were similar to those of **2** with the only difference of the glycolyl group at C-2 in **2** being replaced by an acetyl group in **3**. The location of the acetyl group at C-2 was confirmed by the HMBC correlations of H-2 with C-1′. Its absolute configuration was proved the same as that of **2** based on their similar ECD spectrum.

Gemmacolide AD (**4**) was isolated as a white amorphous powder. Its molecular formula was established as C_37_H_52_O_15_ by HRESI-MS. ^1^H and ^13^C NMR spectra data of **4** (Table 1, Table 2) greatly resembled to those of **3** except that the acetyl group at C-14 in **3** was replaced by an isovaleryl group in **4**. The location of the two isovalery groups at C-12 and C-14 were indicated by the HMBC correlations from both H-12 and H-14 to the isovaleryl carbonyl carbon. The established structure of **4** was further supported by detailed analysis of its 2D NMR data. Its absolute configuration was proven the same as that of **3** based on their similar ECD spectrum.

Gemmacolide AE (**5**) was isolated as a white amorphous powder. The molecular formula C_32_H_44_O_13_ was established by the HRESI-MS. Comparison of overall ^1^H and ^13^C NMR spectra data (Table 1, Table 2) of **5** with those of **19** revealed great similarity. However, signals for two acetyl groups in **19** were disappeared in **5**. The de-acetyl groups were found to be CH_2_-12 and CH_2_-13 based on the proton sequence from H_2_-12 to H-14 as deduced from the ^1^H-^1^H COSY experiment. The relative configuration for other chiral centers remained intact due to the NOESY experiment. The ECD experiment suggested (−)-(1*R*,2*S*,7*S*,8*S*,9*S*,10*S*,11*R*,14*S*,17*R*) absolute configuration for compound **5**.

Gemmacolide AF (**6**) was isolated as a white amorphous powder. The molecular formula C_38_H_52_O_16_ was established by the HRESI-MS. ^1^H and ^13^C NMR spectra of **6** (Table 1, Table 2) were similar to those of compound **3** except that oxygenated methyl group in **3** was replaced by an isovaleryl group in **6**. Two isovaleryl groups were attached at C-12 and C-16 due to the HMBC correlations. The relative and absolute configuration of **6** was proved the same as that of **3** by the analysis of NOESY and ECD spectra.

Gemmacolide AG (**7**), a white amorphous powder, showed a molecular formula of C_35_H_46_O_16_ in the HRESI-MS. ^1^H and ^13^C NMR spectroscopic data of **7** were almost identical to those of **6** (Table 1, Table 3) except for the replacement of the 12-isovaleryl group in **6** by an acetyl group in **7**. The absolute structure of **7** was proved the same as that of **6** by the analysis of NOESY and ECD spectra.

**Table 3:** ^1^H NMR data for gemmacolides AG-AL (**7**–**12**) ^a^.

Position	7	8	9	10	11	12
2	5.61, ov	5.67, ov	5.78, d (9.9)	5.58, d (9.9)	5.7, d (9.9)	5.64, d (9.7)
3	5.61, ov	5.62, dd (10.0, 9.9)	5.61, dd (9.9, 10.3)	5.60, dd (10.1, 9.9)	5.6, dd (9.9, 10.3)	5.60, dd (9.7, 9.8)
4	6.29, br s	6.34, d (10.4)	6.32, d (10.3)	6.40, d (10.1)	6.40, d (10.3)	6.33, d (9.8)
6	5.71, d (8.6)	5.69, ov	5.65, ov	6.06, d (8.4)	5.9, d (8.6)	5.89, d (8.7)
7	4.97, d (8.6)	4.96, d (9.0)	4.96, ov	4.95, d (8.4)	4.99, d (8.6)	4.98, d (8.7)
9	4.74, d (4.4)	4.74, d (4.7)	4.74, d (4.8)	4.74, d (4.7)	4.74, d (4.8)	4.75, d (4.5)
10	3.61, ov	3.61, d (4.7)	3.6, ov	3.59, ov	3.62, d (4.8)	3.62, d (4.5)
12	4.86, br s	4.91, d (3.0)	3.45, br s	4.91, d (3.2)	4.9, d (3.4)	4.89, d (3.1)
13β	5.08, dd (3.0, 2.8)	5.08, dd (3.0, 2.8)	4.97, ov	5.08, dd (3.3, 3.2)	5.08, dd (3.4, 3.5)	5.09, dd (3.1, 3.0)
14	5.22, br s	5.20, d (2.8)	5.30, br s	5.18, d (3.3)	5.16, d (3.5)	5.17, d (3.0)
15	1.14, s	1.13, s	1.13, s	1.13, s	1.13, s	1.13, s
16a	5.45, d (15.6)	5.42, d (15.6)	5.46, d (15.6)	4.68, d (16.2)	4.5, d (14.7)	4.53, d (15.3)
16b	4.64, d (15.6)	4.59, d (15.6)	4.56, d (15.6)	4.45, ov	4.25, d (14.7)	4.11, d (15.3)
17	2.31, q (7.0)	2.30, q (6.9)	2.28, q (6.9)	2.30, q (7.0)	2.31, q (6.9)	2.29, q (6.9)
18	1.15, d (7.0)	1.13, d (6.9)	1.13, d (6.9)	1.14, d (7.0)	1.15, d (6.9)	1.15, d (6.9)
20a	3.61, ov	3.60, ov	3.60, br s	3.59, ov	3.60, d (2.6)	3.60, d (2.1)
20b	2.92, br s	2.93, d (2.2)	2.76, d (2.1)	2.93, d (1.4)	2.97, d (2.6)	2.92, d (2.1)
9-OAc	2.19, s	2.19, s	2.19, s	2.18, s	2.19, s	2.18, s
R_1_	1.95, s	4.52, d (15.6)	4.52, d (15.6)	4.54, d (15.7)	4.15,d (16.9)	4.53, d (15.7)
		4.43, d (15.6)	4.42, d (15.6)	4.44, d (15.7)	4.0, d (16.9)	4.43, d (15.7)
		2.28, ov (×2)	2.28, ov (×2)	2.29, ov (×2)		2.28, ov (×2)
		2.16, m	2.13, m	2.13, m		2.15, ov
		0.98, ov (×2)	0.99, d (6.5) (×2)	0.98, ov (×2)		0.98, ov (×2)
R_2_	2.16, s	2.35, ov (×2)		2.32, ov	2.16, s	2.14, s
				2.23, ov		
		2.16, m		2.17, m		
		0.98, ov (×2)		1.00, ov (×2)		
R_3_	1.95, s	1.94, s	2.03, s	1.94, s	2.2, ov	2.09, ov (×2)
					2.08, ov	1.99, ov
					1.97, m	0.92, d (6.5)
					0.9, d (6.5) (×2)	0.91, d (6.5)
R_4_	2.10, s	2.10, s	2.13, s	2.07, s	2.06, s	2.05, s
R_5_	2.3, ov (×2)	2.26, ov (×2)	2.28, ov (×2)		3.45, s	3.44, s
	2.05, m	2.16, m	2.13, m			
	0.99, d (6.6) (×2)	0.98, ov (×2)	0.99, d (6.6) (×2)			

^a^ 400 MHz, in CDCl_3_, assignments made by DEPT, ^1^H-^1^H COSY, HSQC, and HMBC; “ov” means overlapped signals.

Gemmacolide AH (**8**) was isolated as a white amorphous powder with the molecular formula of C_43_H_60_O_1__8_ being established by HRESI-MS. Its ^1^H and ^13^C NMR spectra data (Table 1, Table 3) were similar to those of **6** with the only difference of the acetyl group at C-2 in **6** being replaced by an isovaleric acetyl group in **8**. This assignment was clearly indicated by the long range correlation from both H_2_-4′ and H_2_-2′ to C-3′, and from both H-2 and H_2_-2′ to C-1′. The relative configuration of all the chiral centers remained intact, which was supported by a NOESY experiment. The absolute configuration of **8** was obtained based on the ECD experiment.

Gemmacolide AI (**9**), a white amorphous powder, had a molecular formula of C_38_H_52_O_17_ as deduced from its HRESI-MS. ^1^H and ^13^C NMR spectra of **9** (Table 1, Table 3) showed similarity to those of compound **8**. The isovaleryl group in at C-12 in **8** was replaced by a hydroxy group in **9**. This conclusion was supported by extensive 2D NMR analysis. Its ECD spectrum indicated the same absolute configuration as that of **8**.

Gemmacolide AJ (**10**) had a molecular of C_38_H_51_O_16_Cl as established by HRESI-MS. An isotopic ratio of 3:1 observed in the molecular ion peak at *m*/*z* 821/823 ([M + Na]^+^) confirmed the appearance of a chlorine atom in the molecule. ^1^H and ^13^C NMR spectra of **10** closely resembled those of **8** (Table 3, Table 4), except for the absence of the signals of one isovaleryl group. This fact together with the up-field shifted signal of C-16 in the ^13^C NMR spectra (δ_C_ 72.0, *t* in **4** and 44.2, *t* in **10**) led to the location of chlorine atom at C-16 [14]. The structure of **10** was thus determined. Its relative and absolute stereochemistry was proved the same as that of **8** by NOESY and ECD measurements.

**Table 4:** ^13^C NMR data for gemmacolides AJ–AR (**10**–**18**) ^a^.

Position	10 ^b^	11 ^b^	12 ^b^	13 ^b^	14 ^c^	15 ^b^	16 ^d^	17^ b^	18 ^b^
1	46.5, C	46.6, C	46.5, C	46.5, C	46.5, C	46.5, C	46.5, C	46.5, C	46.5, C
2	75.3, CH	75.4, CH	75.4, CH	75.2, CH	75.2, CH	75.5, CH	75.2, CH	75.5, CH	74.1, CH
3	131.1, CH	130.6, CH	129.4, CH	131, CH	131.1, CH	131.5, CH	131.1, CH	131.3, CH	132.1, CH
4	129.0, CH	129.5, CH	130.5, CH	129, CH	129.1, CH	128.5, CH	129.0, CH	129.5, CH	127.8, CH
5	139.9, C	141.3, C	141.6, C	139.9, C	139.7, C	139.5, C	144.3, C	144.7, C	139.9, C
6	126.0, CH	123.2, CH	122.6, CH	125, CH	126.3,CH	122.8, CH	126.2, CH	123.4, CH	122.8, CH
7	78.5, CH	78.9, CH	79.0, CH	78.5, CH	78.5, CH	78.7, CH	78.5, CH	78.7, CH	78.7, CH
8	80.9, C	81.0, C	81.1, C	81.1, C	81.0, C	81.0, C	80.0, C	81.1, C	80.2, C
9	63.7, CH	63.8, CH	63.9, CH	63.9, CH	63.6, CH	63.8, CH	63.5, CH	63.8, CH	63.8, CH
10	32.7, CH	32.7, CH	32.7, CH	32.1, CH	32.6, CH	32.7, CH	31.6, CH	32.7, CH	32.6, CH
11	58.3, C	58.2, C	58.2, C	58.2, C	58.1, C	58.3, C	58.0, C	58.4, C	58.5, C
12	72.7, CH	73.2, CH	73.2, CH	73.2, CH	73.0, CH	72.7, CH	73.1, CH	73.3, CH	73.3, CH
13	66.3, CH	66.2, CH	66.3, CH	66.3, CH	66.4, CH	66.2, CH	66.3, CH	66.5, CH	66.6, CH
14	73.6, CH	74.0, CH	73.9, CH	73.9, CH	73.8, CH	73.8, CH	73.3, CH	73.4, CH	73.3, CH
15	14.4, CH_3_	14.4, CH_3_	14.4, CH_3_	14.4, CH_3_	14.3, CH_3_	14.4, CH_3_	14.7, CH_3_	14.5, CH_3_	14.5, CH_3_
16	44.2, CH_2_	72.2, CH_2_	72.0, CH_2_	44.2, CH_2_	44.5, CH_2_	62.8, CH_2_	44.4, CH_2_	63.2, CH_2_	63.2, CH_2_
17	44.0, CH	44.2, CH	44.2, CH	44.0, CH	44.0, CH	44.1, CH	43.2, CH	44.1, CH	44.1, CH
18	6.3, CH_3_	6.3, CH_3_	6.3, CH_3_	6.9, CH_3_	6.3, CH_3_	6.3, CH_3_	6.3, CH_3_	6.3, CH_3_	6.3, CH_3_
19	175.0, C	175.6, C	175.2, C	175.2, C	174.9, C	175.2, C	175.1, C	175.3, C	175.2, C
20	49.0, CH_2_	48.9, CH_2_	48.9, CH_2_	48.9, CH_2_	49.8, CH_2_	49.0, CH_2_	49.0, CH_2_	49.1, CH_2_	49.0, CH_2_
9-OAc	170.1, C	170.3, C	170.2, C	170.2, C	170.1, C	170.1, C	170.2, C	170.3, C	170.2, C
	21.5, CH_3_	21.5, CH_3_	21.5, CH_3_	21.5, CH_3_	21.5, CH_3_	21.6, CH_3_	21.5, CH_3_	21.6, CH_3_	21.5, CH_3_
R_1_	167.0, C	171.9, C	166.6, C	166.6, C	172.2, C	172.0, C	172.8, C	170.7, C	169.4, C
	60.8, CH_2_	61.2, CH_2_	60.9, CH_2_	60.9, CH_2_	61.1, CH_2_	61.2, CH_2_	61.1, CH_2_	21.5, CH_3_	21.3, CH_3_
	172.4, C		172.4, C	172.4, C					
	42.7, CH_2_		42.8, CH_2_	42.7, CH_2_					
	25.6, CH		25.7, CH	25.7, CH					
	22.3, 2 × CH_3_		22.4, 2 × CH_3_	22.4, 2 × CH_3_					
R_2_	171.8, C	169.6, C	169.6, C	169.6, C	169.7, C	171.8, C	169.8, C	169.9, C	169.9, C
	43.6, CH_2_	21.5, CH_3_	20.9, CH_3_	20.9, CH_3_	20.9, CH_3_	43.4, CH_2_	21.3, CH_3_	21.1, CH_3_	20.8, CH_3_
	25.7, CH					25.7, CH			
	22.4, 2 × CH_3_					22.4, 2 × CH_3_			
R_3_	169.7, C	171.8, C	171.7, C	171.7, CH	171.4, C	171.7, C	169.7, C	169.9, C	169.8, C
	20.5, CH_3_	42.6, CH_2_	42.6, CH_2_	42.6, CH_2_	44.5, CH_2_	42.6, CH_2_	20.5, CH_3_	21.1, CH_3_	20.6, CH_3_
		25.0, CH	25.0, CH	25.0, CH	25.7, CH	25.0, CH			
		22.3, 2 × CH_3_	22.5, CH_3_	22.4, 2 × CH_3_	22.4, CH_3_	22.3, 2 × CH_3_			
			22.4, CH_3_		22.3, CH_3_				
R_4_	170.3, C	170.4, C	170.0, C	170.0, C	170.6, C	170.9, C	172.4, C	172.4, C	172.5, C
	20.8, CH_3_	21.0, CH_3_	20.8, CH_3_	20.8, CH_3_	20.8, CH_3_	20.9, CH_3_	42.6, CH_2_	43.1, CH_2_	43.1, CH_2_
							25.0, CH	25.1, CH	25.1, CH
							22.3, 2 × CH_3_	22.5, CH_3_	22.5, CH_3_
								22.4, CH_3_	22.4, CH_3_
R_5_		58.5, CH_3_	58.5, CH_3_			172.2, C			170.0, C
						43.3, CH_2_			21.0, CH_3_
						25.6, CH			
						22.5, 2 × CH_3_			

^a^ In CDCl_3_, assignments made by DEPT, ^1^H-^1^H COSY, HSQC, and HMBC; ^b^ Measured at 100 MHz; ^c^ Measured at 125 MHz; ^d^ Measured at 150 MHz.

Gemmacolide AK (**11**), a white amorphous powder, displayed the molecular formula of C_34_H_46_O_16_ in the HRESI-MS. Its ^1^H and ^13^C NMR spectra data (Table 3, Table 4) showed great similarity to those of **2**. However, the substitutions of isovaleric acetyl group at C-12 and acetyl group at C-13 in **2** had to be interchanged in **11** based on the HMBC experiment. The relative configuration for all chiral centers remained intact due to the NOEY experiment. Its absolute configuration was proved the same as that of **2** due to their similarity in ECD spectrum.

Gemmacolide AL (**12**) was isolated as a white amorphous powder, had a molecular of C_39_H_54_O_17_ as established by HRESI-MS. ^1^H and ^13^C NMR spectroscopic data of **12** were almost identical to those of **11** (Table 3, Table 4) except for the replacement of glycolyl group by the isovaleric acetyl. Detailed analysis of ^1^H-^1^H COSY and HMBC spectra clarified the isovaleric acetyl at C-2. The structure of **12** was thus determined, showing the same relative and absolute configuration as that of **11**, as further confirmed by the NOESY and ECD experiments.

Gemmacolide AM (**13**) was isolated as a white amorphous powder. The molecular formula C_3__8_H_51_ClO_16_ was established by the HRESI-MS. Its ^1^H and ^13^C NMR spectra data (Table 4, Table 5) closely resembled to those of **10**. The substitutions of isovaleric acetyl group at C-12 and acetyl group at C-13 in **10** had to be interchanged in **13** due to the detailed analysis on the HMBC spectra. NOESY and ECD experiments led to the same absolute configuration for both compounds.

**Table 5:** ^1^H NMR data for gemmacolides AM–AR (**13**–**18**) ^a^.

Position	13 ^b^	14 ^c^	15 ^b^	16 ^d^	17 ^b^	18 ^b^
2	5.57, d (9.6)	5.64, ov	5.73, d (9.8)	5.6, d (9.6)	5.61, ov	5.54, d (9.7)
3	5.65, dd (9.6, 10.3)	5.65, ov	5.63, dd (10.3, 9.8)	5.65, dd (9.6, 10.3)	5.60, ov	5.60, dd (9.7, 10.3)
4	6.40, d (10.3)	6.41, d (8)	6.33, d (10.3)	6.40, d (10.3)	6.35, d (8.5)	6.29, d (10.3)
6	6.06, d (8.6)	6.07, d (8.6)	5.73, ov	6.07, d (8.6)	5.81, d (8.5)	5.75, d (8.5)
7	4.95, d (8.6)	4.94, d (8.6)	4.96, d (8.5)	4.93, d (8.6)	4.96, d (8.5)	4.97, d (8.5)
9	4.74, d (4.7)	4.75, d (4.5)	4.75, d (4.5)	4.74, d (4.7)	4.74, d (4.8)	4.74, d (4.8)
10	3.61, ov	3.61, ov	3.62, d (4.5)	3.61, ov	3.60, d (4.8)	3.61, d (4.8)
12	4.96, d (3.0)	4.92, d (3.4)	4.92, d (3.3)	4.88, br s	4.89, d (3.3)	4.88, d (3.2)
13β	5.08, dd (3.0, 3.1)	5.23, dd (3.4, 3.5)	5.09, dd (3.2, 3.3)	5.08, dd (3.2, 3.3)	5.10, dd (3.3, 3.3)	5.09, dd (3.2, 3.0)
14	5.16, d (3.1)	5.18, d (3.5)	5.19, d (3.2)	5.21, br s	5.28, d (3.3)	5.26, d (3.0)
15	1.13, s	1.14, s	1.15, s	1.13, s	1.14, s	1.14, s
16a	4.67, d (13.6)	4.67, d (13.5)	5.46, d (15.6)	4.65, d (13.8)	4.49, br s	5.31, d (16.1)
16b	4.47, d (13.6)	4.54, d (13.5)	4.62, d (15.6)	4.57, d (13.8)	4.49, br s	4.72, d (16.1)
17	2.31, ov	2.3, ov	2.30, ov	2.31, ov	2.30, ov	2.31, q (7.1)
18	1.13, d (6.9)	1.14, d (6.9)	1.14, ov	1.14, d (7.2)	1.15, ov	1.15, ov
20a	3.60, ov	3.60, ov	3.60, ov	3.60, ov	3.63, br s	3.60, br s
20b	2.94, d (2.2)	2.94, d (2.0)	2.94, br s	2.95, br s	2.93, br s	2.93, br s
9-OAc	2.19, s	2.19, s	2.19, s	2.19, s	2.20, s	2.19, s
R_1_	4.54, d (15.6)	4.17, d (16.8)	4.12, d (15.2)	4.15, d (16.8)	1.98, s	1.94, s
	4.44, d (15.6)	4.02, d (16.8)	3.99, d (15.2)	4.06, d (16.8)		
	2.28, ov (×2)					
	2.10, ov					
	0.99, d (6.5) (×2)					
R_2_	2.16, s	2.16, s	2.31, ov (×2)	2.16, s	2.17, s	2.13, s
			2.16, ov			
			0.99, ov (×2)			
R_3_	2.32, ov	2.08, ov (×2)	2.06, ov (×2)	1.95, s	1.95, s	1.95, s
	2.25, ov					
	1.99, ov	1.99, ov	1.99, ov			
	0.90, d (6.0)	0.91, d (6.5)	0.99, d (6.7)			
	0.93, d (6.0)	0.90, d (6.5)	0.99, d (6.7)			
R_4_	2.07, s	2.09, s	2.12, s	2.3, ov (×2)	2.31, ov	2.29, ov
					2.18, ov	2.21, ov
				2.1, ov	2.11, ov	2.09, ov
				0.99, d (6.6)	1.00, d (6.8)	0.99, d (6.8)
				0.99, d (6.6)	0.98, d (6.8)	0.97, d (6.8)
R_5_			2.29, ov (×2)			2.17, s
			2.18, ov			
			0.99, ov (×2)			

^a^ In CDCl_3_, assignments made by DEPT, ^1^H-^1^H COSY, HSQC, and HMBC; “ov” means overlapped signals; ^b^ Measured at 400 MHz; ^c^ Measured at 500 MHz; ^d^ Measured at 600 MHz.

Gemmacolide AN (**14**) was obtained as a white amorphous powder with the molecular formula of C_33_H_43_ClO_15_ being established by HRESI-MS. The structure of **14** was similar to those of **13** with the only difference of the isovaleric acetyl at C-2 in **13** being replaced by a glycolyl group in **14** (Table 4, Table 5). The location of the glycolyl group at C-2 was confirmed by the HMBC correlations of both H-2′ and H-2 with C-1′. The structure of **14** was thus determined. Its absolute configuration was proved the same as that of **13** based on their similar ECD spectra data.

Gemmacolide AO (**15**) was obtained as a white amorphous powder and exhibited a molecular formula of C_41_H_58_O_17_ as deduced from its HRESI-MS. ^1^H and ^13^C NMR spectra of **15** were similar to those of **6**. However, two of the acetyl groups in **6** were replaced by a glycolyl at C-2 and an isovaleryl group at C-13 in **15** (Table 4, Table 5). The planar and relative structure of **15** was confirmed by extensive 2D NMR analysis and correlations with co-isolated analogues. Its ECD spectrum suggested the same absolute configuration as that of **6**.

Gemmacolide AP (**16**) was isolated as a white amorphous powder, had a molecular of C_33_H_43_ClO_15_ as established by HRESI-MS. ^1^H and ^13^C NMR spectroscopic data of **16** were almost identical to those of **14** (Table 4, Table 5). Detailed analysis of ^1^H-^1^H COSY and HMBC spectra indicated an interchangeable acetyl group and isovaleryl group in **16** with respected to those in **14**. Their absolute configuration was proven the same based on NOESY and ECD experiment.

Gemmacolide AQ (**17**) was isolated as a white amorphous powder, showed a molecular formula of C_33_H_44_O_15_ as deduced from its HRESI-MS. ^1^H and ^13^C NMR spectroscopic data of **17** were almost identical to those of **3** (Table 4, Table 5), showing a similar substituted functionalities with the exception of the disappearance of the signals for the oxygenated methyl group. The isovaleryl group, however, was proven to be attached to C-14 instead of C-12 based on the analysis of ^1^H-^1^H COSY and HMBC spectra. The structure of **17** was then determined, having the same relative and absolute stereochemistry as that of **3** due to the NOESY and ECD measurements.

Gemmacolide AR (**18**) was found to be a white amorphous powder, having the molecular formula of C_35_H_46_O_16_ based on the HRESI-MS. ^1^H and ^13^C NMR spectra of **18** resembled to those of compound **17** except for the appearance of an additional acetyl group (Table 4, Table 5). The five acetyl groups were thus assigned to C-2, C-9, C-12, C-13, and C-16, which was supported by the ^1^H-^1^H COSY and HMBC experiments. The replacement of the hydroxyl group by an acetoxyl group at C-16 was further supported by the remarkable downfield proton signal of H_2_-16 from δ_H_ 4.47 (2H, br s) in **17** to in **18** δ_H_ 5.31, 4.72 (each d, *J* = 16.0 Hz). The relative and absolute configuration of **18** was also proved the same as those of **17** by the NOESY and ECD experiments.

All the compounds were evaluated for their tumor cell growth inhibition activity towards tumor cell lines A549 and MG63 [18]. In the *in vitro* bioassays, compounds **1**–**3**, **5**, **6**, **8**–**12**, and **14**–**19** exhibited different level of growth inhibition against tested tumor cells whereas compounds **4**, **7**, **13**, **20**, and **21** were not active (Table 6). Compound **8** showed potent growth inhibition towards both tumor cell lines, being similar as that of positive control adriamycin. This observation, when comparing with the activity of **9** and **20**, showed a positive contribution of the 12-*O*-isovalerate to the activity as described previously [10,11]. The increased activity of **6** with respect to that of **7** further supported the above conclusion. The replacement of an acetyl group by an isovaleryl group at C-13 will marked decrease the activity as observed in **12** and **19**, and **1** and **11** as well. The observation was in good agreement with the remarkably decreasing activity of **2** and **10** with respect to their 12,13-interchangeble analogues **11** and **13**, respectively. Similar situation was also suggested for C-14 by comparing the activity of **3** and **21** with those **4** and **17**, respectively. These facts suggested that 13- or 14-*O*-isovalerate may decrease the activity. As for the isovaleric acetyl substitution at C-2, **8** and **12** showed a marked increasing activity comparing with their 2-OAc or glycolyl analogues whereas **13** showed a marked decreasing activity comparing with its 2-glycolyl analogue. This observation led to somewhat confliction for the contribution of isovaleric acetyl or glycoly to the activity. This confliction was also observed for 16-substitutio when 16-OMe briaranes **11** and **12** compared with their 16-Cl analogues **14** and **13**, respectively. 

**Table 6:** Cytotoxic assay for compounds **1**–**21** (IC_50_ μM).

Compound	A549	MG63	Compound	A549	MG63
**1**	14.7	28.7	**12**	>37.8	37.8
**2**	19.4	22.8	**13**	-	-
**3**	17.9	42.7	**14**	13.4	12.1
**4**	-	-	**15**	78.5	25.8
**5**	20.1	41.3	**16**	10.1	17.7
**6**	27.4	33.0	**17**	28.7	>100.0
**7**	-	-	**18**	16.8	-
**8**	5.0	5.0	**19**	9.7	14.9
**9**	27.7	37.5	**20**	-	-
**10**	39.9	9.1	**21**	-	-
**11**	-	39.0	Adriamycin	2.8	3.2

## 3. Experimental Section

### 3.1. General Experimental Procedures

Commercial silica gel (Yantai, China, 200–300; 400–500 mesh) was used for column chromatography. Precoated silica gel plates (Yantai, China, HSGF-254) were used for analytical Thin Layer Chromatography (TLC). Spots were detected on TLC under UV or by heating after spraying with anisaldehyde-sulphuric acid reagent. The NMR spectra were recorded at 300 K on Bruker DRX 400 and Avance 600 spectrometers. Chemical shifts are reported in parts per million (δ), with use of the residual CDCl_3_ signal (δ_H_ = 7.27 ppm) as an internal standard for ^1^H NMR and CDCl_3_ (δ_C_ = 77.02 ppm) for ^13^C NMR; Coupling constants (*J*) in Hz. ^1^H NMR and ^13^C NMR assignments were supported by ^1^H-^1^H COSY, HSQC, HMBC and NOESY experiments. The following abbreviations are used to describe spin multiplicity: s = singlet, d = doublet, t = triplet, m = multiplet, br s = broad singlet, br d = broad doublet, dd = doublet of doublets, ov = overlapped signals. Optical rotations were measured in CHCl_3_ on an Autopol IV polarimeter at the sodium D line (590 nm). Infrared spectra were recorded in thin polymer films on a Nexus 470 FT-IR spectrophotometer (Nicolet); peaks are reported in cm^−1^. UV absorption spectra were recorded on a Varian Cary 100 UV-Vis spectrophotometer; peaks wavelengths are reported in nm. Circular dichroism (CD) spectra were recorded on a JASCO J-715 CD Spectropolarimeter. The mass spectra and high resolution mass spectra were performed on a Q-TOF Micro mass spectrometer, resolution 5000. An isopropyl alcohol solution of sodium iodide (2 mg/mL) was used as a reference compound. Semi-preparative RP-HPLC was performed on an Agilent 1100 system equipped with a refractive index detector using an YMC Pack ODS-A column (particle size 5 μm, 250 × 10 mm). 

### 3.2. Animal Material

The South China Sea gorgonian coral *Dichotella gemmacea* (ZS-3, 3.5 kg, wet weight and ZH-1, 10.0 kg, wet weight) were collected from the South China Sea, in August 2007 and December 2011, and identified by Dr. Xiu-Bao Li, South China Sea Institute of Oceanology, Chinese Academy of Sciences. The voucher specimens (ZS-3, ZH-1) were deposited in the Second Military Medical University.

### 3.3. Extraction and Isolation

The frozen animals of ZS-3 (3.5 kg, wet weight) were extracted ultrasonically for three times with acetone and MeOH, respectively. The combined residue was partitioned between H_2_O and EtOAc to afford 16.1 g of EtOAc extract. The EtOAc extract was further partitioned between MeOH and hexane, affording 11.2 g of MeOH soluble residue. The MeOH extract was subjected to column chromatography (CC) on silica to give 16 fractions, using hexane/acetone (from 100:0 to 0:100) as eluent. Fraction 4 was further fractionated by RP-silical gel column chromatography (gradient elution from MeOH/H_2_O, 3:7 to MeOH, in 5% increments) and purified by HPLC (MeOH/H_2_O, 80:20, 1.5 mL/min) to yield **8** (2.5 mg, 49.2 min). Fraction 5 was subjected to repeated CCs on normal phase silica gel, Sephadex LH-20, and RP-silical gel, to give two subfractions 5-A and 5-B. Subfraction 5-A was purified by HPLC (MeOH/H_2_O, 75:25, 1.5 mL/min) to yield **2** (3.1 mg, 22.6 min) while subfraction 5-B was split by HPLC (MeOH/H_2_O, 77:23, 1.5 mL/min) into **12** (0.8 mg, 38.2 min), **10** (4.5 mg, 47.7 min) and **15** (1.8 mg, 50.8 min). Fraction 6 was chromomatographied over Sephadex LH-20 (CHCl_3_/MeOH, 1:1) to give four sub-fractions (A–D). HPLC purification on sub-fractions A (MeOH/H_2_O, 70:30, 1.5 mL/min) and B (MeOH/H_2_O, 70:30, 1.5 mL/min) gave **5** (2.7 mg, 25.7 min) and **6** (1.7 mg, 61.7 min), respectively. Fraction 7 was subjected to CCs on normal phase silica gel and Sephadex LH-20 to give two subfraction 7-A and 7-B. Further purification on both subfrations yielded **19** (4.3 mg, 51.7 min) from 7-A (MeOH/H_2_O, 70:30, 1.5 mL/min) and **3** (1.6 mg, 66.7 min) from7-B (eluent MeOH/H_2_O, 65:35, 1.5 mL/min). Fraction 9 was purified by HPLC (MeOH/H_2_O, 67:33, 1.5 mL/min) to yield **1** (1.6 mg, 24.5 min). Fraction 10 was chromatographied over normal phase silica gel and Sephadex LH-20 and then purified by HPLC (eluent MeOH/H_2_O, 70:30, 1.5 mL/min) yielding **18** (3.8 mg, 28.7 min). Fraction 11 was further fractionated by RP-silical gel CC (MeOH/H_2_O, 27:73 to 76:24) to give five subfractions (11A–11E). Subfraction 11C was purified by HPLC (eluent MeOH/H_2_O, 63:37, 1.5 mL/min) to yield **11** (1.1 mg, 26.8 min) and **14** (1.0 mg, 27.9 min). Subfraction 11D was purified by HPLC (MeOH/H_2_O, 72:28, 1.5 mL/min), yielding **4** (0.9 mg, 27.7 min) and **16** (1.0 mg, 28.1 min). Fraction 12 was further fractionated by normal phase silica gel CC (*n*-hexane/acetone, 4:1 to 1:1, in 5 increments) to give three subfractions (12A–12C). **13** (1.1 mg, 29.5 min) was obtained from subfraction 12B by HPLC (MeOH/H_2_O, 73:27, 1.5 mL/min). Fraction 13 was fractionated by RP-silical gel column chromatography (gradient elution from MeOH/H_2_O, 2:7 to 2:1, in 5% increments) to give 6 sub-fractions (13A–13F), a HPLC purification (eluent MeOH/H_2_O, 65:35, 1.5 mL/min) on 13E yielded **17** (4.6 mg, 32.6 min). 

The frozen animals of ZH-1 (10.0 kg, wet weight) were extracted partitioned using the above procedure to afford 20.0 g of Et_2_O extract. The residue was subjected to Sephadex LH-20 (CHCl_3_/MeOH, 1:1) give 8 fractions. Fraction 2 was subjected to reversed-phasesilica gel (gradient MeOH/H_2_O, from 1:9 to 4:1), followed by HPLC (MeOH/H_2_O, 65:35, 1.5 mL/min) to yield **9** (2.5 mg, 52.5 min), **20** (5.2 mg, 67.5 min). Fraction 3 was chromatographed on a silica gel column (gradient *n*-hexane/acetone, from 5:1 to 1:2) and HPLC (MeOH/H_2_O, 50:50, 1.5 mL/min) to yield **7** (5.5 mg, 69.2 min), **21** (3.5 mg, 16.1 min).

**Gemmacolide AA** (**1**): White amorphous powder; [α]^24^_D_ = −44 (*c* 0.24, CHCl_3_); UV (MeOH) λ_max_ (log ε) 206(1.85) nm; CD (CH_3_CN, *c* 2.0 × 10^−4^) λ_max_ (Δε) positive below 190 nm, 201.5(−7.26) nm; IR (film) ν_max_ 3470, 1775, 1741 cm^−1^; ^1^H and ^13^C NMR spectroscopic data, see Table 1, Table 2; ESI-MS *m*/*z* 691 [M + Na]^+^; HRESI-MS *m*/*z* 691.2150 [M + Na]^+^ (calcd. for C_31_H_40_O_16_Na, 691.2214).

**Gemmacolide AB** (**2**): White amorphous powder; [α]^24^_D_ = −52 (*c* 0.105, CHCl_3_); UV (MeOH) λ_max_ (log ε) 205(1.78) nm; CD (CH_3_CN, *c* 1.8 × 10^−4^) λ_max_ (Δε) positive below 190 nm, 216(−5.61) nm; IR (film) ν_max_ 3485, 1778, 1744 cm^−1^; ^1^H and ^13^C NMR spectroscopic data, see Table 1, Table 2; ESI-MS *m*/*z* 733 [M + Na]^+^; HRESI-MS *m*/*z* 733.2680 [M + Na]^+^ (calcd. for C_34_H_46_O_16_Na, 733.2680).

**Gemmacolide AC** (**3**): White amorphous powder; [α]^24^_D_ = −28 (*c* 0.04, CHCl_3_); UV (MeOH) λ_max_ (log ε) 205(1.71) nm; CD (CH_3_CN, *c* 2.6 × 10^−4^) λ_max_ (Δε) positive below 190 nm, 215.5(−6.41) nm; IR (film) ν_max_ 3467, 1774, 1742 cm^−1^; ^1^H and ^13^C NMR spectroscopic data, see Table 1, Table 2; ESI-MS *m*/*z* 717 [M + Na]^+^; HRESI-MS *m*/*z* 717.2737 [M + Na]^+^ (calcd. for C_34_H_46_O_15_Na, 717.2734).

**Gemmacolide AD** (**4**): White amorphous powder; [α]^24^_D_ = −11 (*c* 0.09, CHCl_3_); UV (MeOH) λ_max_ (log ε) 213(1.73) nm; CD (CH_3_CN, *c* 2.3 × 10^−4^) λ_max_ (Δε) positive below 197 nm, 212.5(−6.01) nm; IR (film) ν_max_ 3477, 1778, 1743 cm^−1^; ^1^H and ^13^C NMR spectroscopic data, see Table 1, Table 2; ESI-MS *m*/*z* 759 [M + Na]^+^; HRESI-MS *m*/*z* 759.3207 [M + Na]^+^ (calcd. for C_37_H_52_O_15_Na, 759.3204).

**Gemmacolide AE** (**5**): White amorphous powder; [α]^24^_D_ = −5 (*c* 0.085, CHCl_3_); UV (MeOH) λ_max_ (log ε) 204(1.41) nm; CD (CH_3_CN, *c* 3.0 × 10^−4^) λ_max_ (Δε) positive below 190 nm, 209(−6.01) nm; IR (film) ν_max_ 3483, 1777, 1742 cm^−1^; ^1^H and ^13^C NMR spectroscopic data, see Table 1, Table 2; ESI-MS *m*/*z* 659 [M + Na]^+^; HRESI-MS *m*/*z* 659.2676 [M + Na]^+^ (calcd. for C_32_H_44_O_13_Na, 659.2680).

**Gemmacolide AF** (**6**): White amorphous powder; [α]^24^_D_ = −40 (*c* 0.085, CHCl_3_); UV (MeOH) λ_max_ (log ε) 204(1.36) nm; CD (CH_3_CN, *c* 1.6 × 10^−4^) λ_max_ (Δε) positive below 190 nm, 200(−10.53) nm; IR (film) ν_max_ 3462, 1776, 1743 cm^−1^; ^1^H and ^13^C NMR spectroscopic data, see Table 1, Table 3; ESI-MS *m*/*z* 787 [M + Na]^+^; HRESI-MS *m*/*z* 787.3150 [M + Na]^+^ (calcd. for C_38_H_52_O_16_Na, 787.3153).

**Gemmacolide AG** (**7**): White amorphous powder; [α]^24^_D_ = −26.9 (*c* 0.17, CHCl_3_); UV (MeOH) λ_max_ (log ε) 209(1.65) nm; CD (CH_3_CN, *c* 2.3 × 10^−4^) λ_max_ (Δε) positive below 190 nm, 202.0(−10.05) nm; IR (film) ν_max_ 3473, 1778, 1738 cm^−1^; ^1^H and ^13^C NMR spectroscopic data, seeTable 1, Table 3; ESI-MS *m*/*z* 745 [M + Na]^+^; HRESI-MS *m*/*z* 745.2686 [M + Na]^+^ (calcd. for C_35_H_46_O_16_Na, 745.2684).

**Gemmacolide AH** (**8**): White amorphous powder; [α]^24^_D_ = −11 (*c* 0.035, CHCl_3_); UV (MeOH) λ_max_ (log ε) 206(1.88) nm; CD (CH_3_CN, *c* 2.8 × 10^−4^) λ_max_ (Δε) positive below 190 nm, 199.0(−11.96) nm; IR (film) ν_max_ 3469, 1775, 1744 cm^−1^; ^1^H and ^13^C NMR spectroscopic data, seeTable 1, Table 3; ESI-MS *m*/*z* 887 [M + Na]^+^; HRESI-MS *m*/*z* 887.3673 [M + Na]^+^ (calcd. for C_43_H_60_O_18_Na, 887.3677). 

**Gemmacolide AI** (**9**): White amorphous powder; [α]^24^_D_ = −21 (*c* 0.25, CHCl_3_); UV (MeOH) λ_max_ (log ε) 212(1.72) nm; CD (CH_3_CN, *c* 2.5 × 10^−4^) λ_max_ (Δε) positive below 190 nm, 204.5(−12.5) nm; IR (film) ν_max_ 3469, 1778, 1741 cm^−1^; ^1^H and ^13^C NMR spectroscopic data, seeTable 1, Table 3; ESI-MS *m*/*z* 815 [M + Cl]^−^; HRESI-MS *m*/*z* 815.2888 [M + Cl]^−^ (calcd. for C_38_H_52_ClO_17_, 815.2893).

**Gemmacolide AJ** (**10**): White amorphous powder; [α]^24^_D_ = −49 (*c* 0.10, CHCl_3_); UV (MeOH) λ_max_ (log ε) 204(1.49) nm; CD (CH_3_CN, *c* 3.1 × 10^−4^) λ_max_ (Δε) positive below 190 nm, 203(−13.32) nm; IR (film) ν_max_ 3474, 1778, 1744 cm^−1^; ^1^H and ^13^C NMR spectroscopic data, see Table 3, Table 4; ESI-MS *m*/*z* 821 [M + Na]^+^; HRESI-MS *m*/*z* 821.2767 [M + Na]^+^ (calcd. for C_38_H_51_O_16_NaCl, 821.2763). 

**Gemmacolide AK** (**11**): White amorphous powde; [α]^24^_D_ = −11.5 (*c* 0.11, CHCl_3_); UV (MeOH) λ_max_ (log ε) 201(1.16) nm; CD (CH_3_CN, *c* 3.4 × 10^−4^) λ_max_ (Δε) positive below 198 nm, 210.5(−6.89) nm; IR (film) ν_max_ 3478, 1778, 1744 cm^−1^; ^1^H and ^13^C NMR spectroscopic data, see Table 3, Table 4; ESI-MS *m*/*z* 733 [M + Na]^+^; HRESI-MS *m*/*z* 733.2690 [M + Na]^+^ (calcd. for C_34_H_46_O_16_Na, 733.2684).

**Gemmacolide AL** (**12**): White amorphous powder; [α]^24^_D_ = −36 (*c* 0.10, CHCl_3_); UV (MeOH) λ_max_ (log ε) 205 (1.78) nm; CD (CH_3_CN, *c* 3.3 × 10^−4^) λ_max_ (Δε) positive below 190 nm, 205(−8.58) nm; IR (film) ν_max_ 3475, 1776, 1745 cm^−1^; ^1^H and ^13^C NMR spectroscopic data, see Table 4, Table 5; ESI-MS *m*/*z* 817 [M + Na]^+^; HRESI-MS *m*/*z* 817.3252 [M + Na]^+^ (calcd. for C_39_H_54_O_17_Na, 817.3259).

**Gemmacolide AM** (**13**): White amorphous powder; [α]^24^_D_ = −11.3 (*c* 0.11, CHCl_3_); UV (MeOH) λ_max_ (log ε) 206(1.55) nm; CD (CH_3_CN, *c* 3.2 × 10^−4^) λ_max_ (Δε) positive below 190 nm, 199(−4.05) nm; IR (film) ν_max_ 3468, 1779, 1731 cm^−1^; ^1^H and ^13^C NMR spectroscopic data, see Table 4, Table 5; ESI-MS *m*/*z* 833 [M + Cl]^−^, HRESI-MS *m*/*z* 833.2549 [M + Cl]^−^ (calcd. for C_38_H_51_Cl_2_O_16_ 833.2554).

**Gemmacolide AN** (**14**): White amorphous powder; [α]^24^_D_ = −14.8 (*c* 0.10, CHCl_3_); UV (MeOH) λ_max_ (log ε) 205(1.58) nm; CD (CH_3_CN, *c* 3.3 × 10^−4^) λ_max_ (Δε) positive below 190 nm, 203.5(−4.02) nm; IR (film) ν_max_ 3465, 1778, 1738 cm^−1^; ^1^H and ^13^C NMR spectroscopic data, see Table 4, Table 5; ESI-MS *m*/*z* 737 [M + Na]^+^; HRESI-MS *m*/*z* 737.2182 [M + Na]^+^ (calcd. for C_33_H_43_O_15_ClNa, 737.2188).

**Gemmacolide AO** (**15**): White amorphous powder; [α]^24^_D_ = −39 (*c* 0.08, CHCl_3_); UV (MeOH) λ_max_ (log ε) 204(1.52) nm; CD (CH_3_CN, *c* 1.5 × 10^−4^) λ_max_ (Δε) positive below 190 nm, 204.5(−13.93) nm; IR (film) ν_max_ 3468, 1778, 1743 cm^−1^; ^1^H and ^13^C NMR spectroscopic data, see Table 4, Table 5; ESI-MS *m*/*z* 845 [M + Na]^+^; HRESI-MS *m*/*z* 845.3575 [M + Na]^+^ (calcd. for C_41_H_58_O_17_Na, 845.3572).

**Gemmacolide AP** (**16**): White amorphous powder; [α]^24^_D_ = −51.3 (*c* 0.10, CHCl_3_); UV (MeOH) λ_max_ (log ε) 214(1.89) nm; CD (CH_3_CN, *c* 2.0 × 10^−4^) λ_max_ (Δε) positive below 195 nm, 203.5(−2.25) nm; IR (film) ν_max_ 3478, 1778, 1743 cm^−1^; ^1^H and ^13^C NMR spectroscopic data, see Table 4, Table 5; ESI-MS *m*/*z* 749 [M + Cl]^−^; HRESI-MS *m*/*z* 749.1981 [M + Cl]^−^ (calcd. for C_33_H_43_Cl_2_O_15_, 749.1979).

**Gemmacolide AQ** (**17**): White amorphous powder; [α]^24^_D_ = −36 (*c* 0.175, CHCl_3_); UV (MeOH) λ_max_ (log ε) 204(1.41) nm; CD (CH_3_CN, *c* 2.1 × 10^−4^) λ_max_ (Δε) positive below 190 nm, 200(−4.85) nm; IR (film) ν_max_ 3479, 1770, 1743 cm^−1^; ^1^H and ^13^C NMR spectroscopic data, see Table 4, Table 5; ESI-MS *m*/*z* 703 [M + Na]^+^; HRESI-MS *m*/*z* 703.2572 [M + Na]^+^ (calcd. for C_33_H_44_O_15_Na, 703.2578).

**Gemmacolide AR** (**18**): White amorphous powder; [α]^24^_D_ = −38 (*c* 0.08, CHCl_3_); UV (MeOH) λ_max_ (log ε) 210(2.54) nm; CD (CH_3_CN, *c* 8.8 × 10^−4^) λ_max_ (Δε) positive below 190 nm, 198(−4.11) nm; IR (film) ν_max_ 3469, 1777, 1742 cm^−1^; ^1^H and ^13^C NMR spectroscopic data, see Table 4, Table 5; ESI-MS *m*/*z* 803 [M + Na]^+^; HRESI-MS *m*/*z* 745.2689 [M + Na]^+^ (calcd. for C_35_H_46_O_16_Na, 745.2684).

### 3.4. Cytotoxicity Assay

Cytotoxicity was tested against human lung adenocarcinoma (A549) and human osteosarcoma cell (MG63), using a modification of the MTT (3-(4,5-dimethylthiazol-2-yl)-2,5-diphenyltetrazolium bromide) colorimetric method [18]. Adriamycin was used as positive control, IC_50_ = 2.8 μM for A549 cells and 3.2 μM for MG63 cells.

## 4. Conclusions

The observation of the potent activity of gemmacolides in tumor cell growth inhibition [10,12] promotes the systematic study on briarane diterpenoids regarding their chemistry and bioactivities, leadindg to the isolation and structural elucidation of twenty-one 11,20-epoxy-3*Z*,5*E*-dien briaranes from the South China Sea gorgonian *Dichotella gemmacea*. In the *in vitro* bioassay, compounds **1**–**3**, **5**, **6**, **8**–**12**, and **14**–**19** exhibited different levels of growth inhibition activity against A549 and MG63 cell lines. Preliminary structure-activity analysis suggests that 12-*O*-isovalerate may increase the activity whereas 13- or 14-*O*-isovalerate may decrease the activity. Contribution of substitutions at C-2 and C-16 remains uncertain. The interesting discovery may encourage further investigations on the chemistry and tumor cell growth inhibitory activity of the cluster of metabolites.

## Supplementary Files

Supplementary File 1 Supplementary Information (PDF, 5867 KB)

## References

[1] Blunt J.W., Copp B.R., Keyzers R.A., Munro M.H.G., Prinsep M.R. (2013). Marine natural products. Nat. Prod. Rep..

[2] Berrue F., Kerr R.G. (2009). Diterpenes from gorgonian corals. Nat. Prod. Rep..

[3] Sung P.J., Su J.H., Wang W.H., Sheu J.H., Fang L.S., Wu Y.C., Chen Y.H., Chung H.M., Su Y.D., Chang Y.C. (2011). Survey of briarane-type diterpenoids—Part IV. Heterocycles.

[4] Zhang W., Guo Y.W., Gu Y.C. (2006). Secondary metabolites from the South China Sea invertebrateschemistry andbiological activity. Curr. Med. Chem..

[5] Sun J.F., Han Z., Zhou X.F., Yang B., Lin X., Liu J., Peng Y., Yang X.W., Liu Y.H. (2013). Antifouling briarane type diterpenoids from South China Sea gorgonians *Dichotella gemmacea*. Tetrahedron.

[6] Liu T.F., Lu X., Tang H., Zhang M.M., Wang P., Sun P., Liu Z.Y., Wang Z.L., Li L., Rui Y.C. (2013). 3β,5α,6β-Oxigenated sterols from the South China Sea gorgonian *Muriceopsis flavida* and their tumor cell growth inhibitory activity and apoptosis-inducing function. Steroids.

[7] Wang Z.L., Zhang H.Y., Yuan W.H., Gong W., Tang H., Liu B.S., Krohn K., Yi Y.H., Zhang W. (2012). Antifungal nortriterpene and triterpene glycosides from the sea cucumber *Apostichopus japonicus* Selenka. Food Chem..

[8] Sun P., Meng L.Y., Tang H., Liu B.S., Li L., Yi Y.H., Zhang W. (2012). Sinularosides A and B, bioactive 9,11-secosteroidal glycosides from the South China Sea soft coral *Sinularia* sp.. J. Nat. Prod..

[9] Geng W.L., Wang X.Y., Kurtán T., Mándi A., Tang H., Schulz B., Sun P., Zhang W. (2012). Herbarone, a rearranged heptaketide derivative from the sea hare associated fungus *Torula herbarum*. J. Nat. Prod..

[10] Li C., La M.P., Li L., Li X.B., Tang H., Liu B.S., Krohn K., Sun P., Yi Y.H., Zhang W. (2011). Bioactive 11,20-epoxy-3,5(16)-diene briarane diterpenoids from the South China Sea gorgonian *Dichotella gemmacea*. J. Nat. Prod..

[11] Li C., La M.P., Sun P., Kurtán T., Mándi A., Tang H., Liu B.S., Yi Y.H., Li L., Zhang W. (2011). Bioactive (3*Z*,5*E*)-11,20-epoxybriara-3,5-diene-7,18-olide diterpenoids from the South China Sea gorgonian *Dichotella gemmacea*. Mar. Drugs.

[12] Li C., La M.P., Tang H., Pan W.H., Sun P., Yi Y.H., Zhang W. (2012). Bioactive briaranes from the South China Sea gorgonian *Dichotella gemmacea*. Bioorg. Med. Chem. Lett..

[13] Roe M.B., Whittaker M., Procter G. (1995). Studies on the synthesis of solenolide F; a Cr(II)-mediated cyclization to form the ten-membered ring. Tetrahedron Lett..

[14] Balasubramaniam R.P., Moss D.K., Wyatt J.K., Spence J.D., Gee A., Nantz M.H. (1997). Methylation-ring opening of 3,3-disubstituted 2,3-epoxy alcohols. Synthesis of chiral quaternary fragments for assembly of briaran diterpenes. Tetrahedron.

[15] Rodriguez A.D., Ramirez C., Cobar O.M. (1996). Briareins C–L, 10 new briarane diterpenoids from the common Caribbean gorgonian *Briareum asbestinum*. J. Nat. Prod..

[16] Iwasaki J., Ito H., Nakamura M., Iguch K. (2006). A synthetic study of briarane-type marine diterpenoid, pachyclavulide B. Tetrahedron Lett..

[17] Shen Y.C., Lin Y.C., Ko C.L., Wang L.T. (2003). New briaranes from the Taiwanese gorgonian *Junceella juncea*. J. Nat. Prod..

[18] Mosmann T.J. (1983). Rapid colorimetric assay for cellular growth and survival: Application to proliferation and cytotoxicity assays. J. Immunol. Methods.

