# Supplementary material for: Chemistry and Tumor Cell Growth Inhibitory Activity of 11,20-Epoxy-3Z,5(6)E-diene Briaranes from the South China Sea Gorgonian Dichotella gemmacea
Source: Mar Drugs. 2013 May 15;11(5):1565–82. doi: 10.3390/md11051565 (PMC3707162; doi:10.3390/md11051565)

Supplementary Information

	Table of Contents	P1–P4
1	Spectra of the new compound **1**	P5–P8
	HR-ESIMS spectrum of the new compound **1**	P5
	^{1}H NMR spectrum of the new compound **1**	P5
	^{13}C NMR spectrum of the new compound **1**	P6
	DEPT spectrum of the new compound **1**	P6
	HSQC spectrum of the new compound **1**	P7
	^{1}H-^{1}H COSY spectrum of the new compound **1**	P7
	HMBC spectrum of the new compound **1**	P8
	NOESY spectrum of the new compound **1**	P8
2	Spectra of the new compound **2**	P9–P12
	HR-ESIMS spectrum of the new compound **2**	P9
	^{1}H NMR spectrum of the new compound **2**	P9
	^{13}C NMR spectrum of the new compound **2**	P10
	DEPT spectrum of the new compound **2**	P10
	HSQC spectrum of the new compound **2**	P11
	^{1}H-^{1}H COSY spectrum of the new compound **2**	P11
	HMBC spectrum of the new compound **2**	P12
	NOESY spectrum of the new compound **2**	P12
3	Spectra of the new compound **3**	P13–P16
	HR-ESIMS spectrum of the new compound **3**	P13
	^{1}H NMR spectrum of the new compound **3**	P13
	^{13}C NMR spectrum of the new compound **3**	P14
	DEPT spectrum of the new compound **3**	P14
	HSQC spectrum of the new compound **3**	P15
	^{1}H-^{1}H COSY spectrum of the new compound **3**	P15
	HMBC spectrum of the new compound **3**	P16
	NOESY spectrum of the new compound **3**	P16
4	Spectra of the new compound **4**	P17–P20
	HR-ESIMS spectrum of the new compound **4**	P17
	^{1}H NMR spectrum of the new compound **4**	P17
	^{13}C NMR spectrum of the new compound **4**	P18
	DEPT spectrum of the new compound **4**	P18
	HSQC spectrum of the new compound **4**	P19
	^{1}H-^{1}H COSY spectrum of the new compound **4**	P19
	HMBC spectrum of the new compound **4**	P20
	NOESY spectrum of the new compound **4**	P20
5	Spectra of the new compound **5**	P21–P24
	HR-ESIMS spectrum of the new compound **5**	P21
	^{1}H NMR spectrum of the new compound **5**	P21
	^{13}C NMR spectrum of the new compound **5**	P22

	DEPT spectrum of the new compound **5**	P22
	HSQC spectrum of the new compound **5**	P23
	^{1}H-^{1}H COSY spectrum of the new compound **5**	P23
	HMBC spectrum of the new compound **5**	P24
	NOESY spectrum of the new compound **5**	P24
6	Spectra of the new compound **6**	P25–P28
	HR-ESIMS spectrum of the new compound **6**	P25
	^{1}H NMR spectrum of the new compound **6**	P25
	^{13}C NMR spectrum of the new compound **6**	P26
	DEPT spectrum of the new compound **6**	P26
	HSQC spectrum of the new compound **6**	P27
	^{1}H-^{1}H COSY spectrum of the new compound **6**	P27
	HMBC spectrum of the new compound **6**	P28
	NOESY spectrum of the new compound **6**	P28
7	Spectra of the new compound **7**	P29–P32
	HR-ESIMS spectrum of the new compound **7**	P29
	^{1}H NMR spectrum of the new compound **7**	P29
	^{13}C NMR spectrum of the new compound **7**	P30
	DEPT spectrum of the new compound **7**	P30
	HSQC spectrum of the new compound **7**	P31
	^{1}H-^{1}H COSY spectrum of the new compound **7**	P31
	HMBC spectrum of the new compound **7**	P32
	NOESY spectrum of the new compound **7**	P32
8	Spectra of the new compound **8**	P33–P36
	HR-ESIMS spectrum of the new compound **8**	P33
	^{1}H NMR spectrum of the new compound **8**	P33
	^{13}C NMR spectrum of the new compound **8**	P34
	DEPT spectrum of the new compound **8**	P34
	HSQC spectrum of the new compound **8**	P35
	^{1}H-^{1}H COSY spectrum of the new compound **8**	P35
	HMBC spectrum of the new compound **8**	P36
	NOESY spectrum of the new compound **8**	P36
9	Spectra of the new compound **9**	P37–P40
	HR-ESIMS spectrum of the new compound **9**	P37
	^{1}H NMR spectrum of the new compound **9**	P37
	^{13}C NMR spectrum of the new compound **9**	P38
	DEPT spectrum of the new compound **9**	P38
	HSQC spectrum of the new compound **9**	P39
	^{1}H-^{1}H COSY spectrum of the new compound **9**	P39
	HMBC spectrum of the new compound **9**	P40
	NOESY spectrum of the new compound **9**	P40
10	Spectra of the new compound **10**	P41–P44

	HR-ESIMS spectrum of the new compound **10**	P41
	^{1}H NMR spectrum of the new compound **10**	P41
	^{13}C NMR spectrum of the new compound **10**	P42
	DEPT spectrum of the new compound **10**	P42
	HSQC spectrum of the new compound **10**	P43
	^{1}H-^{1}H COSY spectrum of the new compound **10**	P43
	HMBC spectrum of the new compound **10**	P44
	NOESY spectrum of the new compound **10**	P44
11	Spectra of the new compound **11**	P45–P48
	HR-ESIMS spectrum of the new compound **11**	P45
	^{1}H NMR spectrum of the new compound **11**	P45
	^{13}C NMR spectrum of the new compound **11**	P46
	DEPT spectrum of the new compound **11**	P46
	HSQC spectrum of the new compound **11**	P47
	^{1}H-^{1}H COSY spectrum of the new compound **11**	P47
	HMBC spectrum of the new compound **11**	P48
	NOESY spectrum of the new compound **11**	P48
12	Spectra of the new compound **12**	P49–P52
	HR-ESIMS spectrum of the new compound **12**	P49
	^{1}H NMR spectrum of the new compound **12**	P49
	^{13}C NMR spectrum of the new compound **12**	P50
	DEPT spectrum of the new compound **12**	P50
	HSQC spectrum of the new compound **12**	P51
	^{1}H-^{1}H COSY spectrum of the new compound **12**	P51
	HMBC spectrum of the new compound **12**	P52
	NOESY spectrum of the new compound **12**	P52
13	Spectra of the new compound **13**	P53–P56
	HR-ESIMS spectrum of the new compound **13**	P53
	^{1}H NMR spectrum of the new compound **13**	P53
	^{13}C NMR spectrum of the new compound **13**	P54
	DEPT spectrum of the new compound **13**	P54
	HSQC spectrum of the new compound **13**	P55
	^{1}H-^{1}H COSY spectrum of the new compound **13**	P55
	HMBC spectrum of the new compound **13**	P56
	NOESY spectrum of the new compound **13**	P56
14	Spectra of the new compound **14**	P57–P60
	HR-ESIMS spectrum of the new compound **14**	P57
	^{1}H NMR spectrum of the new compound **14**	P57
	^{13}C NMR spectrum of the new compound **14**	P58
	DEPT spectrum of the new compound **14**	P58
	HSQC spectrum of the new compound **14**	P59
	^{1}H-^{1}H COSY spectrum of the new compound **14**	P59

	HMBC spectrum of the new compound **14**	P60
	NOESY spectrum of the new compound **14**	P60
15	Spectra of the new compound **15**	P61–P64
	HR-ESIMS spectrum of the new compound **15**	P61
	^{1}H NMR spectrum of the new compound **15**	P61
	^{13}C NMR spectrum of the new compound **15**	P62
	DEPT spectrum of the new compound **15**	P62
	HSQC spectrum of the new compound **15**	P63
	^{1}H-^{1}H COSY spectrum of the new compound **15**	P63
	HMBC spectrum of the new compound **15**	P64
	NOESY spectrum of the new compound **15**	P64
16	Spectra of the new compound **16**	P65–P68
	HR-ESIMS spectrum of the new compound **16**	P65
	^{1}H NMR spectrum of the new compound **16**	P65
	^{13}C NMR spectrum of the new compound **16**	P66
	DEPT spectrum of the new compound **16**	P66
	HSQC spectrum of the new compound **16**	P67
	^{1}H-^{1}H COSY spectrum of the new compound **16**	P67
	HMBC spectrum of the new compound **16**	P68
	NOESY spectrum of the new compound **16**	P68
17	Spectra of the new compound **17**	P69–P72
	HR-ESIMS spectrum of the new compound **17**	P69
	^{1}H NMR spectrum of the new compound **17**	P69
	^{13}C NMR spectrum of the new compound **17**	P70
	DEPT spectrum of the new compound **17**	P70
	HSQC spectrum of the new compound **17**	P71
	^{1}H-^{1}H COSY spectrum of the new compound **17**	P71
	HMBC spectrum of the new compound **17**	P72
	NOESY spectrum of the new compound **17**	P72
18	Spectra of the new compound **18**	P73–P76
	HR-ESIMS spectrum of the new compound **18**	P73
	^{1}H NMR spectrum of the new compound **18**	P73
	^{13}C NMR spectrum of the new compound **18**	P74
	DEPT spectrum of the new compound **18**	P74
	HSQC spectrum of the new compound **18**	P75
	^{1}H-^{1}H COSY spectrum of the new compound **18**	P75
	HMBC spectrum of the new compound **18**	P76
	NOESY spectrum of the new compound **18**	P76

Figure S1. HR-ESIMS spectrum of the new compound **1**.

Tolerance = 10.0 PPM / DBE: min = -1.5, max = 50.0
Selected filters: None

Monoisotopic Mass, Even Electron Ions
32 formula(e) evaluated with 2 results within limits (up to 50 closest results for each mass)
Elements Used:
C: 5-38 H: 1-50 O: 5-18

SIPI Q-Tof micro 24-Jun-2010,13:40:34
LC-34 M.W=668 YA019 0.00000000
Q101286NH 12 (0.415) AM (Cen,6, 80.00, Ar,5000.0,644.24,0.70); Sm (SG, 2x3.00); Cm (8:46) TOF MS ES- 7.45e4

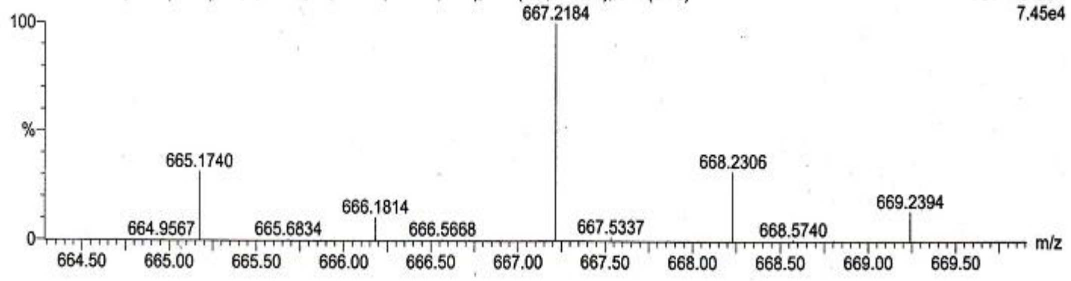

Minimum:	35.00				-1.5		
Maximum:	100.00		5.0	10.0	50.0		

Mass	RA	Calc. Mass	mDa	PPM	DBE	i-FIT	Formula
667.2184	100.00	667.2179	0.5	0.7	21.5	1231.0	√C38 H35 O11
		667.2238	-5.4	-8.1	12.5	627.2	C31 H39 O16

Figure S2. ^{1}H MNR (600 MHz, $CDCl_3$) spectrum of the new compound **1**.

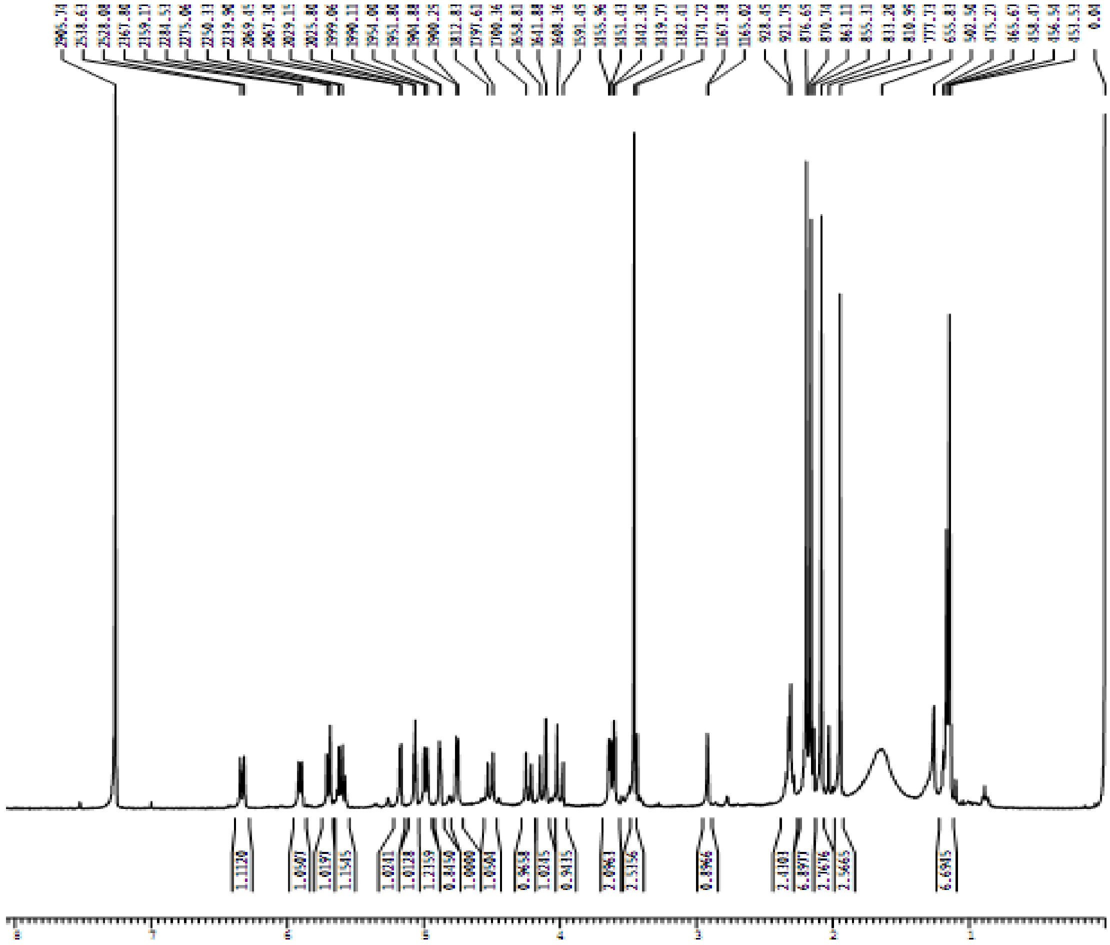

Figure S3. ^{13}C MNR (150 MHz, $CDCl_3$) spectrum of the new compound **1**.

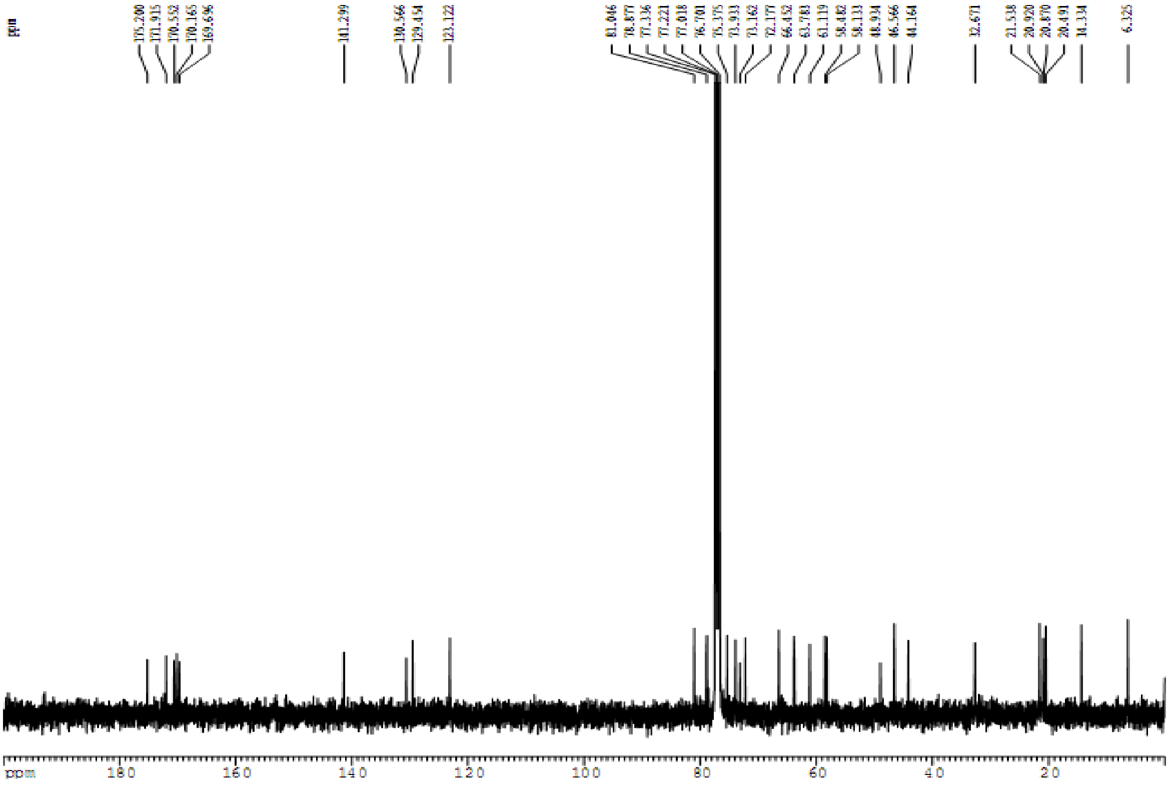

Figure S4. DEPT spectrum of the new compound **1**.

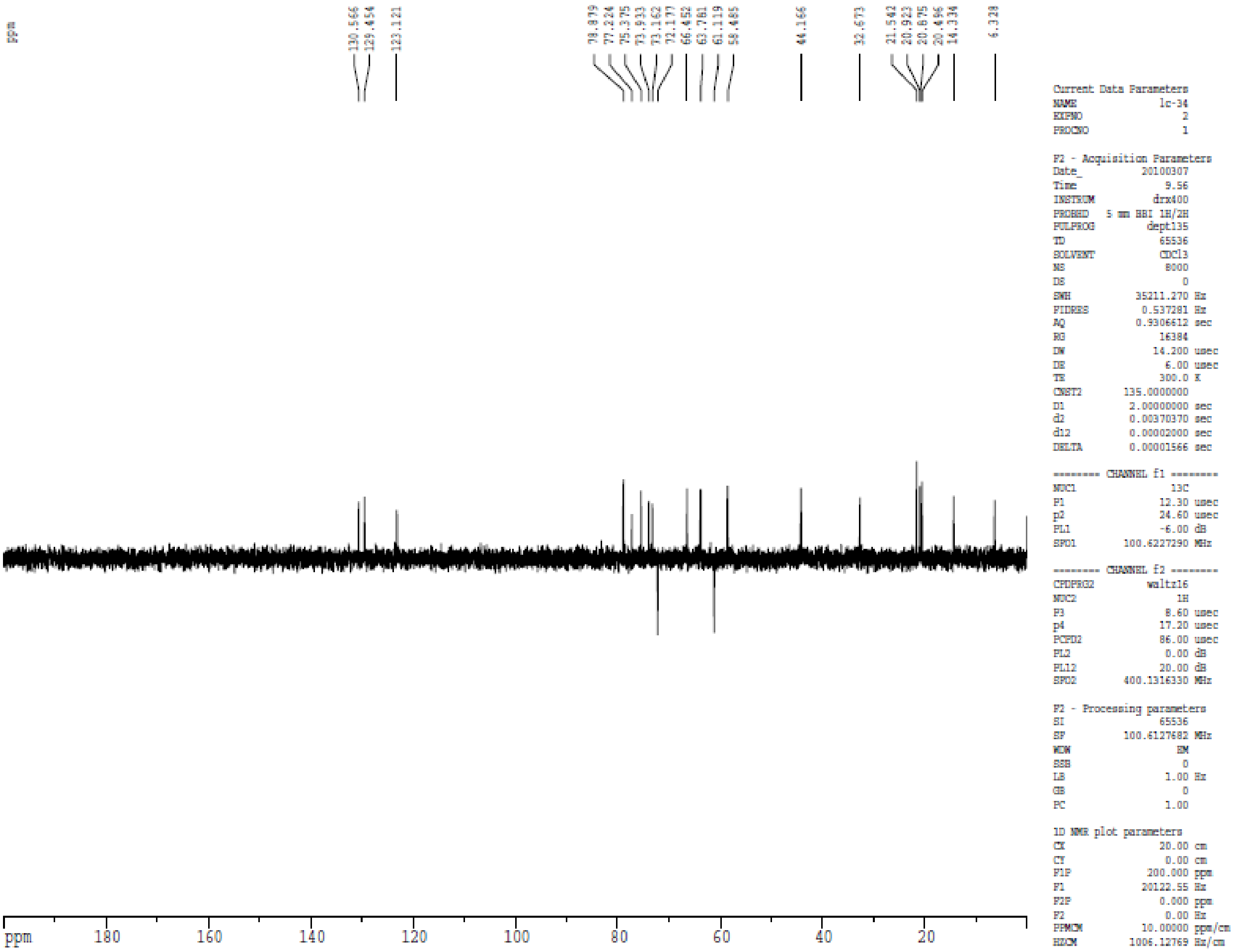

Figure S5. HSQC spectrum of the new compound **1**.

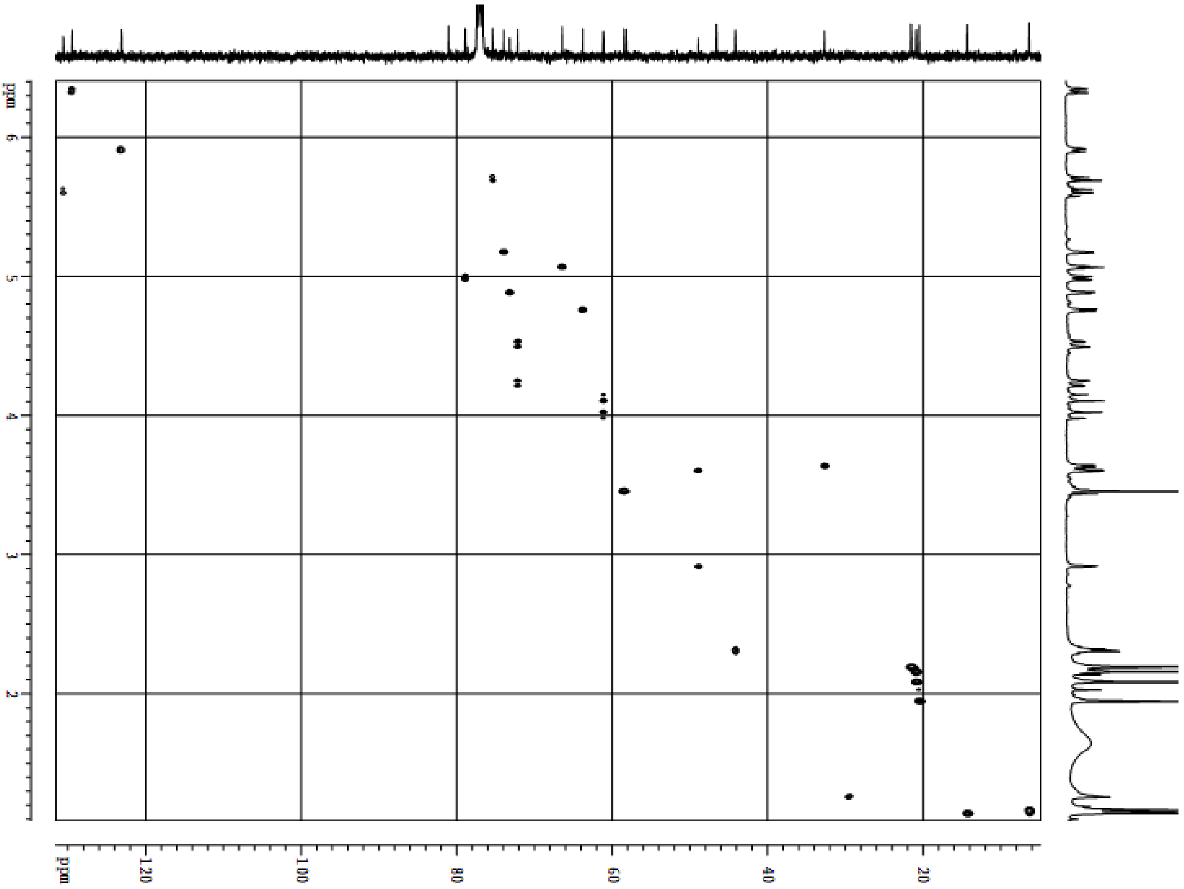

Figure S6. ^{1}H-^{1}H COSY spectrum of the new compound **1**.

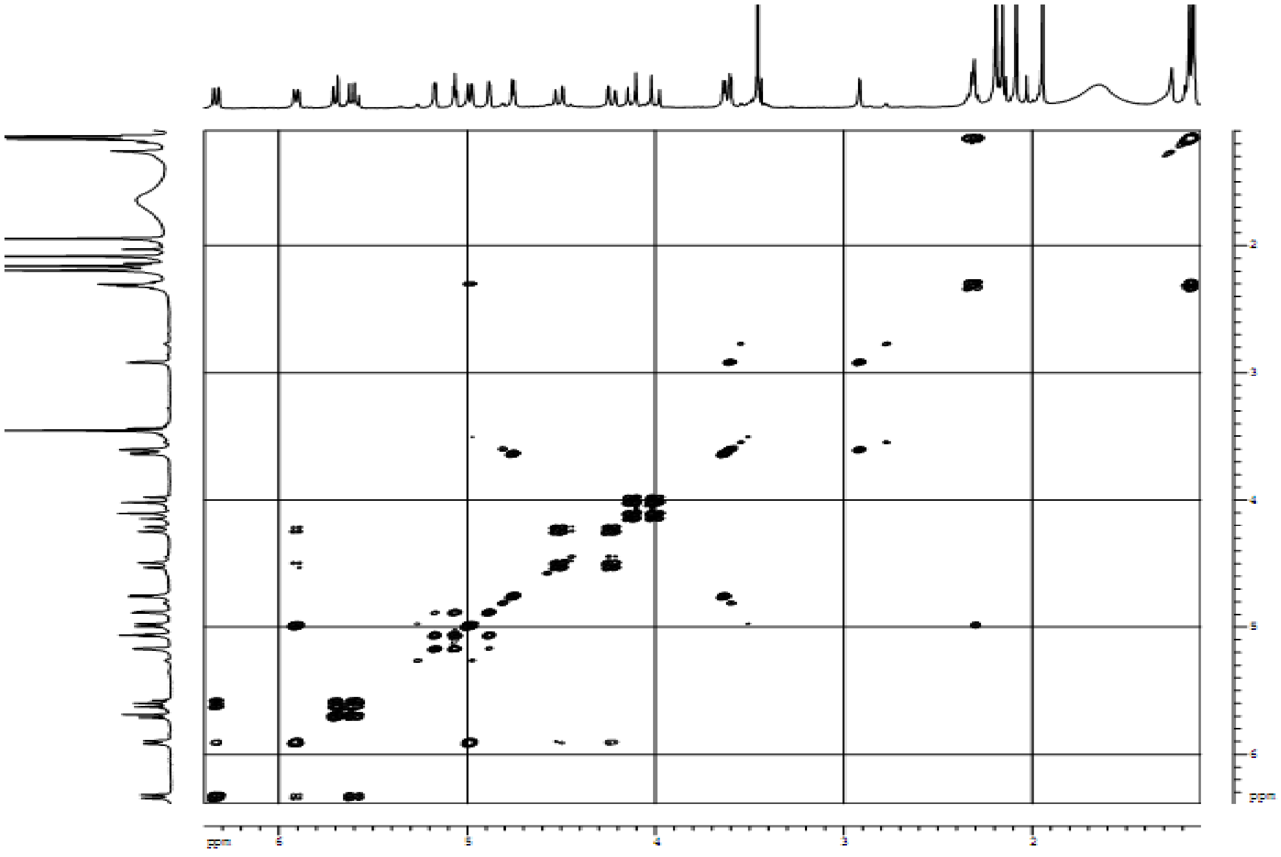

Figure S7. HMBC spectrum of the new compound **1**.

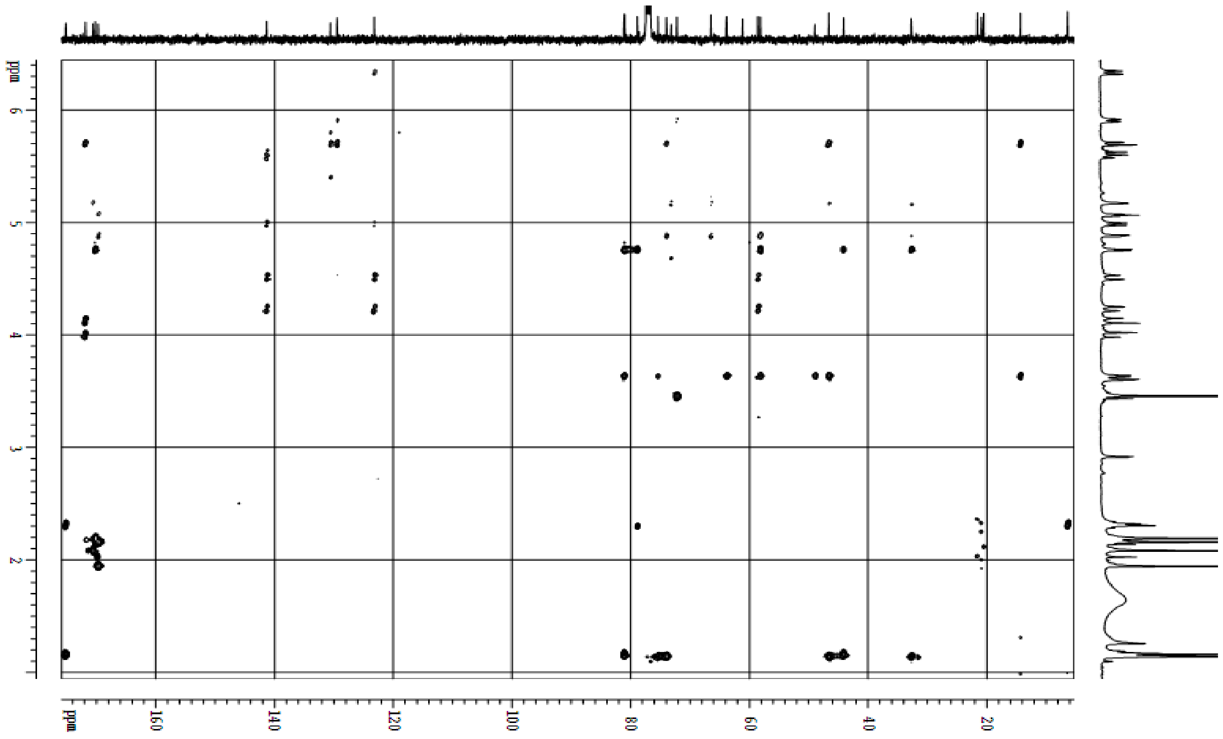

Figure S8. NOESY spectrum of the new compound **1**.

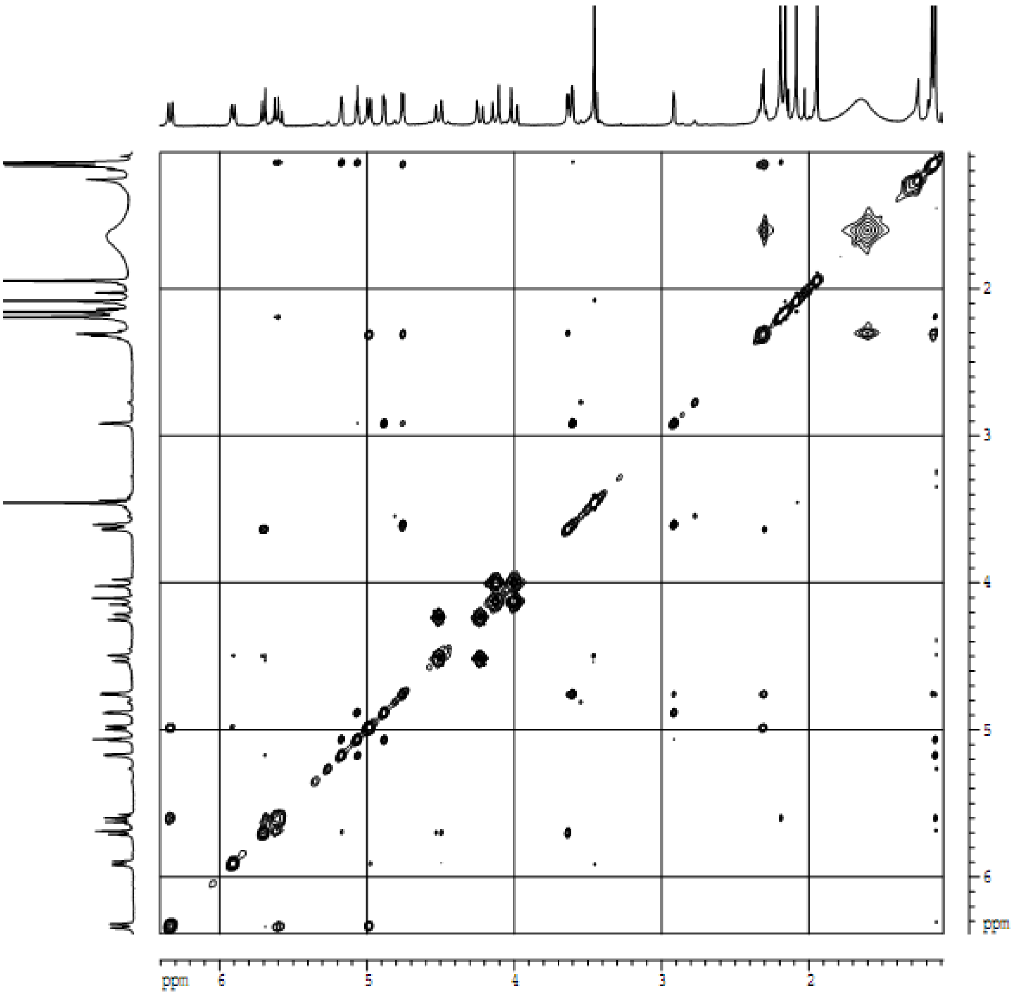

Figure S9. HR-ESIMS spectrum of the new compound **2**.

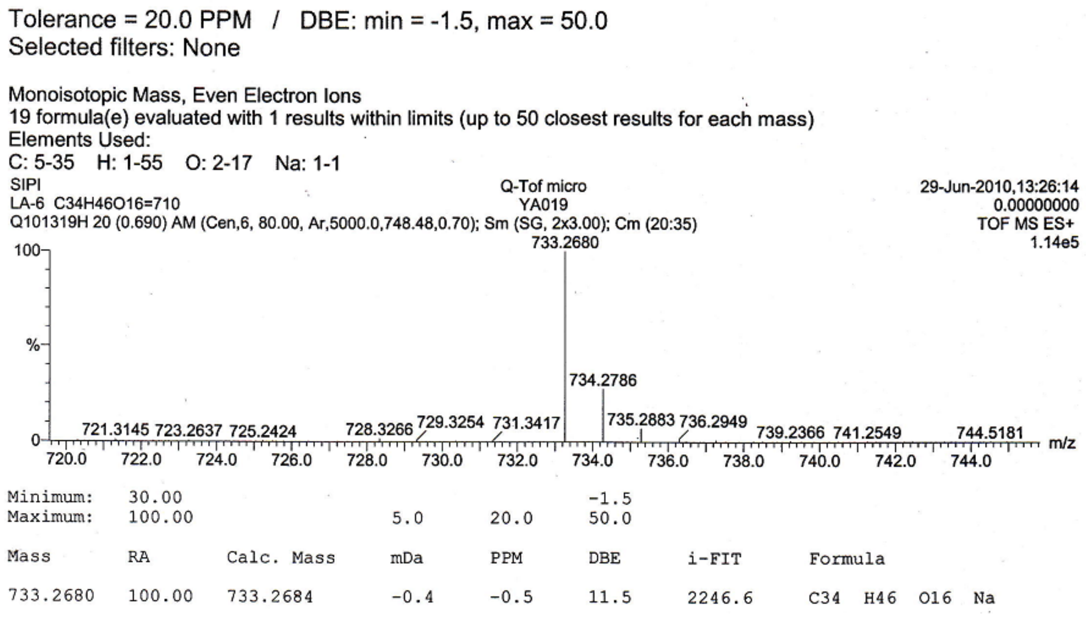

Figure S10. ^{1}H MNR (400 MHz, $CDCl_3$) spectrum of the new compound **2**.

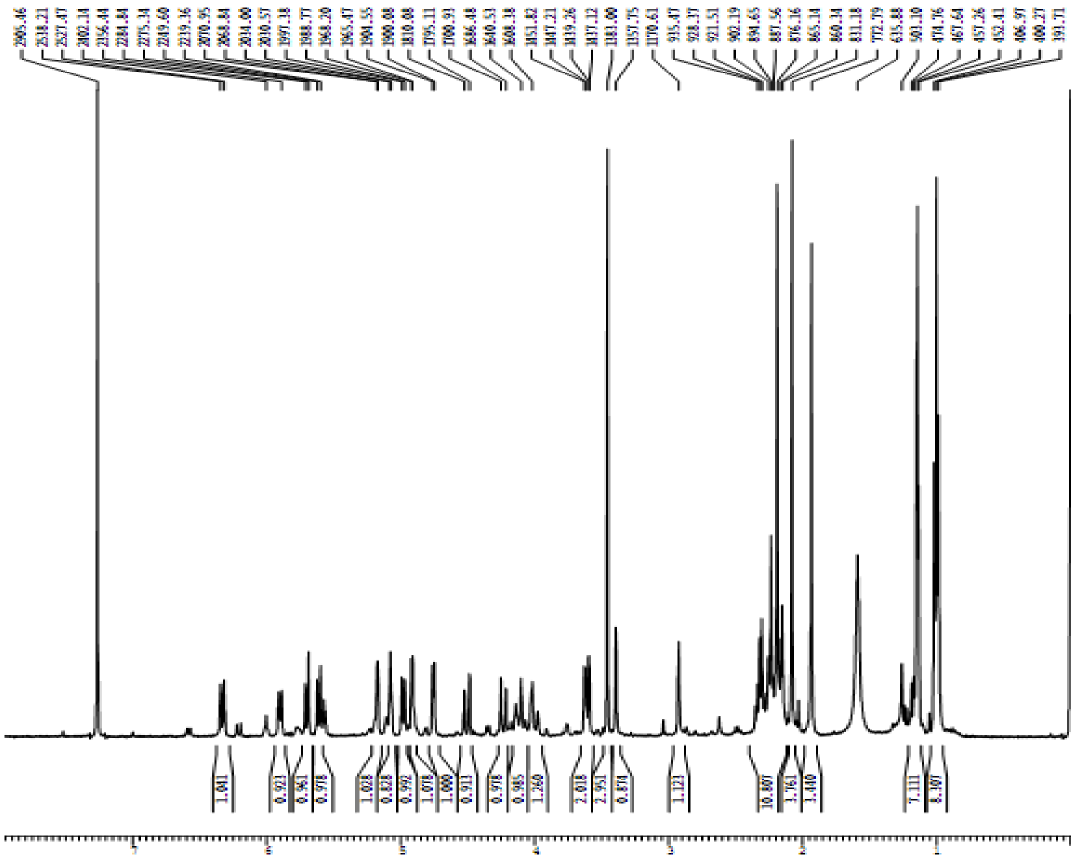

Figure S11. ^{13}C MNR (125 MHz, $CDCl_3$) spectrum of the new compound **2**.

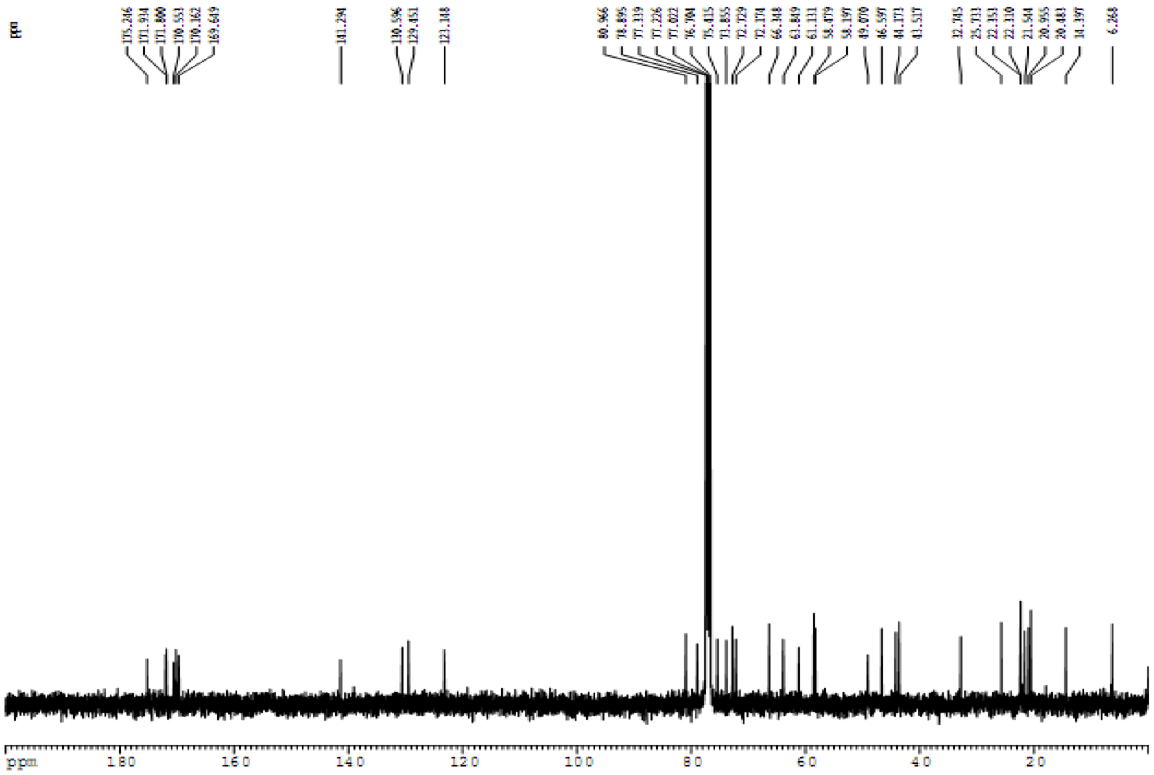

Figure S12. DEPT spectrum of the new compound **2**.

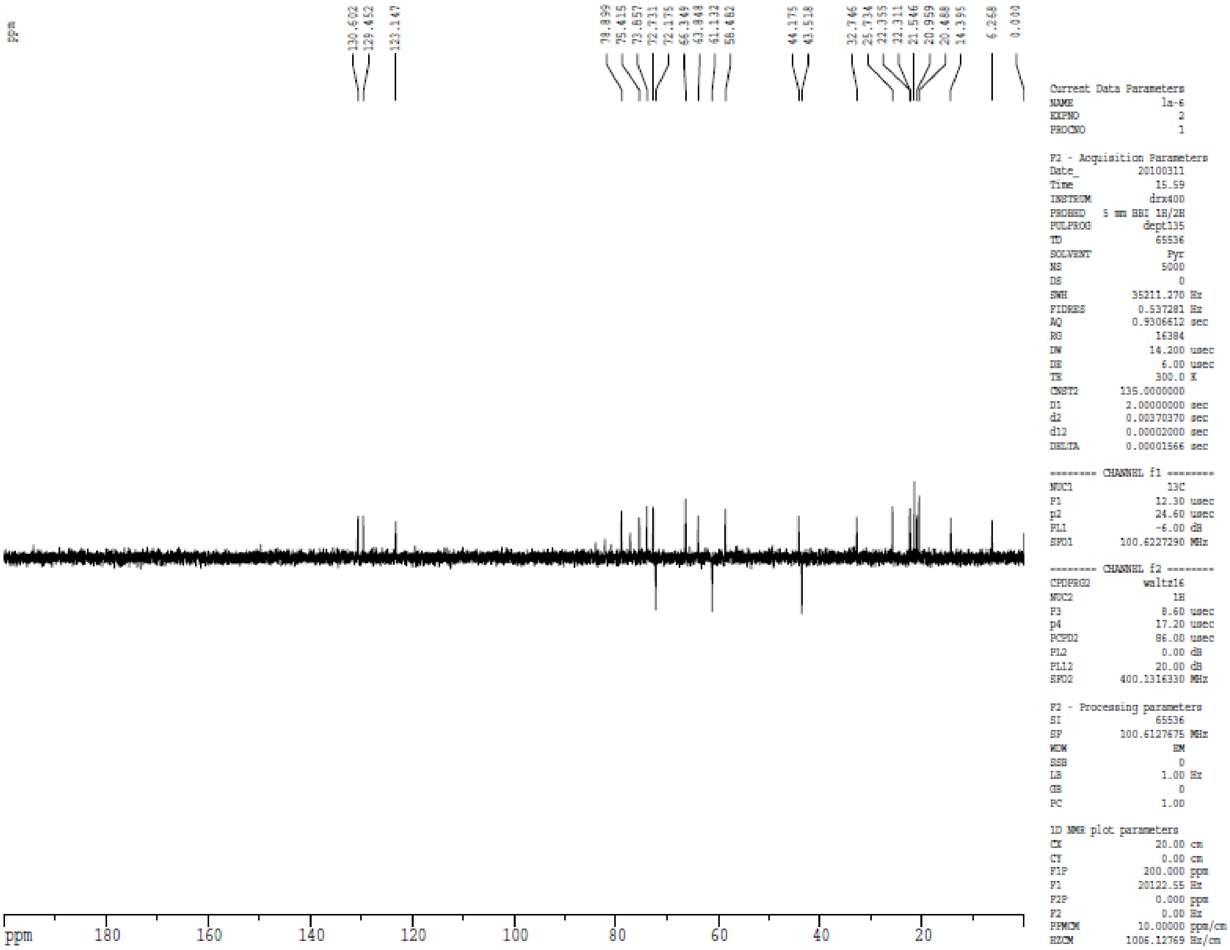

Figure S13. HSQC spectrum of the new compound **2**.

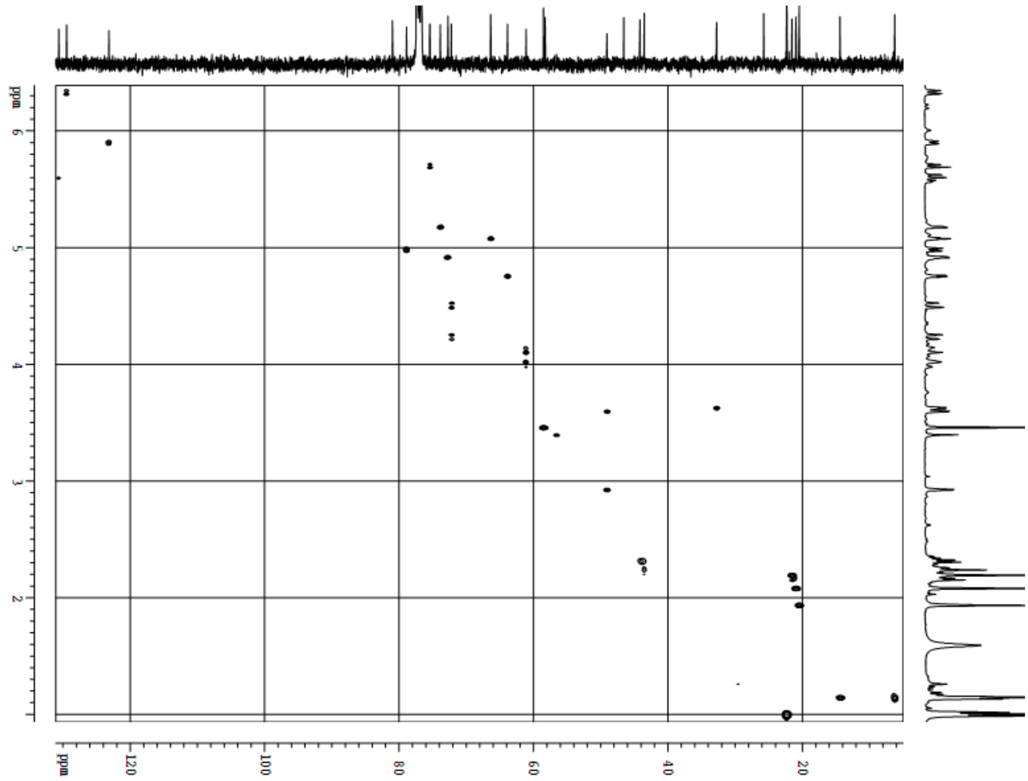

Figure S14. ^{1}H-^{1}H COSY spectrum of the new compound **2**.

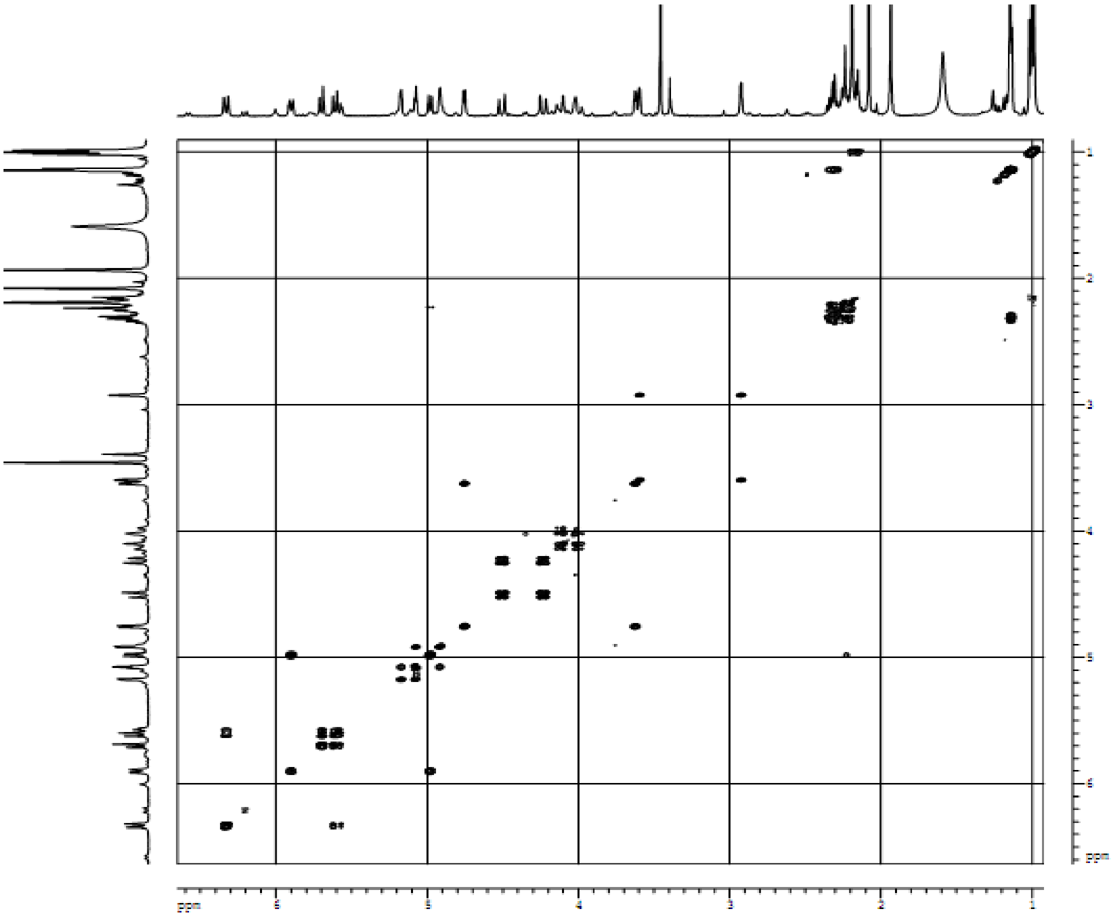

Figure S15. HMBC spectrum of the new compound **2**.

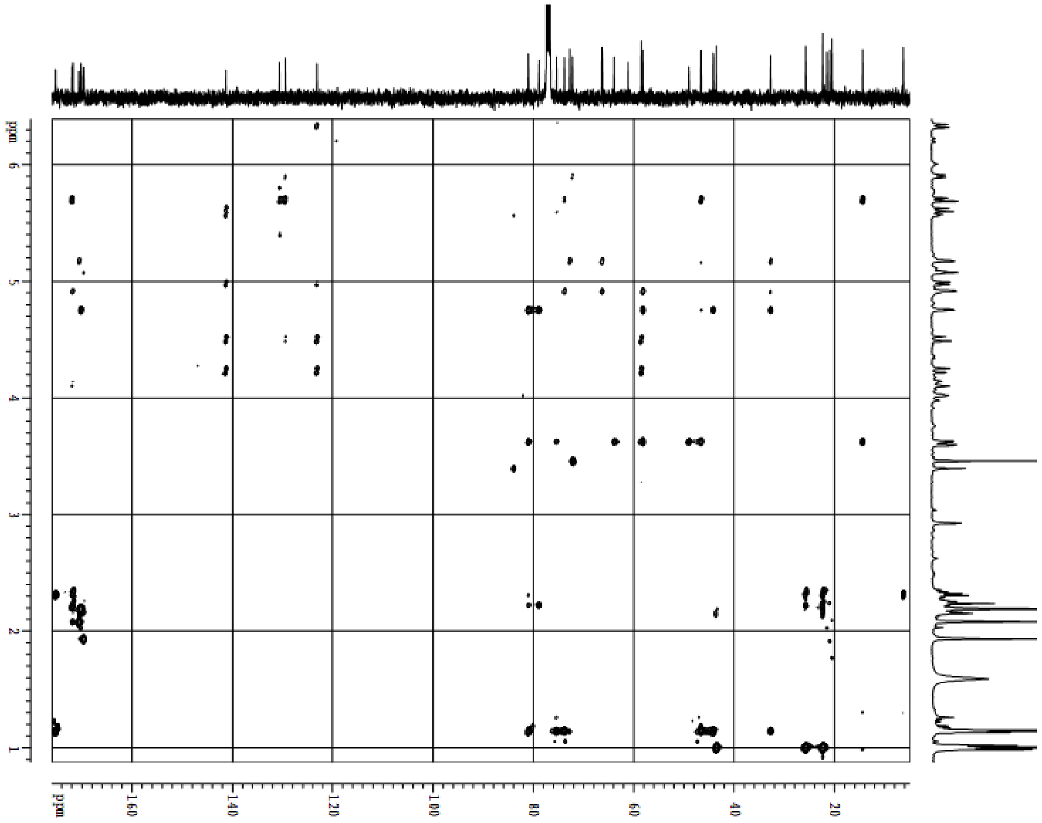

Figure S16. NOESY spectrum of the new compound **2**.

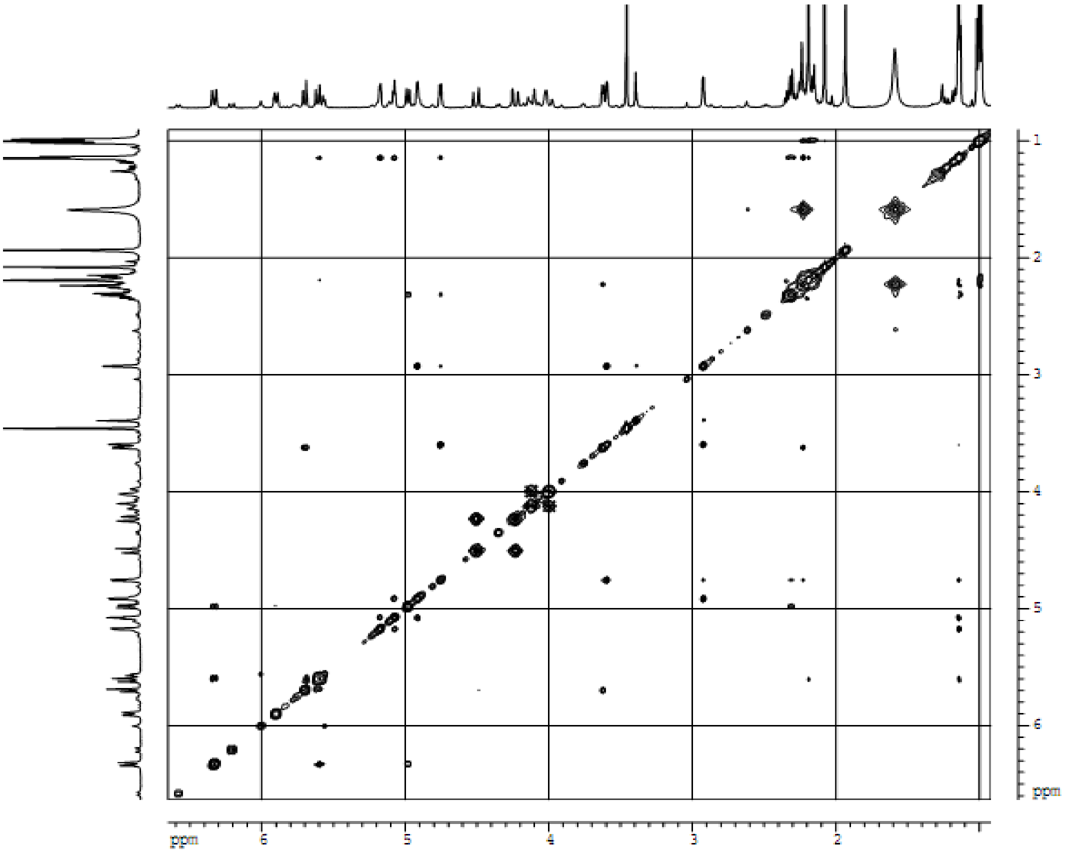

Figure S17. HR-ESIMS spectrum of new compound **3**.

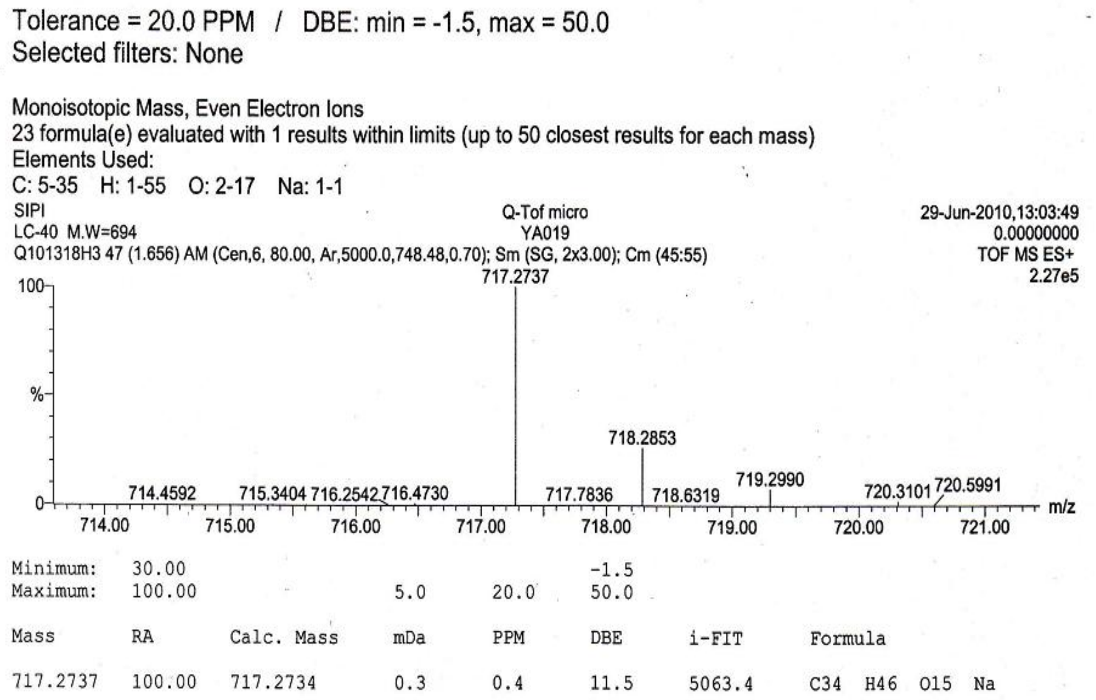

Figure S18. ^{1}H MNR (400 MHz, $CDCl_3$) spectrum of the new compound **3**.

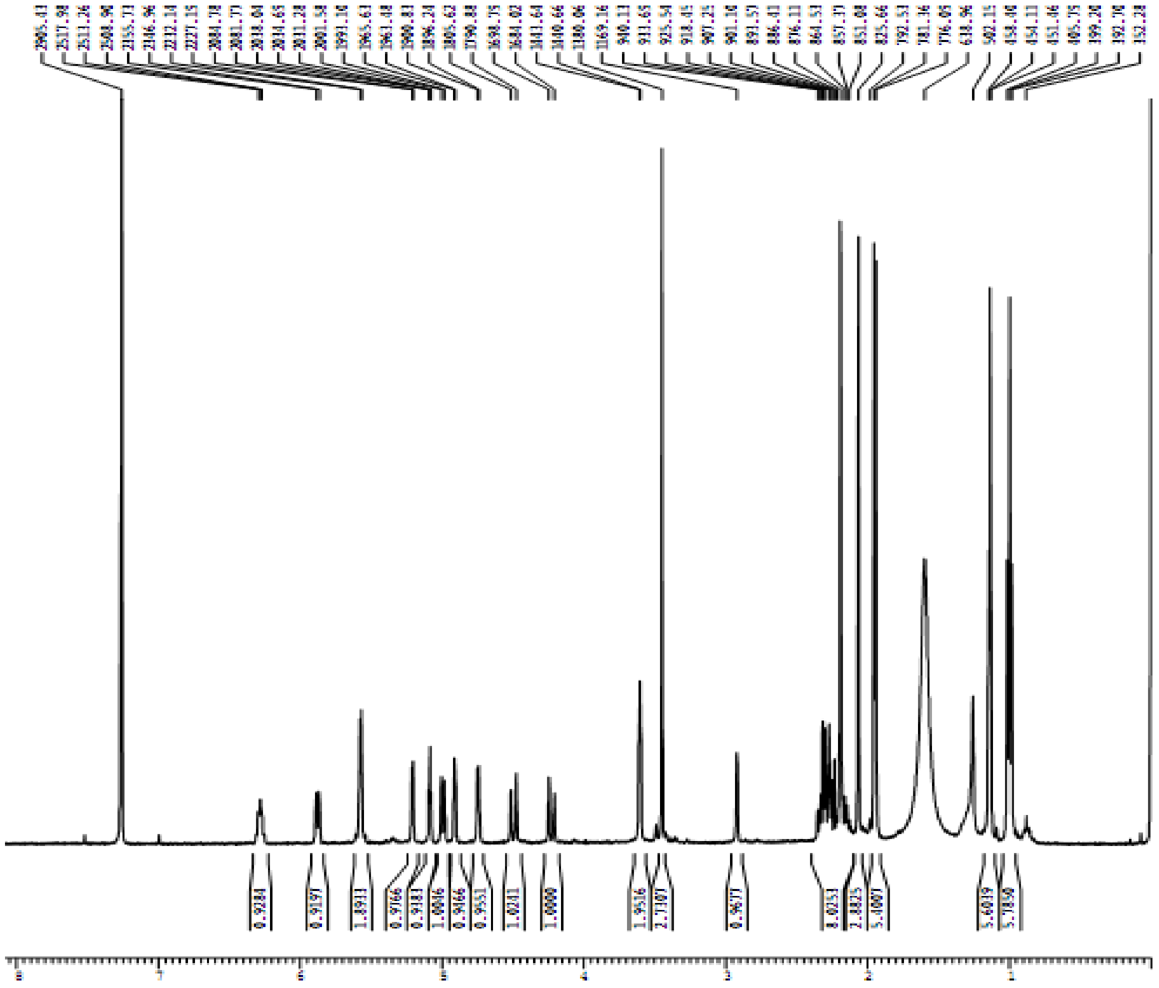

Figure S19. ^{13}C MNR (100 MHz, $CDCl_3$) spectrum of the new compound **3**.

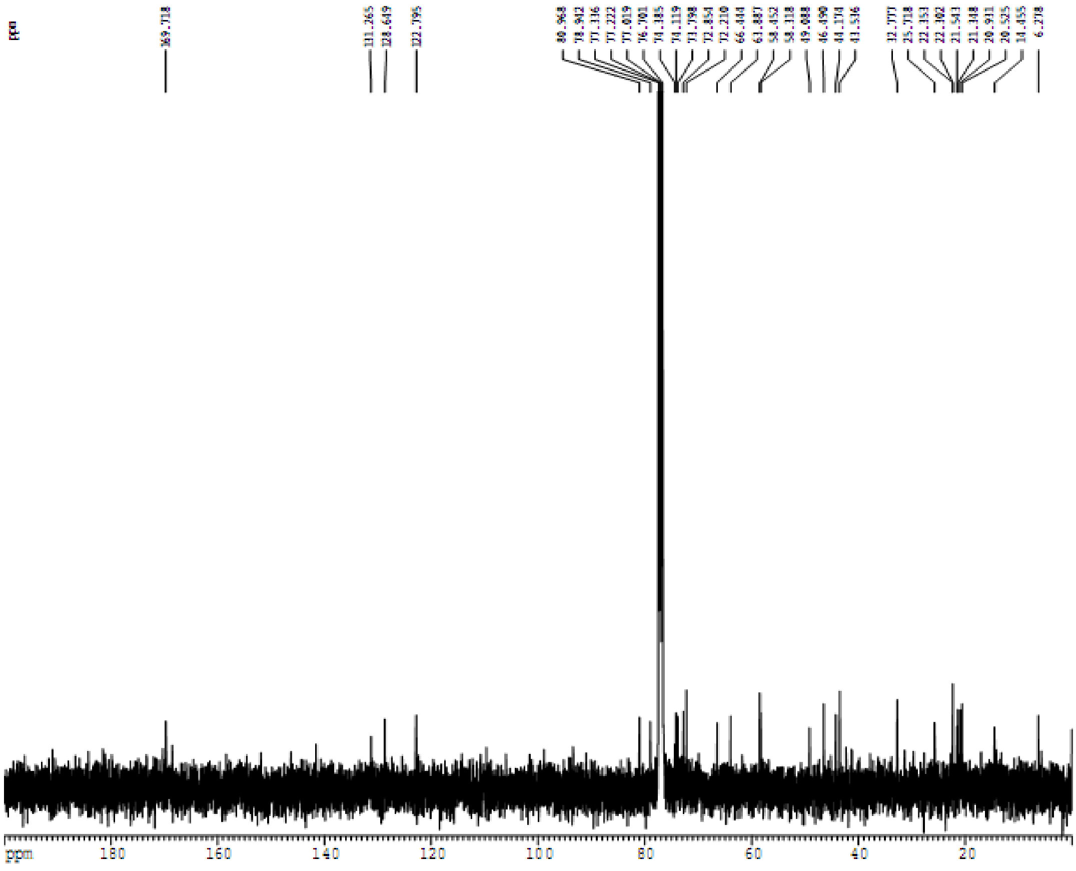

Figure S20. DEPT spectrum of the new compound **3**.

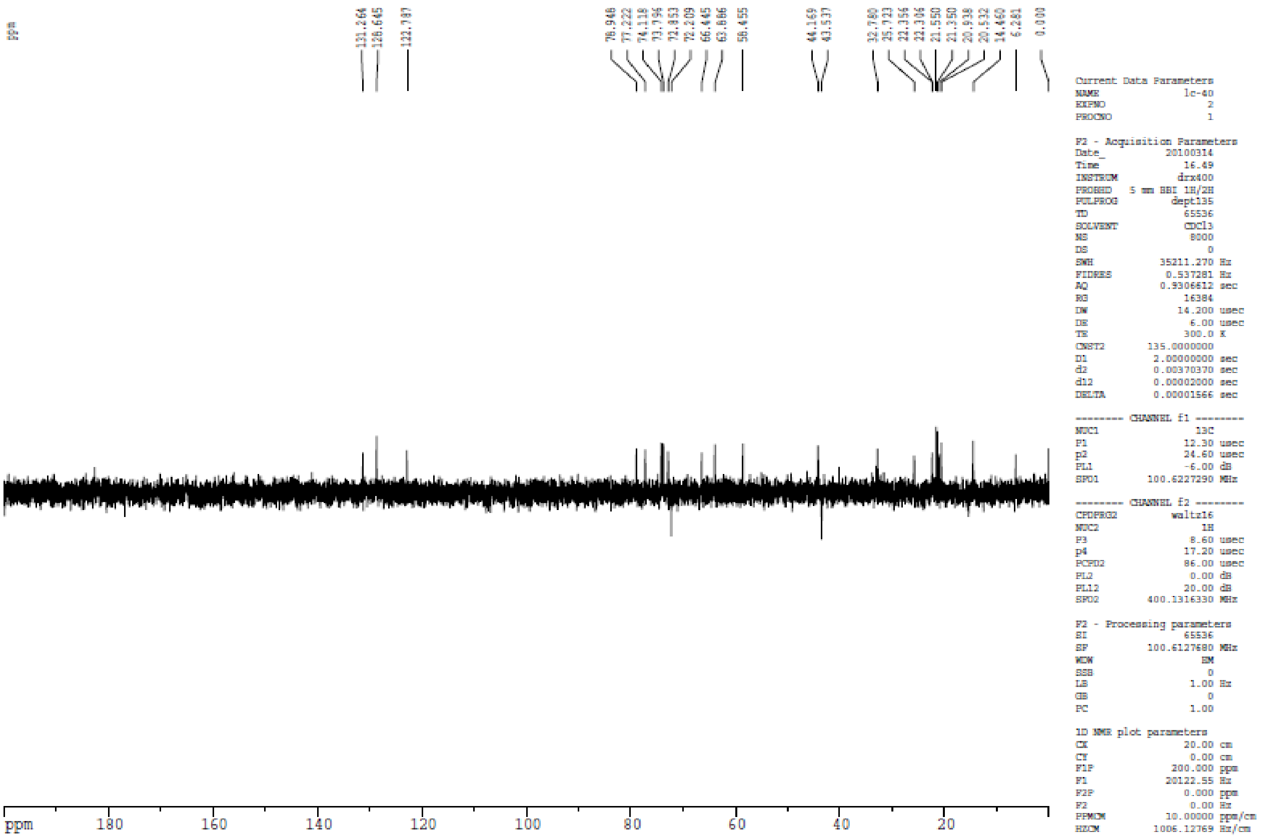

Figure S21. HSQC spectrum of the new compound **3**.

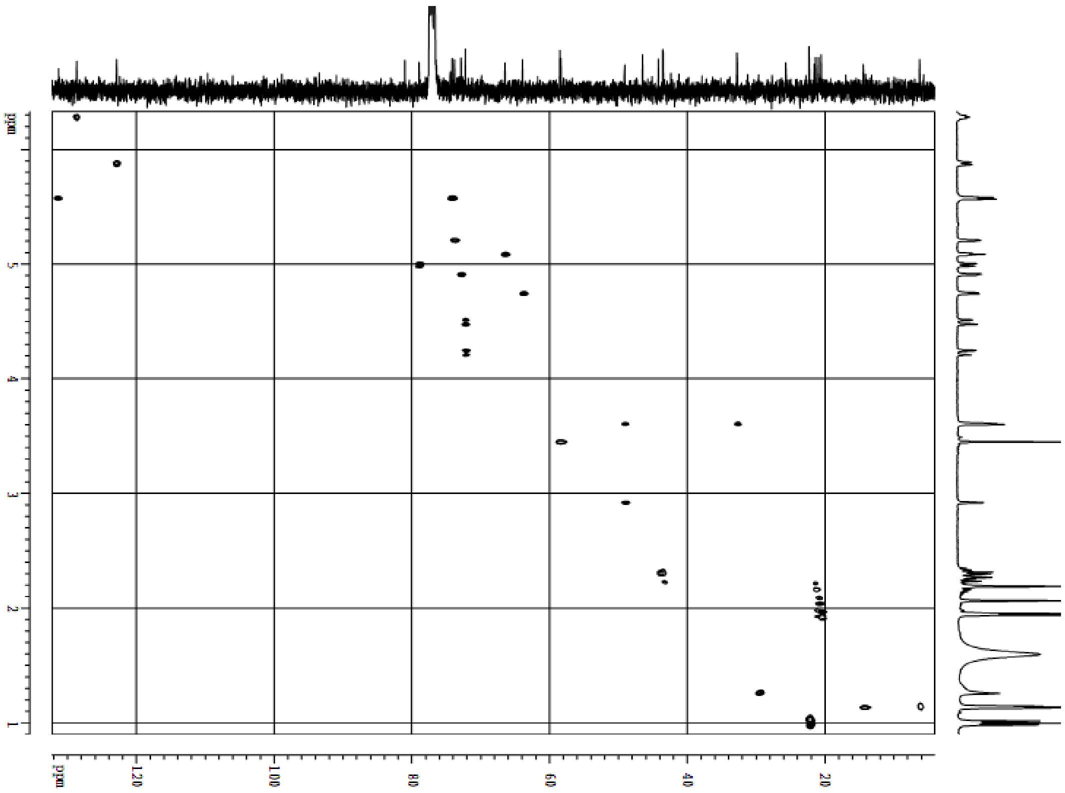

Figure S22. ^{1}H-^{1}H COSY spectrum of the new compound **3**.

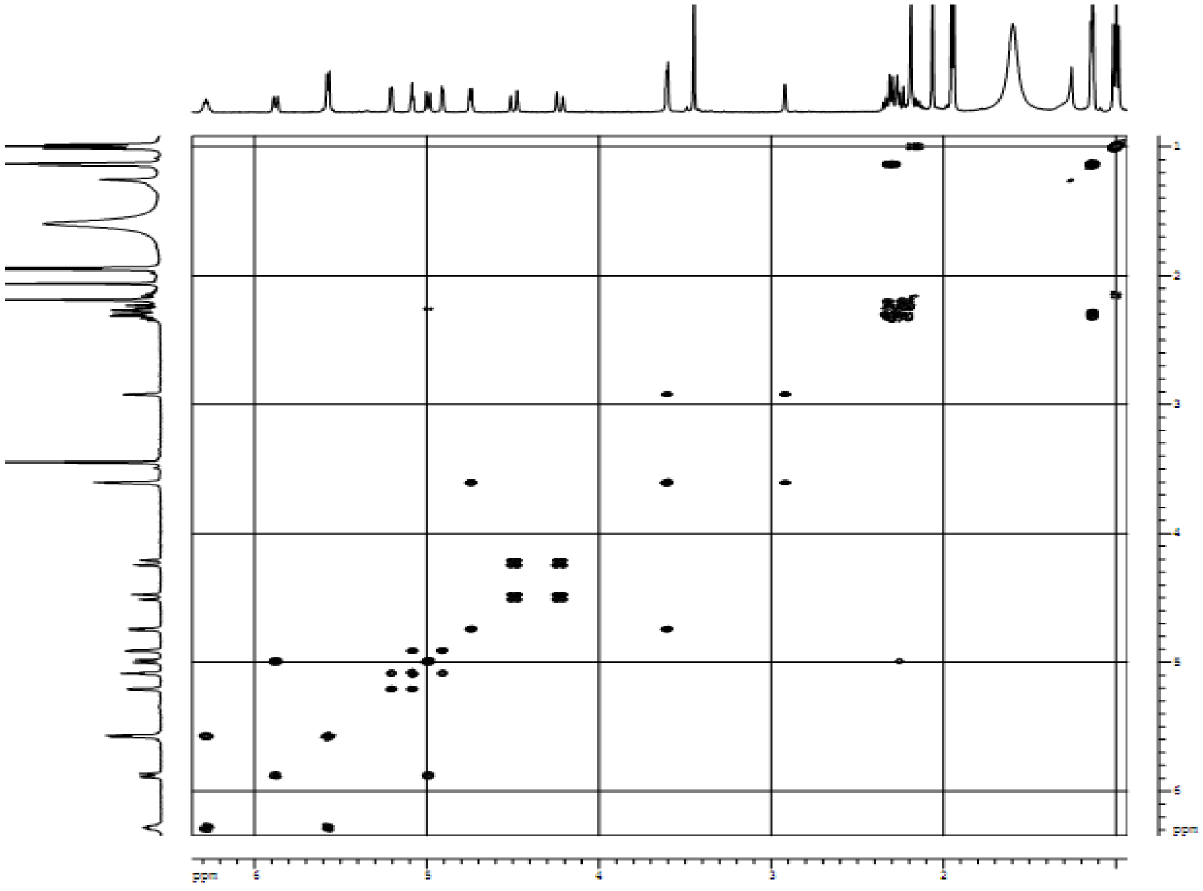

Figure S23. HMBC spectrum of the new compound **3**.

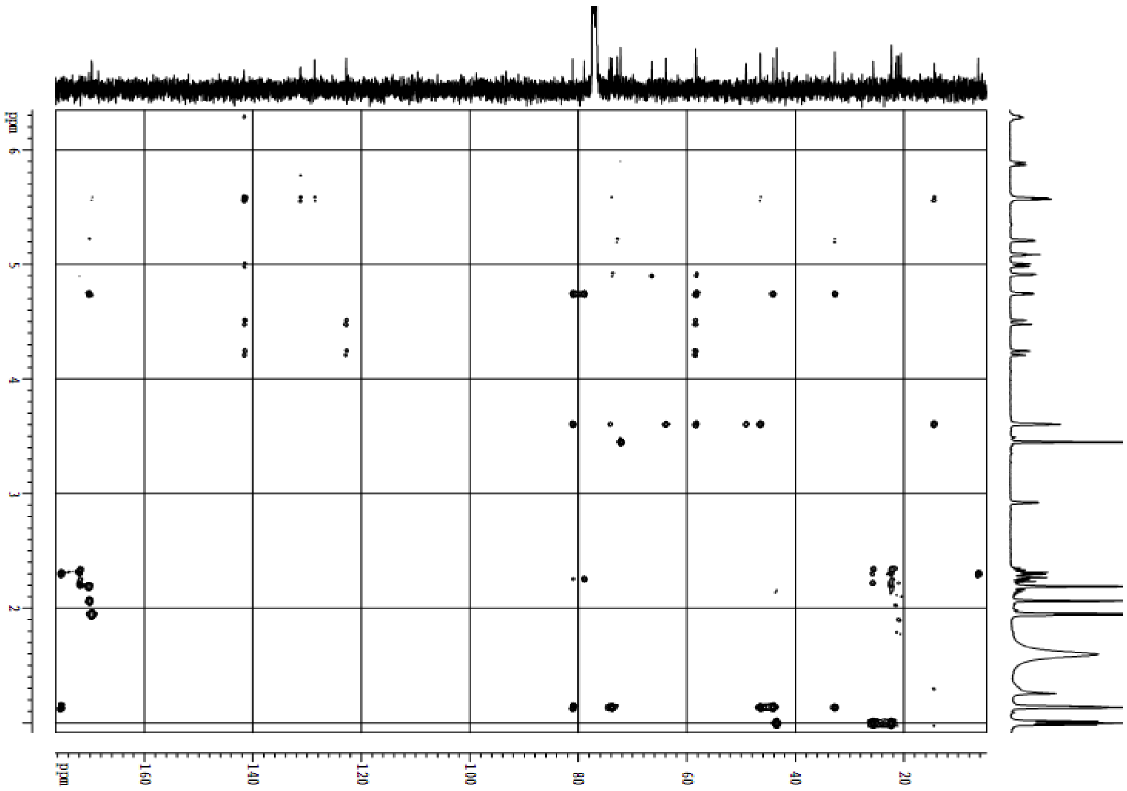

Figure S24. NOESY spectrum of the new compound **3**.

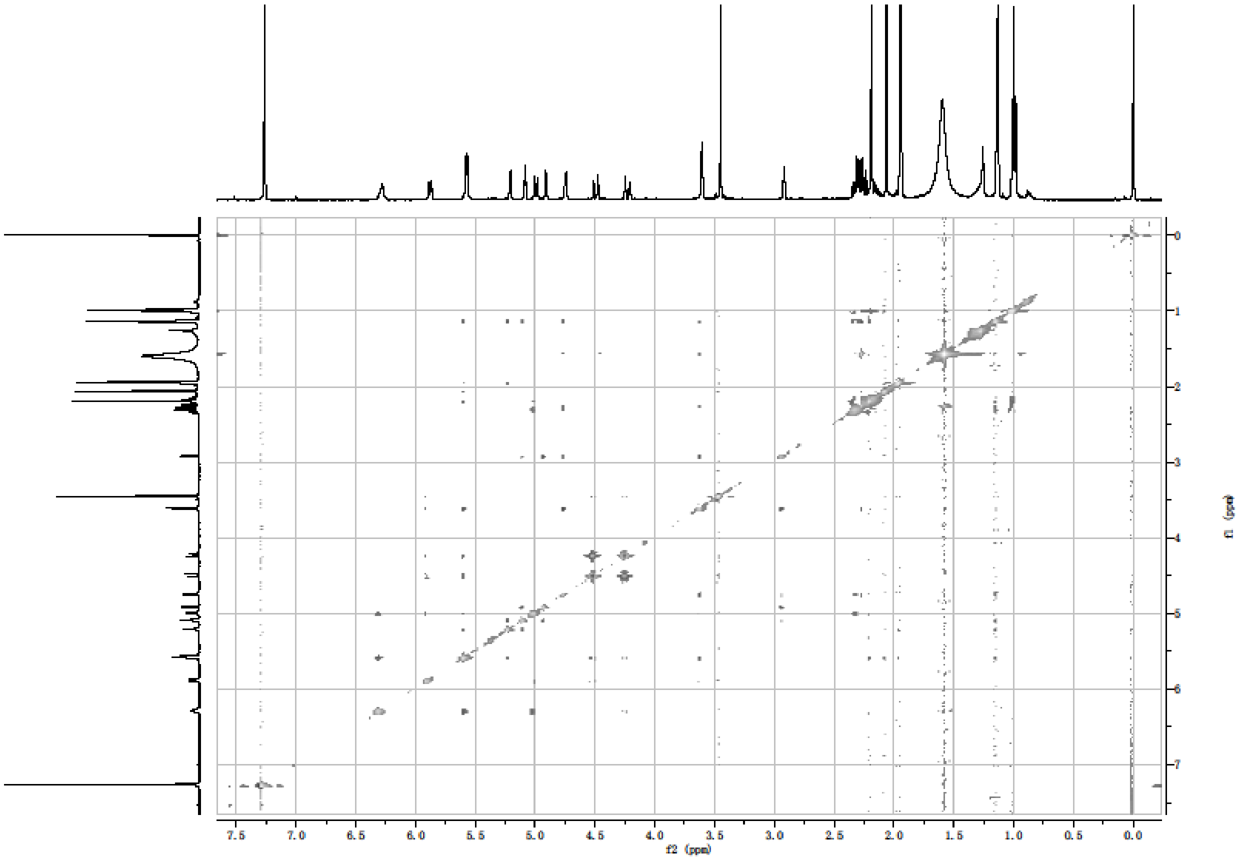

Figure S25. HRESIMS spectrum of compound **4**.

Tolerance = 10.0 PPM / DBE: min = -1.5, max = 50.0
Selected filters: None

Monoisotopic Mass, Even Electron Ions
19 formula(e) evaluated with 1 results within limits (up to 50 closest results for each mass)
Elements Used:
C: 10-40 H: 10-60 O: 5-16 Na: 1-1

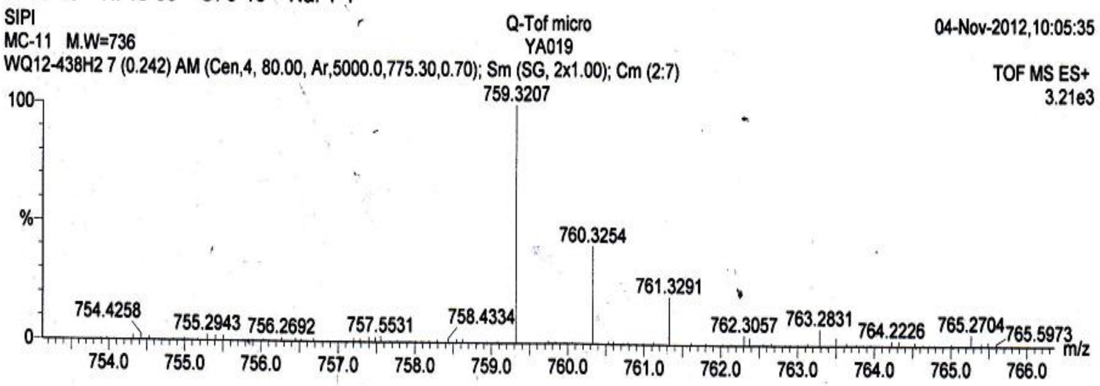

Minimum:	60.00				-1.5		
Maximum:	100.00		5.0	10.0	50.0		
Mass	RA	Calc. Mass	mDa	PPM	DBE	i-FIT	Formula
759.3207	100.00	759.3204	0.3	0.4	11.5	51.5	C37 H52 O15 Na

Figure S26. ^{1}H NMR spectrum of compound **4** in $CDCl_3$.

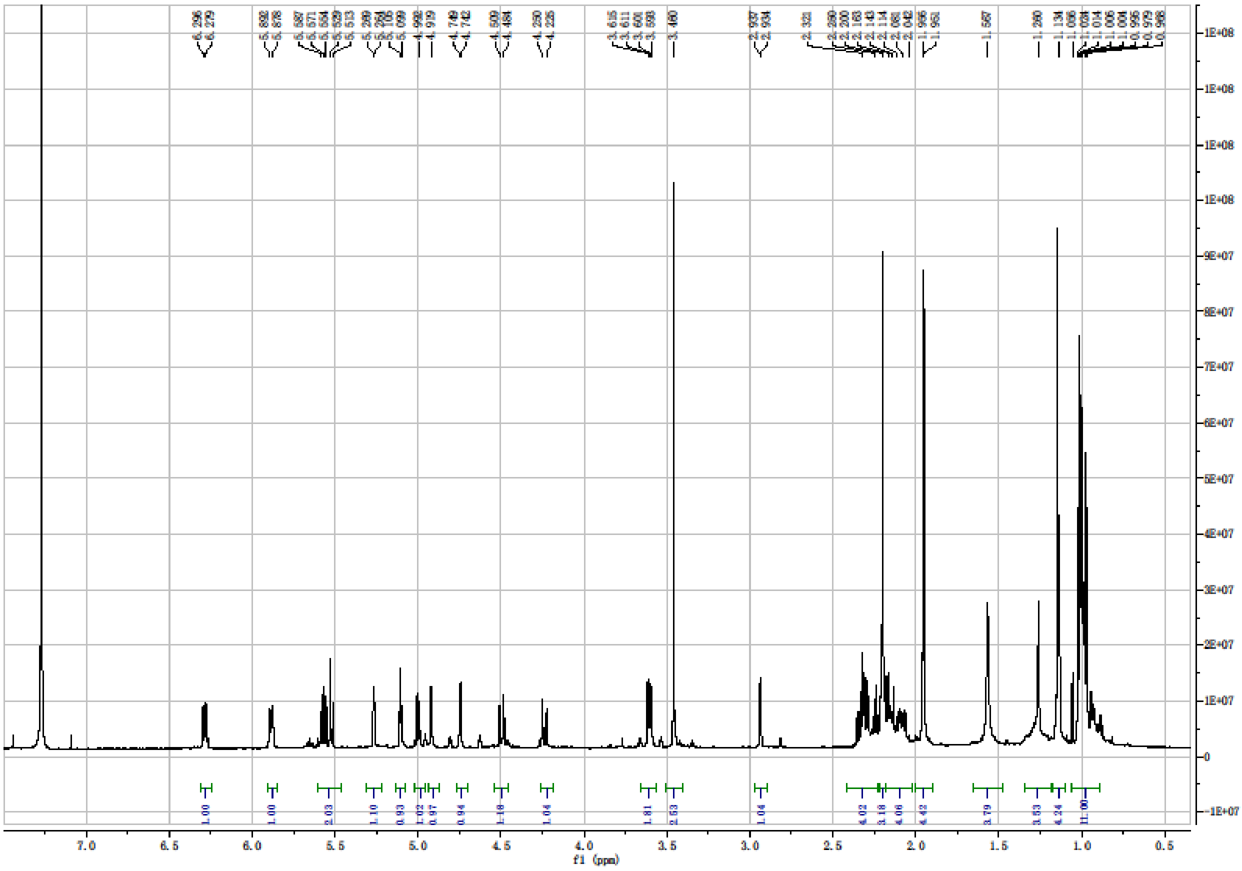

Figure S27. ^{13}C NMR spectrum of compound **4** in $CDCl_3$.

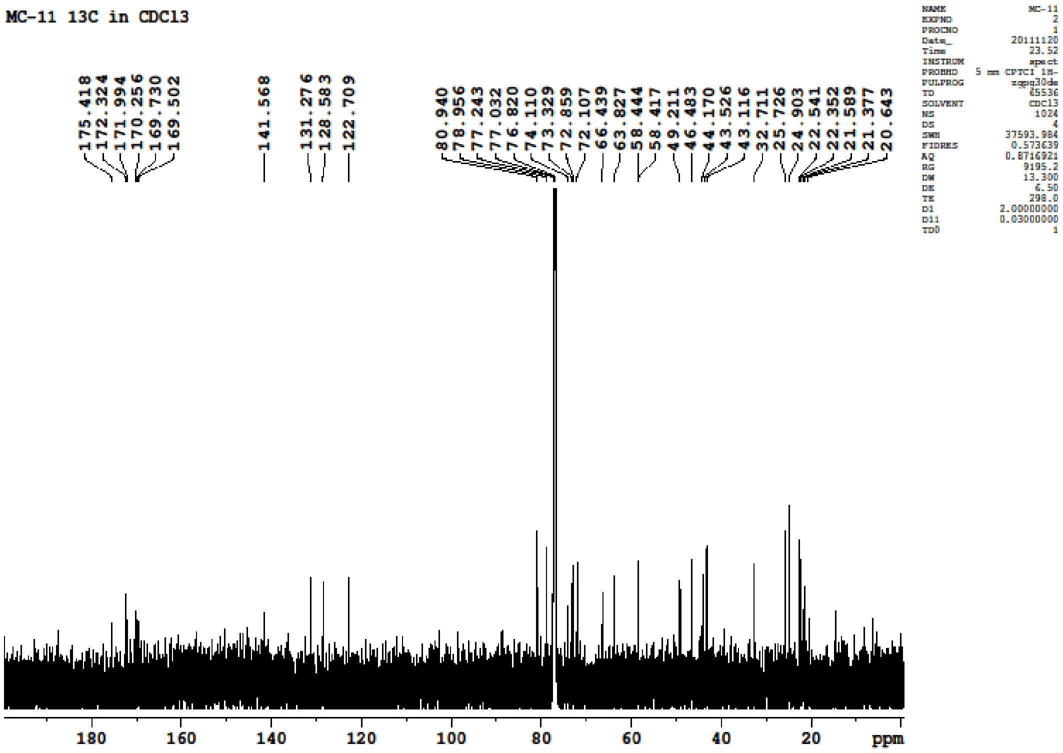

Figure S28. DEPT spectrum of compound **4** in $CDCl_3$.

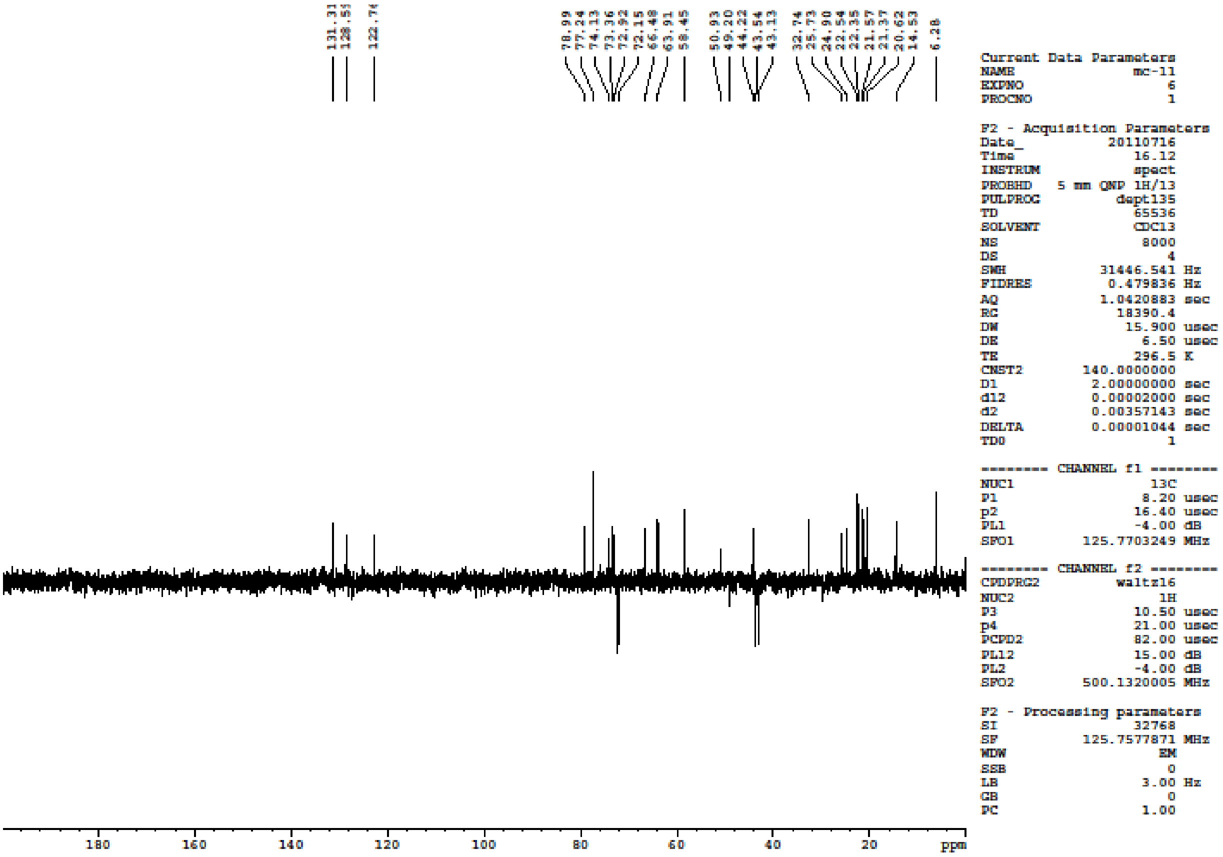

Figure S29. HSQC spectrum of compound **4** in $CDCl_3$.

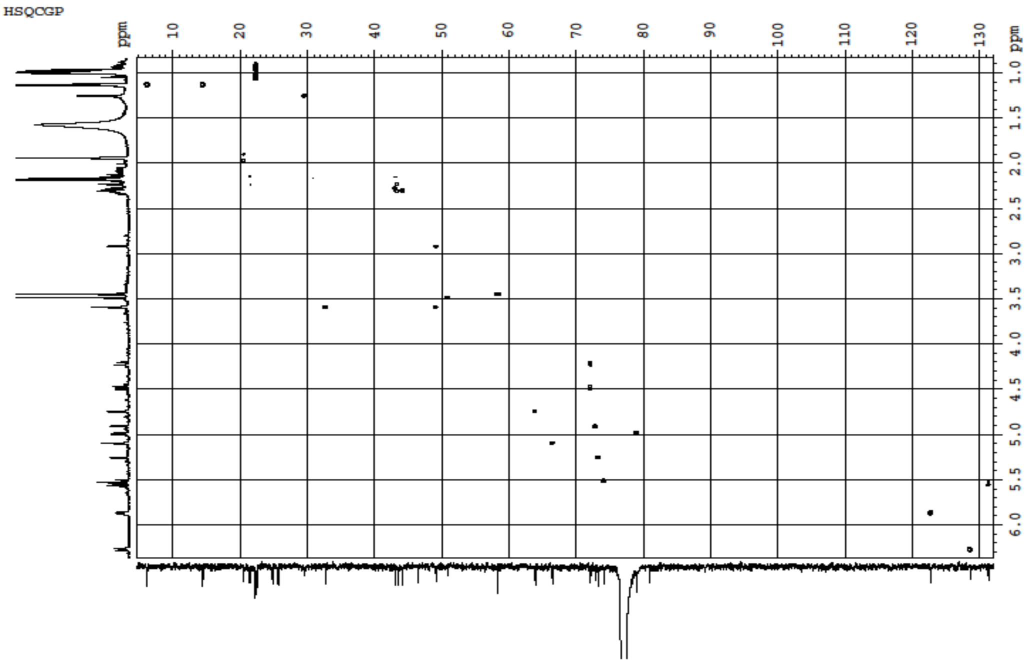

Figure S30. ^{1}H-^{1}H COSY spectrum of compound **4** in $CDCl_3$.

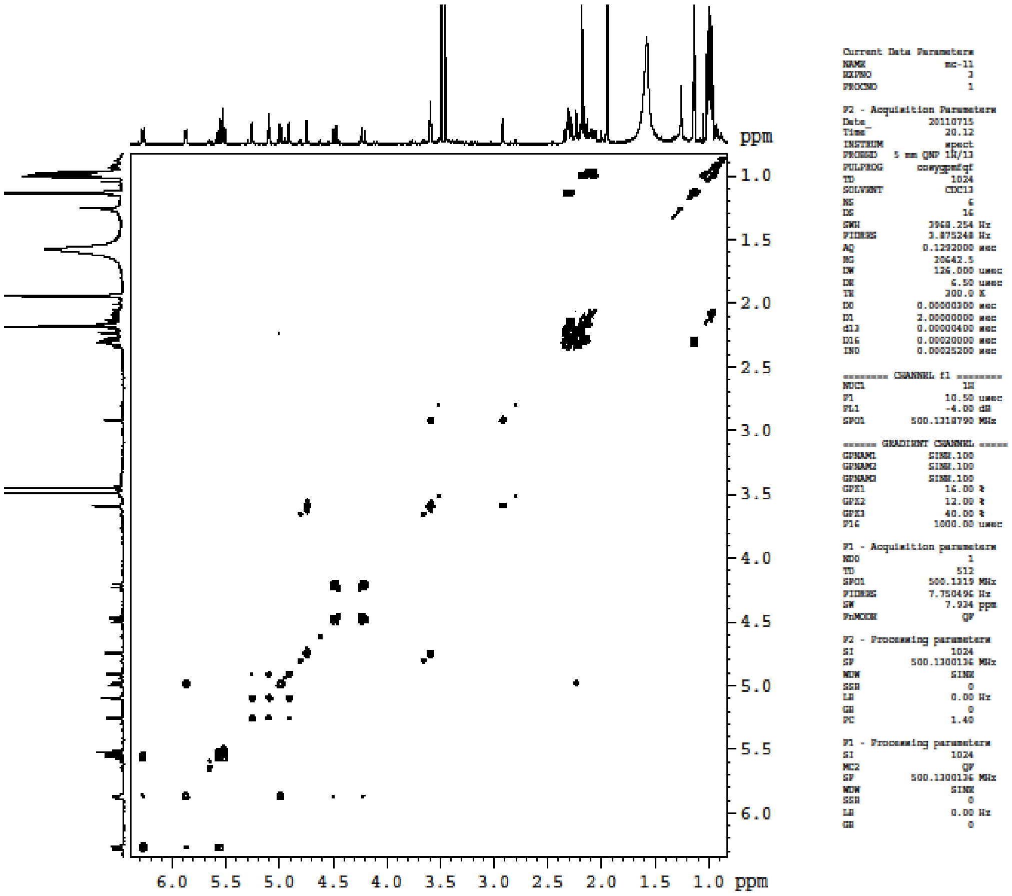

Figure S31. HMBC spectrum of compound **4** in $CDCl_3$.

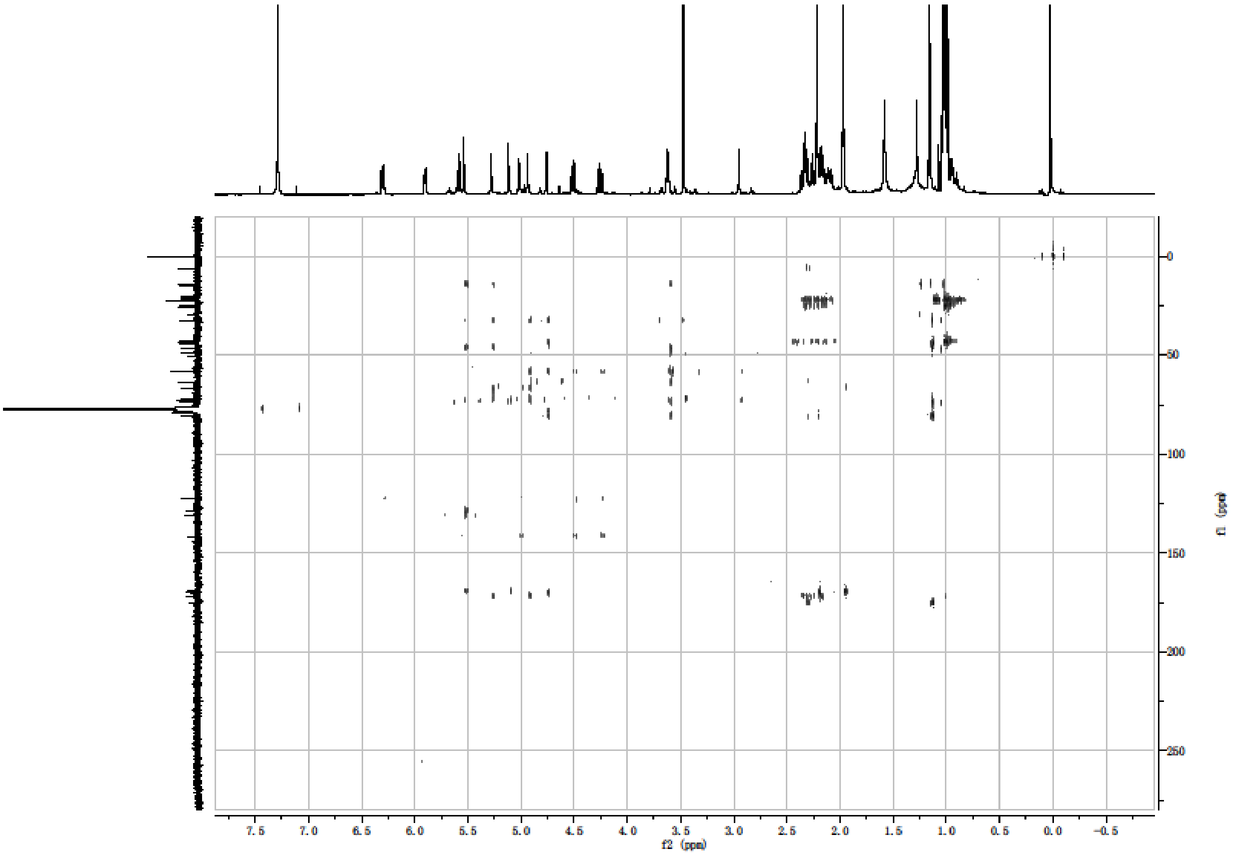

Figure S32. NOESY spectrum of compound **4** in $CDCl_3$.

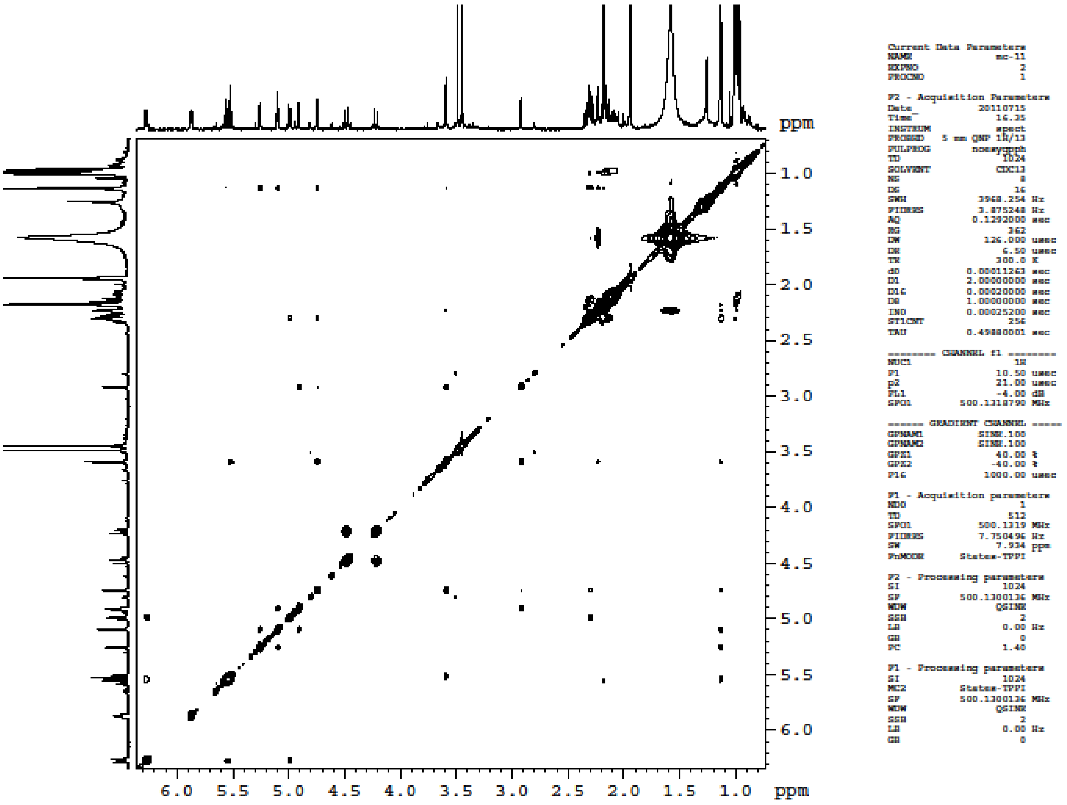

Figure S33. HR-ESIMS spectrum of the new compound **5**.

Tolerance = 10.0 PPM / DBE: min = -1.5, max = 50.0
Selected filters: None

Monoisotopic Mass, Even Electron Ions
30 formula(e) evaluated with 1 results within limits (up to 50 closest results for each mass)
Elements Used:
C: 5-38 H: 1-50 O: 5-16 Na: 1-1

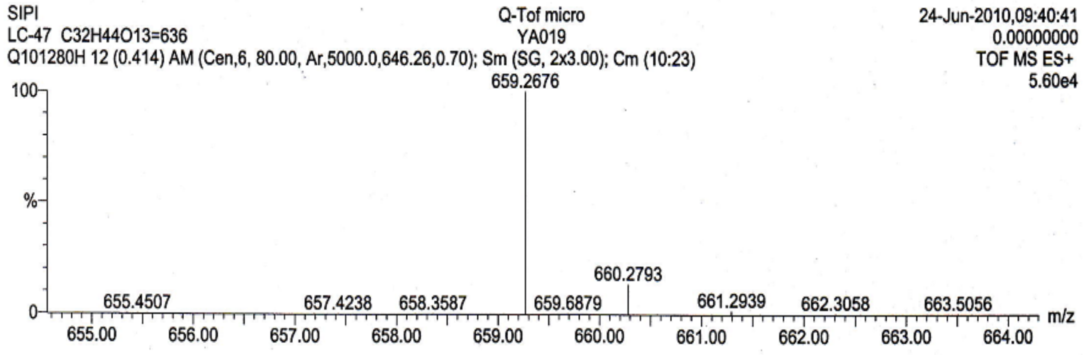

Minimum:	35.00				-1.5		
Maximum:	100.00		5.0	10.0	50.0		
Mass	RA	Calc. Mass	mDa	PPM	DBE	i-FIT	Formula
659.2676	100.00	659.2680	-0.4	-0.6	10.5	8933.2	C32 H44 O13 Na

Figure S34. ^{1}H MNR (400 MHz, $CDCl_3$) spectrum of the new compound **5**.

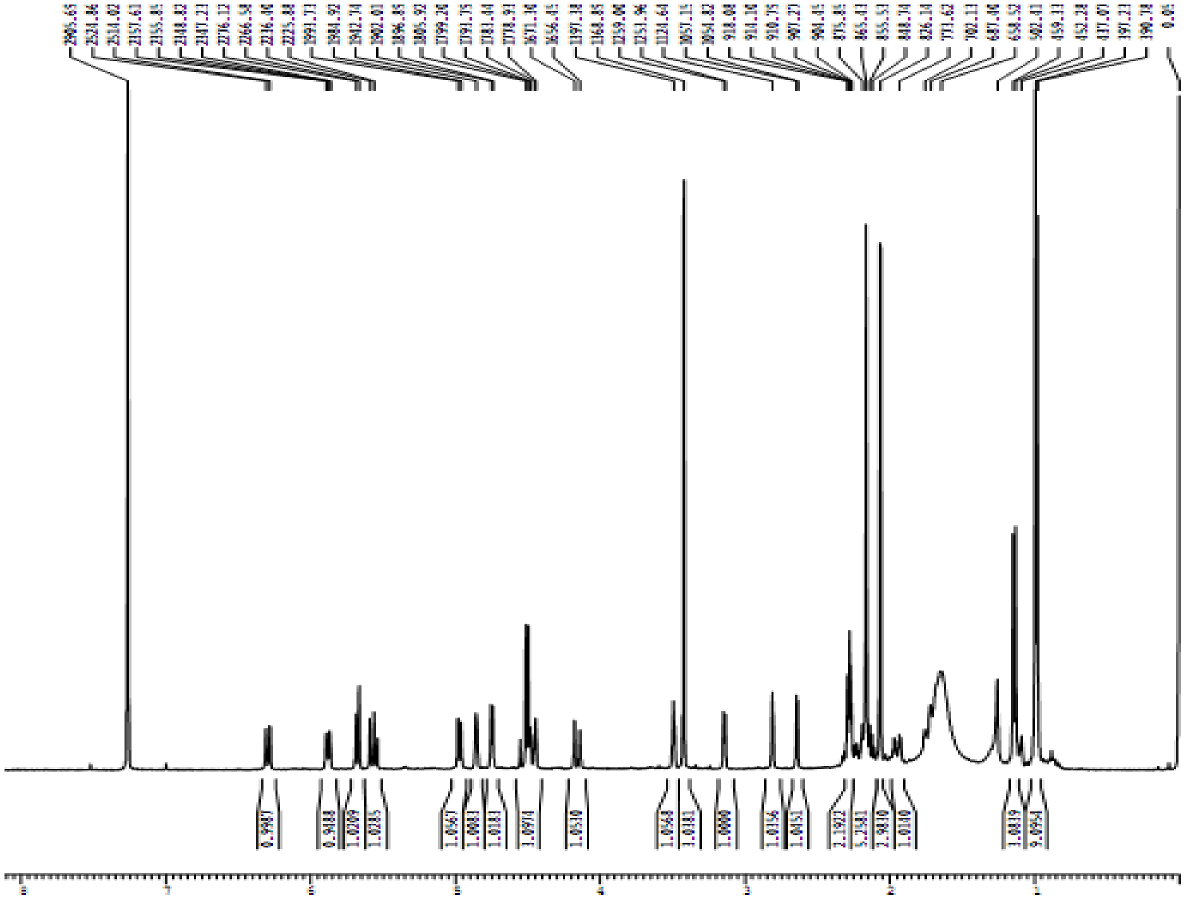

Figure S35. ^{13}C MNR (100 MHz, $CDCl_3$) spectrum of the new compound **5**.

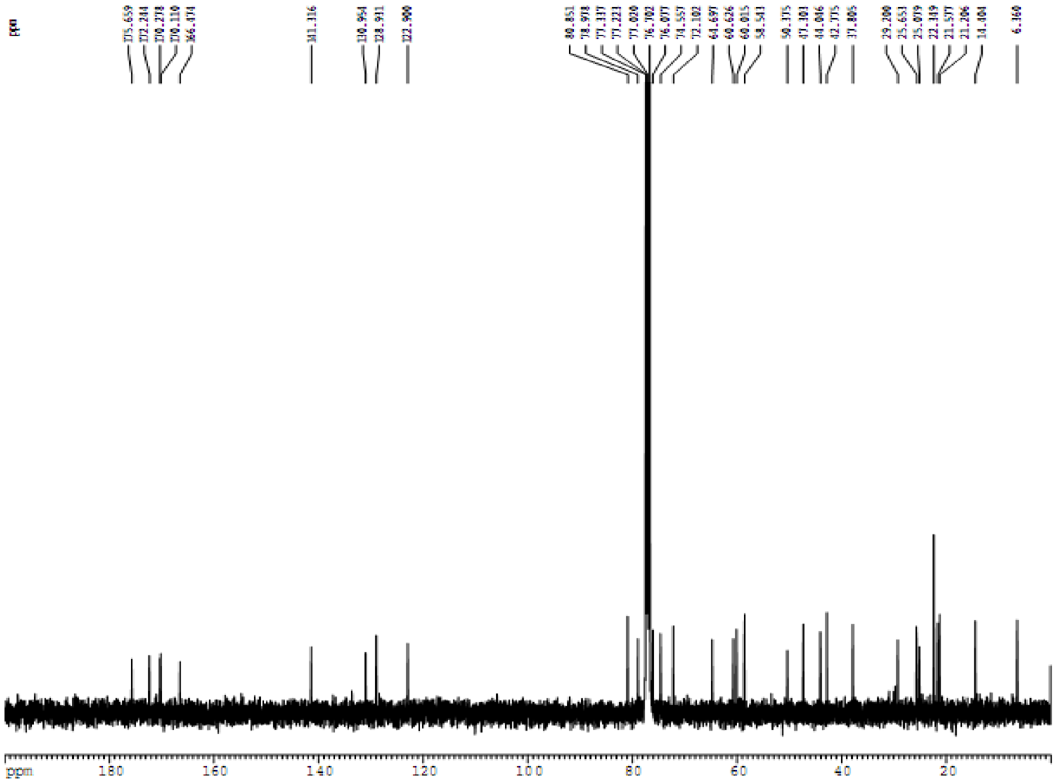

Figure S36. DEPT spectrum of the new compound **5**.

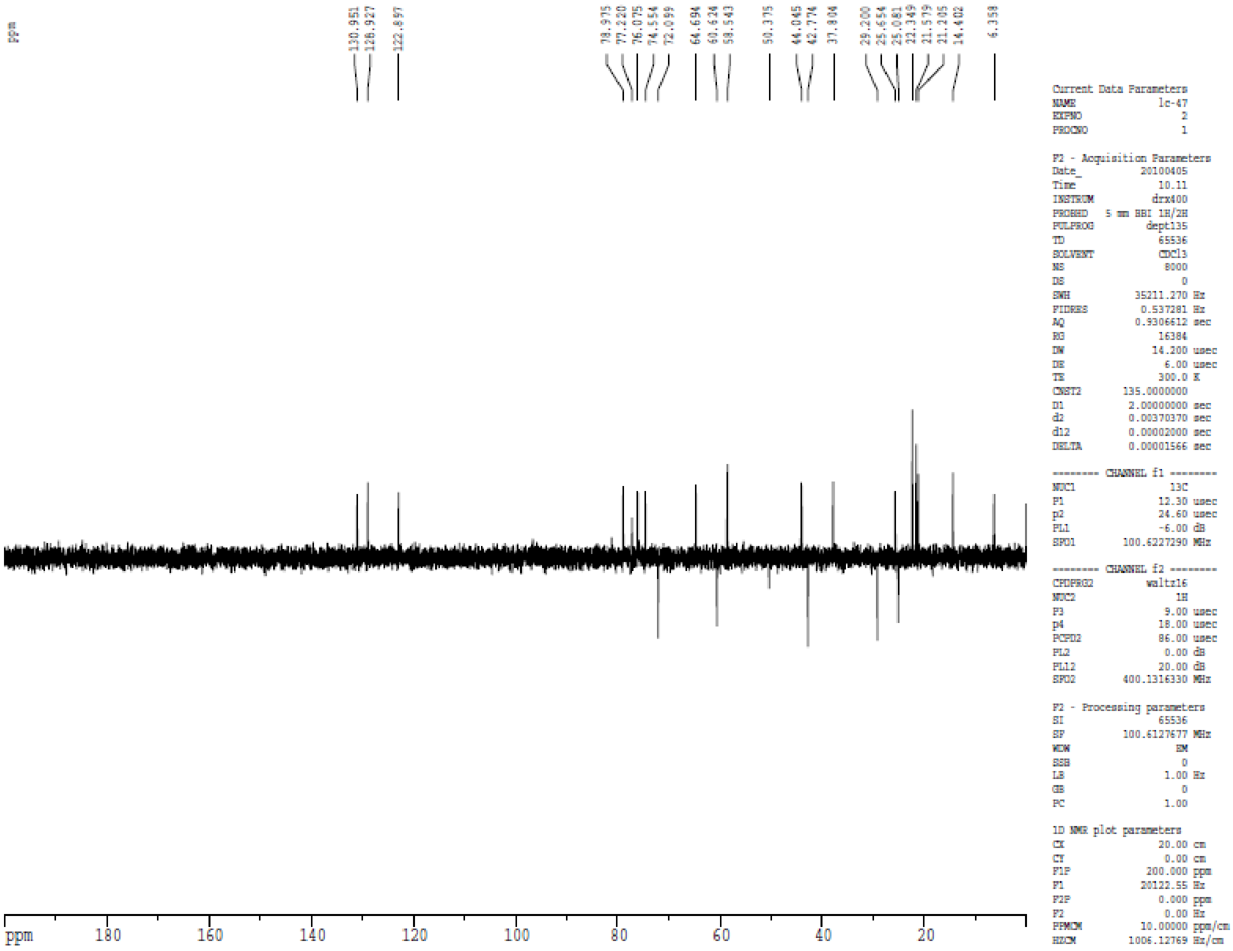

Figure S37. HSQC spectrum of the new compound **5**.

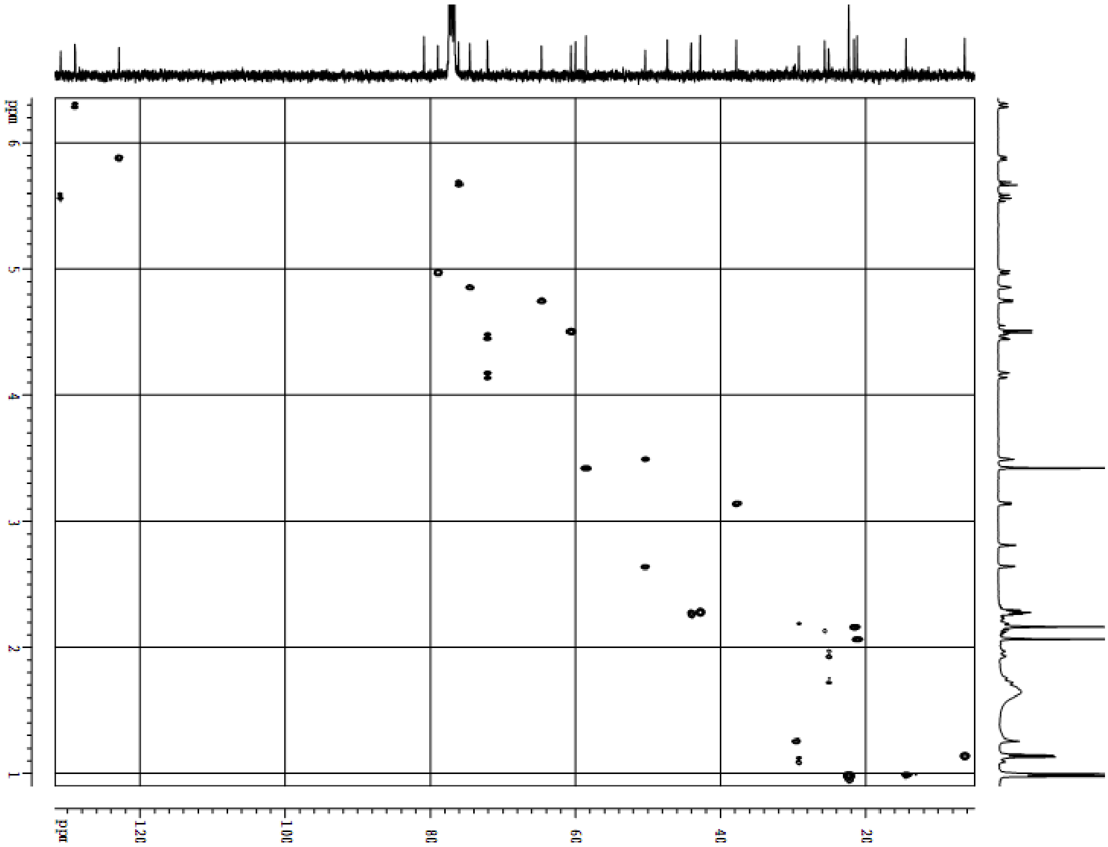

Figure S38. ^{1}H-^{1}H COSY spectrum of the new compound **5**.

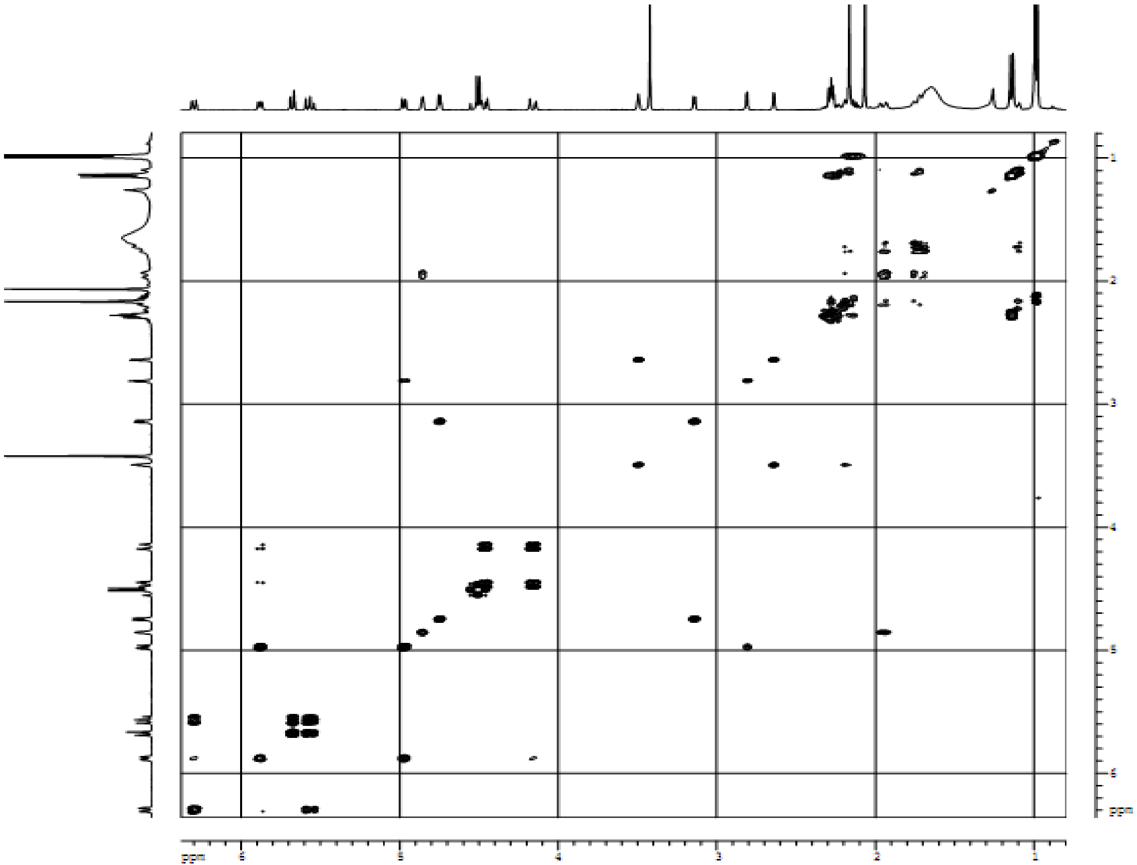

Figure S39. HMBC spectrum of the new compound **5**.

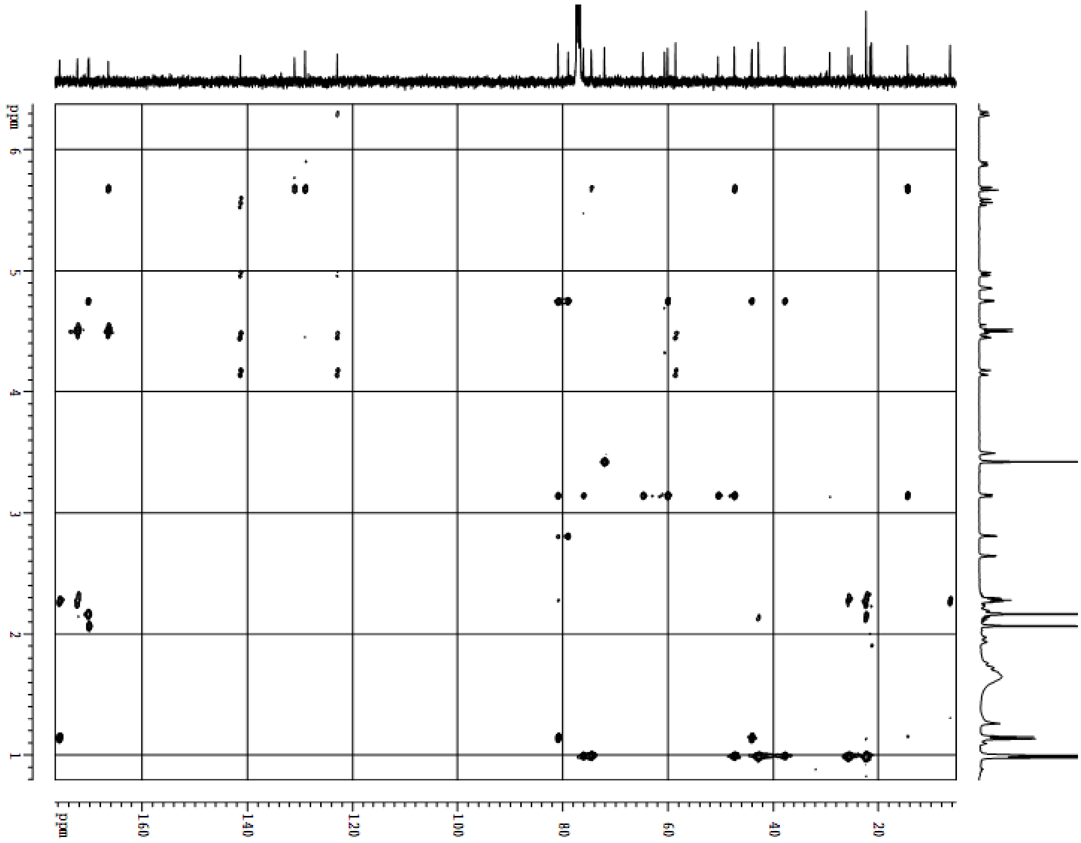

Figure S40. NOESY spectrum of the new compound **5**.

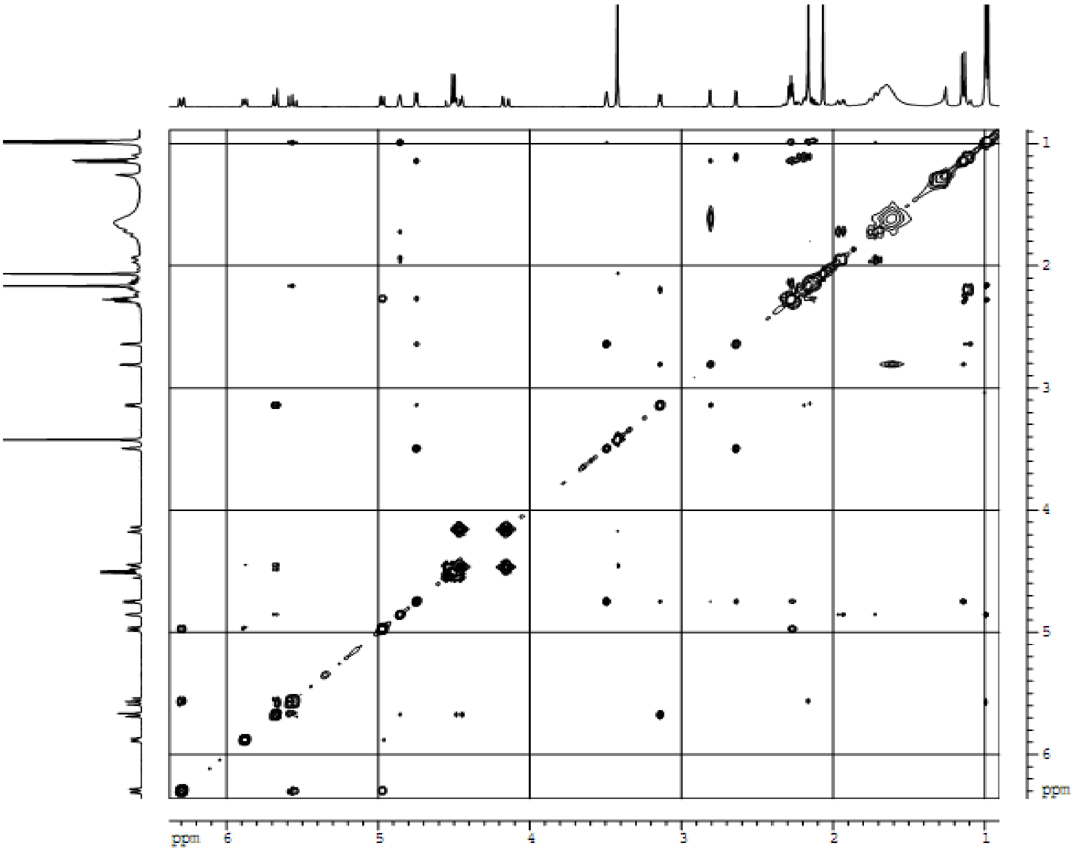

Figure S41. HR-ESIMS spectrum of the new compound **6**.

Tolerance = 5.0 PPM / DBE: min = -1.5, max = 50.0
Selected filters: None

Monoisotopic Mass, Even Electron Ions
39 formula(e) evaluated with 1 results within limits (up to 50 closest results for each mass)
Elements Used:
C: 5-40 H: 10-70 O: 1-20 Na: 1-1

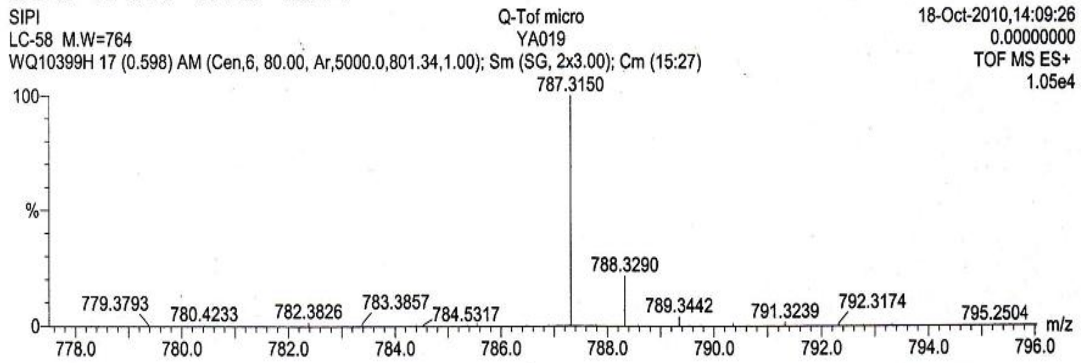

Minimum:	35.00				-1.5		
Maximum:	100.00		5.0	5.0	50.0		
Mass	RA	Calc. Mass	mDa	PPM	DBE	i-FIT	Formula
787.3150	100.00	787.3153	-0.3	-0.4	12.5	986.6	C38 H52 O16 Na

Figure S42. ^{1}H NMR spectrum of the new compound **6**.

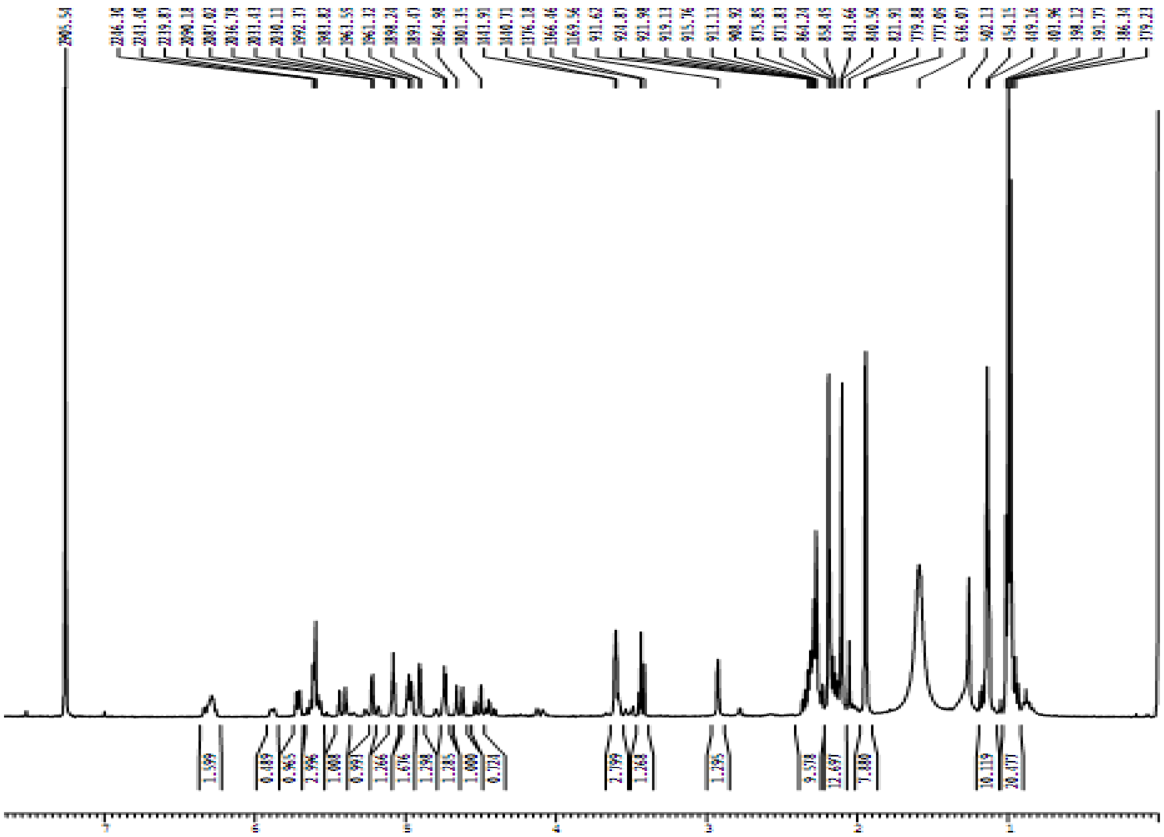

Figure S43. ^{13}C NMR spectrum of the new compound **6**.

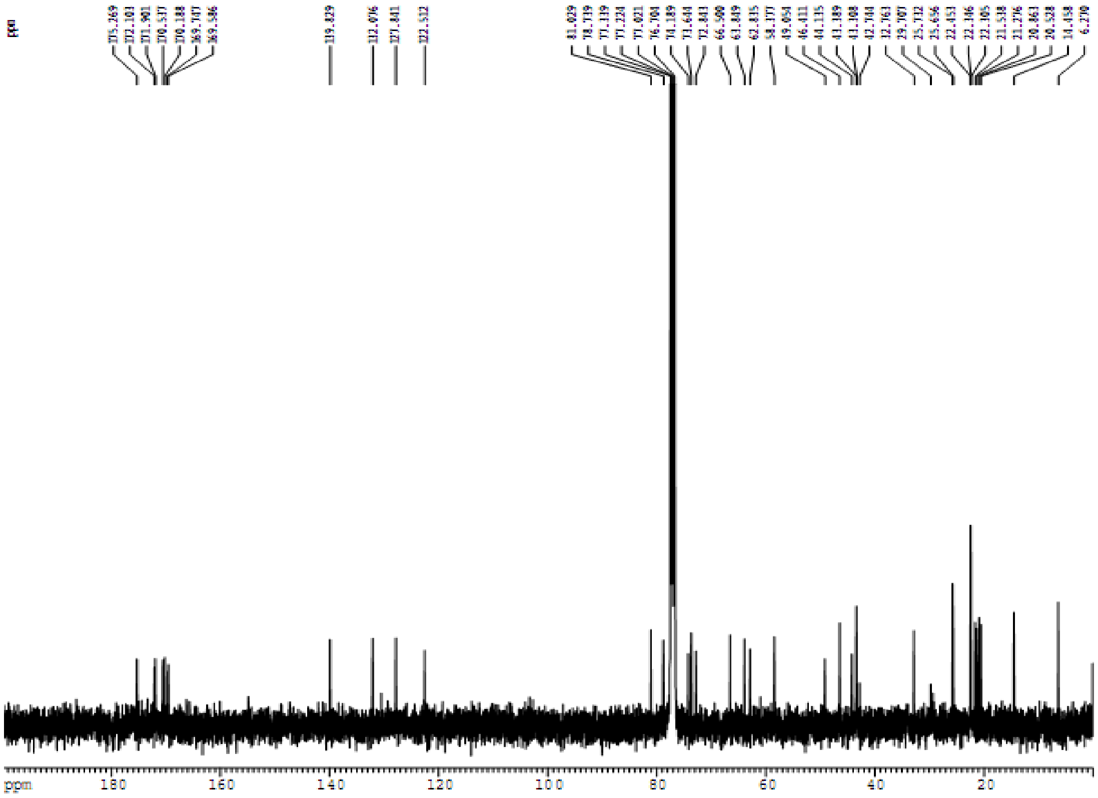

Figure S44. DEPT spectrum of the new compound **6**.

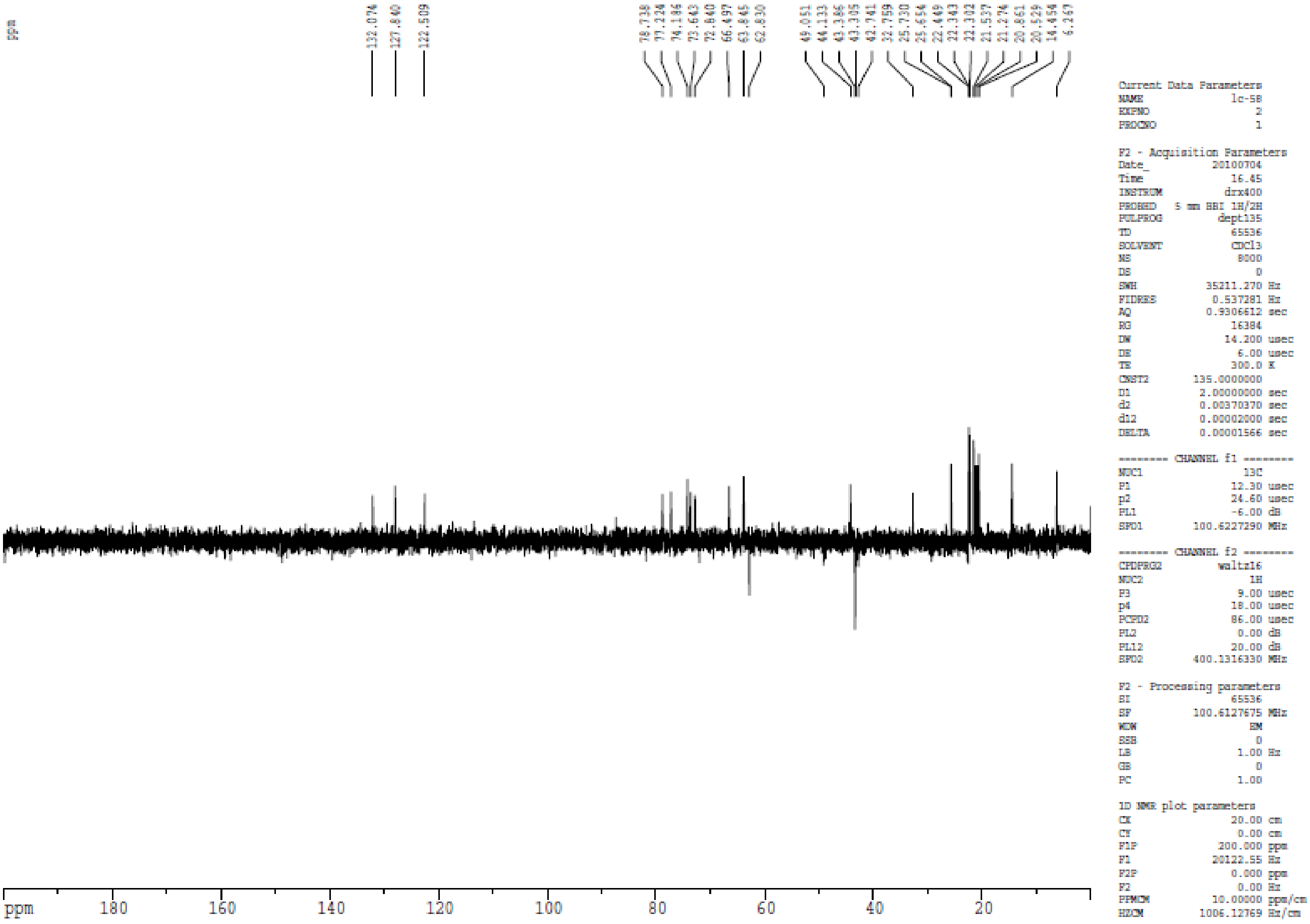

Figure S45. HSQC spectrum of the new compound **6**.

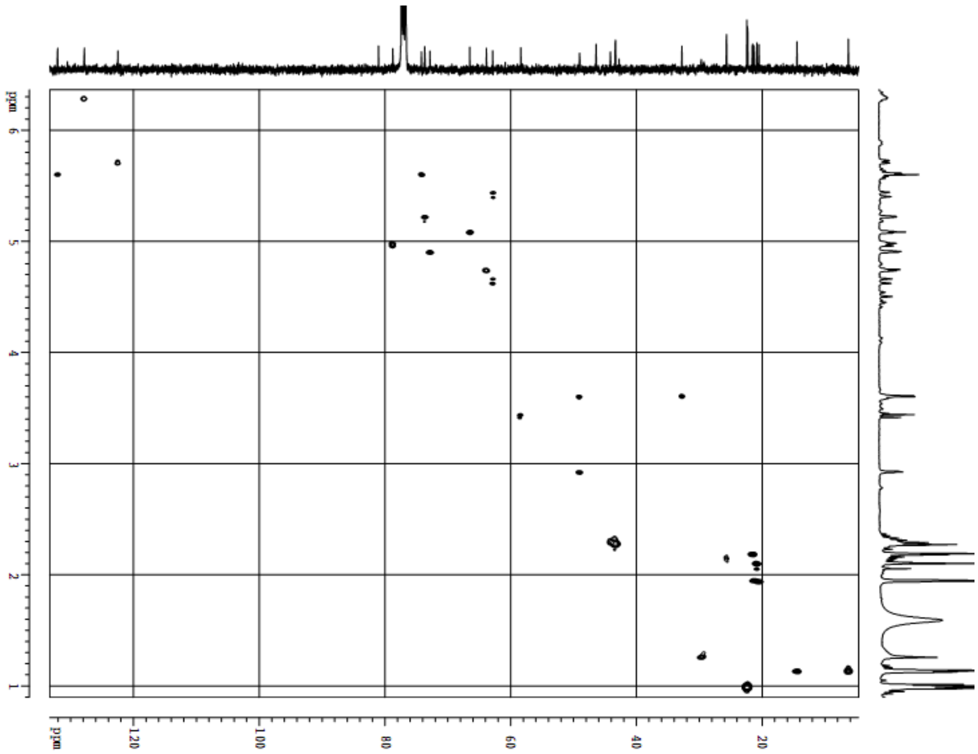

Figure S46. ^{1}H-^{1}H COSY spectrum of the new compound **6**.

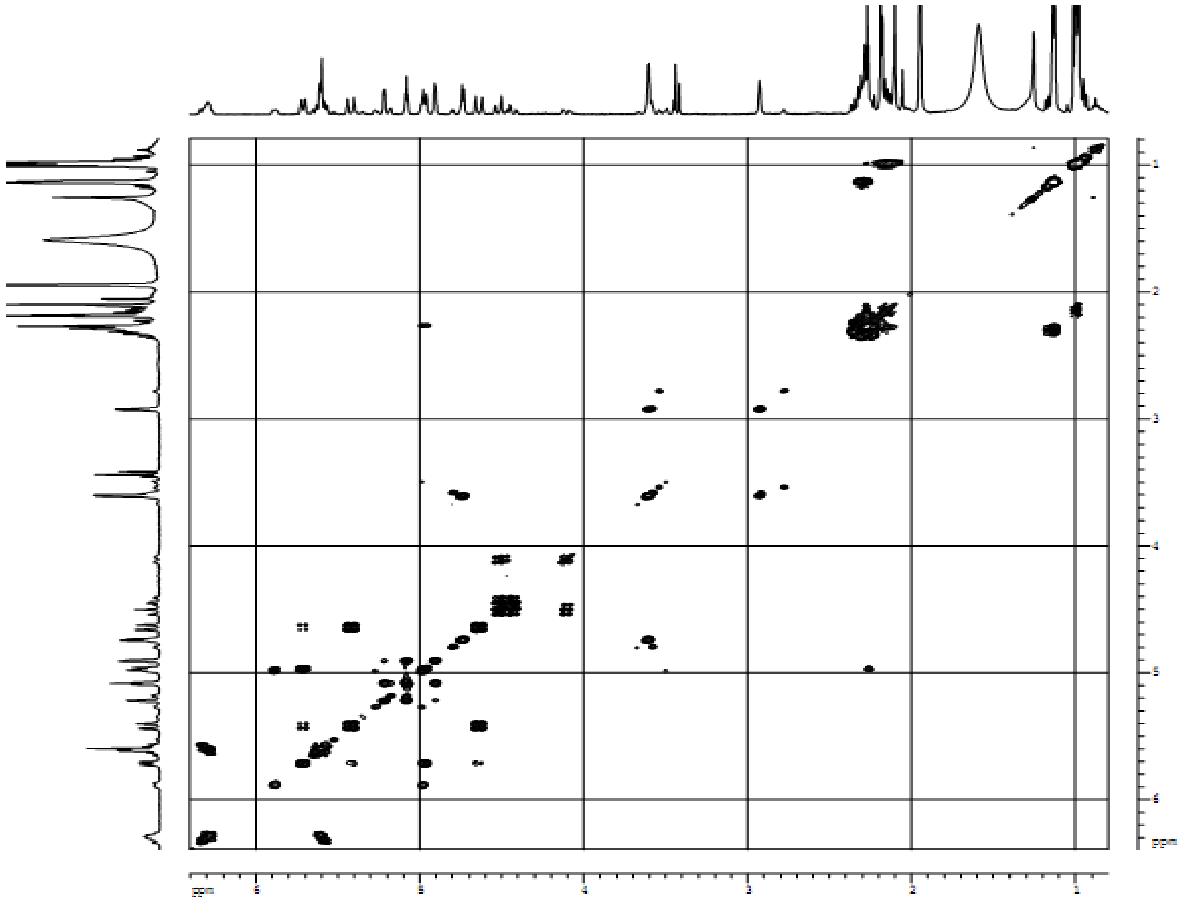

Figure S47. HMBC spectrum of the new compound **6**.

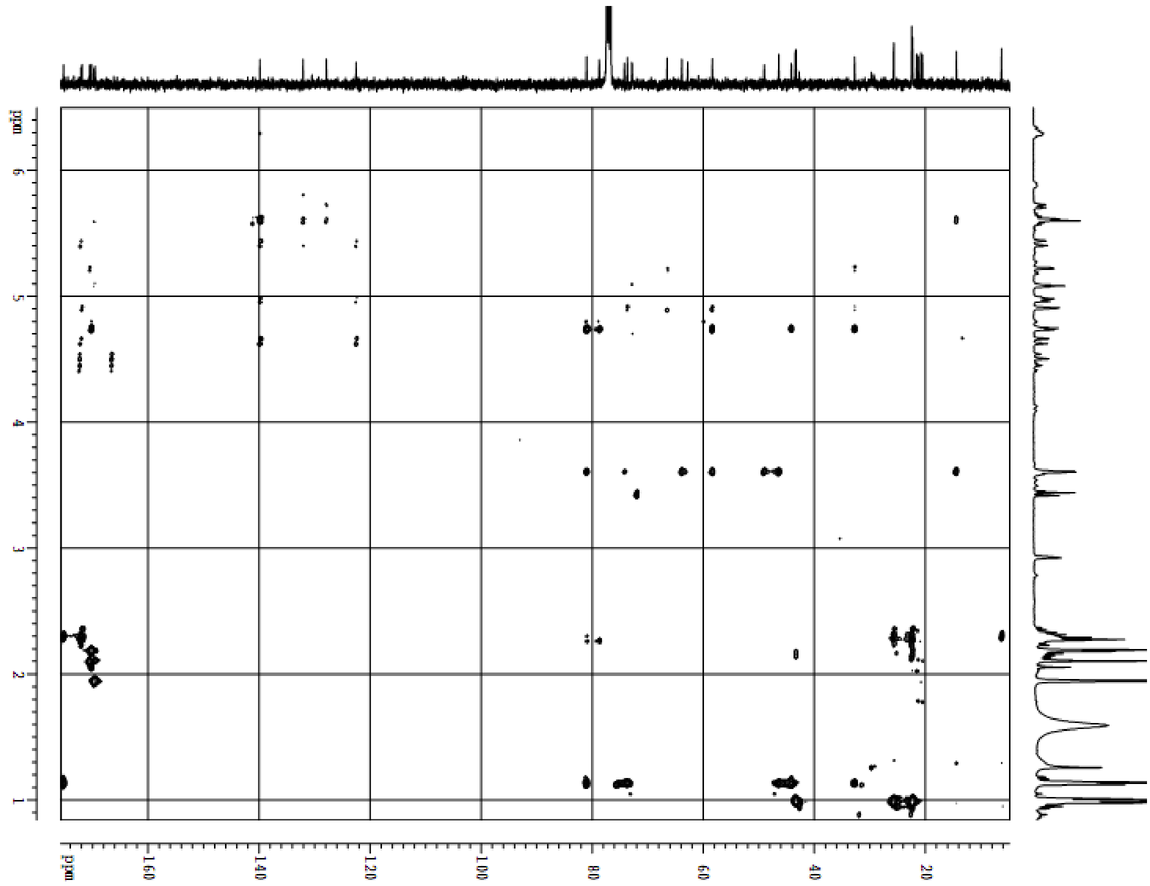

Figure S48. NOESY spectrum of the new compound **6**.

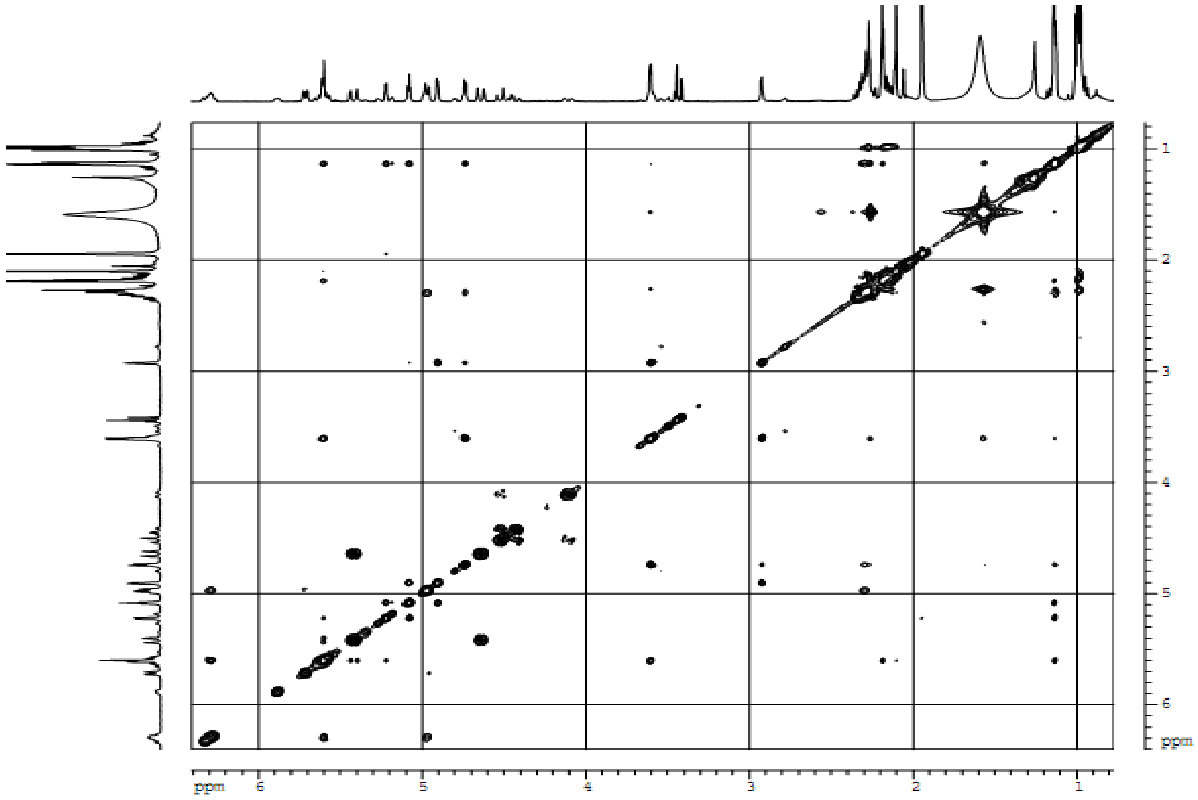

Figure S49. HRESIMS spectrum of compound **7**.

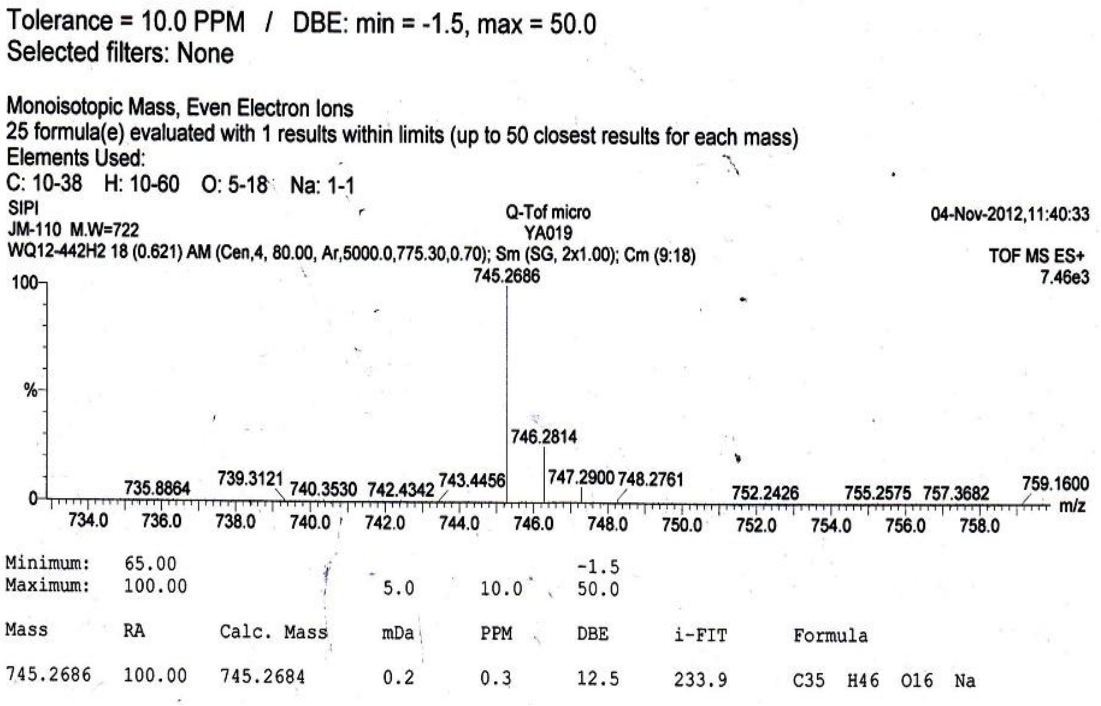

Figure S50. ^{1}H NMR spectrum of compound **7** in $CDCl_3$.

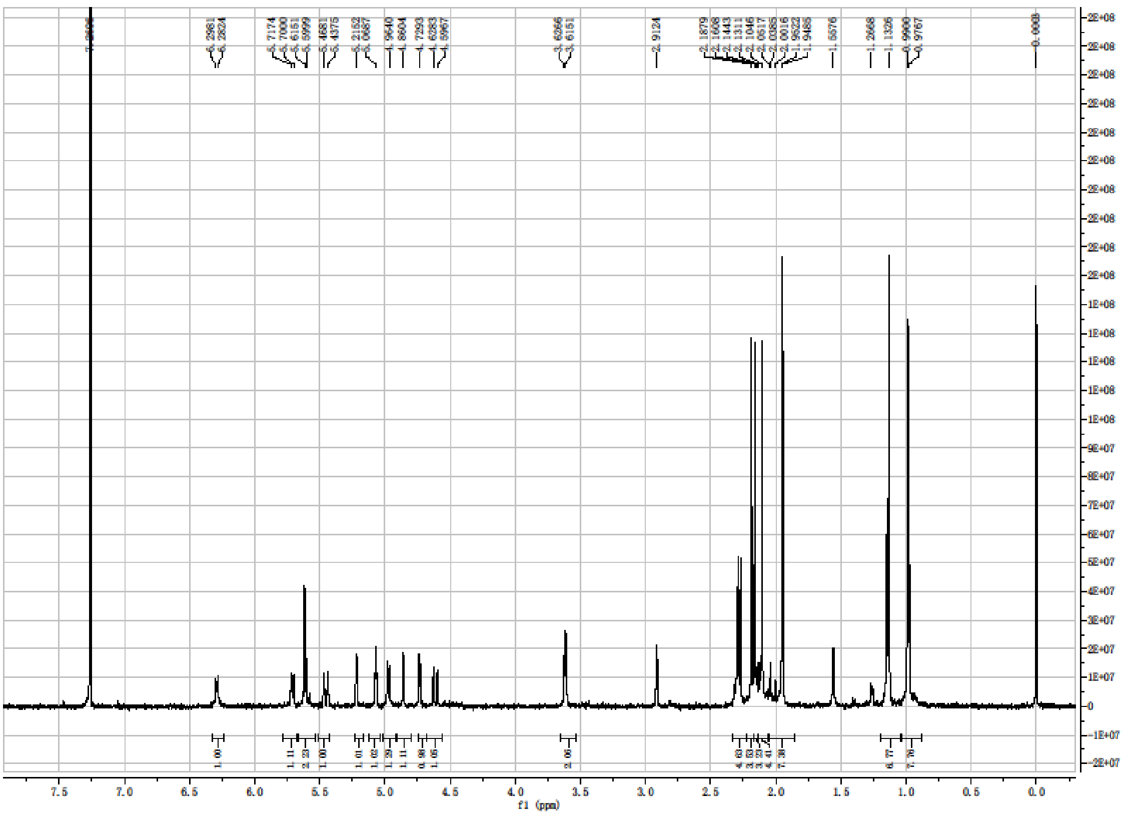

Figure S51. ^{13}C NMR spectrum of compound **7** in $CDCl_3$.

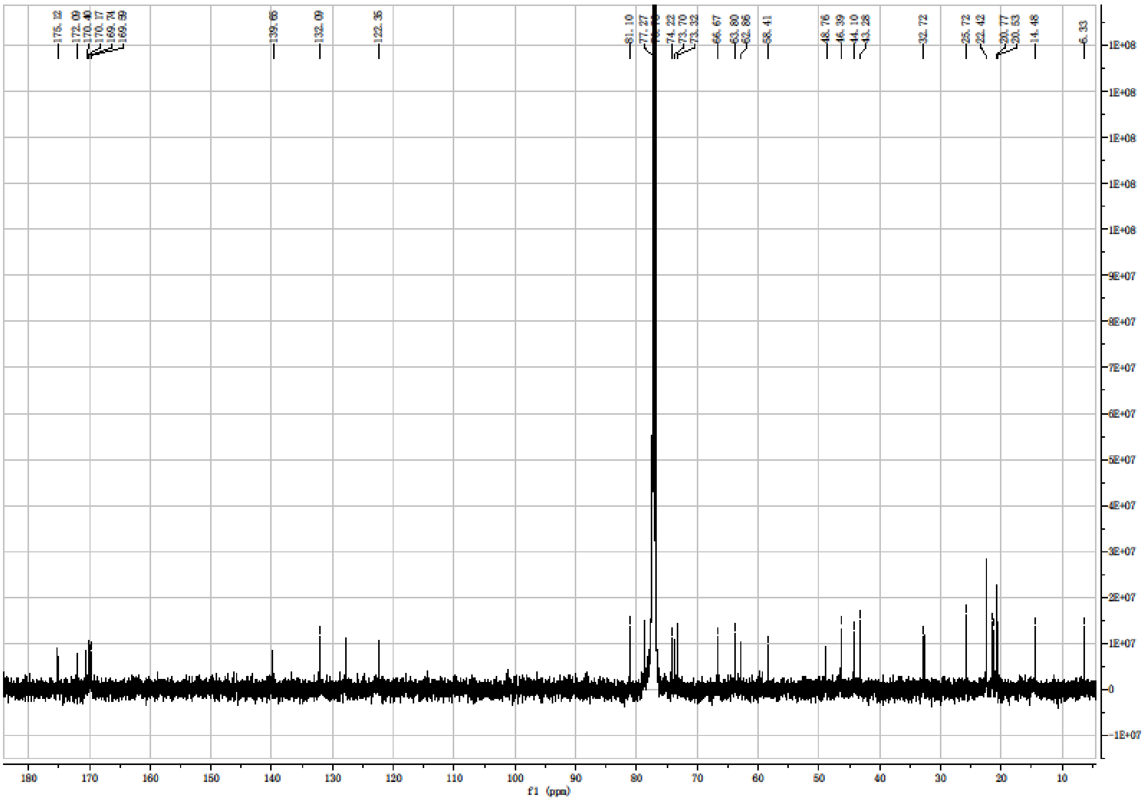

Figure S52. DEPT spectrum of compound **7** in $CDCl_3$.

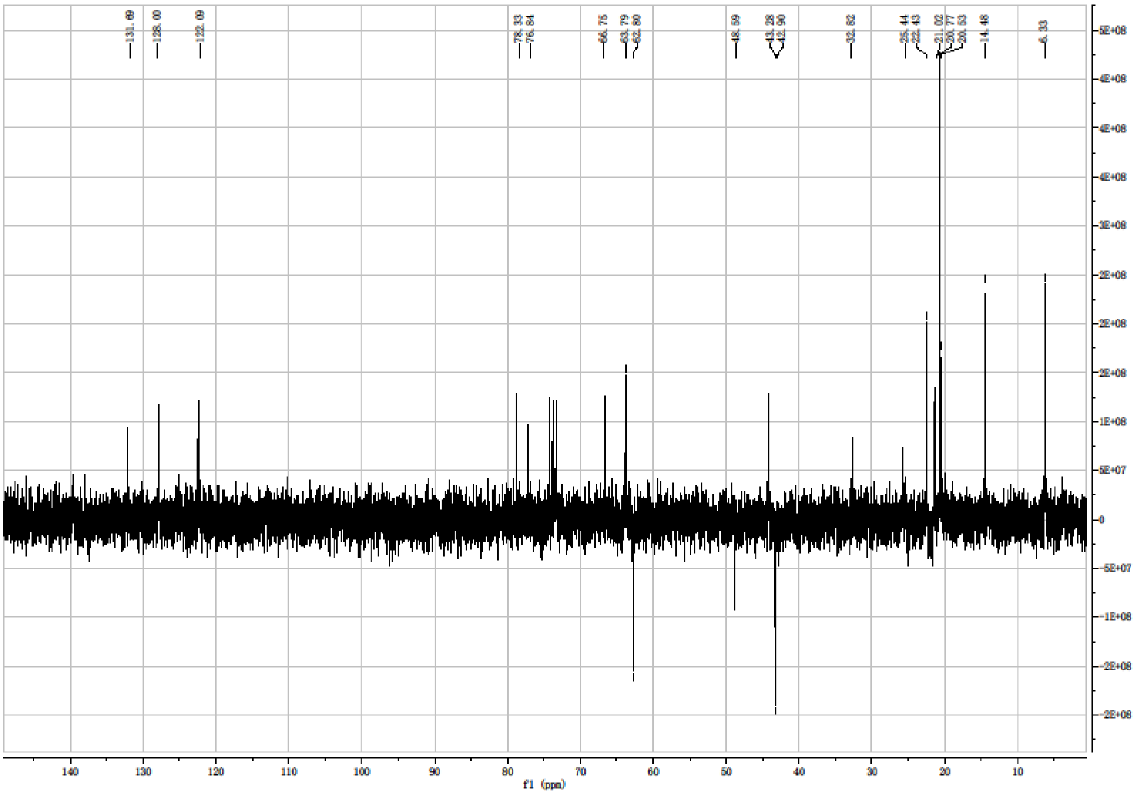

Figure S53. HSQC spectrum of compound **7** in $CDCl_3$.

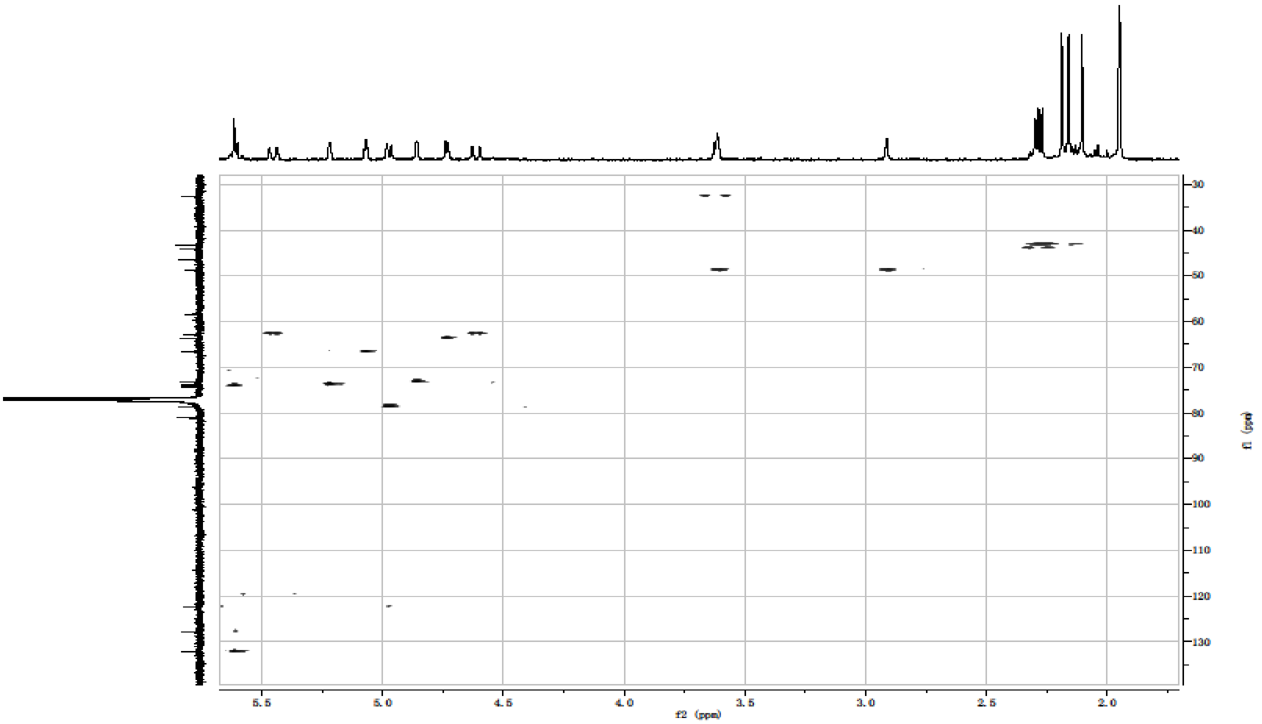

Figure S54. ^1H-^1H COSY spectrum of compound **7** in $CDCl_3$.

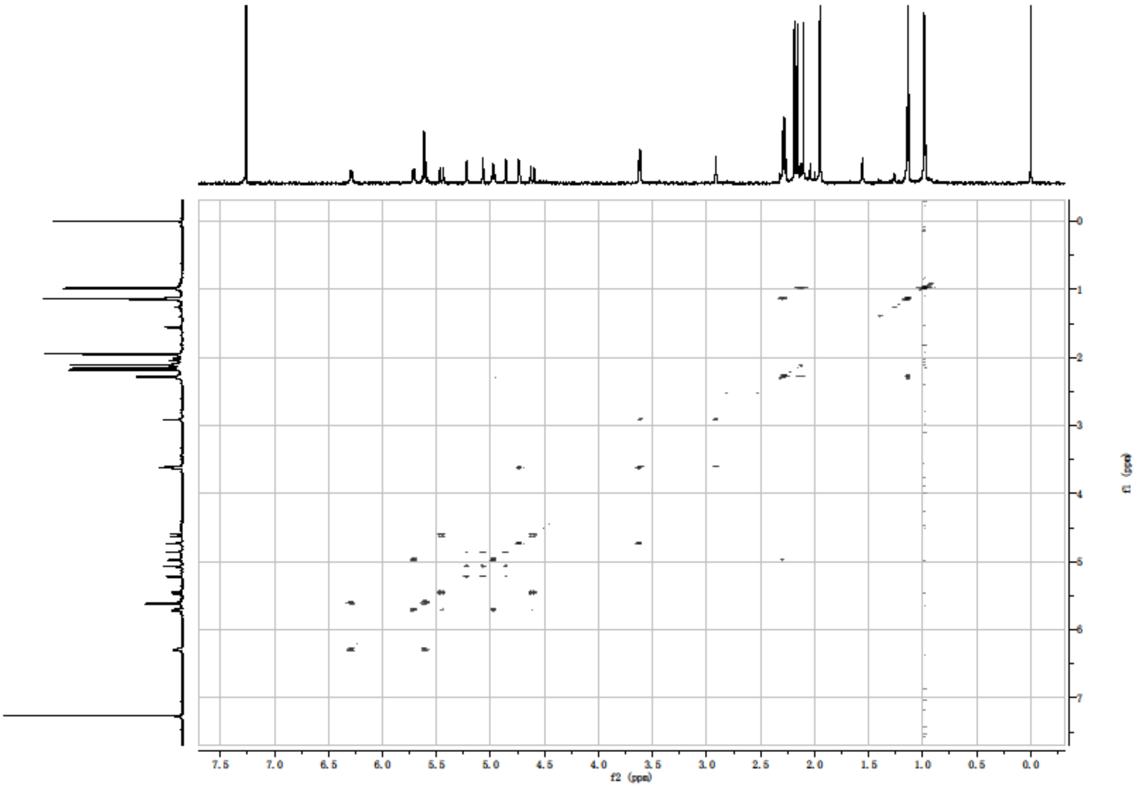

Figure S55. HMBC spectrum of compound **7** in $CDCl_3$.

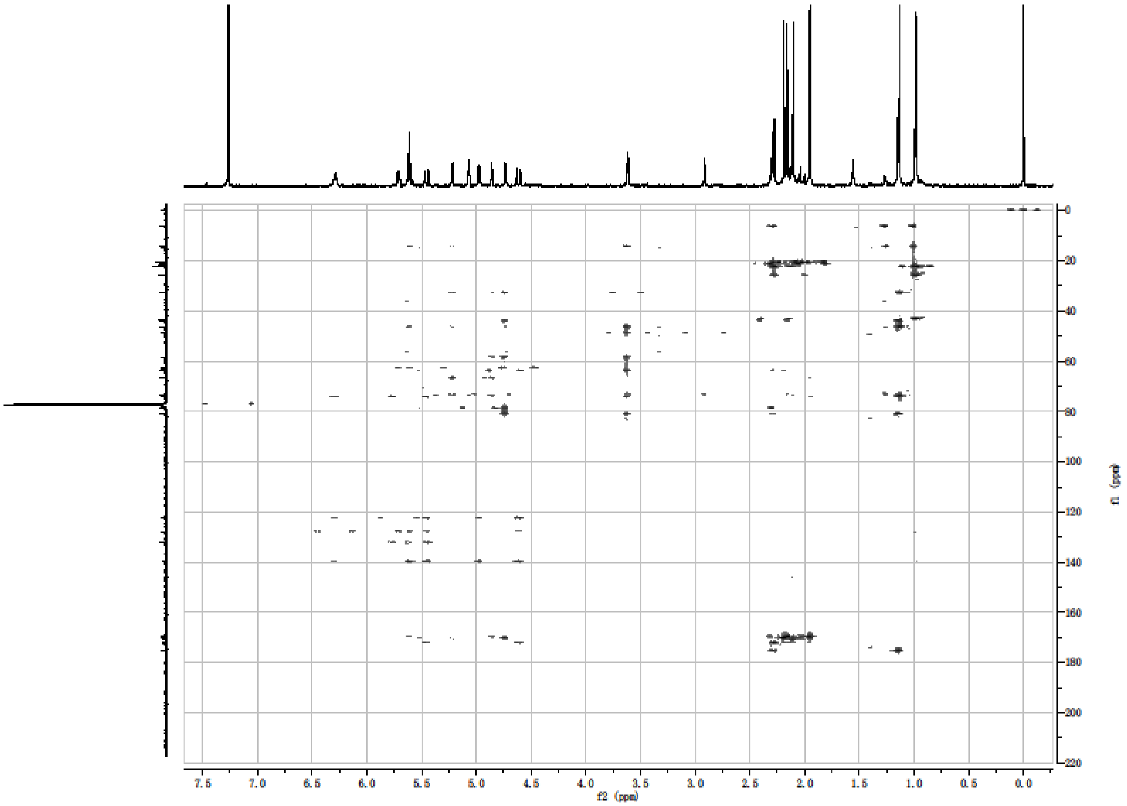

Figure S56. NOESY spectrum of compound **7** in $CDCl_3$.

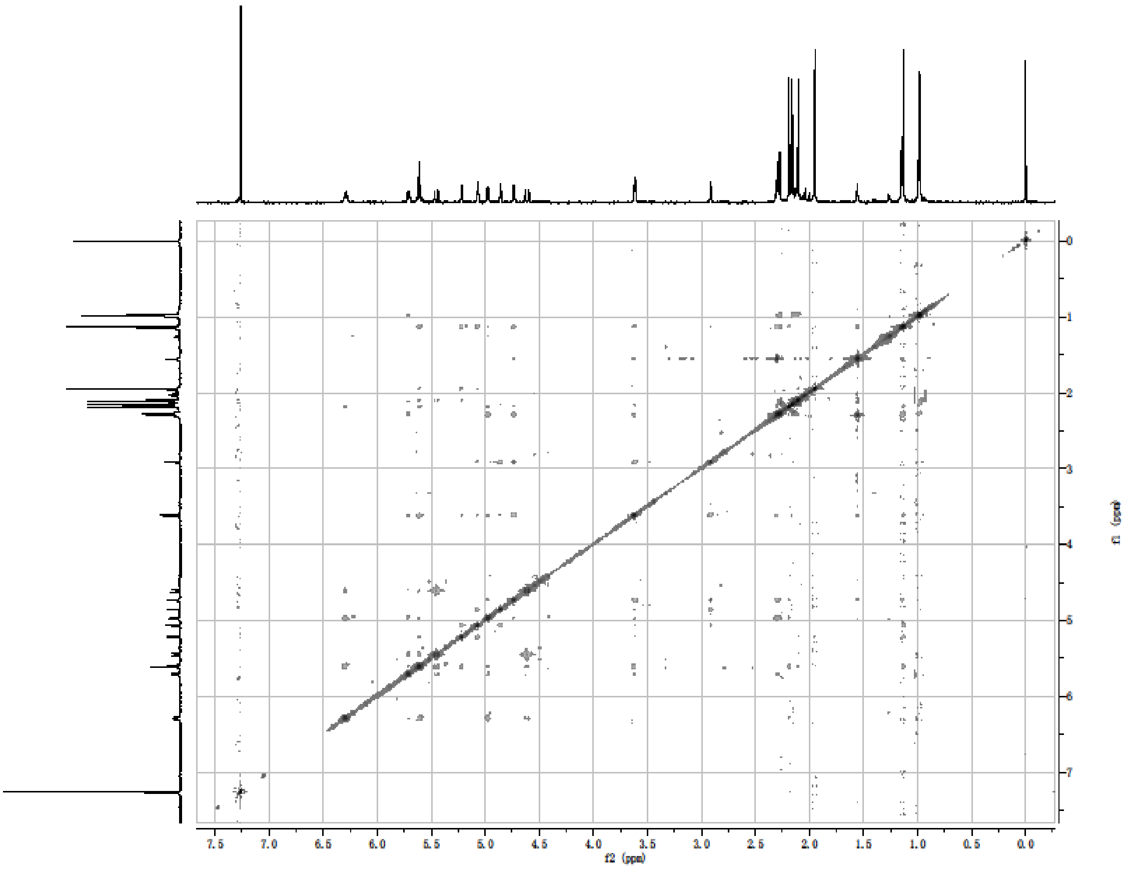

Figure S57. HR-ESIMS spectrum of the new compound **8**.

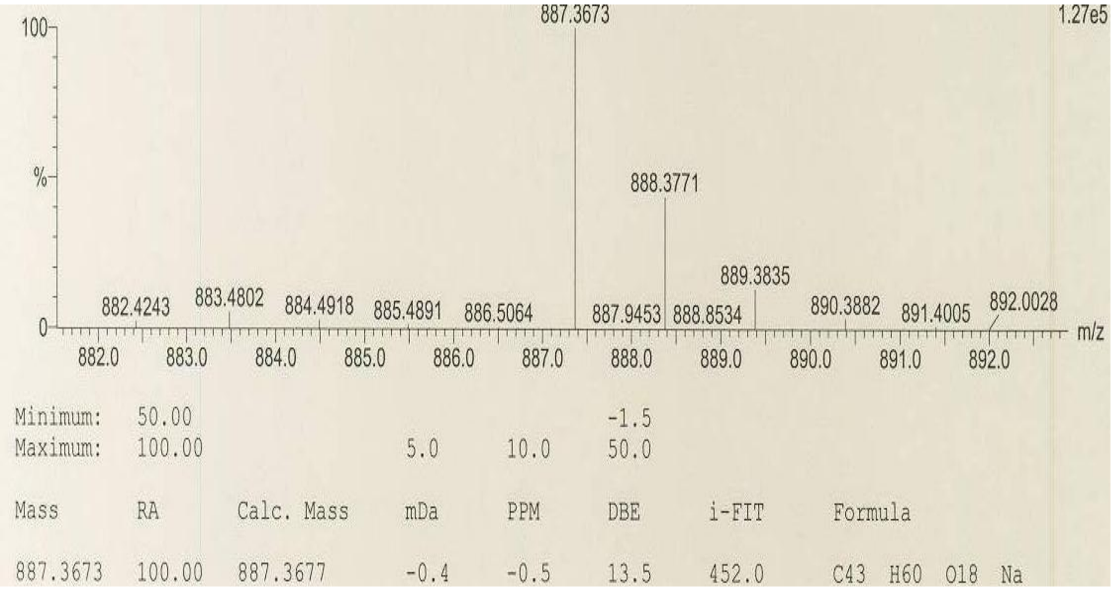

Figure S58. ^{1}H NMR spectrum of the new compound **8**.

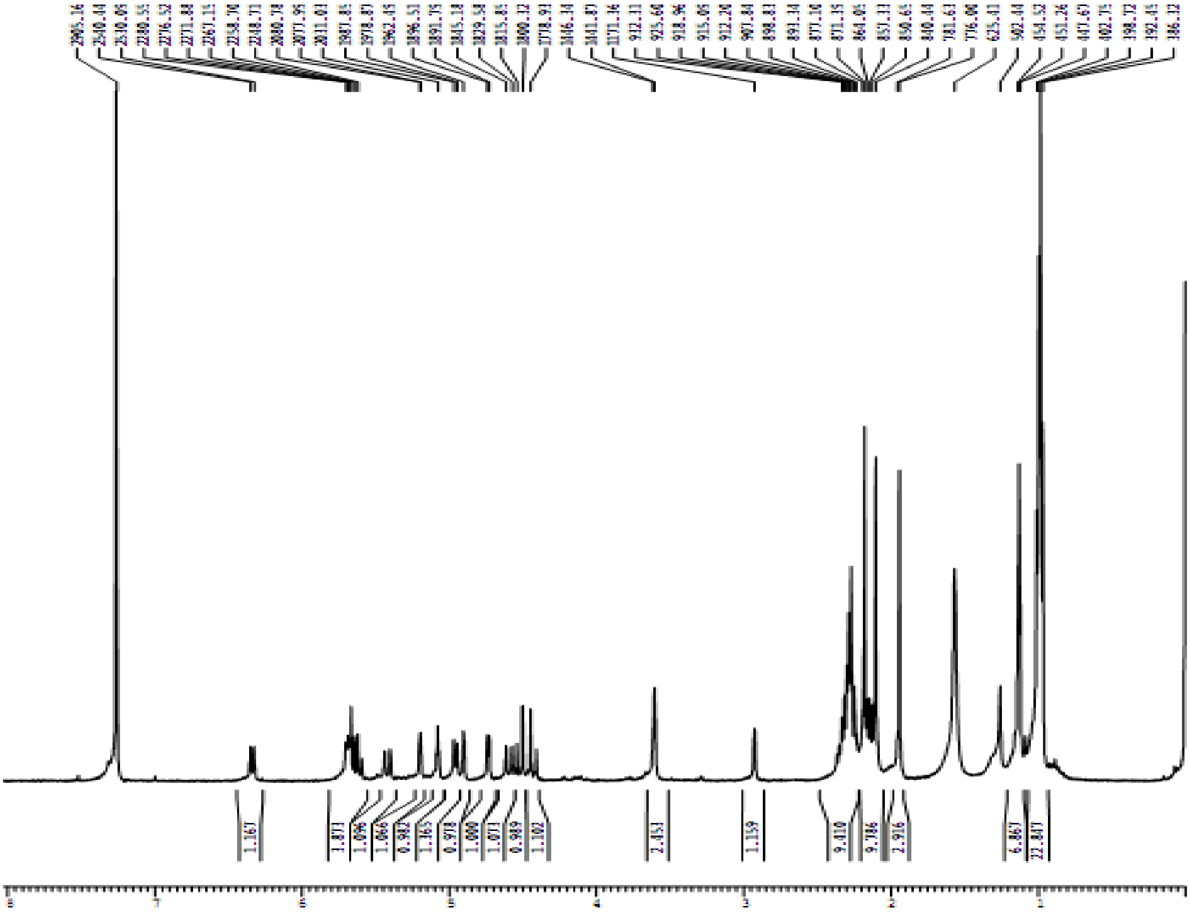

Figure S59. ^{13}C NMR spectrum of the new compound **8**.

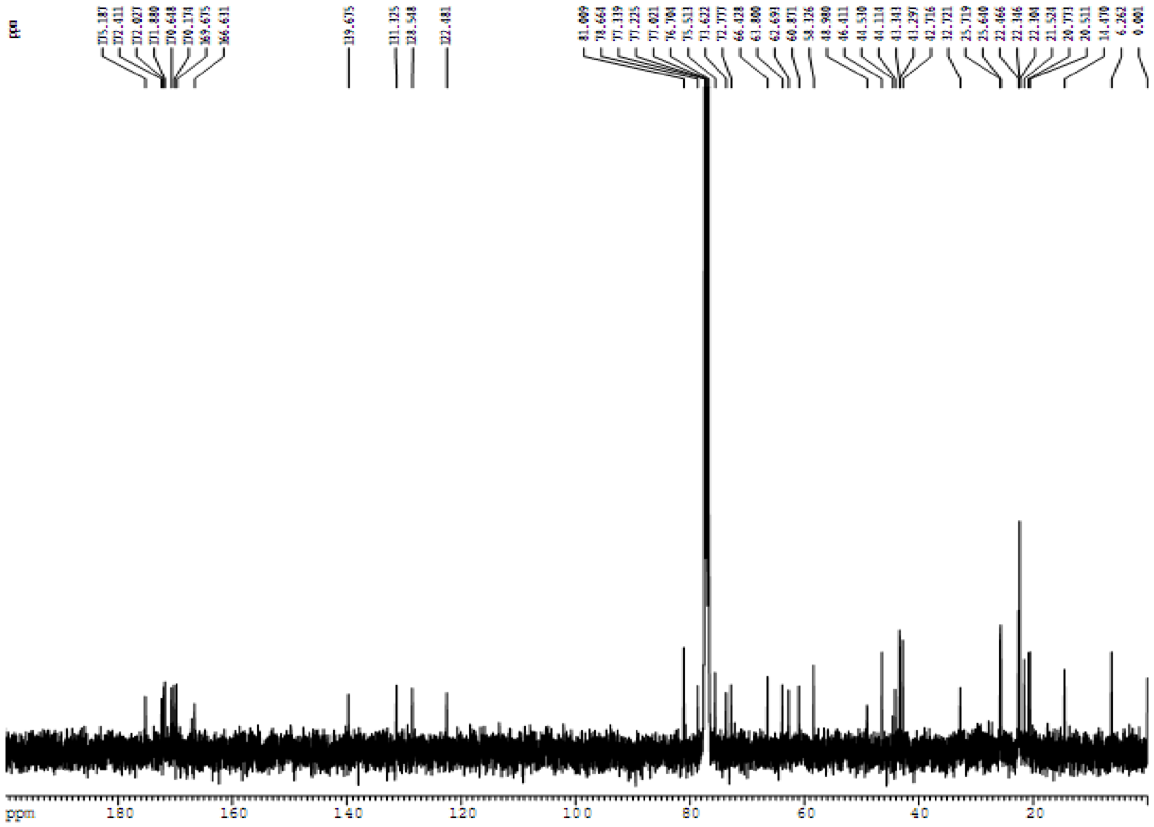

Figure S60. DEPT spectrum of the new compound **8**.

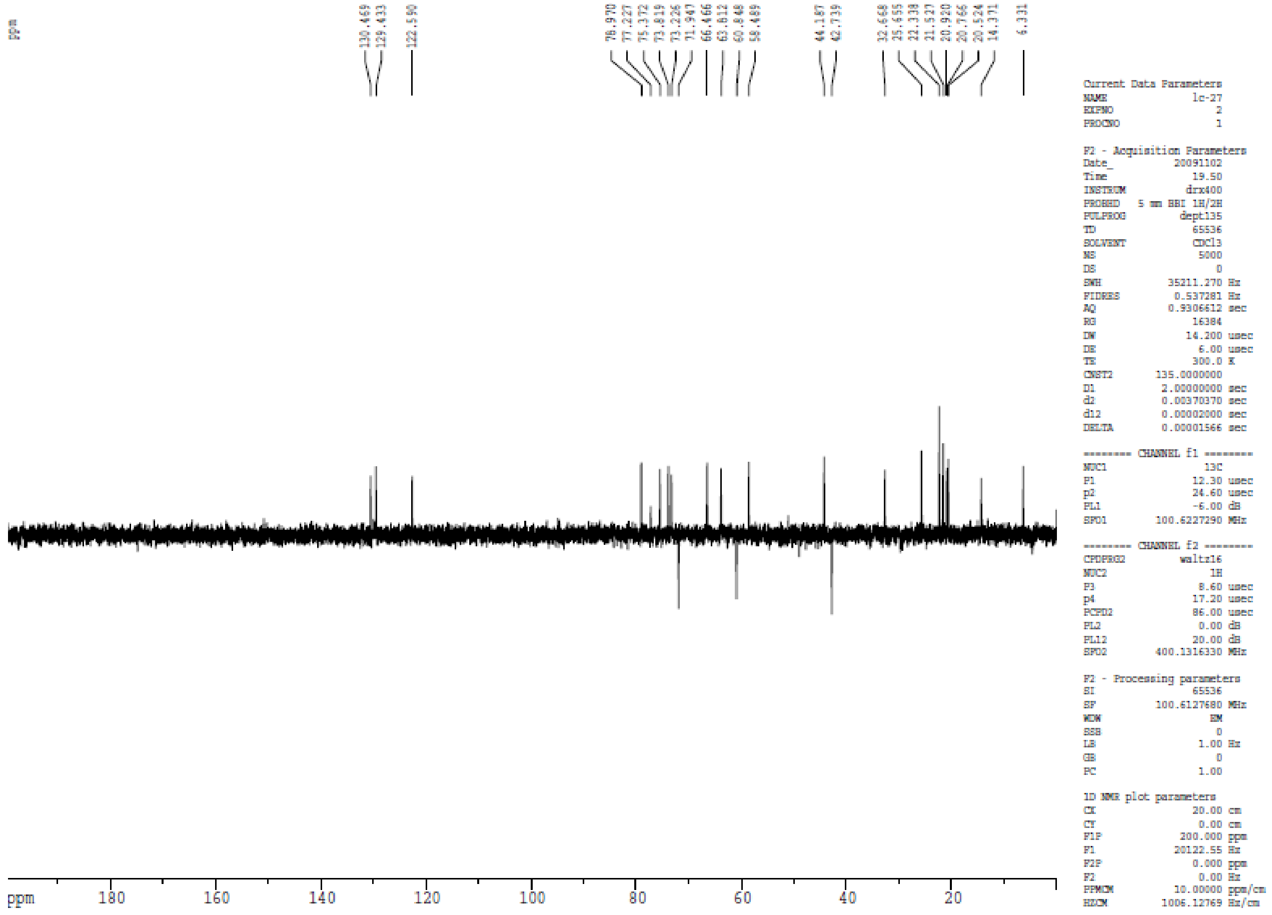

Figure S61. HSQC spectrum of the new compound **8**.

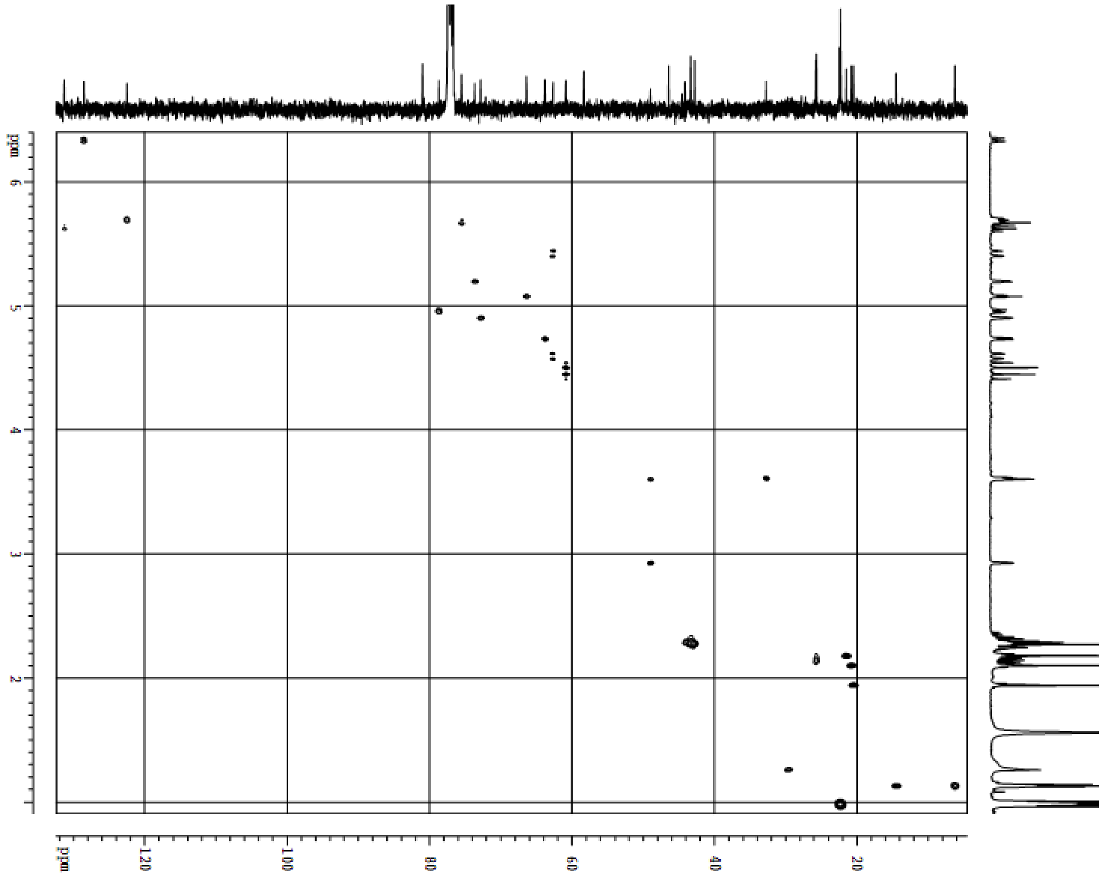

Figure S62. ^{1}H-^{1}H COSY spectrum of the new compound **8**.

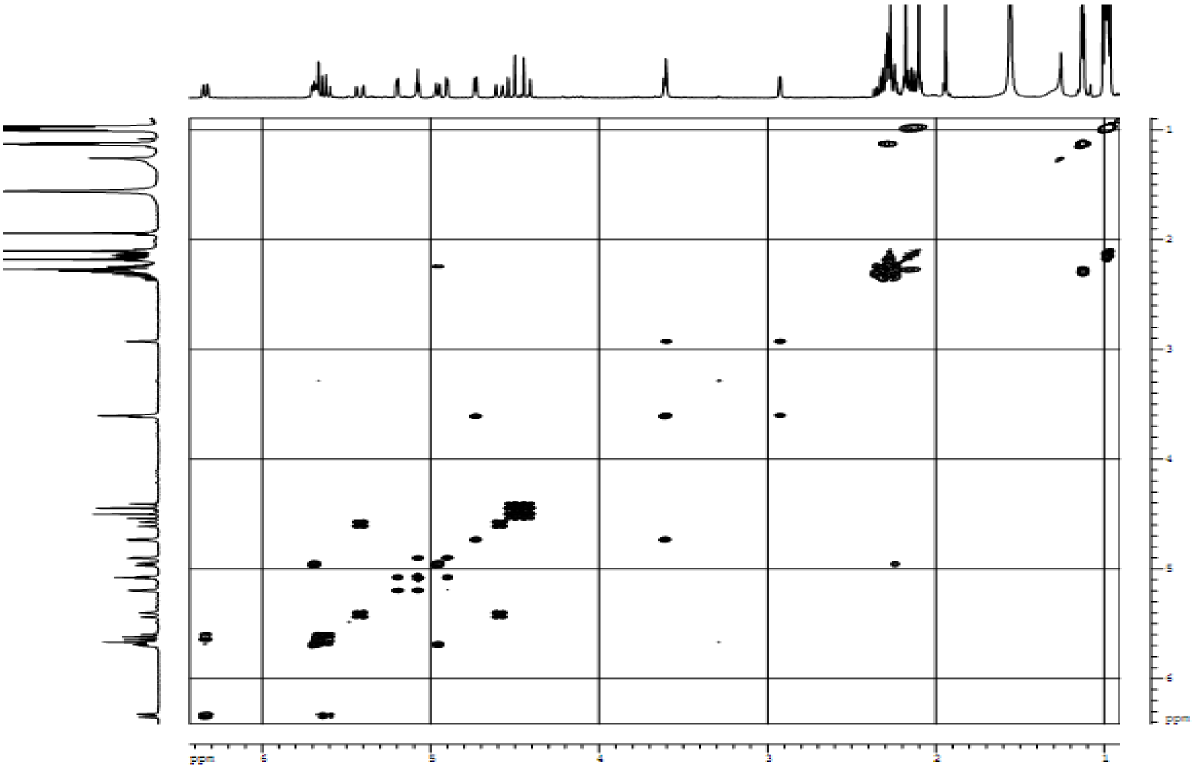

Figure S63. HMBC spectrum of the new compound **8**.

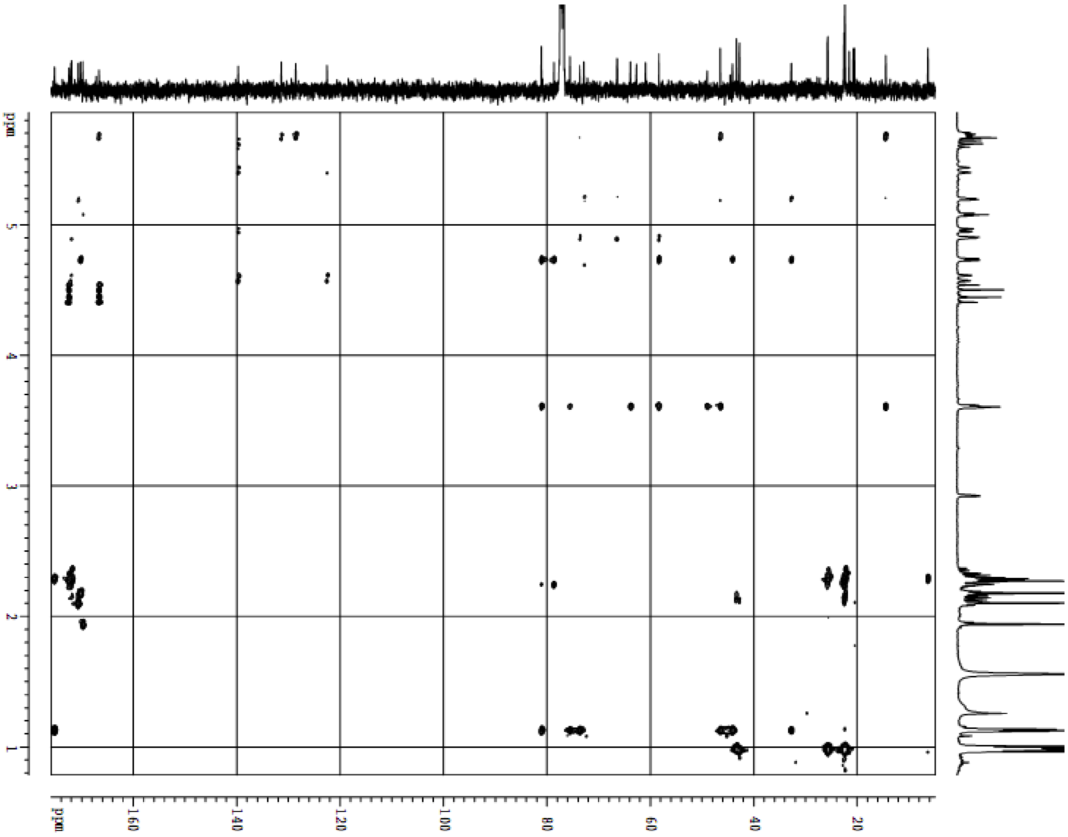

Figure S64. NOESY spectrum of the new compound **8**.

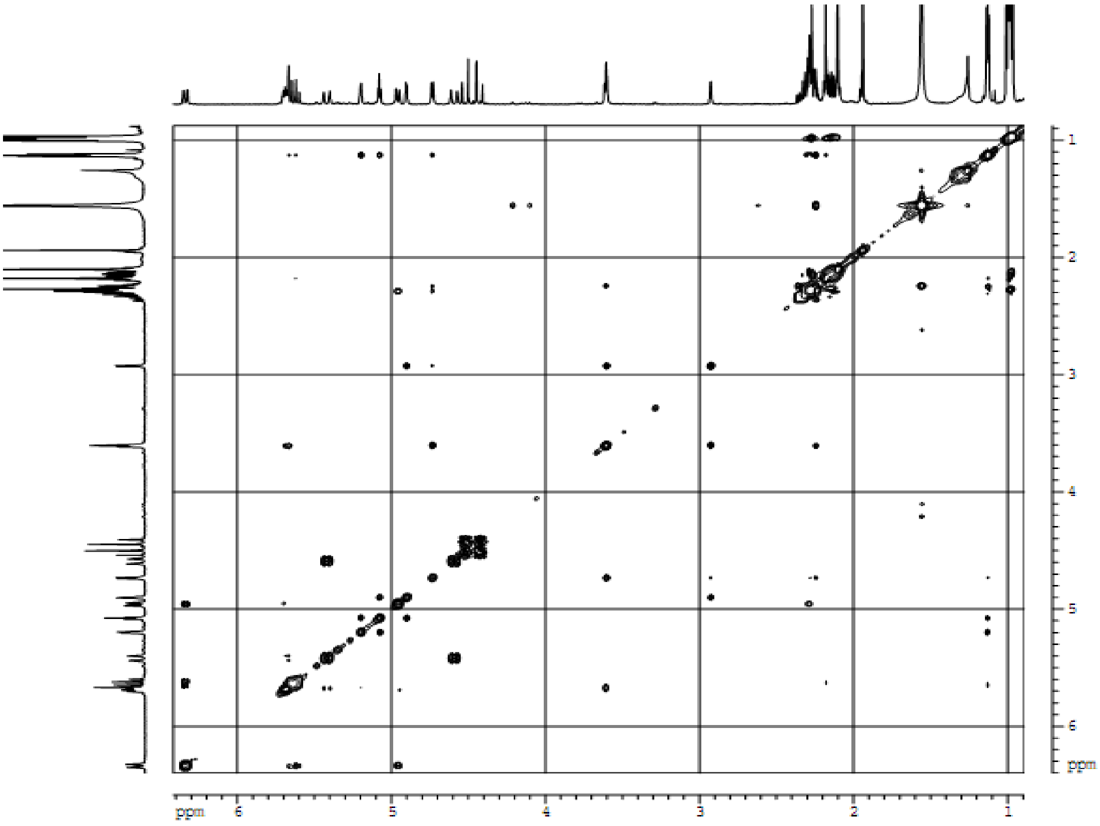

Figure S65. HRESIMS spectrum of compound **9**.

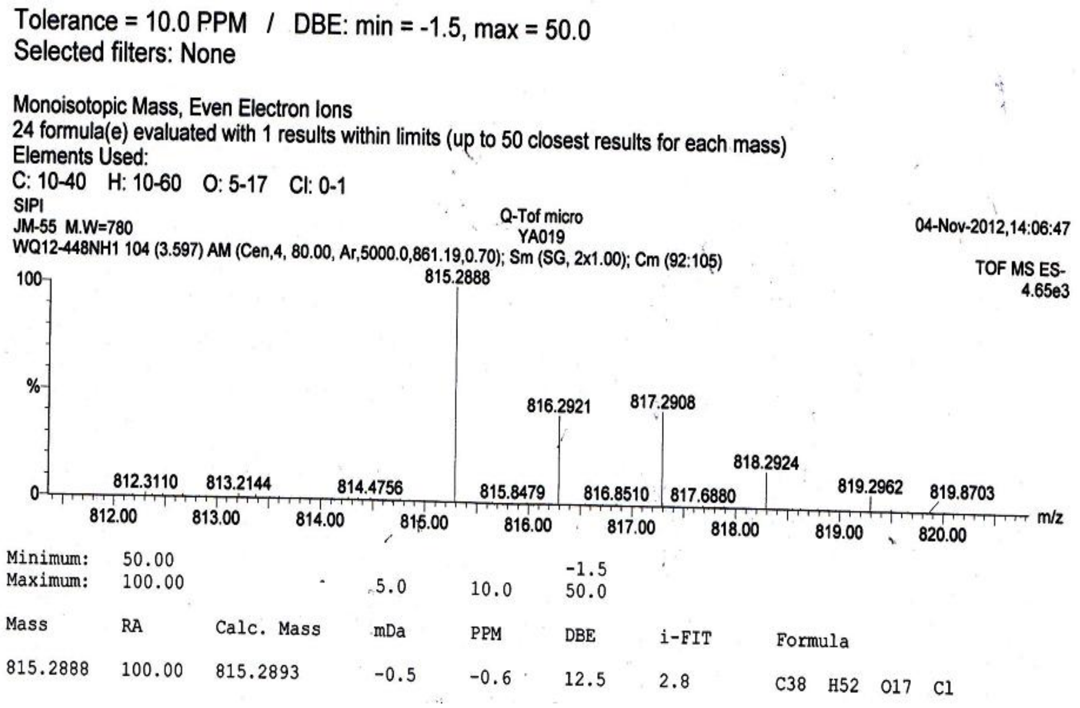

Figure S66. ^1H NMR spectrum of compound **9** in $CDCl_3$.

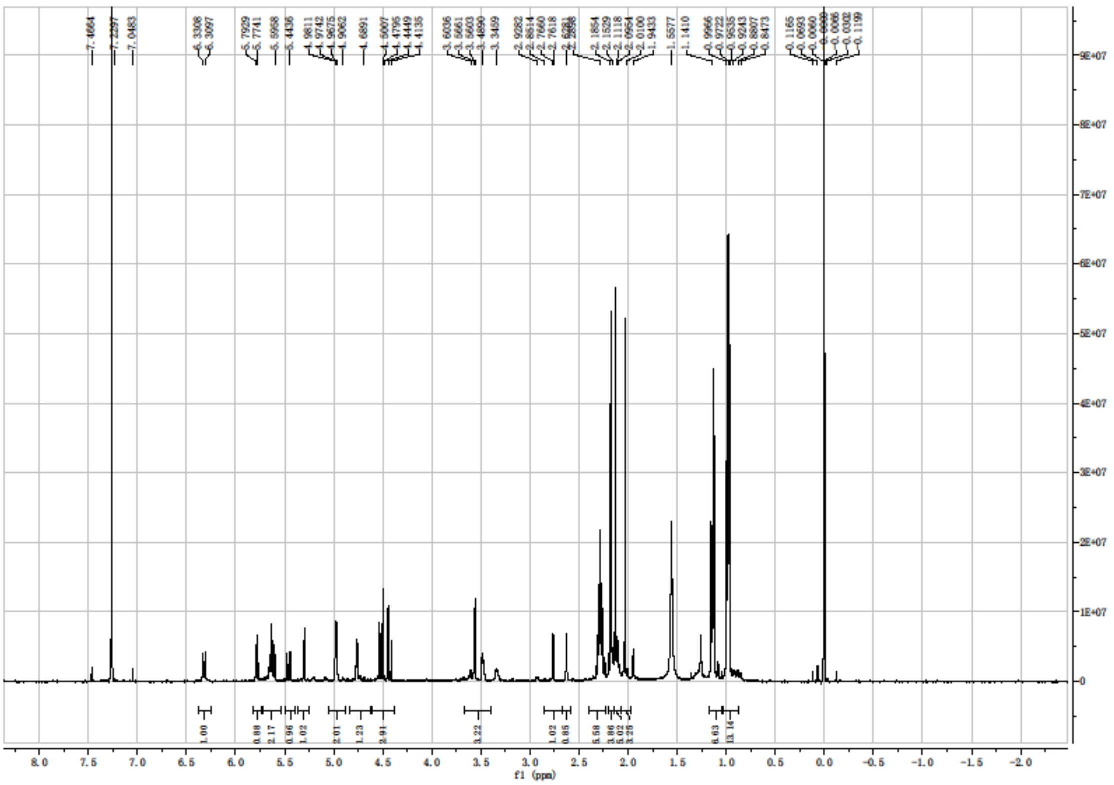

Figure S67. ^{13}C NMR spectrum of compound **9** in $CDCl_3$.

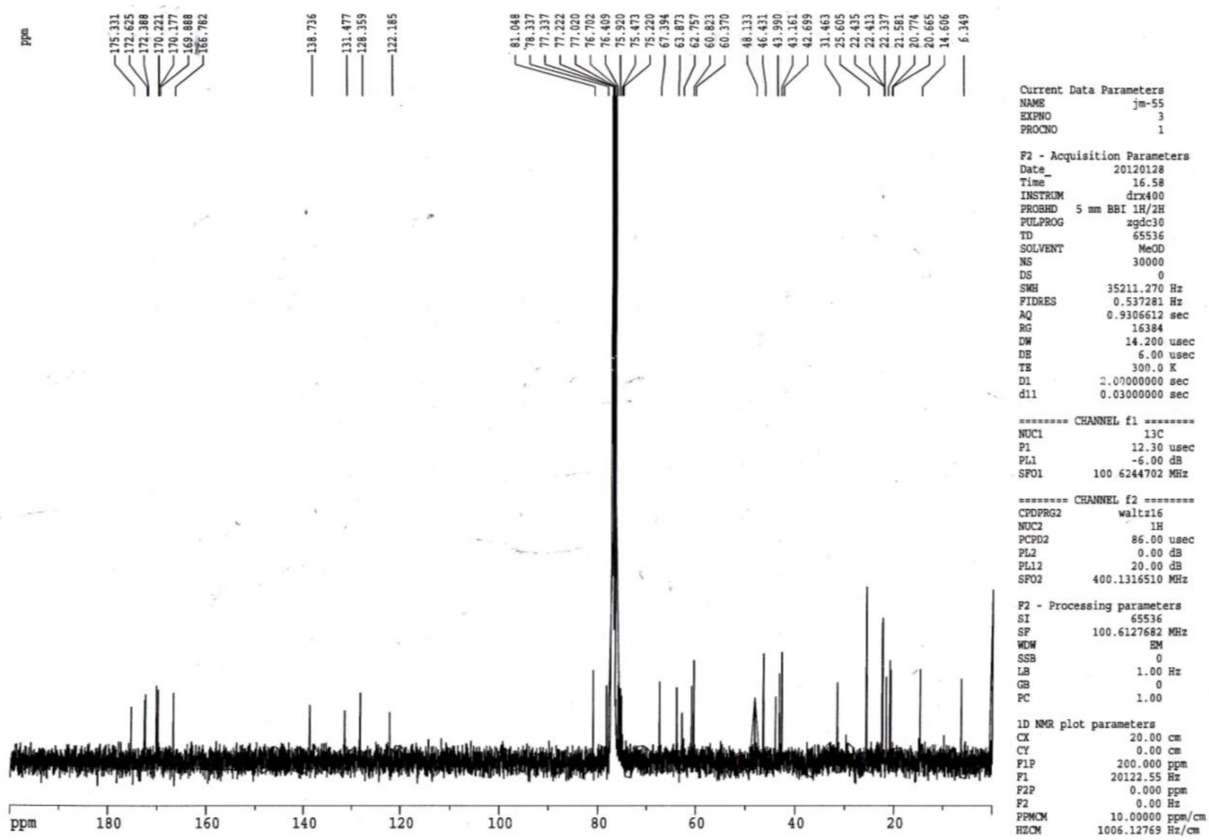

Figure S68. DEPT spectrum of compound **9** in $CDCl_3$.

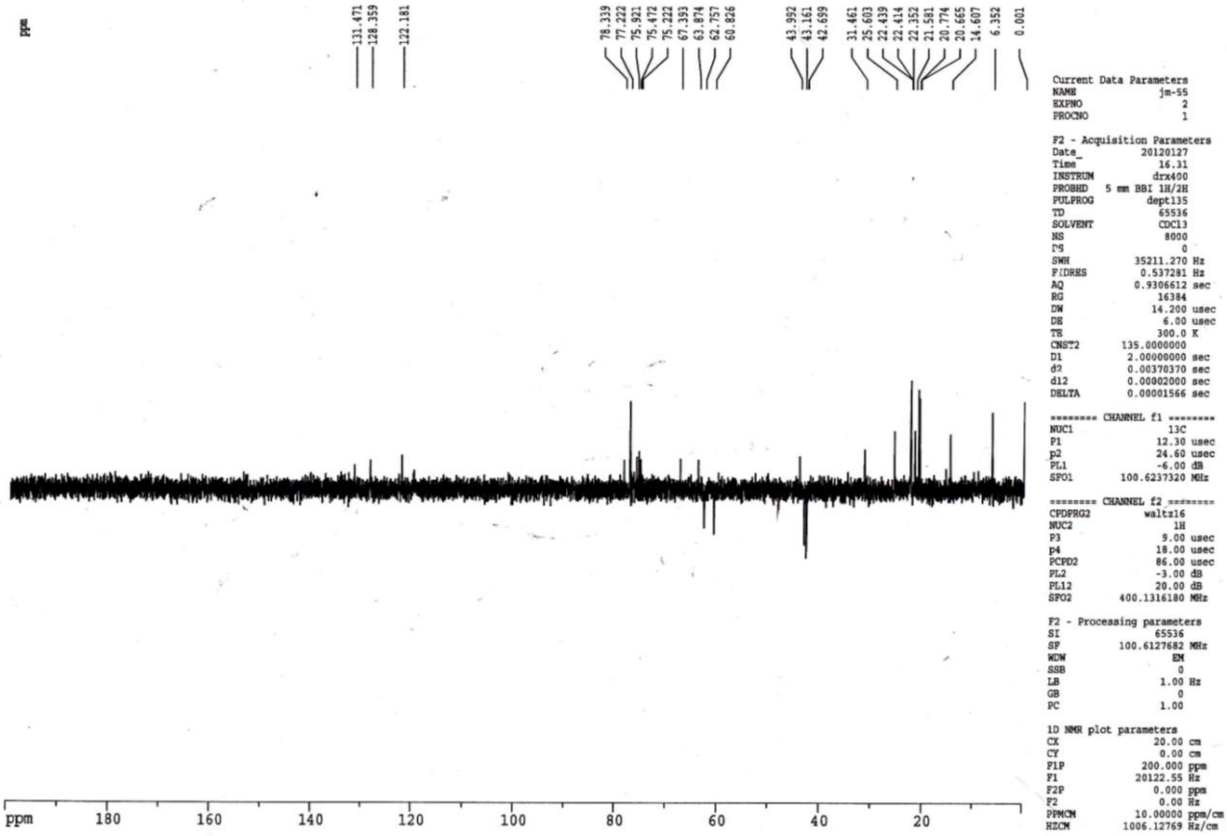

Figure S69. HSQC spectrum of compound **9** in $CDCl_3$.

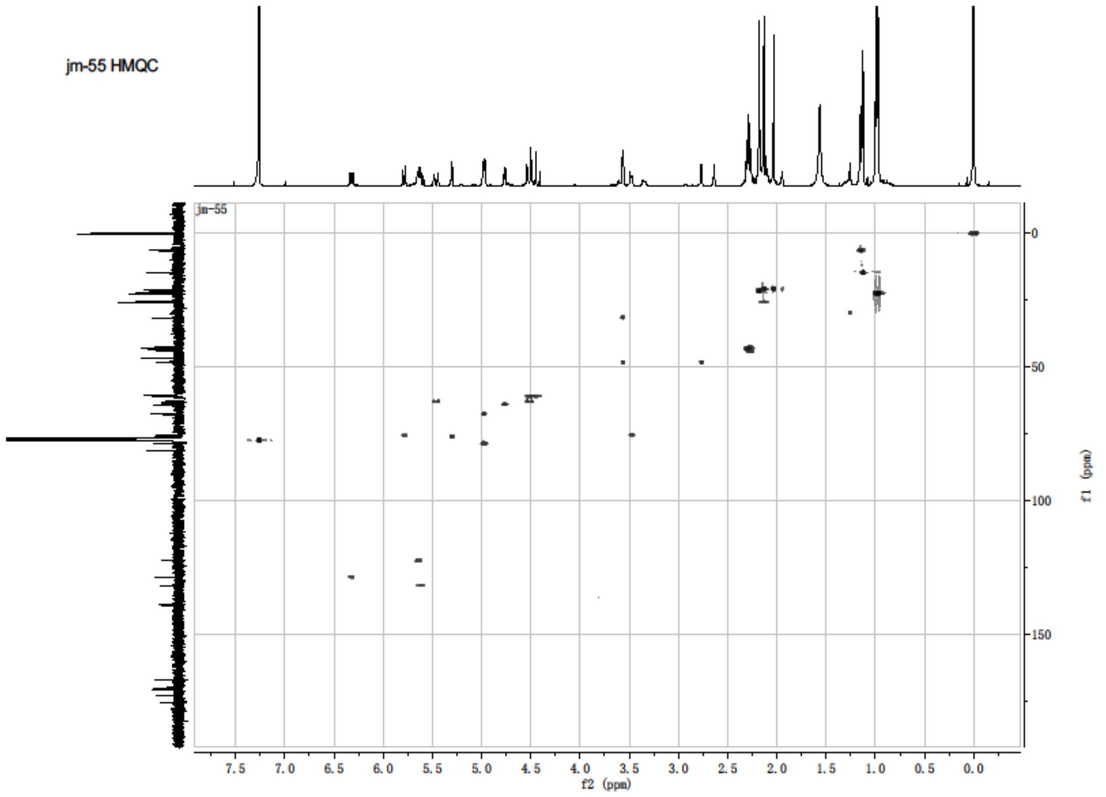

Figure S70. ^1H-^1H COSY spectrum of compound **9** in $CDCl_3$.

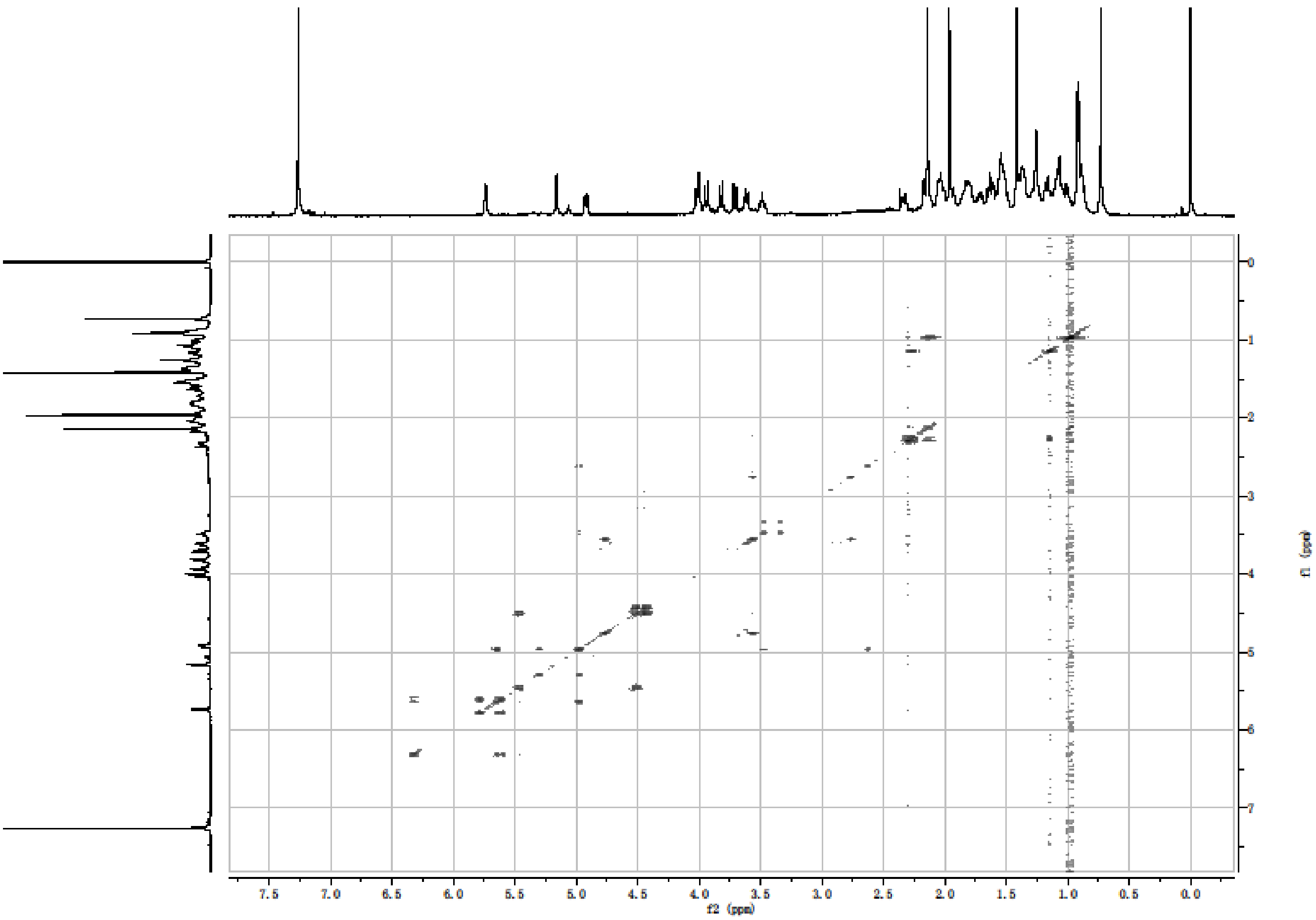

Figure S71. HMBC spectrum of compound **9** in $CDCl_3$.

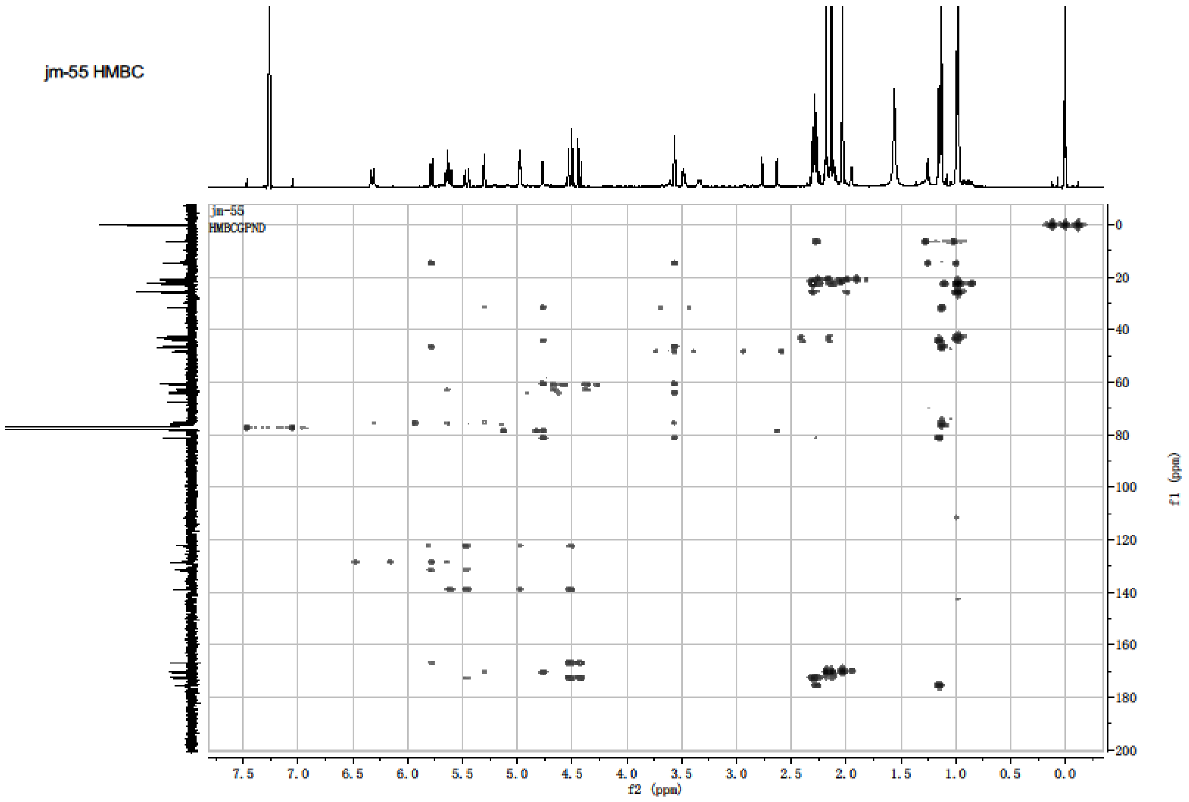

Figure S72. NOESY spectrum of compound **9** in $CDCl_3$.

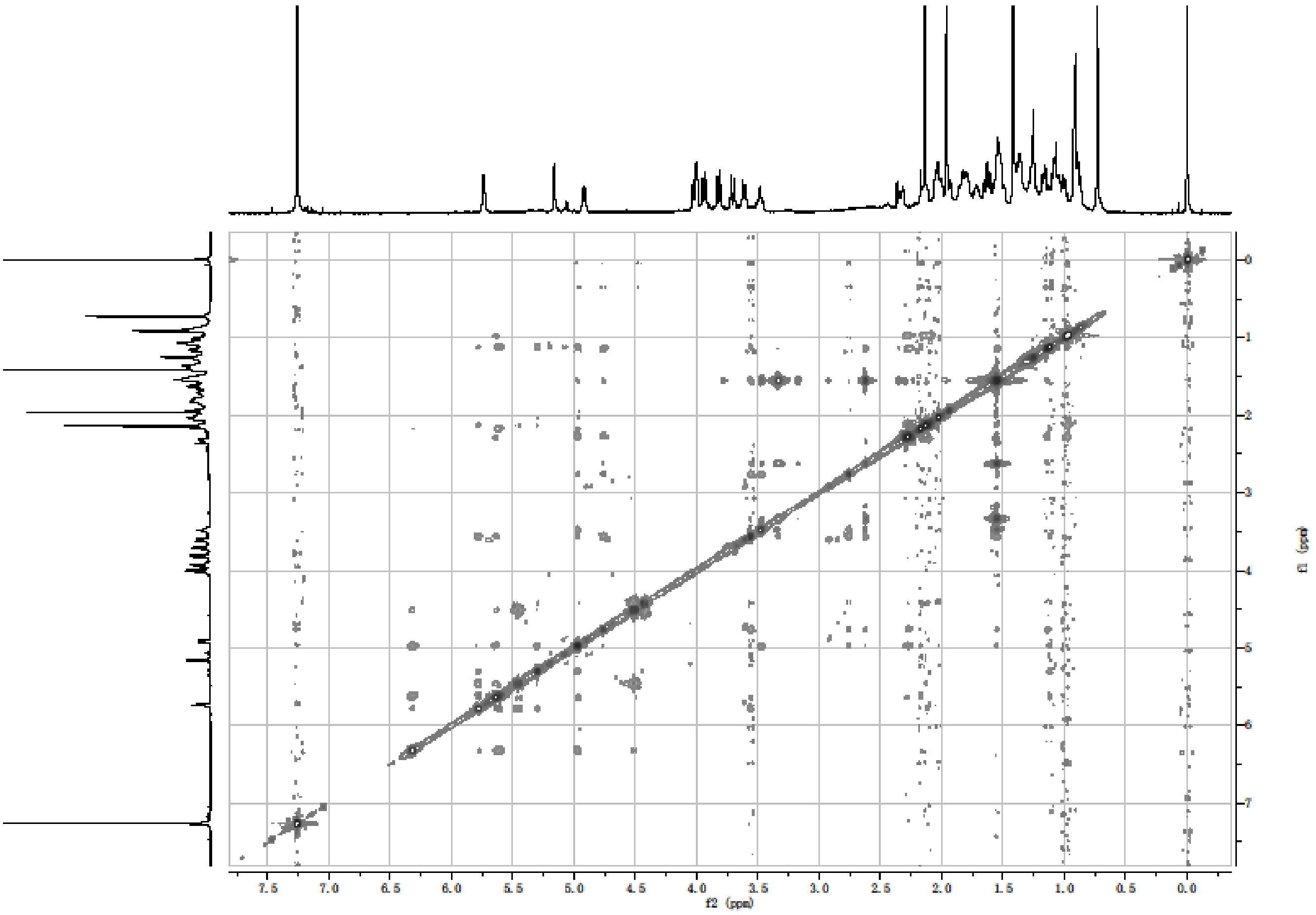

Figure S73. HR-ESIMS spectrum of the new compound **10**.

Multiple Mass Analysis: 2 mass(es) processed
Tolerance = 10.0 PPM / DBE: min = -1.5, max = 50.0
Selected filters: None

Monoisotopic Mass, Even Electron Ions
12 formula(e) evaluated with 1 results within limits (up to 50 closest results for each mass)
Elements Used:
C: 5-38 H: 10-60 O: 3-16 Na: 1-1 Cl: 1-1

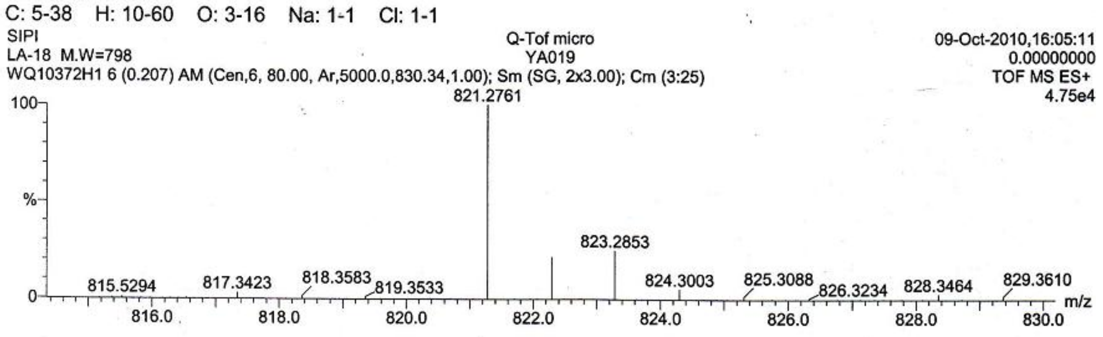

Minimum:	25.00				-1.5		
Maximum:	100.00		5.0	10.0	50.0		
Mass	RA	Calc. Mass	mDa	PPM	DBE	i-FIT	Formula
821.2761	100.00	821.2763	-0.2	-0.2	12.5	3469.0	C38 H51 O16 Na Cl

Figure S74. ^{1}H NMR spectrum of the new compound **10**.

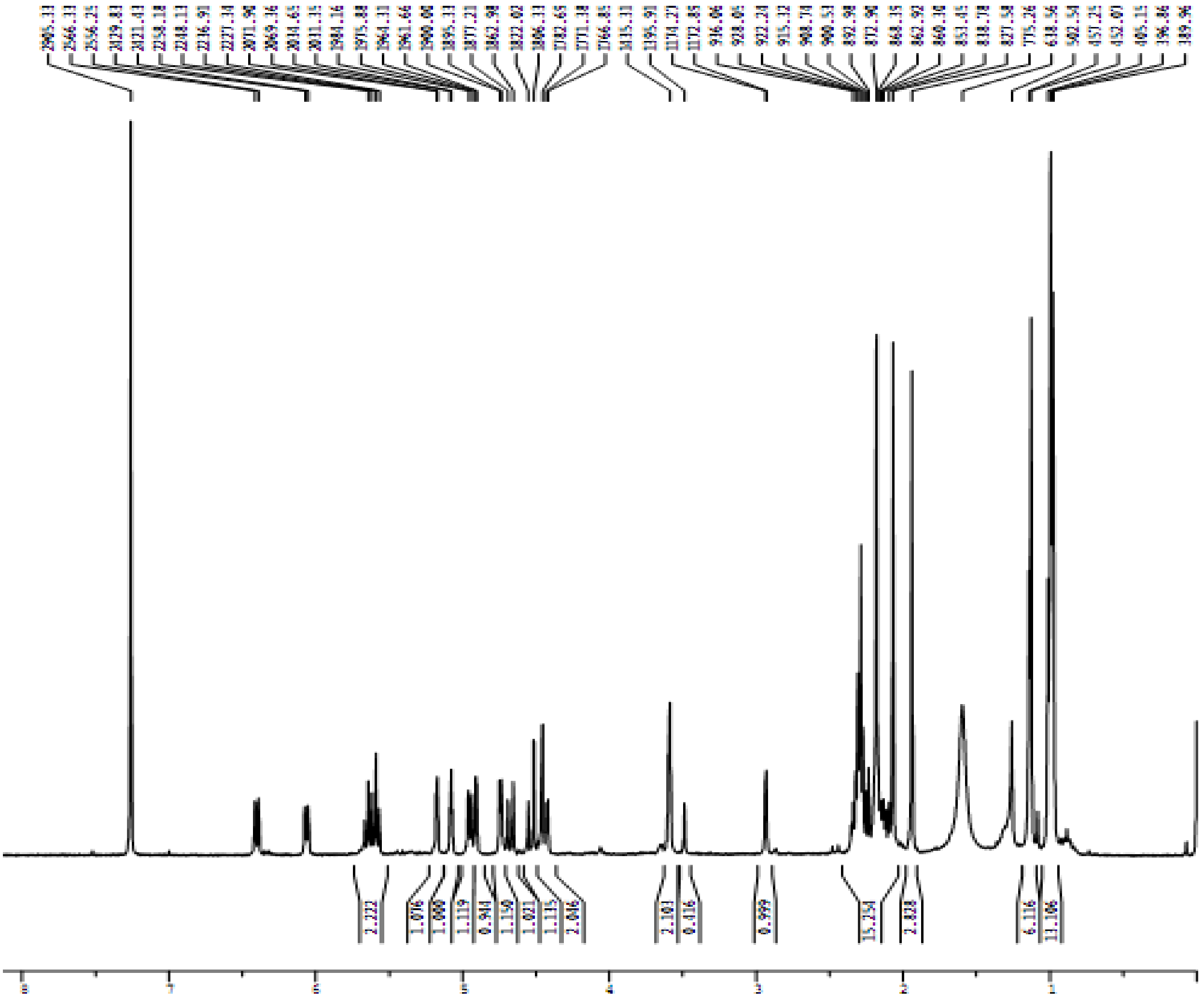

Figure S75. ^{13}C NMR spectrum of the new compound **10**.

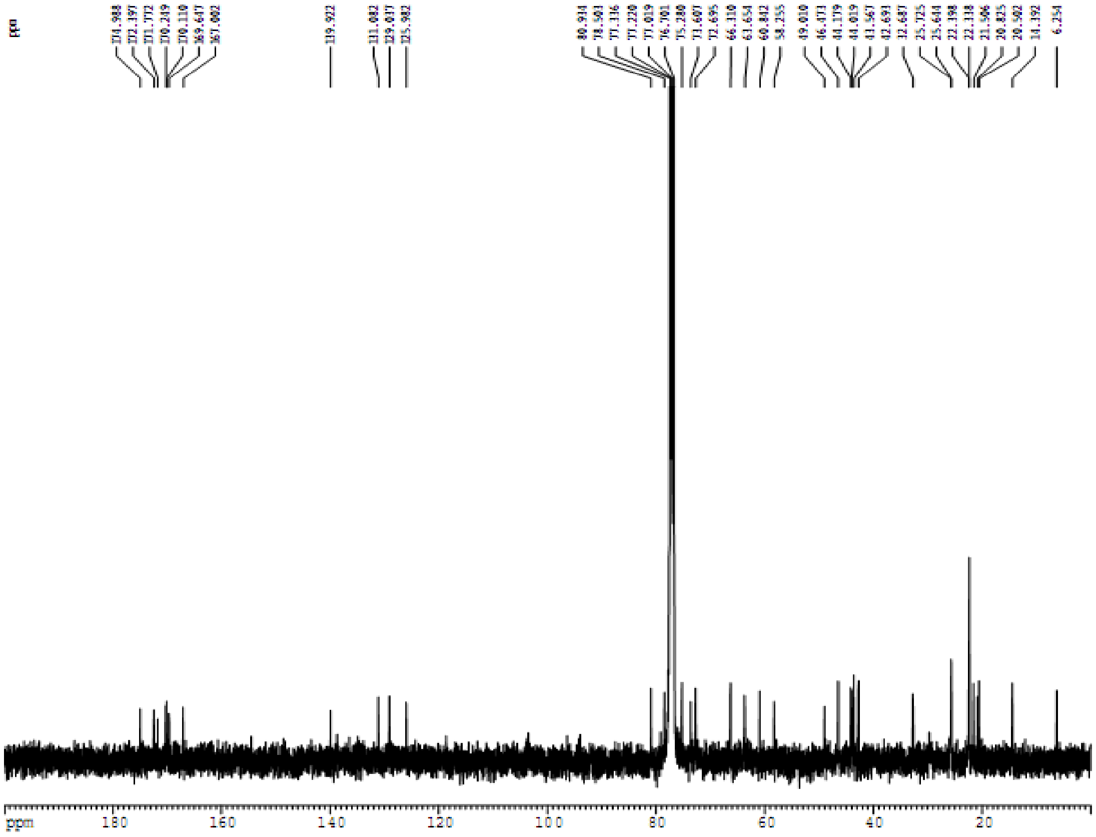

Figure S76. DEPT spectrum of the new compound **10**.

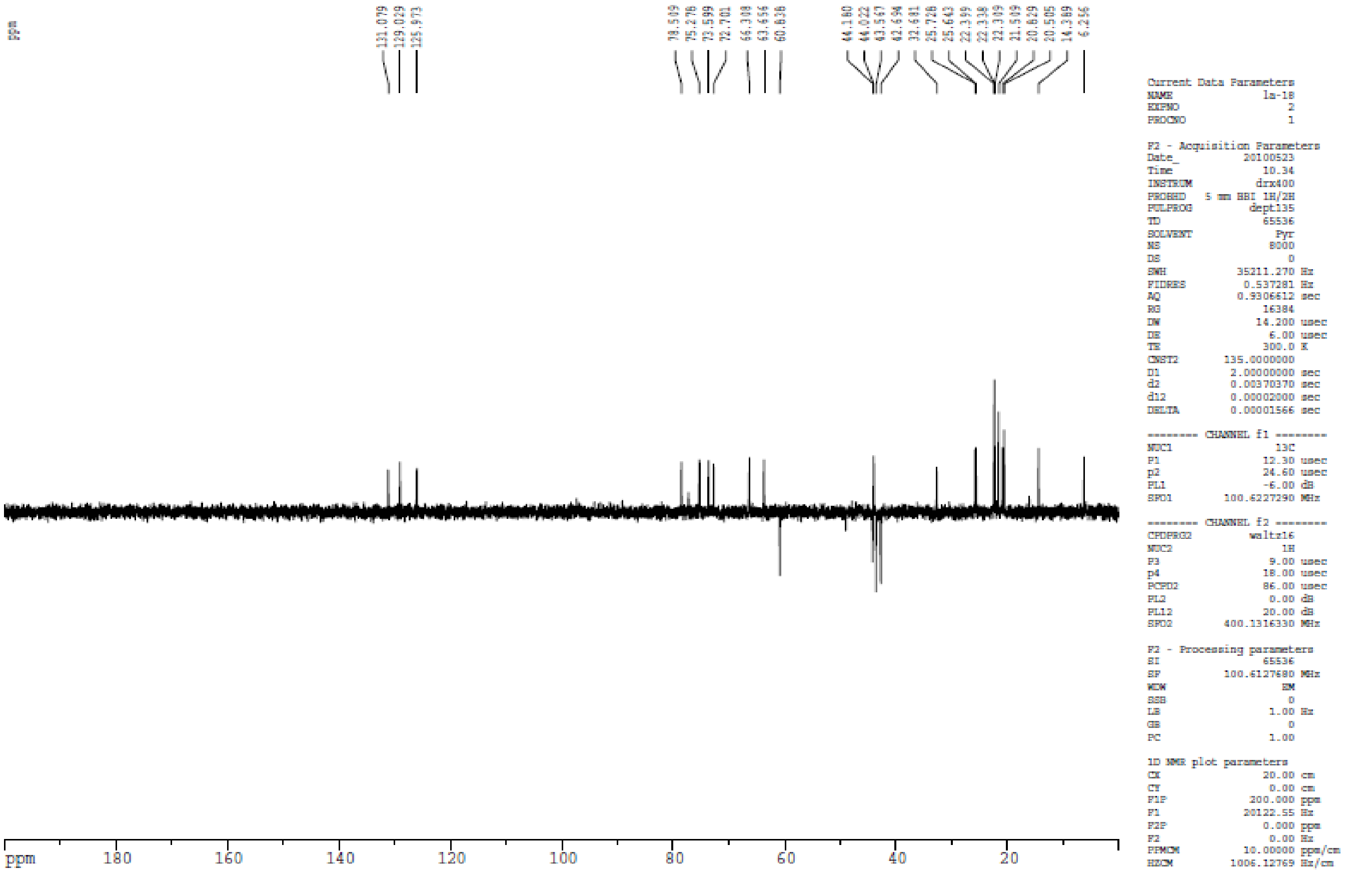

Figure S77. HSQC spectrum of the new compound **10**.

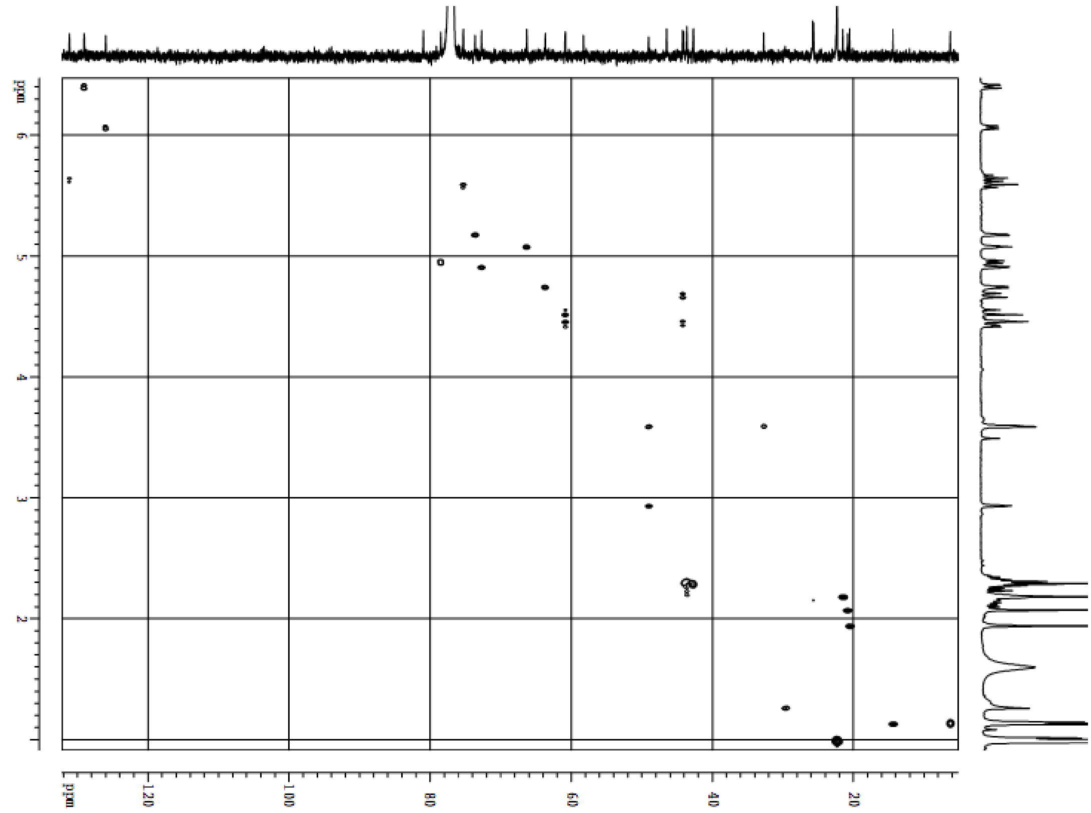

Figure S78. ^{1}H-^{1}H COSY spectrum of the new compound **10**.

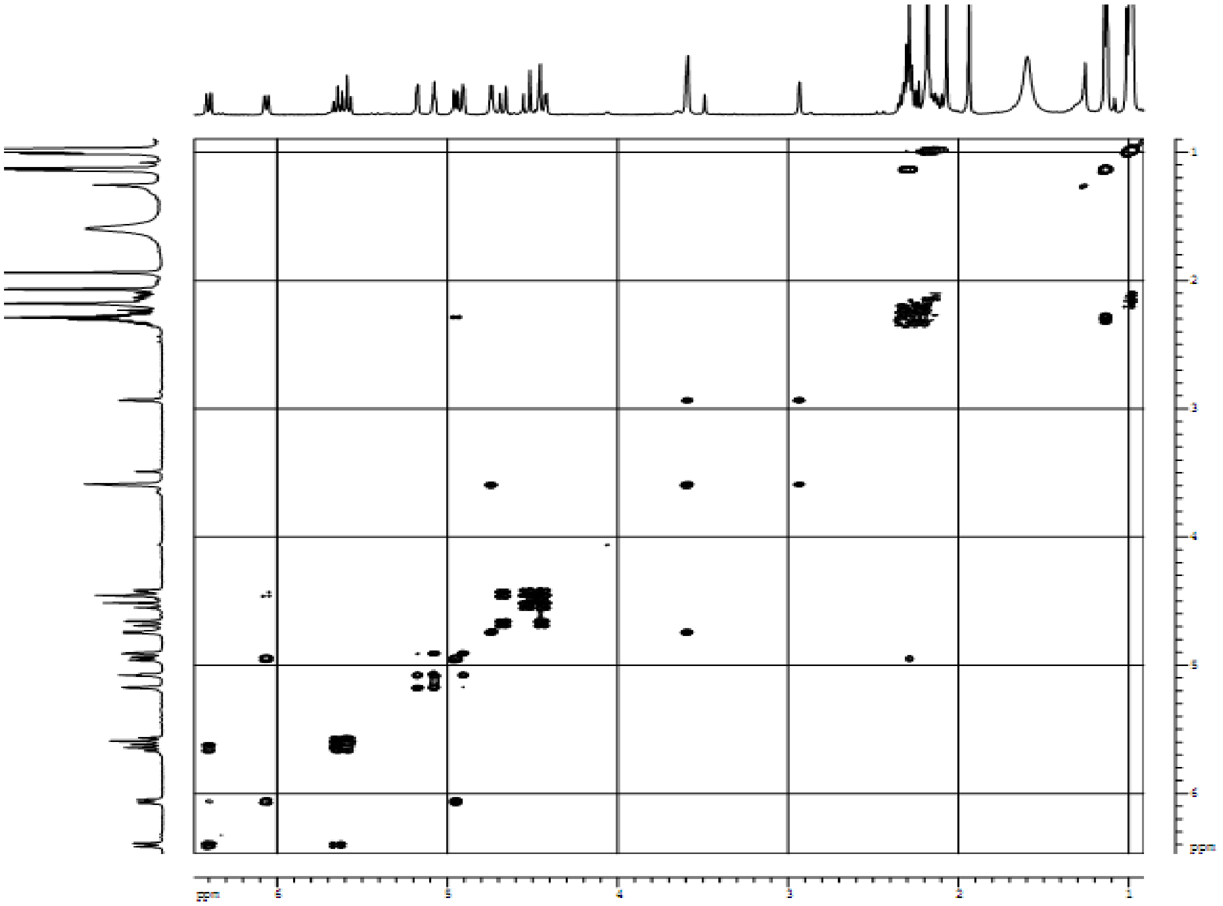

Figure S79. HMBC spectrum of the new compound **10**.

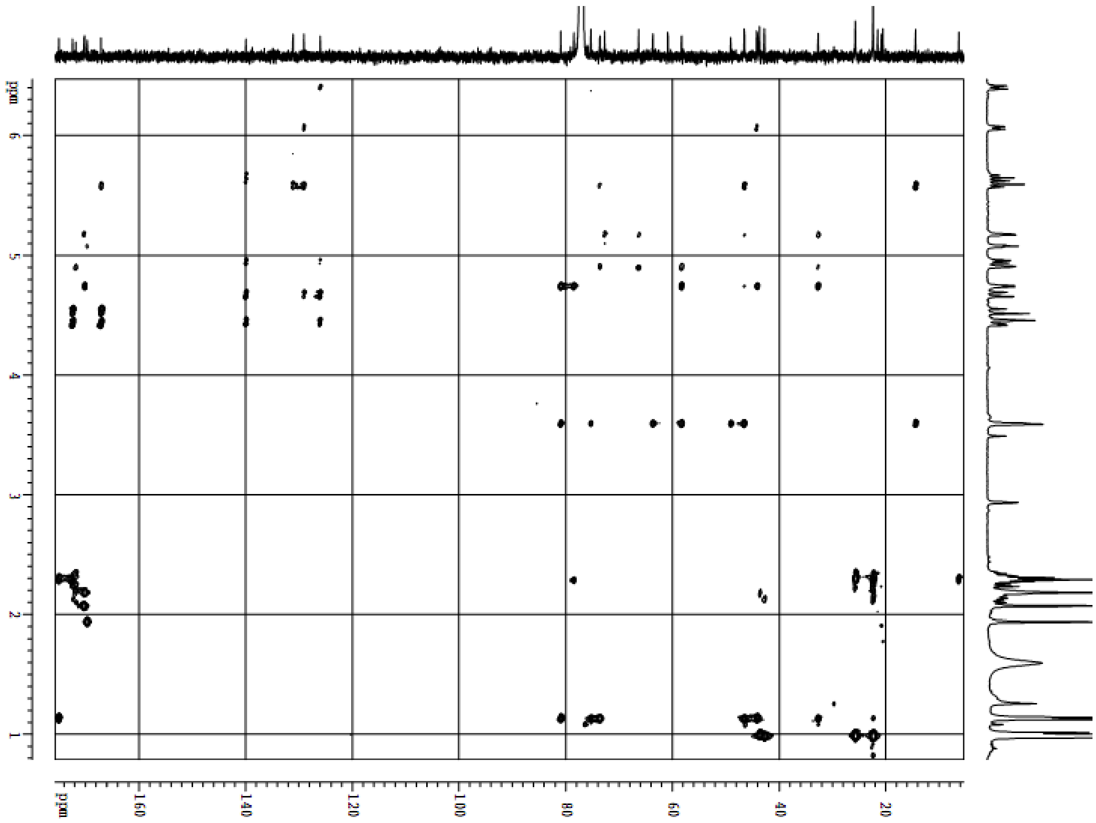

Figure S80. NOESY spectrum of the new compound **10**.

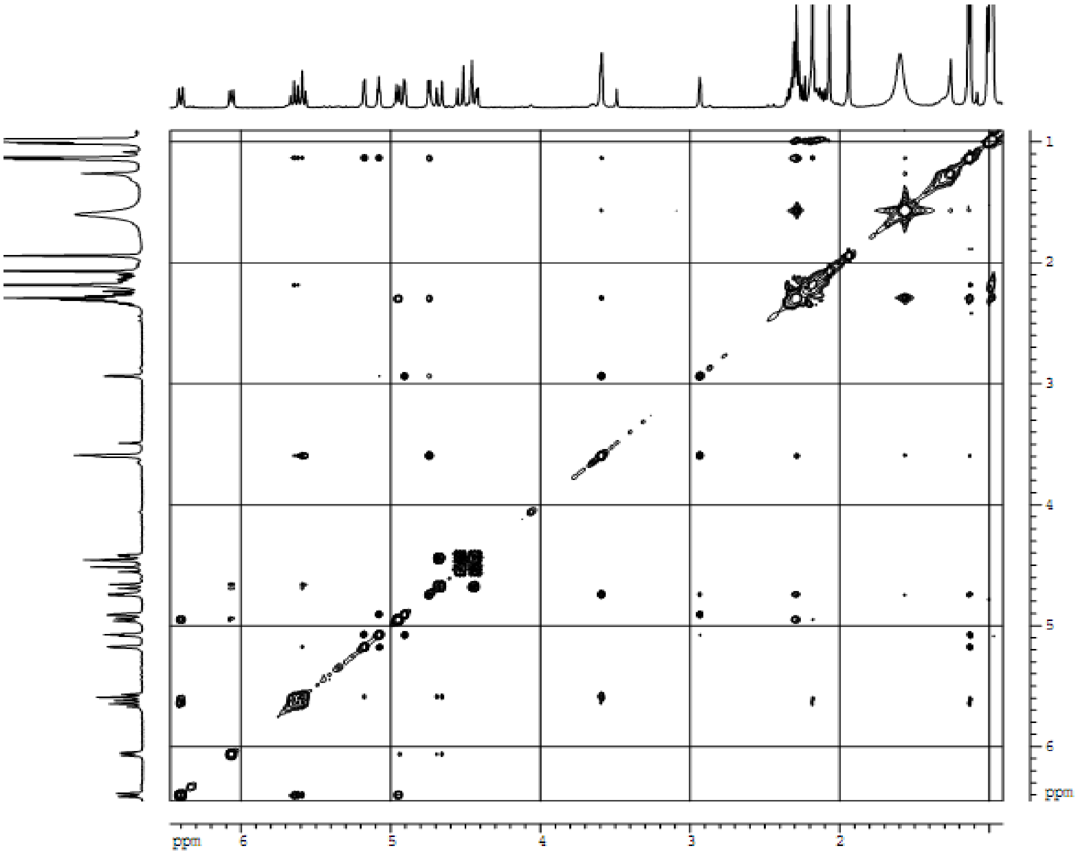

Figure S81. HRESIMS spectrum of compound **11**.

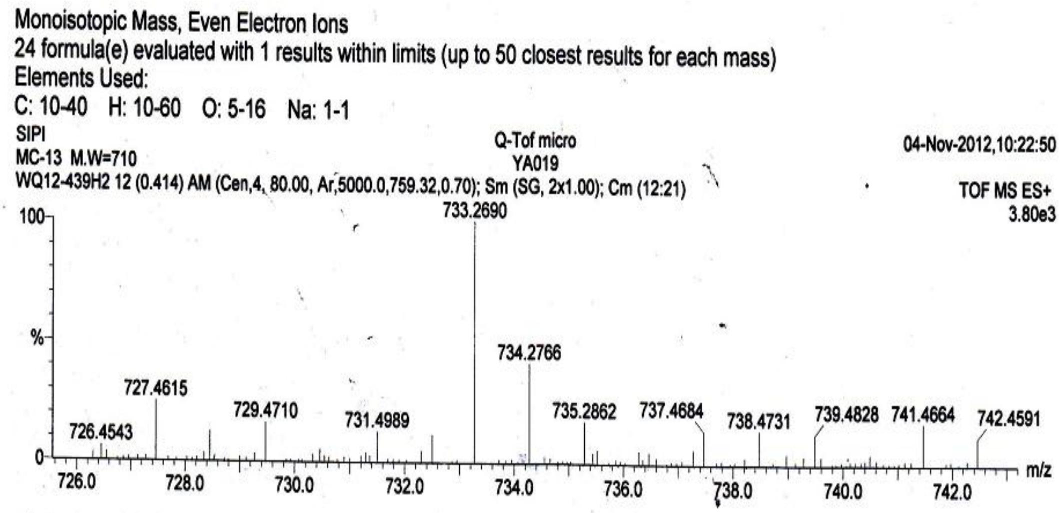

Minimum:	60.00				-1.5		
Maximum:	100.00		5.0	10.0	50.0		
Mass	RA	Calc. Mass	mDa	PPM	DBE	i-FIT	Formula
733.2690	100.00	733.2684	0.6	0.8	11.5	47.1	C34 H46 O16 Na

Figure S82. ^{1}H NMR spectrum of compound **11** in $CDCl_3$.

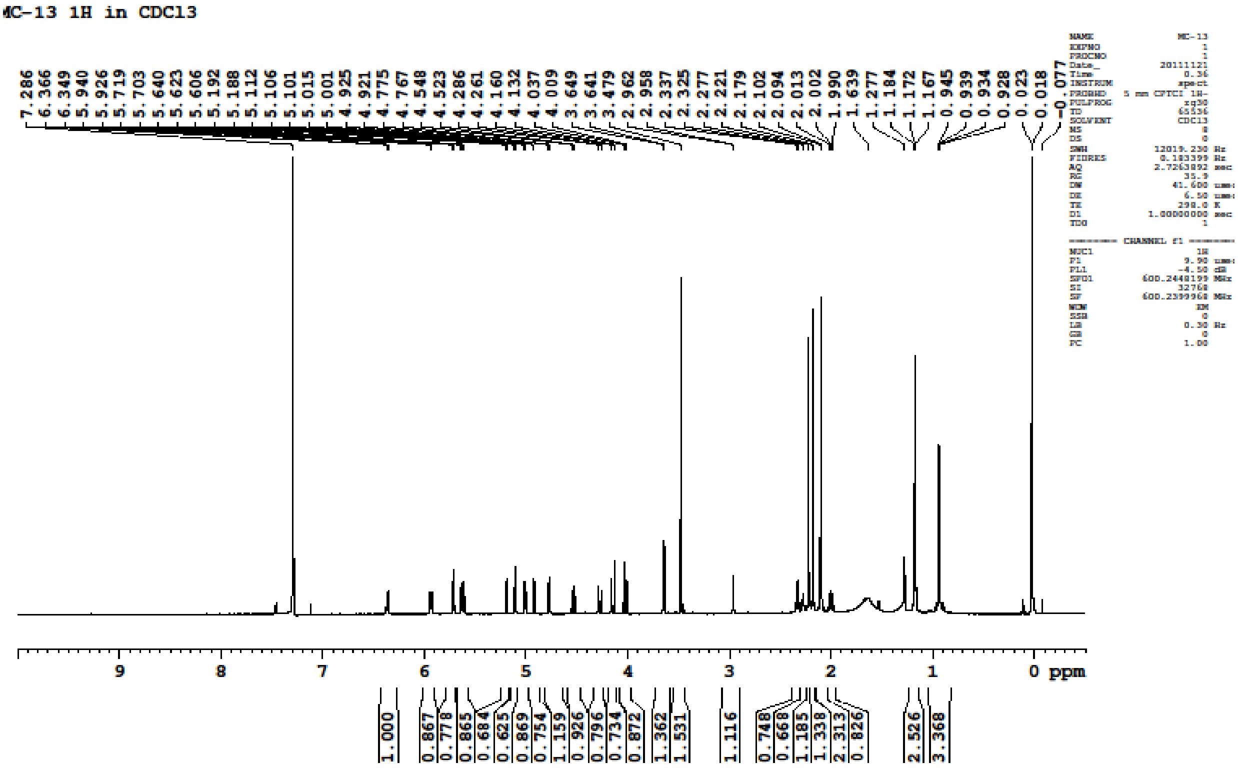

Figure S83. ^{13}C NMR spectrum of compound **11** in $CDCl_3$.

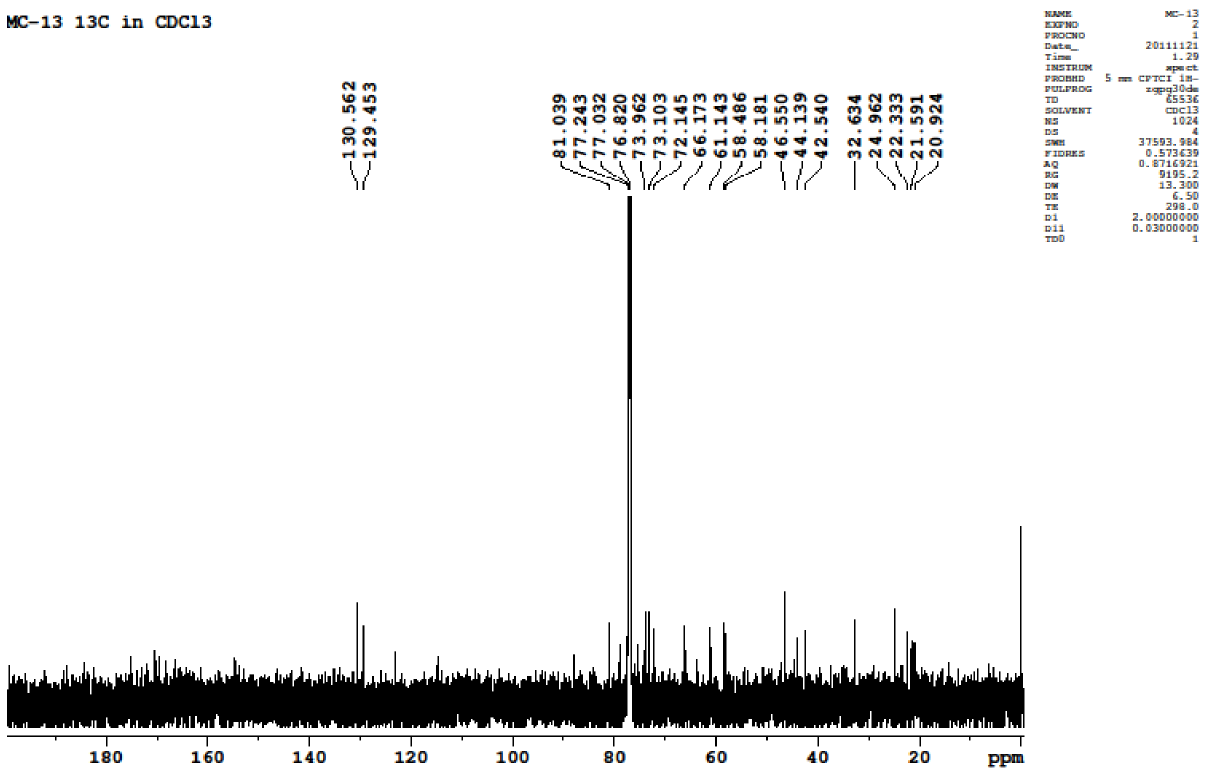

Figure S84. DEPT spectrum of compound **11** in $CDCl_3$.

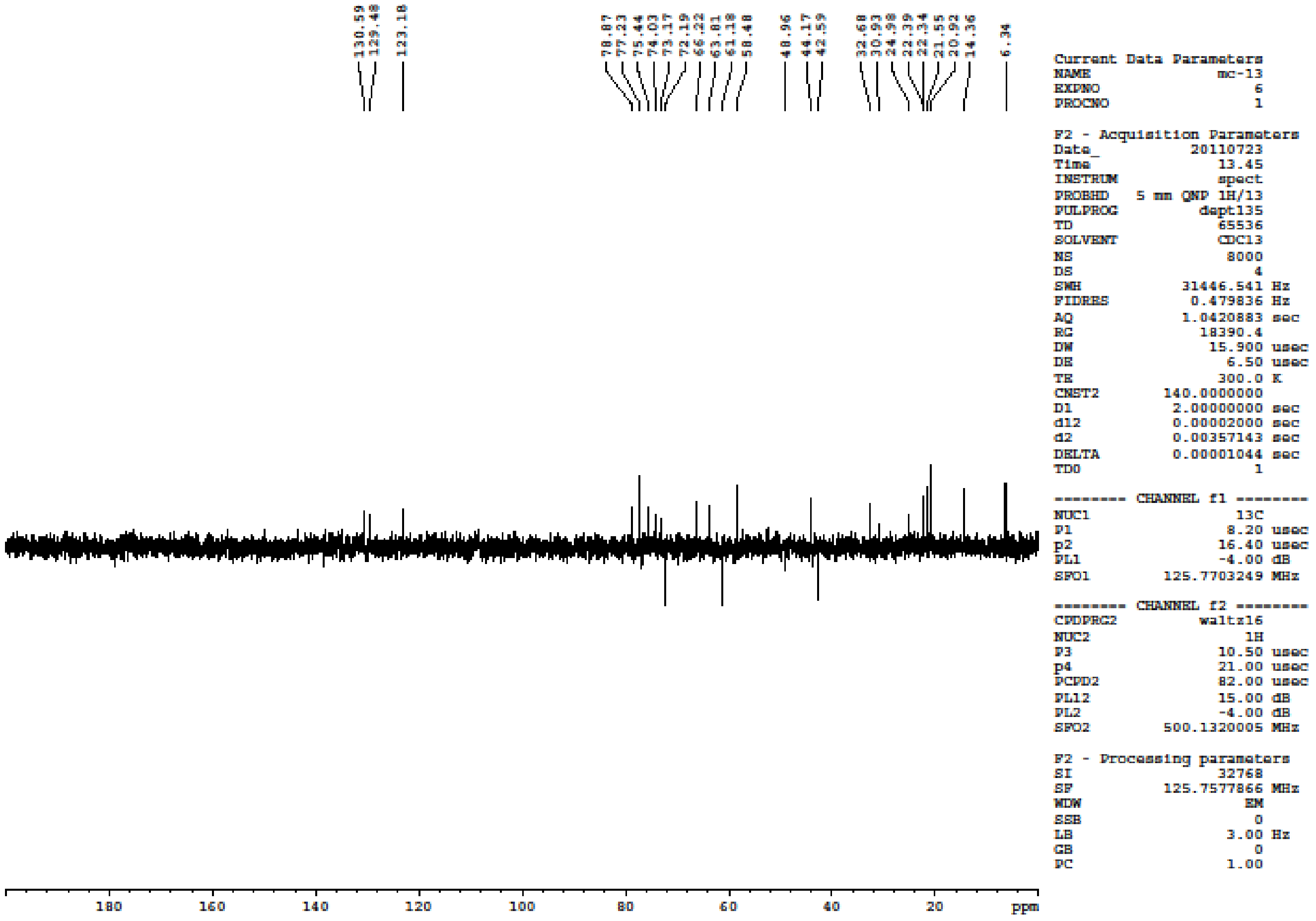

Figure S85. HSQC spectrum of compound **11** in $CDCl_3$.

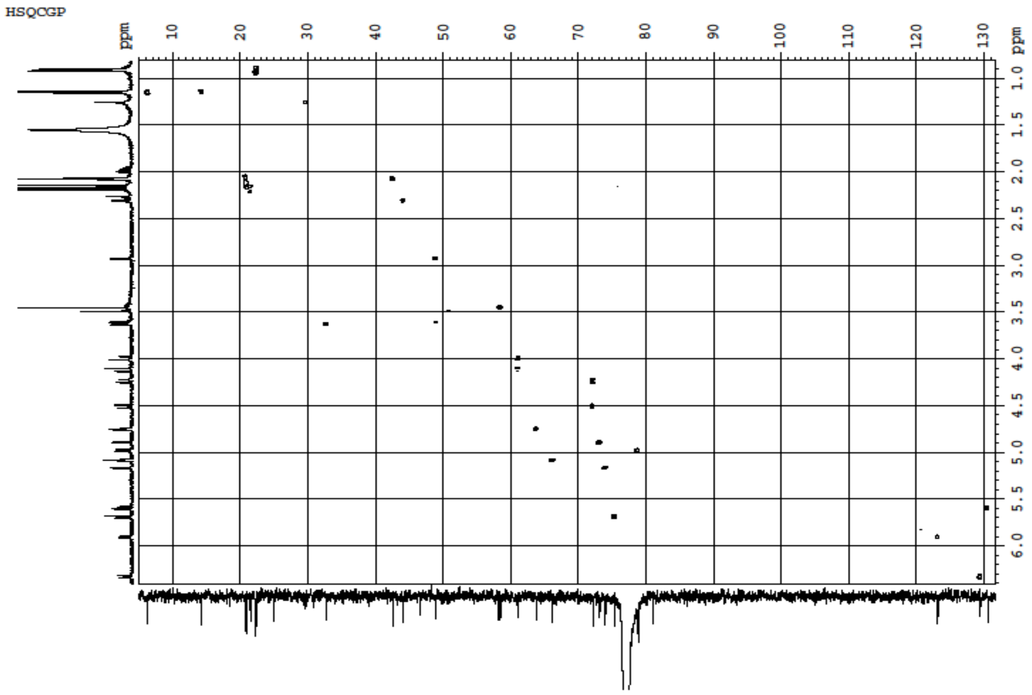

Figure S86. ^{1}H-^{1}H COSY spectrum of compound **11** in $CDCl_3$.

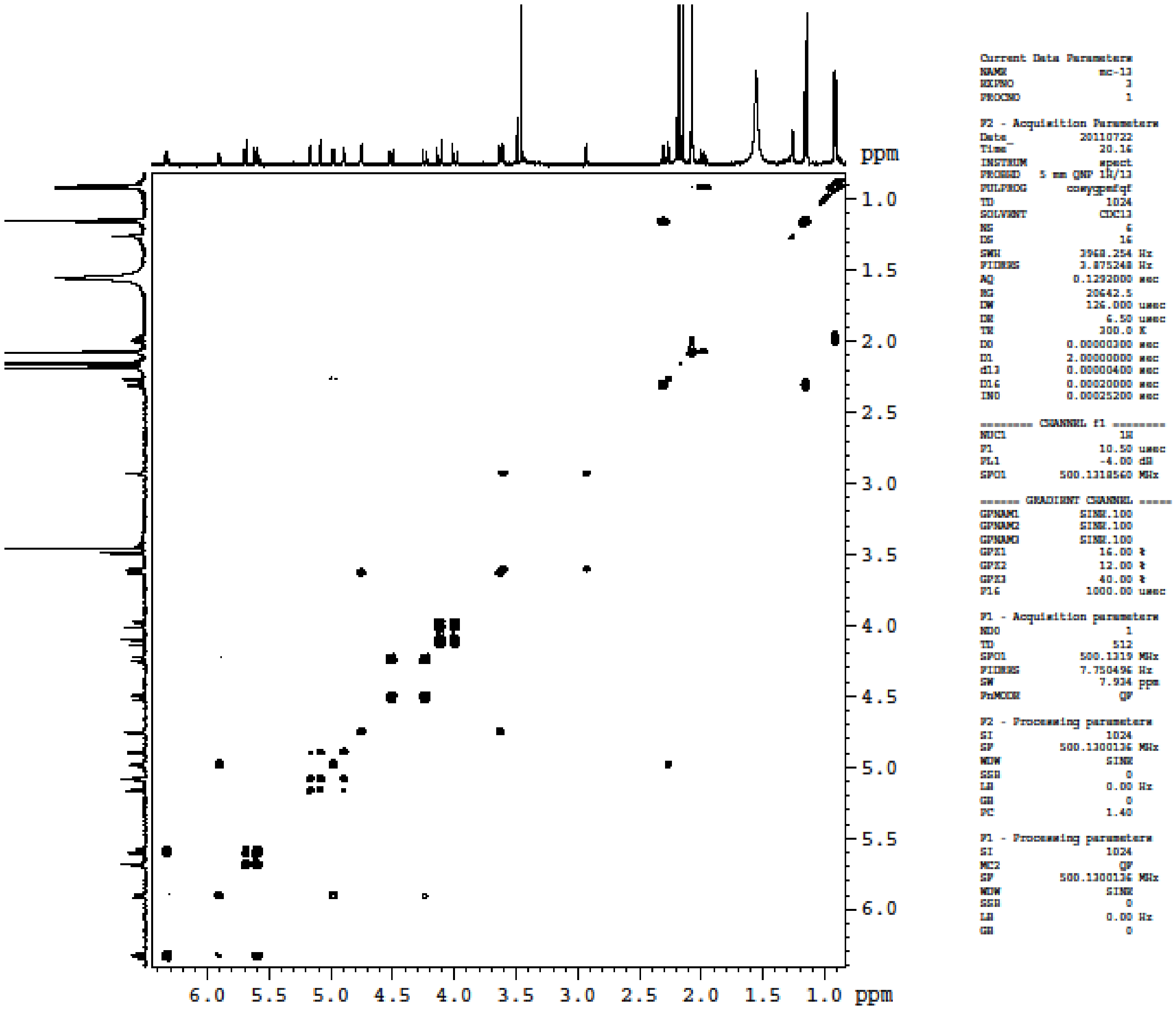

Figure S87. HMBC spectrum of compound **11** in $CDCl_3$.

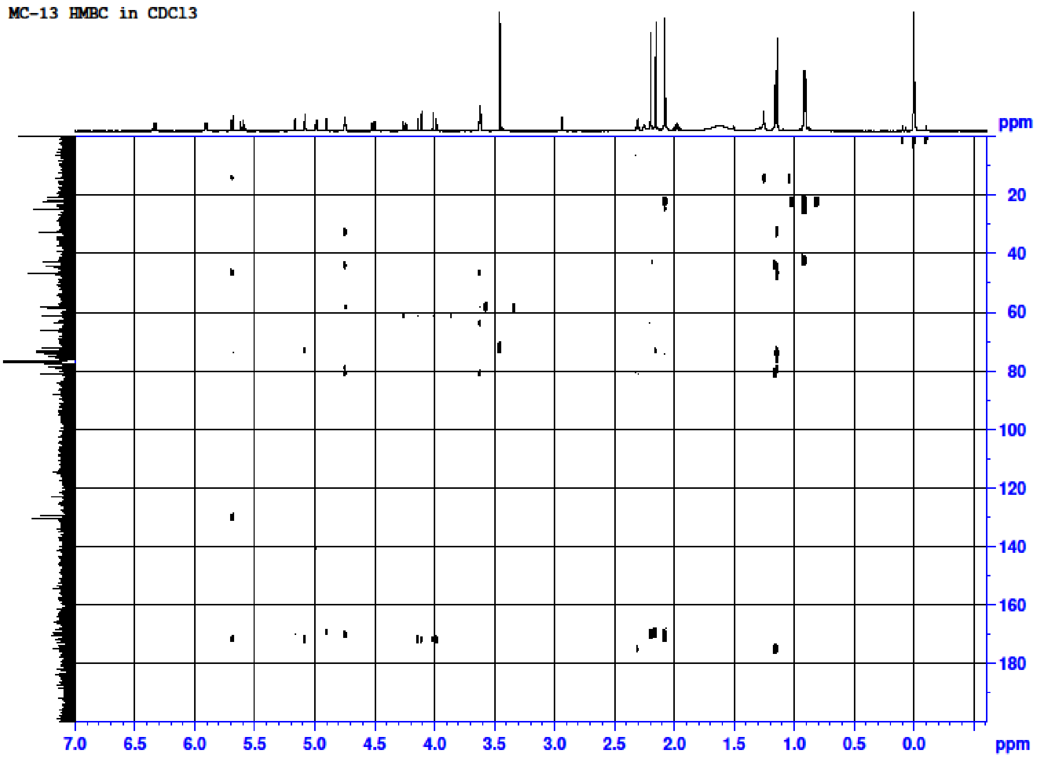

Figure S88. NOESY spectrum of compound **11** in $CDCl_3$.

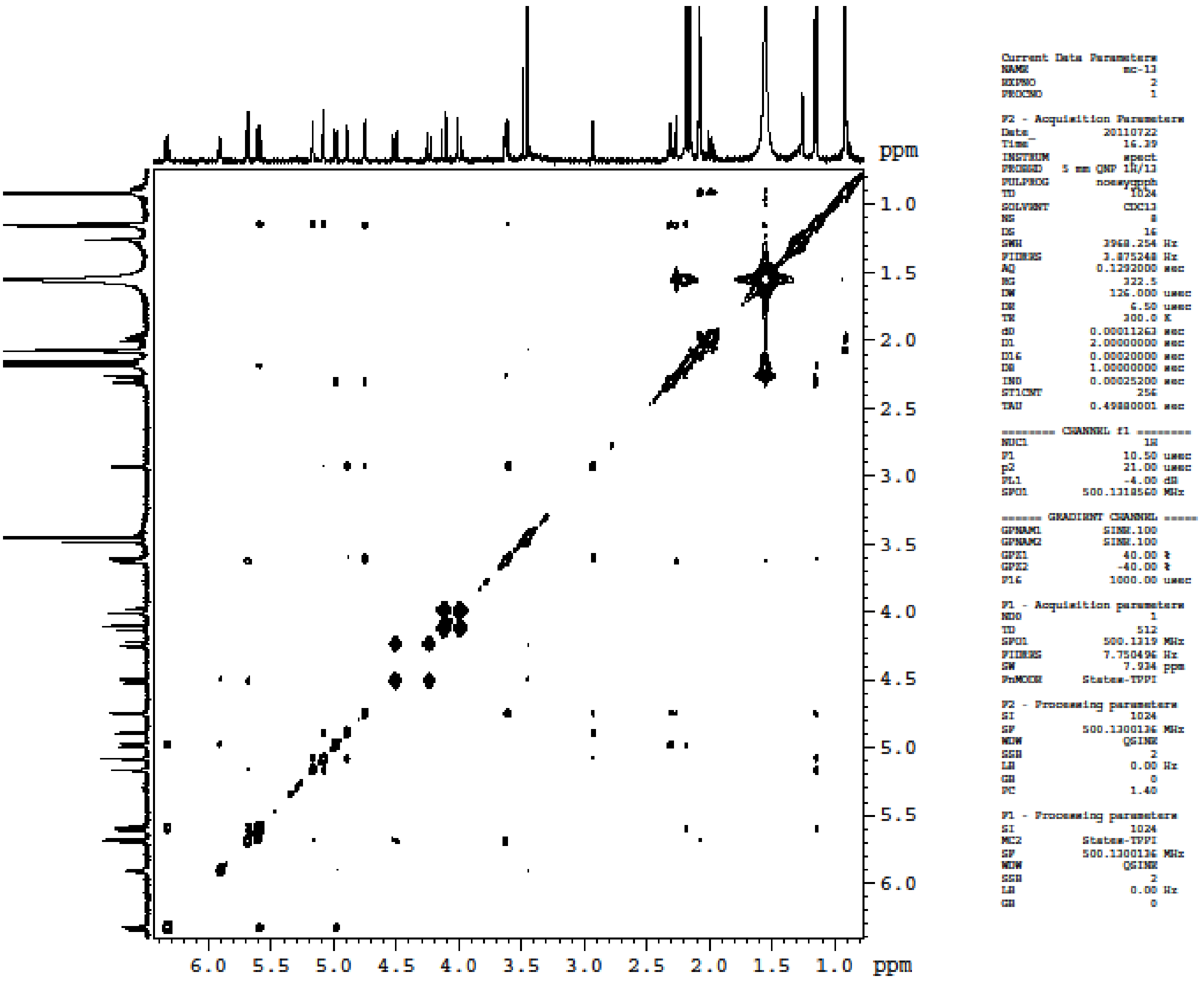

Figure S89. HR-ESIMS spectrum of the new compound **12**.

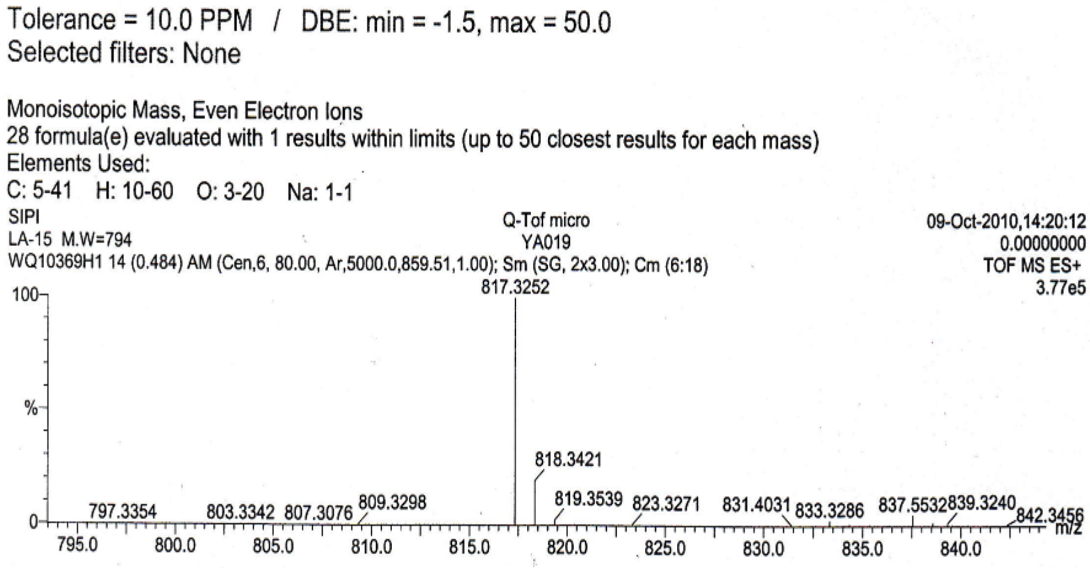

Minimum:	60.00				-1.5		
Maximum:	100.00		5.0	10.0	50.0		
Mass	RA	Calc. Mass	mDa	PPM	DBE	i-FIT	Formula
817.3252	100.00	817.3259	-0.7	-0.9	12.5	68929.6	C39 H54 O17 Na

Figure S90. ^{1}H MNR (400 MHz, $CDCl_3$) spectrum of the new compound **12**.

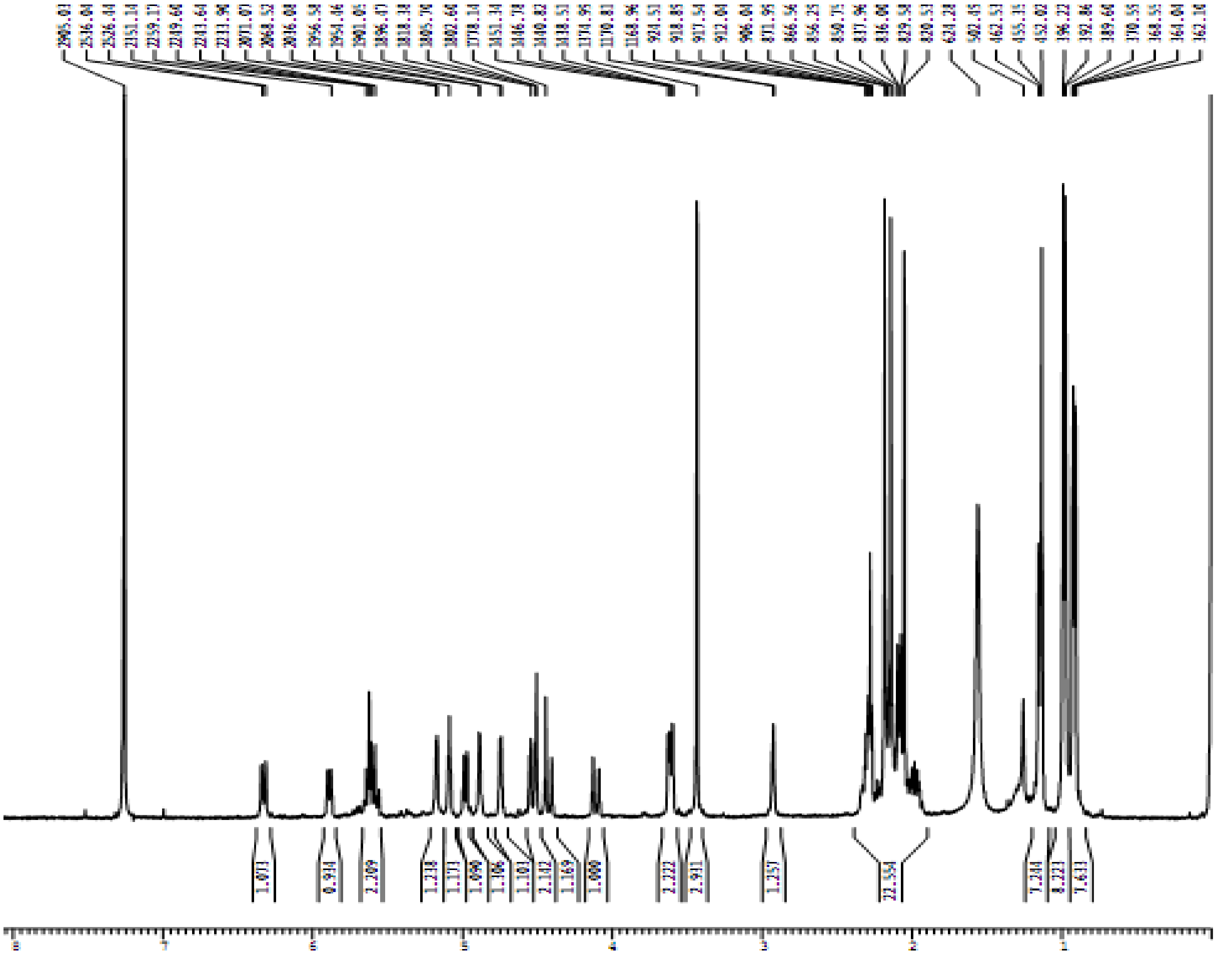

Figure S91. ^{13}C MNR (100 MHz, $CDCl_3$) spectrum of the new compound **12**.

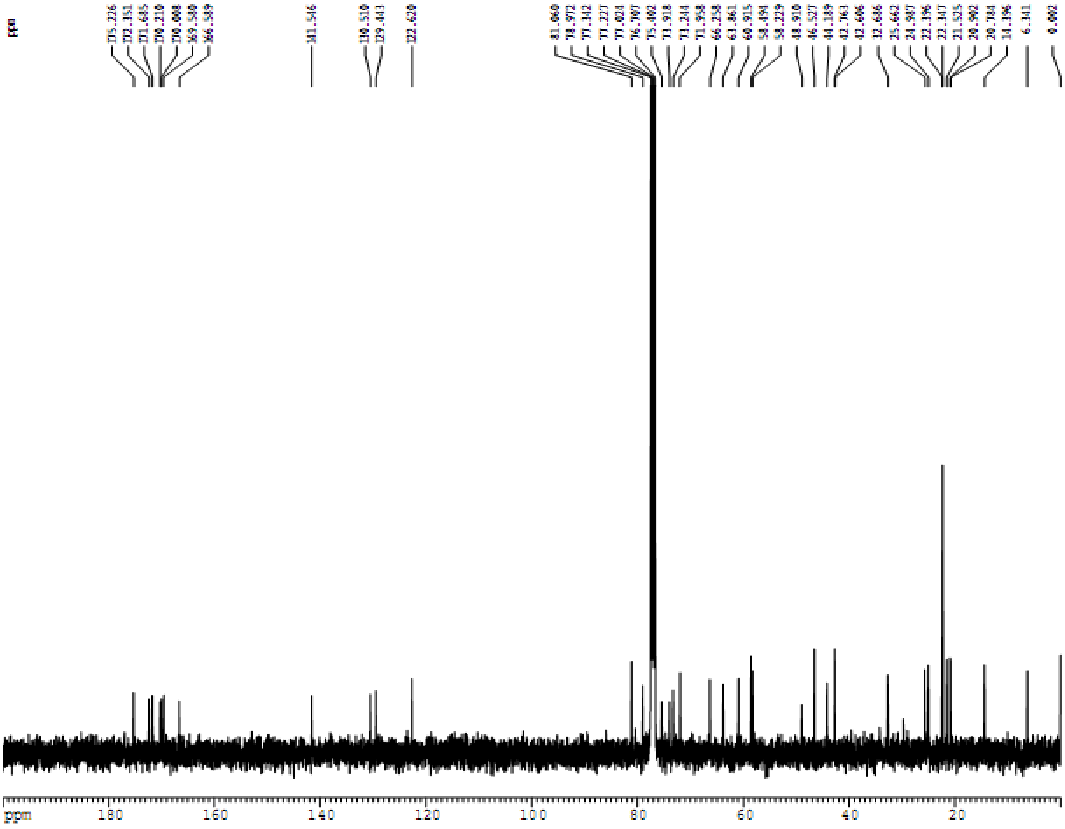

Figure S92. DEPT spectrum of the new compound **12**.

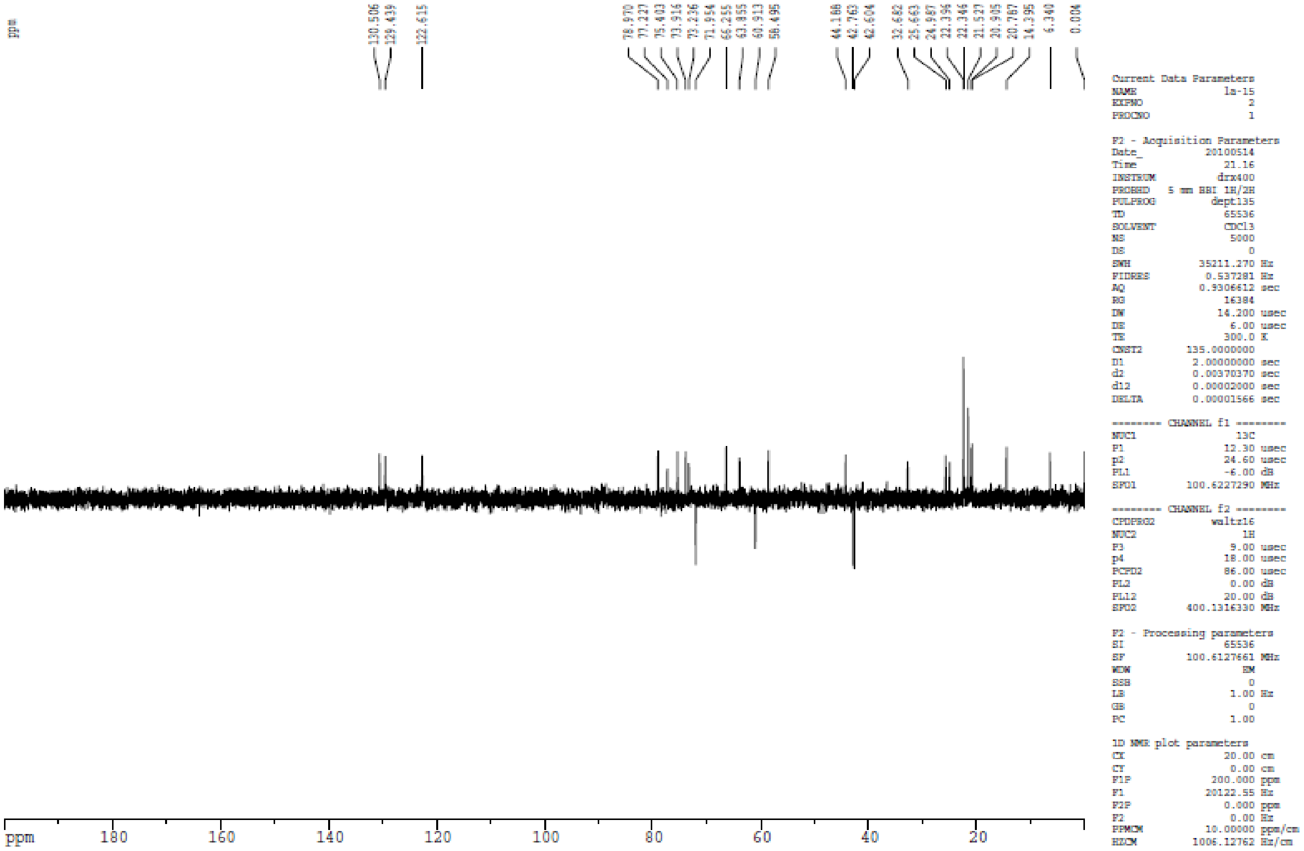

Figure S93. HSQC spectrum of the new compound **12**.

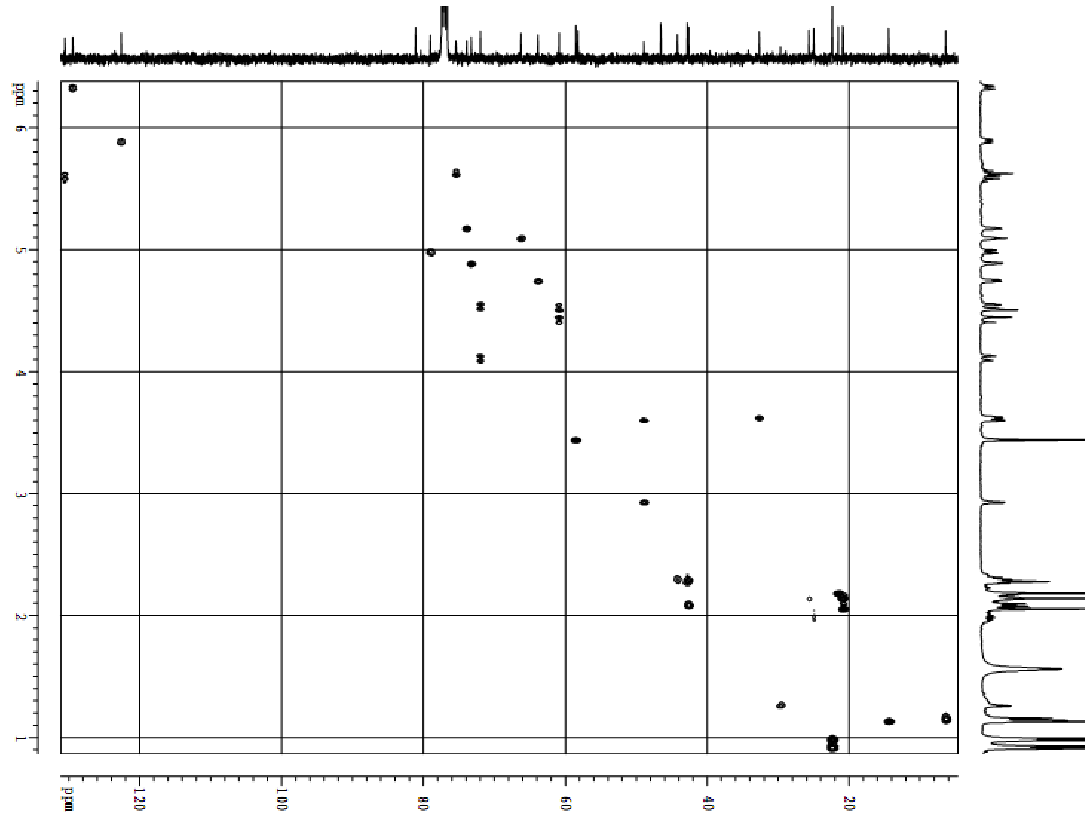

Figure S94. ^1H-^1H COSY spectrum of the new compound **12**.

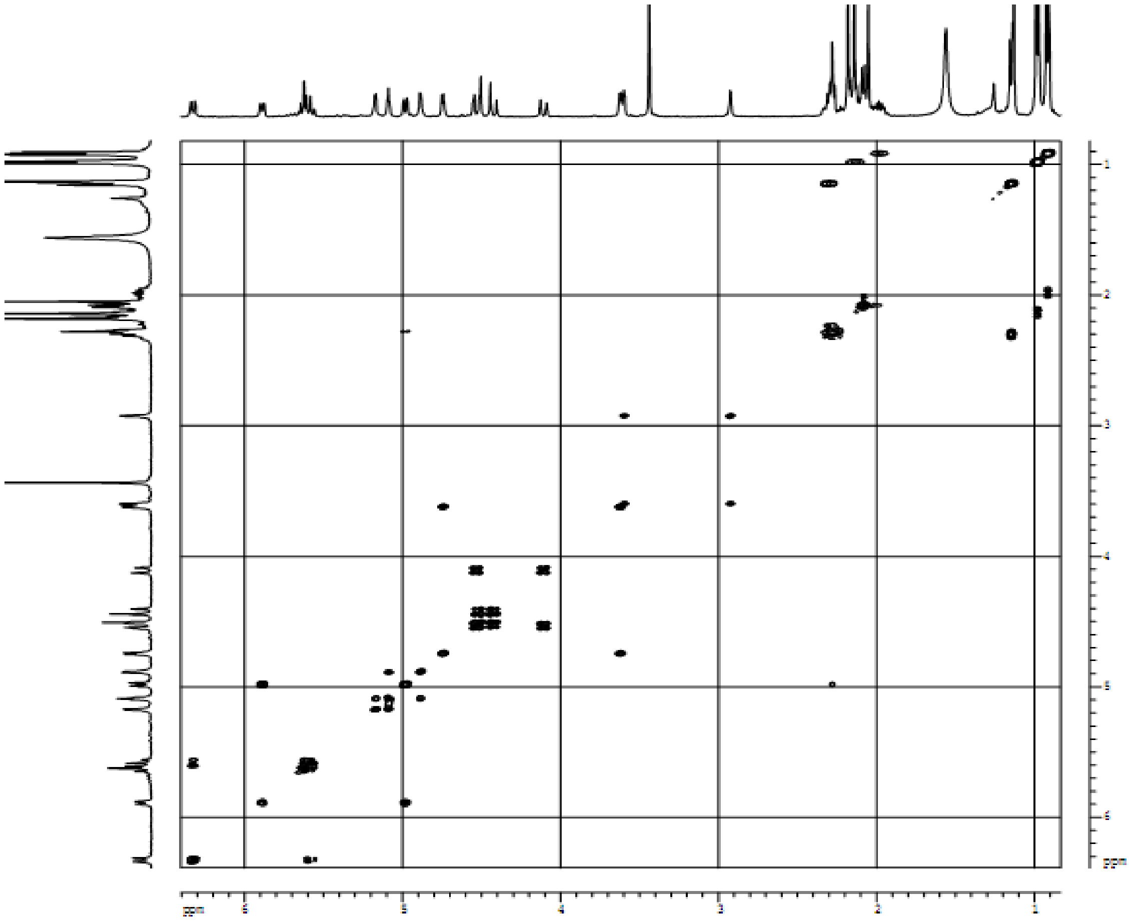

Figure S95. HMBC spectrum of the new compound **12**.

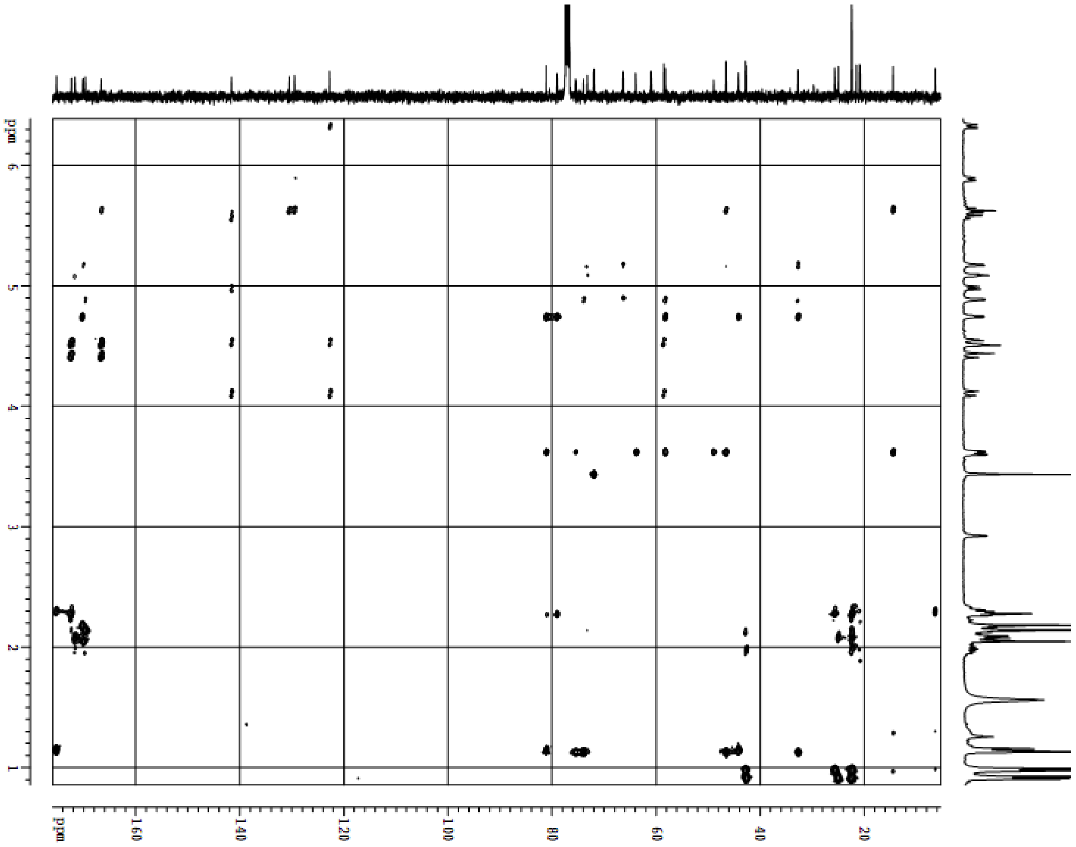

Figure S96. NOESY spectrum of the new compound **12**.

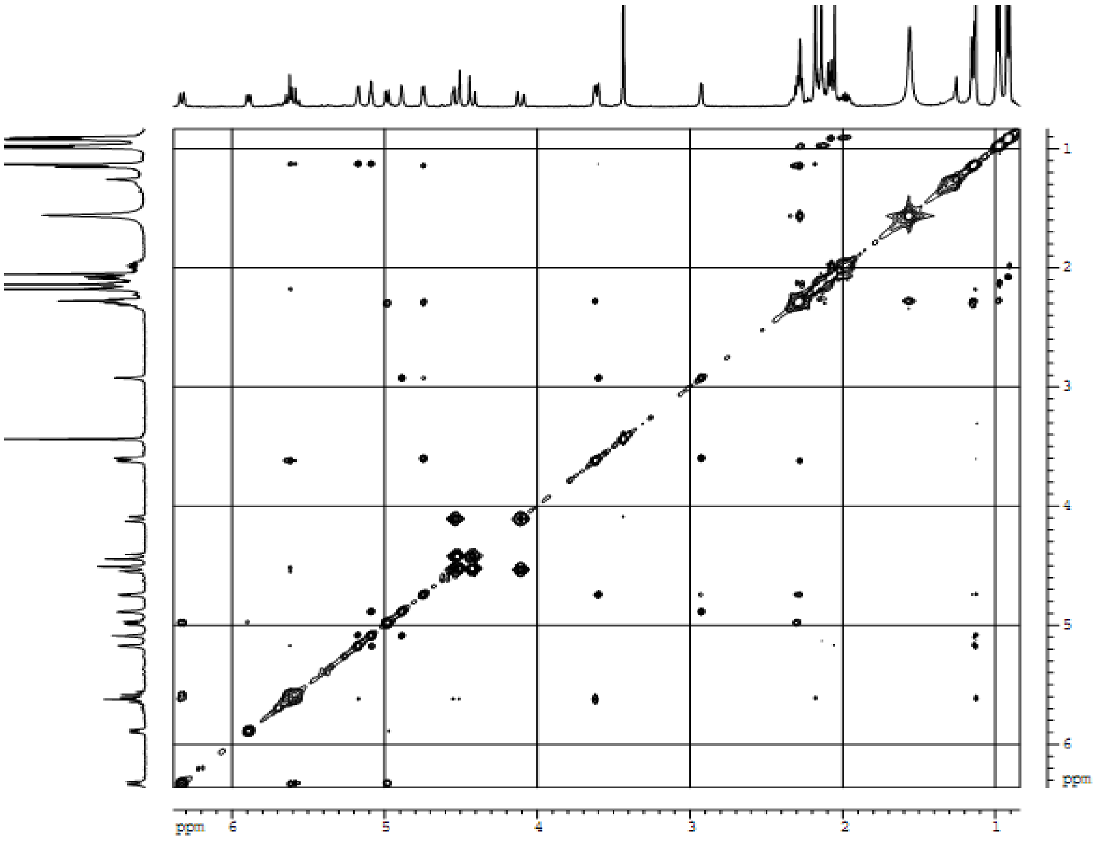

Figure S97. HRESI spectrum of compound **13**.

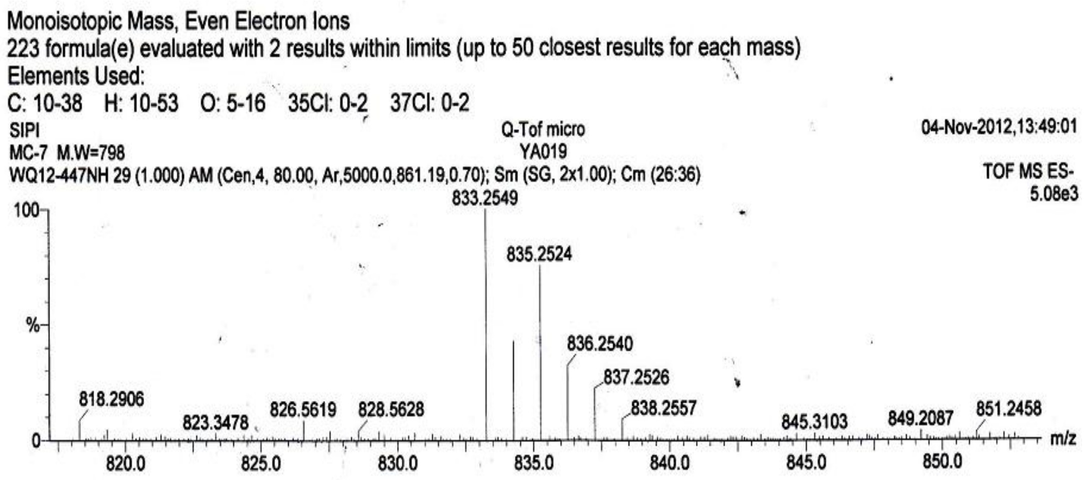

Minimum:	75.00				-1.5		
Maximum:	100.00		5.0	10.0	50.0		

Mass	RA	Calc. Mass	mDa	PPM	DBE	i-FIT	Formula
833.2549	100.00	833.2554	-0.5	-0.6	12.5	0.4	C38 H51 O16 35Cl2
835.2524	75.58	835.2525	-0.1	-0.1	12.5	14.5	C38 H51 O16 35Cl 37Cl

Figure S98. ^{1}H NMR spectrum of compound **13** in $CDCl_3$.

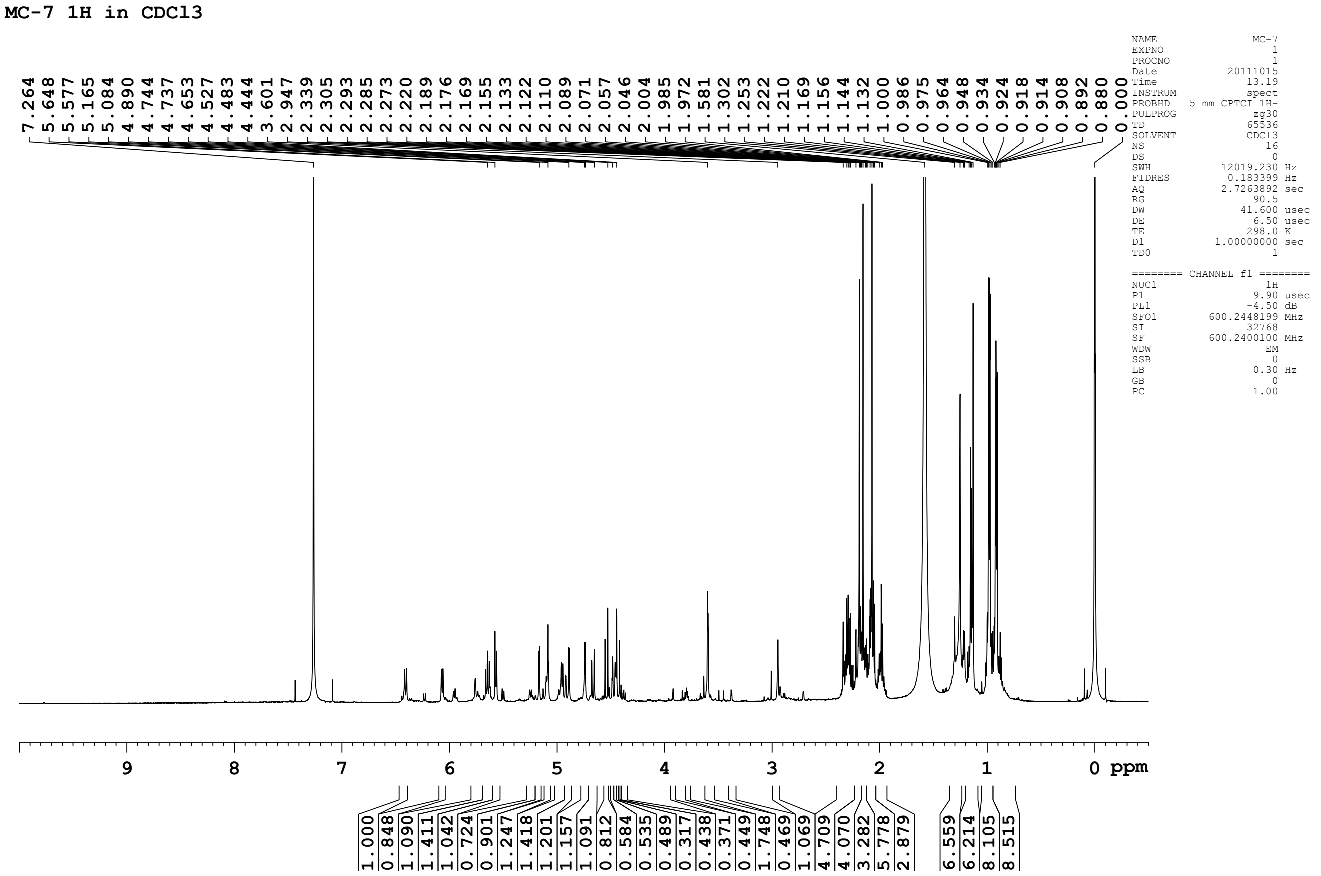

Figure S99. ^{13}C NMR spectrum of compound **13** in $CDCl_3$.

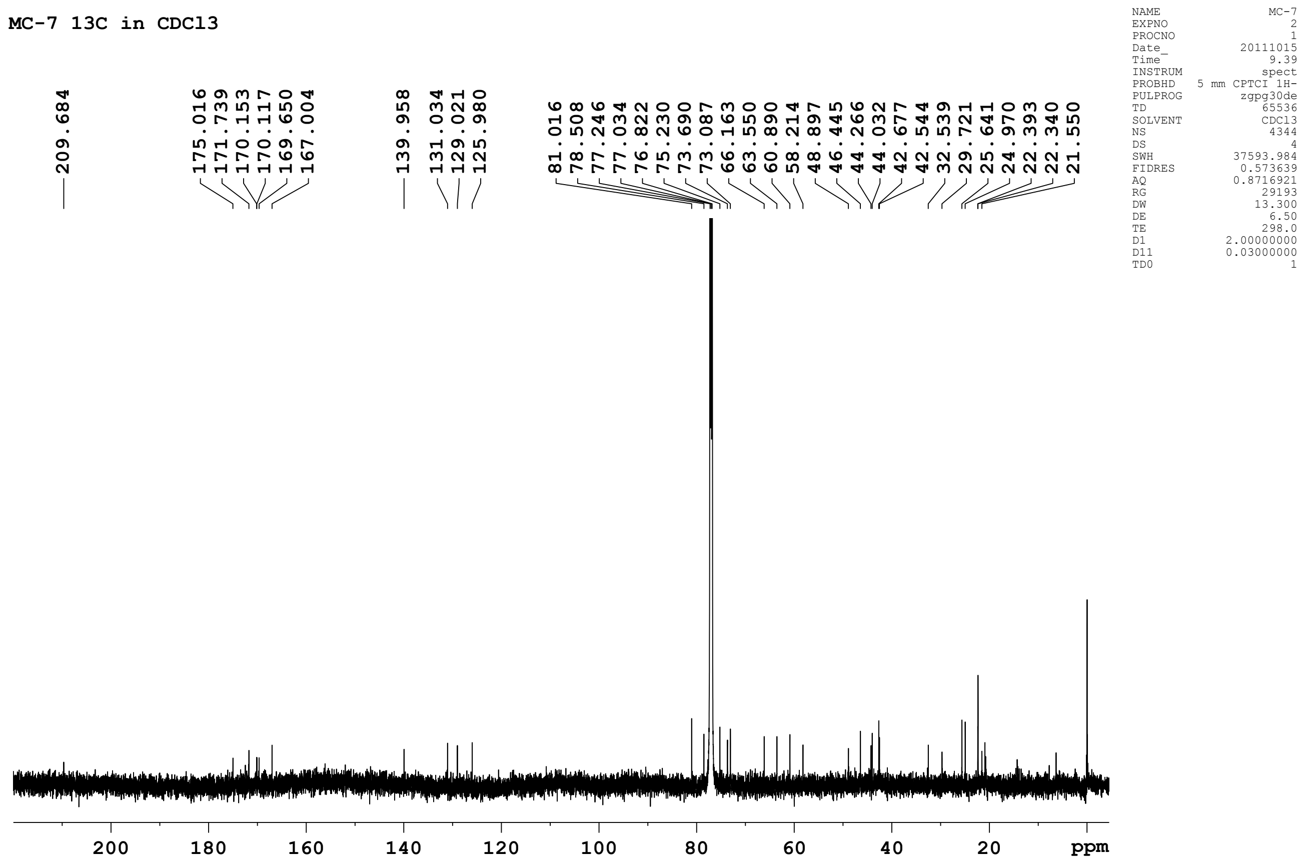

Figure S100. DEPT spectrum of compound **13** in $CDCl_3$.

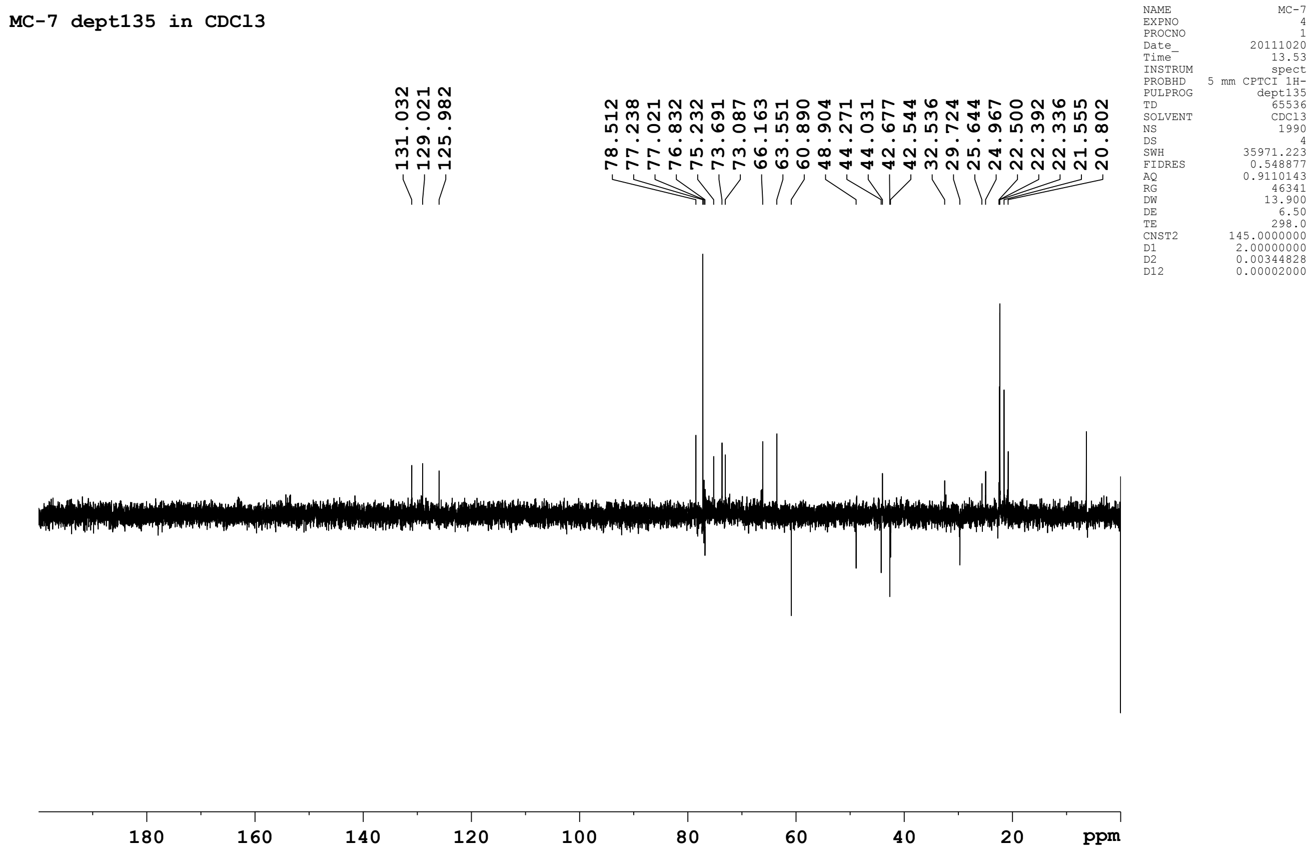

Figure S101. HSQC spectrum of compound **13** in $CDCl_3$.

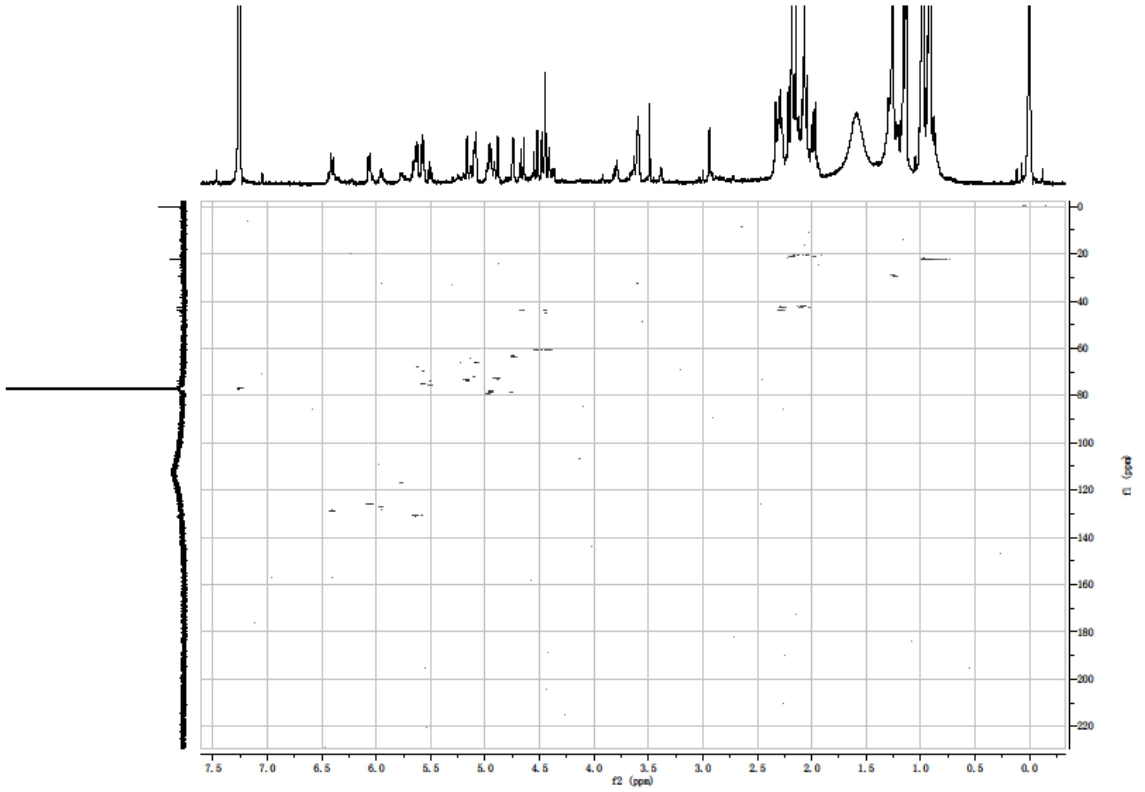

Figure S102. ^{1}H-^{1}H COSY spectrum of compound **13** in $CDCl_3$.

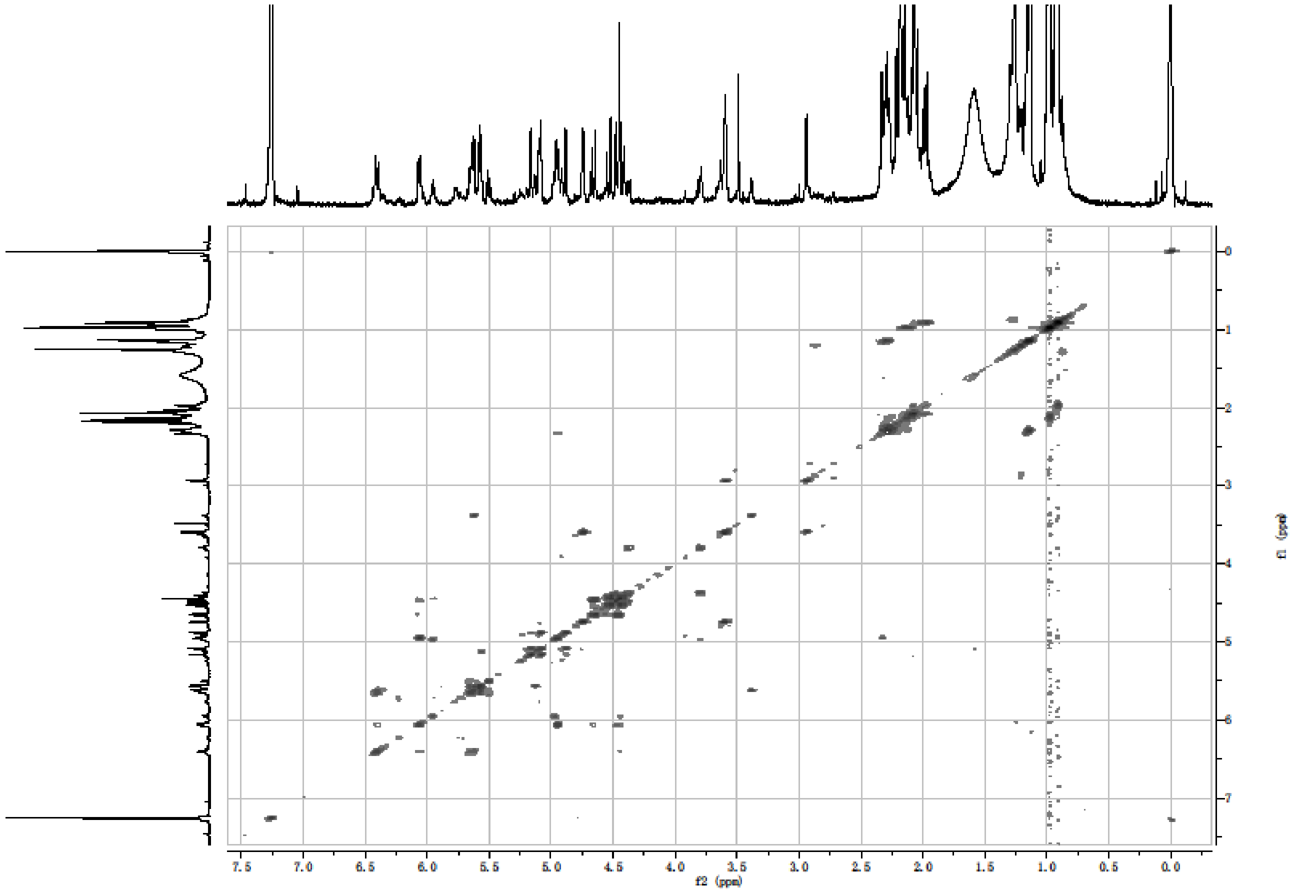

Figure S103. HMBC spectrum of compound **13** in $CDCl_3$.

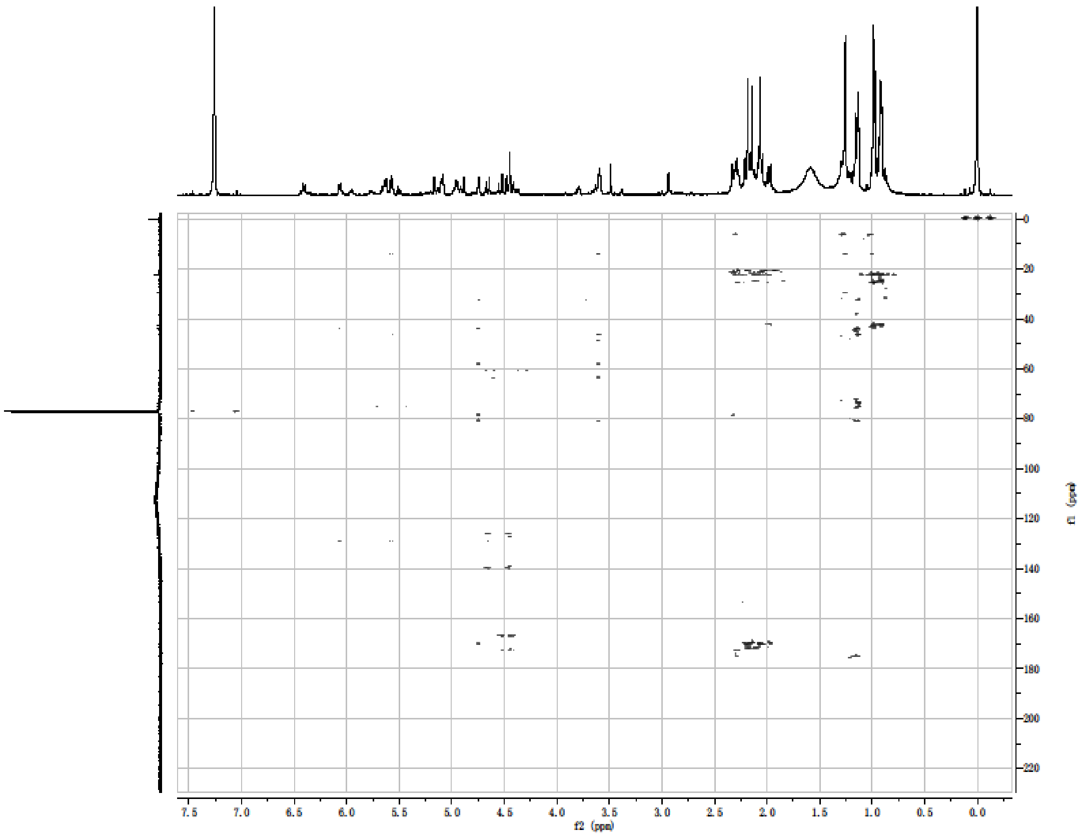

Figure S104. NOESY spectrum of compound **13** in $CDCl_3$.

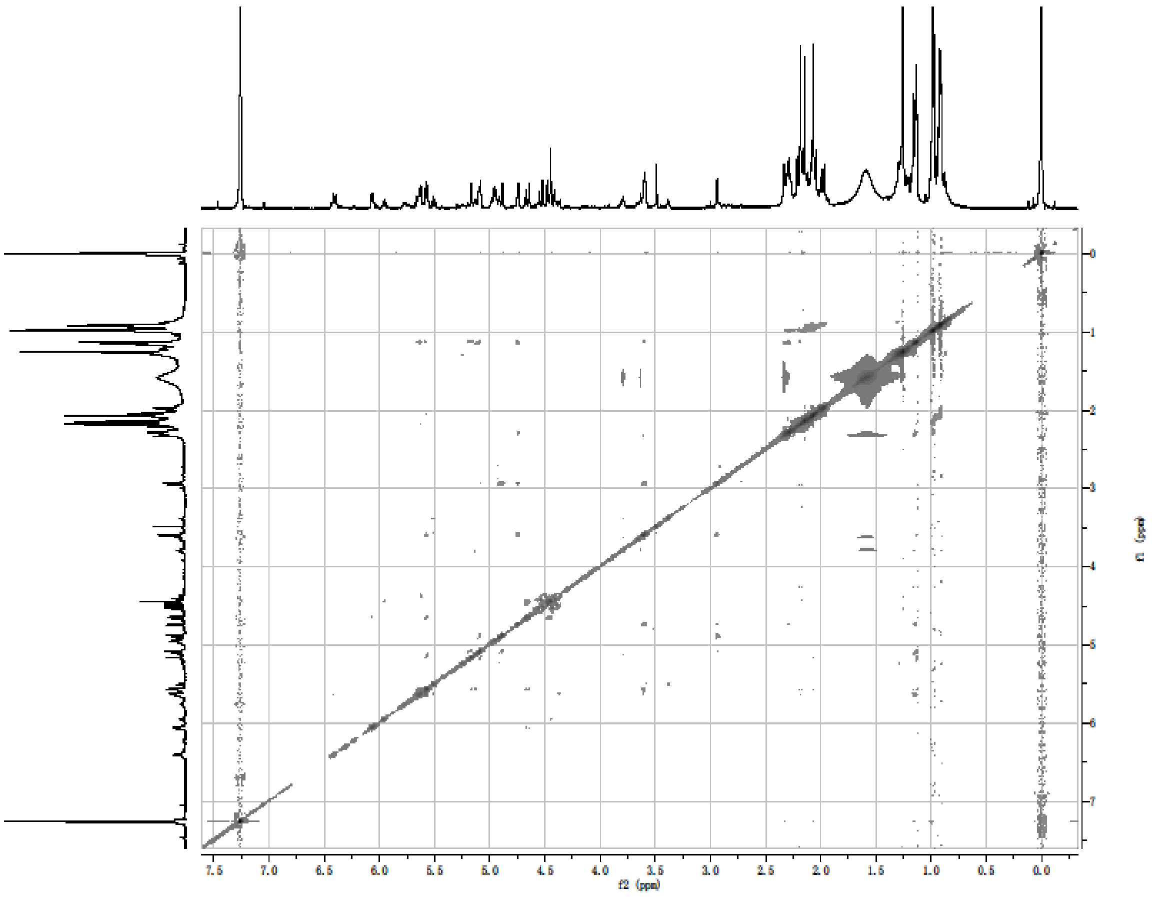

Figure S105. HRESI spectrum of compound **14**.

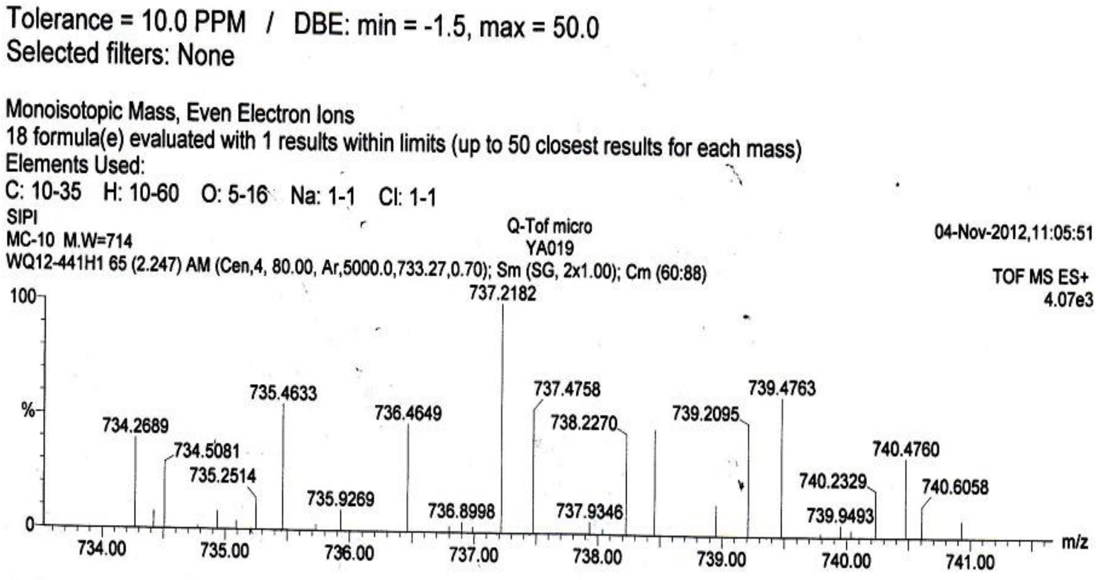

Minimum:	65.00				-1.5		
Maximum:	100.00		5.0	10.0	50.0		
Mass	RA	Calc. Mass	mDa	PPM	DBE	i-FIT	Formula
737.2182	100.00	737.2188	-0.6	-0.8	11.5	22.3	C33 H43 O15 Na Cl

Figure S106. ^{1}H NMR spectrum of compound **14** in $CDCl_3$.

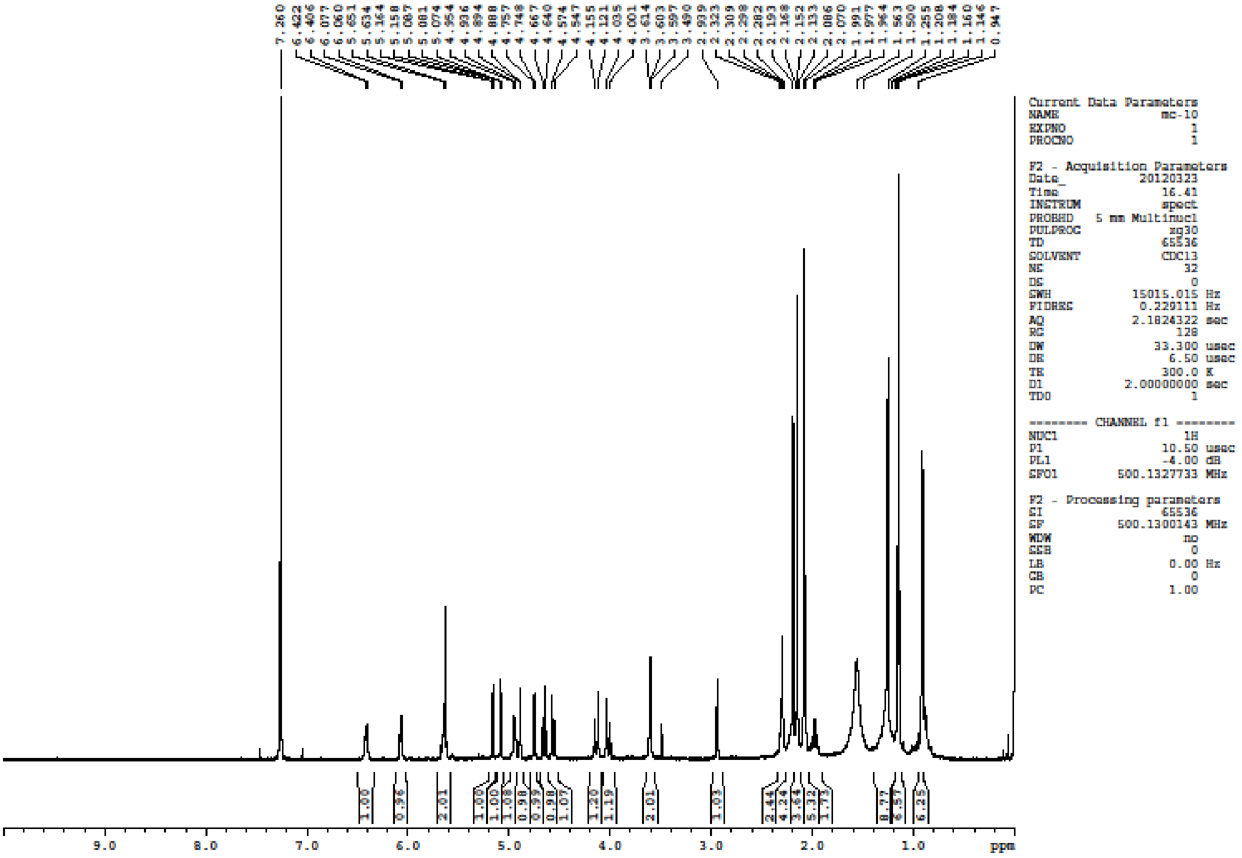

Figure S107. ^{13}C NMR spectrum of compound **14** in $CDCl_3$.

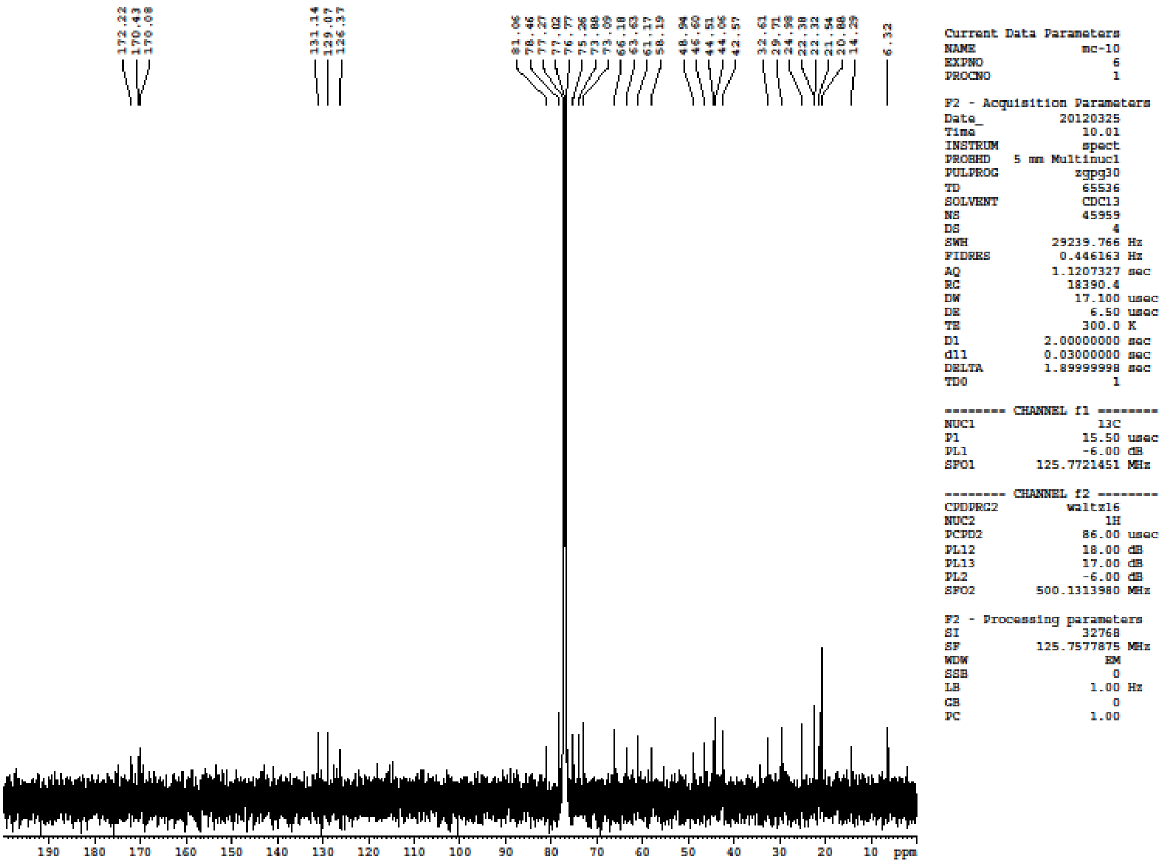

Figure S108. DEPT spectrum of compound **14** in $CDCl_3$.

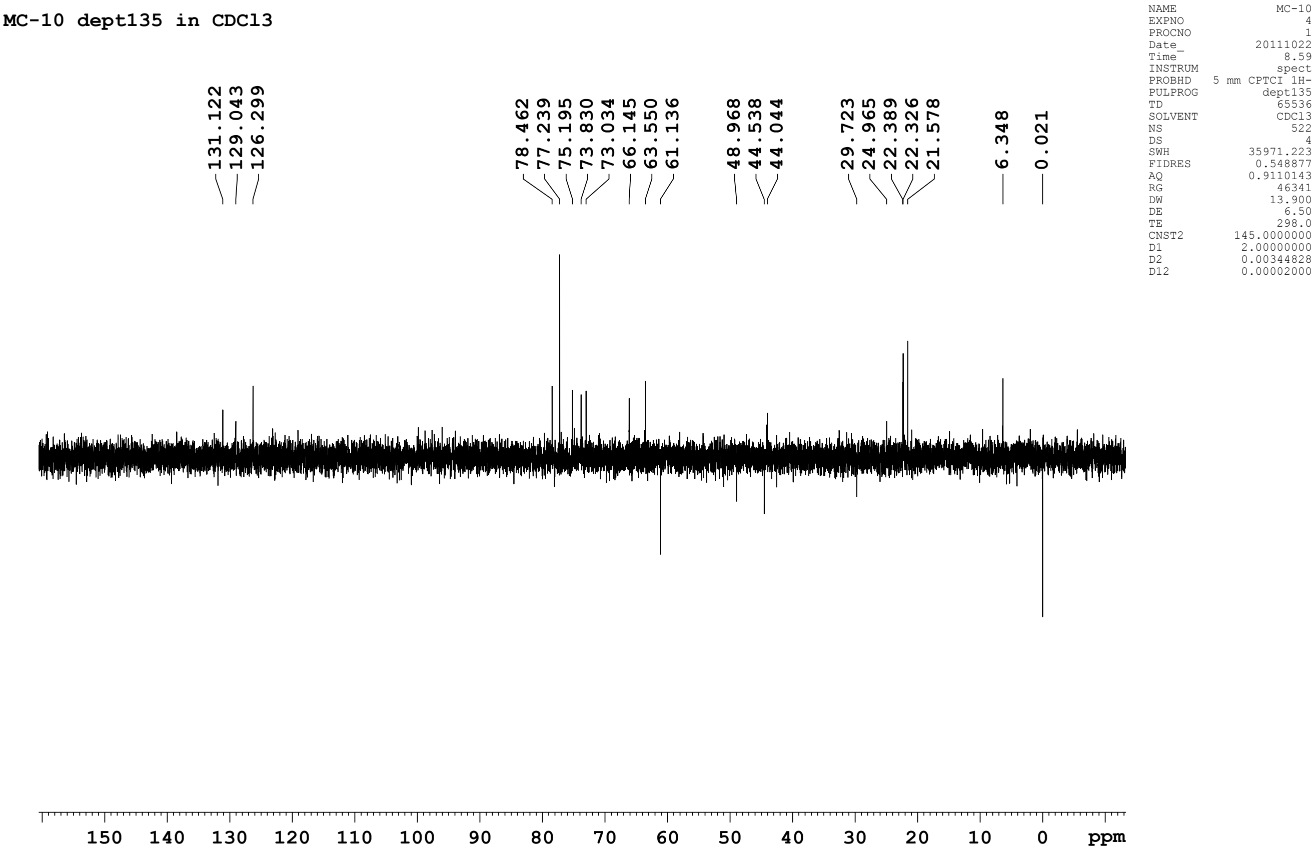

Figure S109. HSQC spectrum of compound **14** in $CDCl_3$.

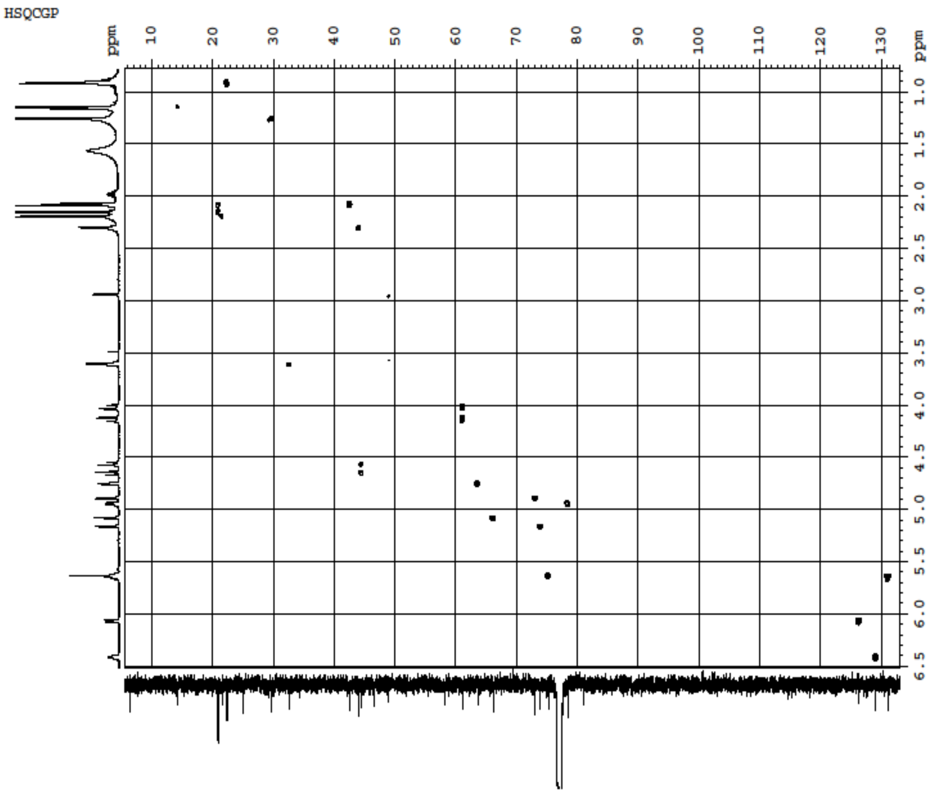

Figure S110. ^{1}H-^{1}H COSY spectrum of compound **14** in $CDCl_3$.

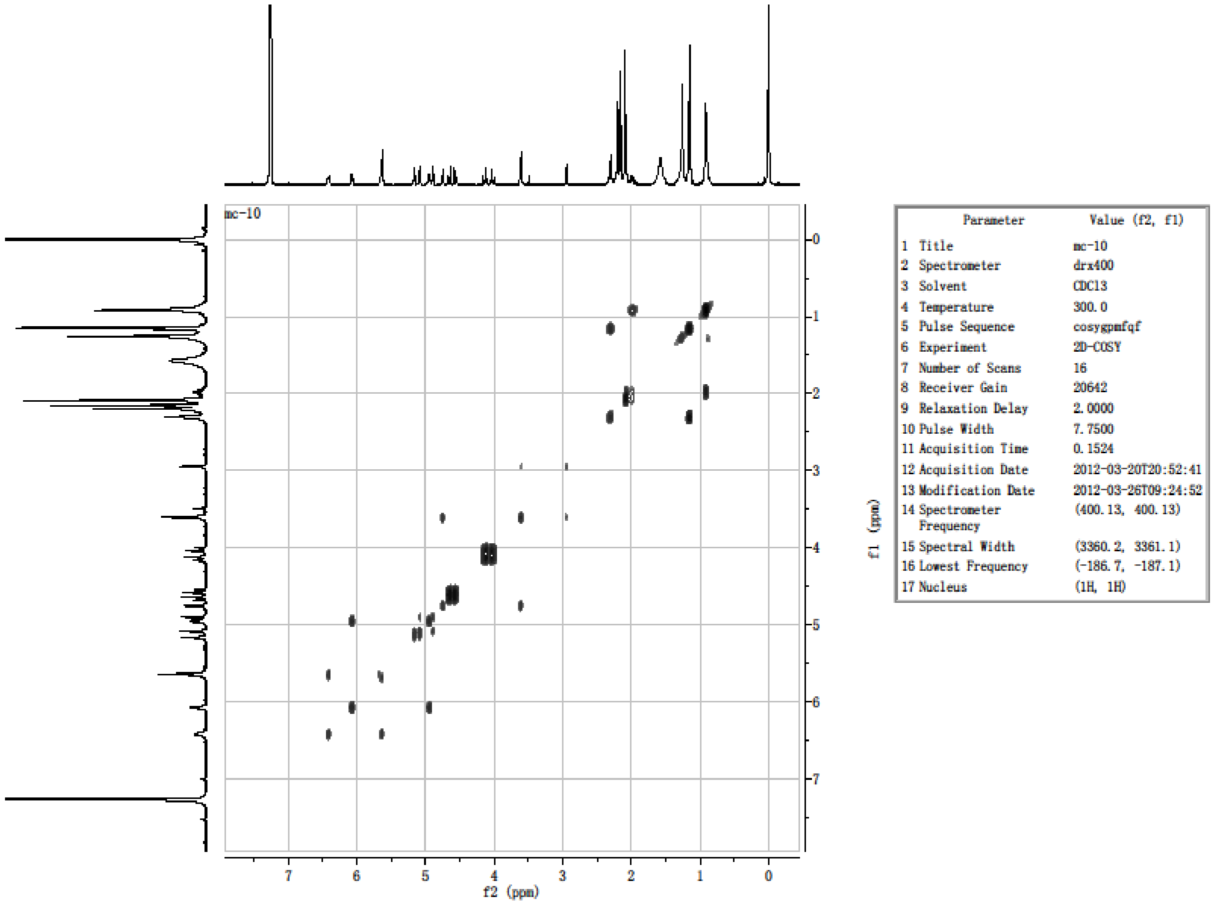

Figure S111. HMBC spectrum of compound **14** in $CDCl_3$.

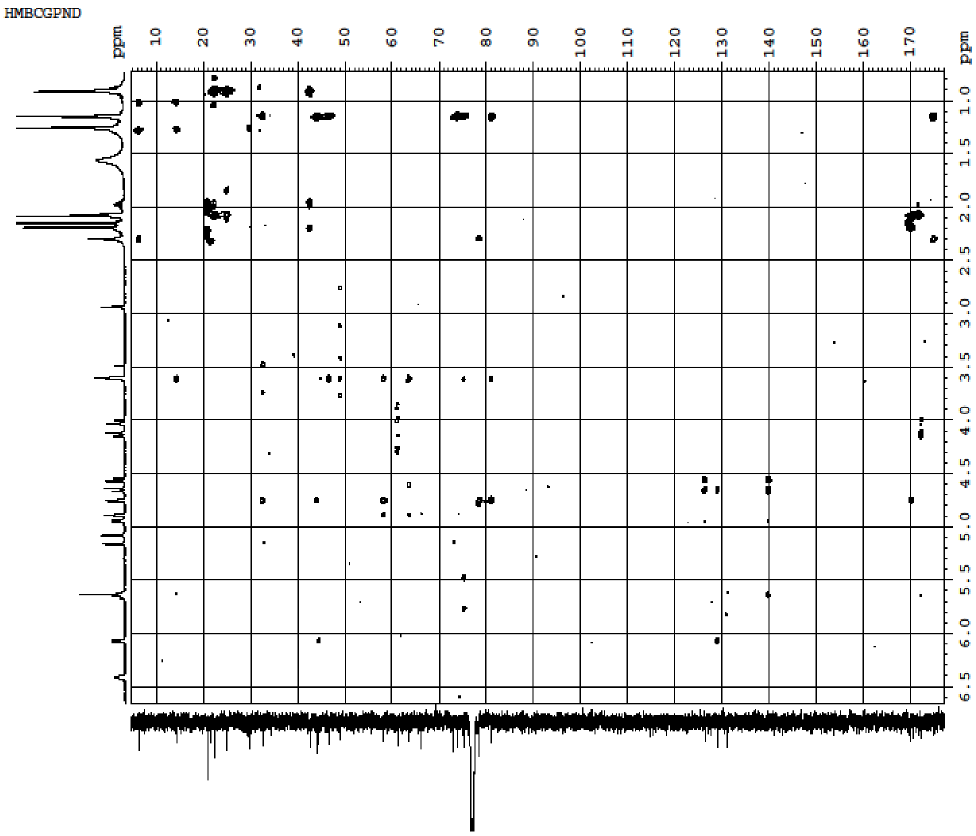

Figure S112. NOESY spectrum of compound **14** in $CDCl_3$.

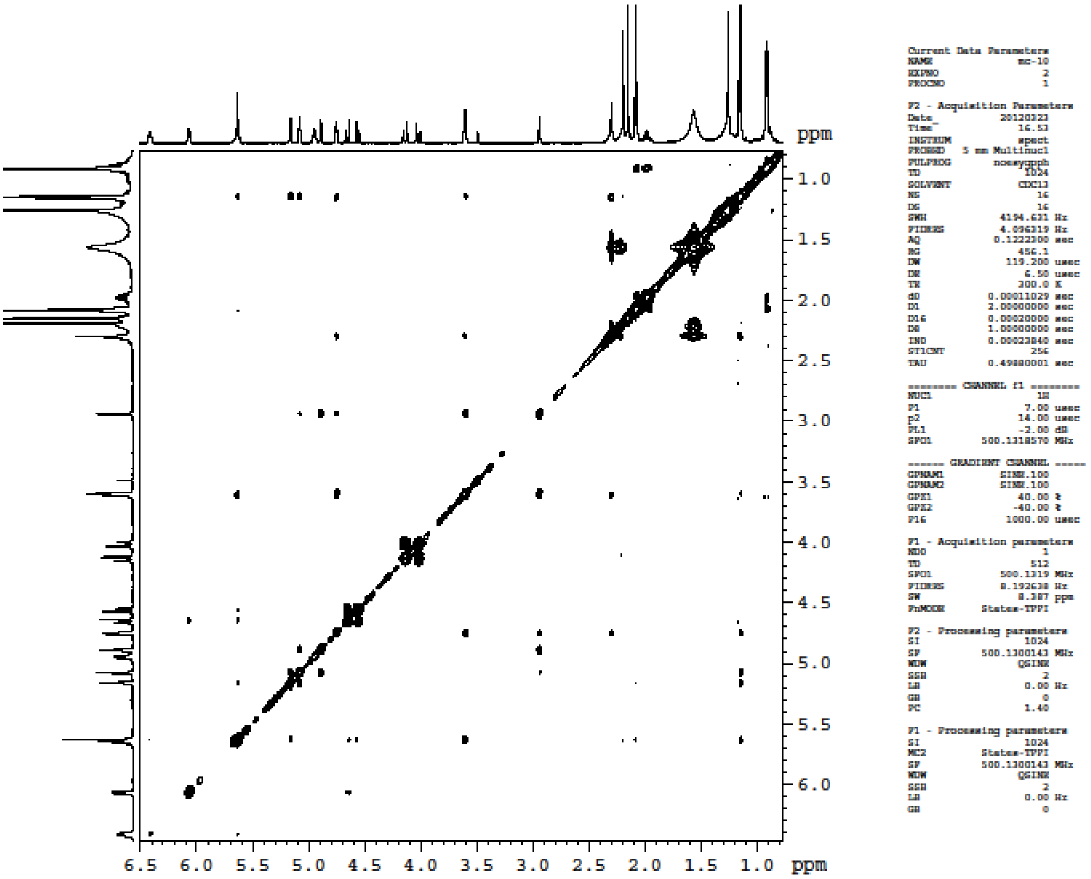

Figure S113. HR-ESIMS spectrum of the new compound **15**.

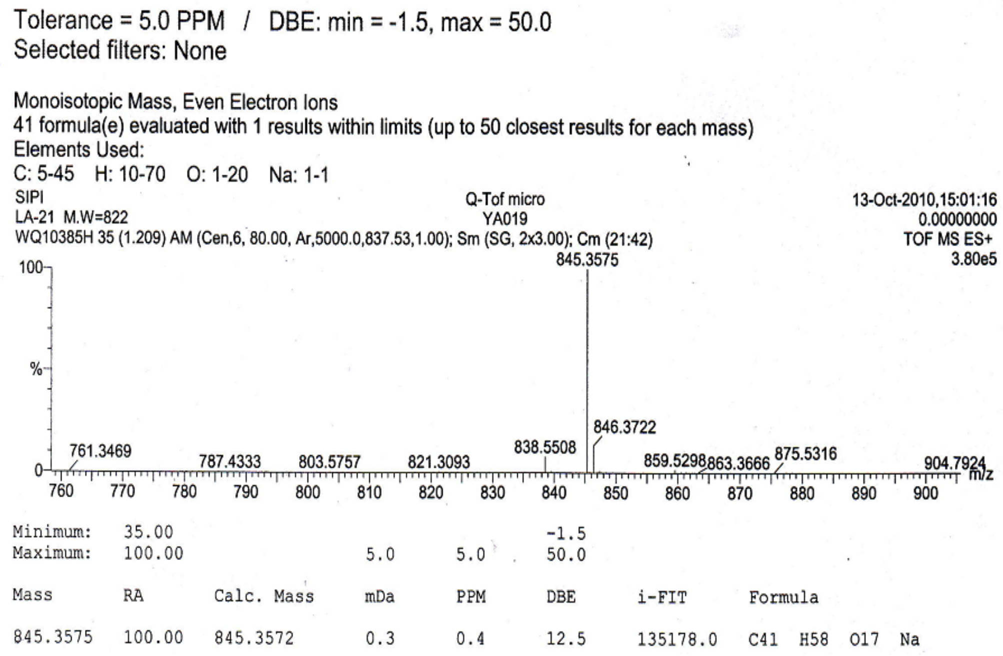

Figure S114. ^{1}H NMR spectrum of the new compound **15**.

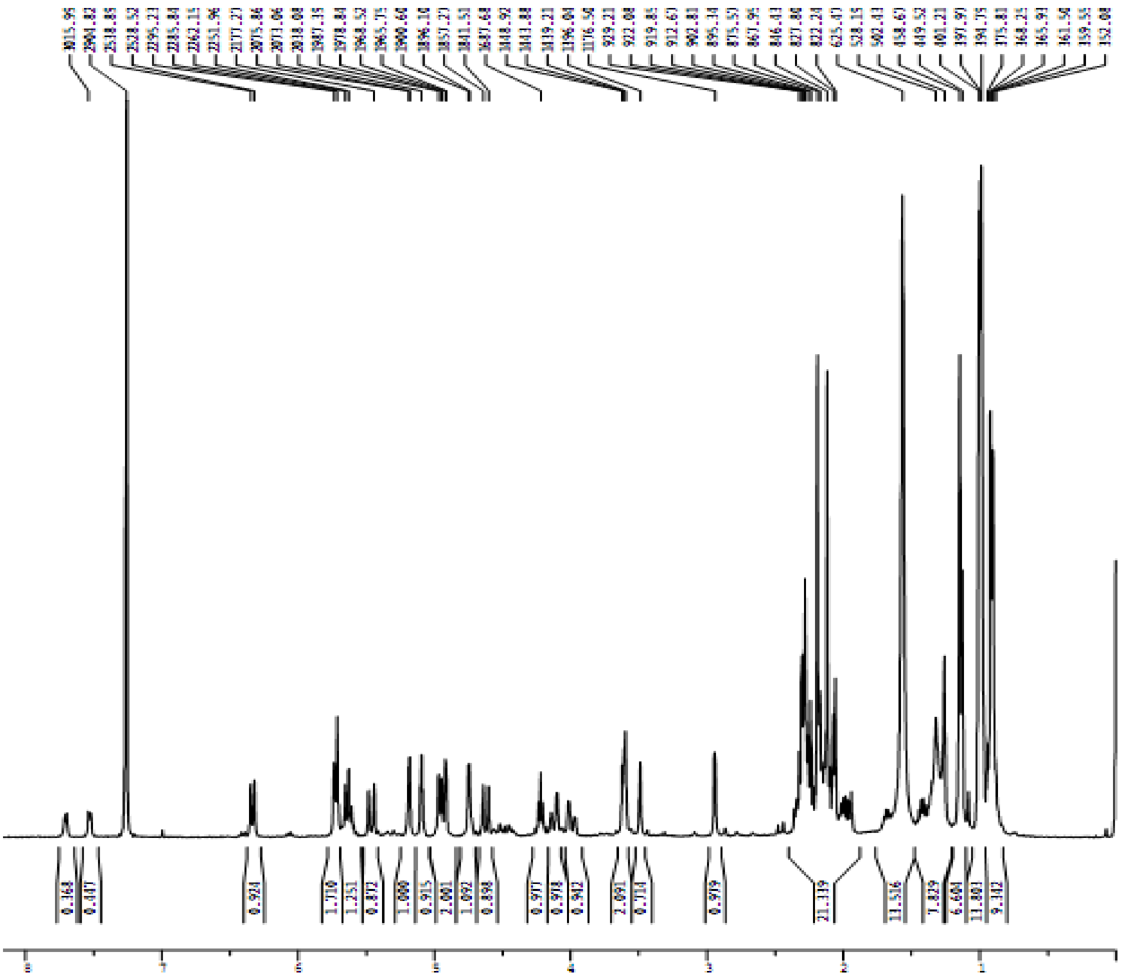

Figure S115. ^{13}C NMR spectrum of the new compound **15**.

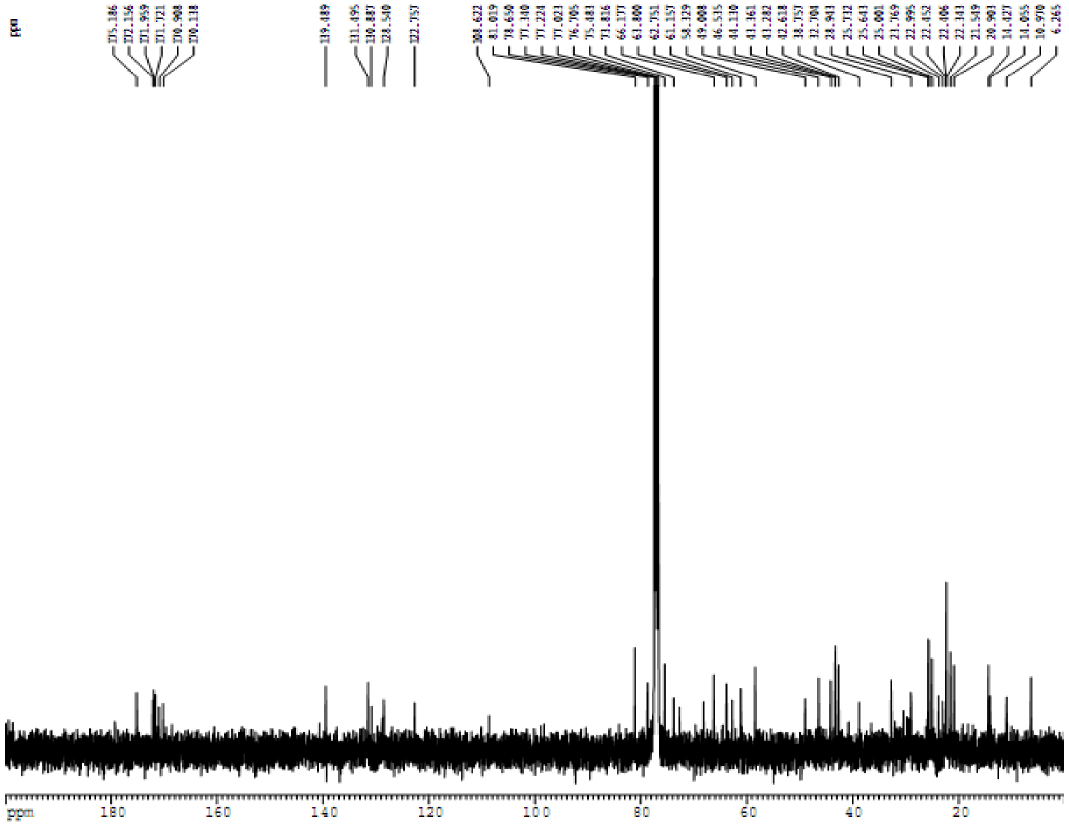

Figure S116. DEPT spectrum of the new compound **15**.

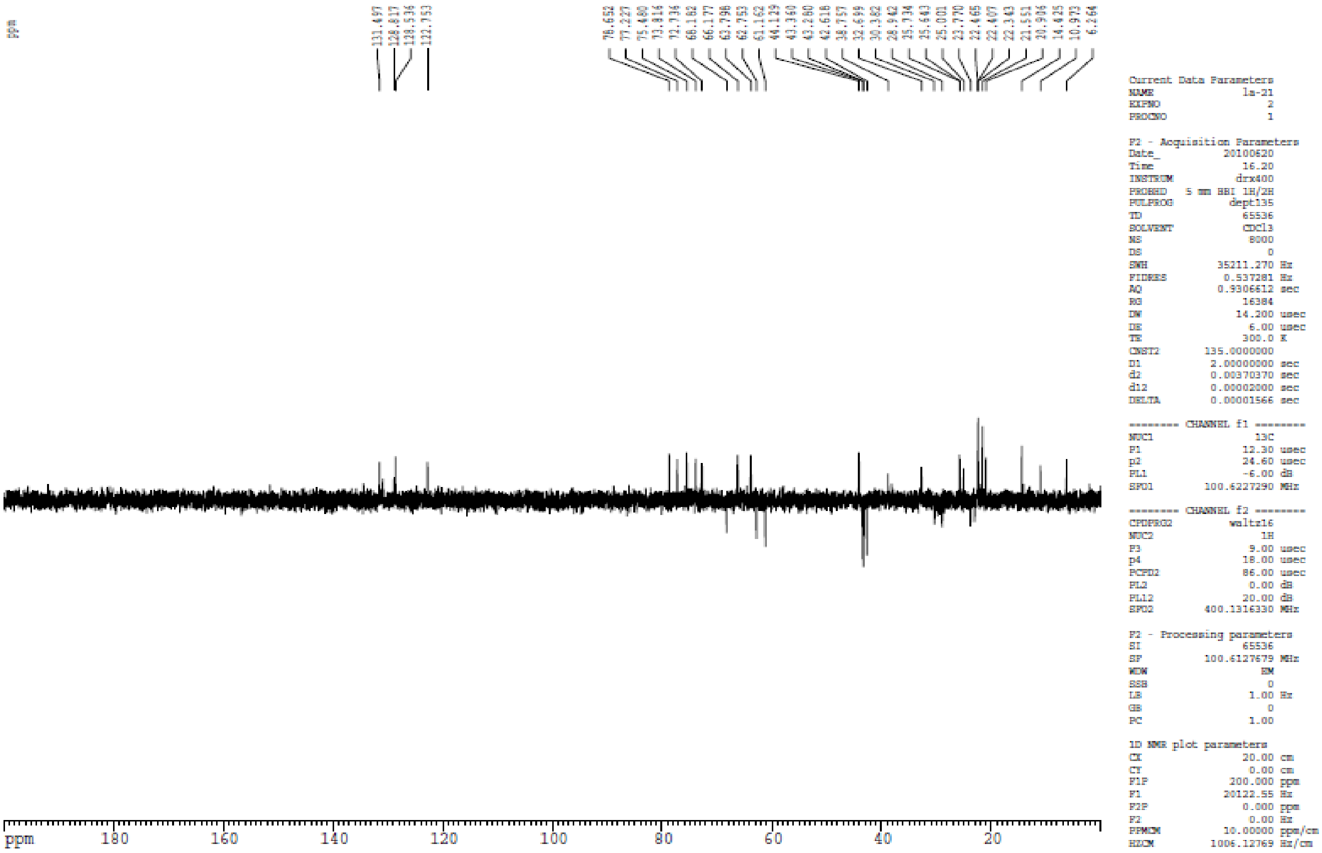

Figure S117. HSQC spectrum of the new compound **15**.

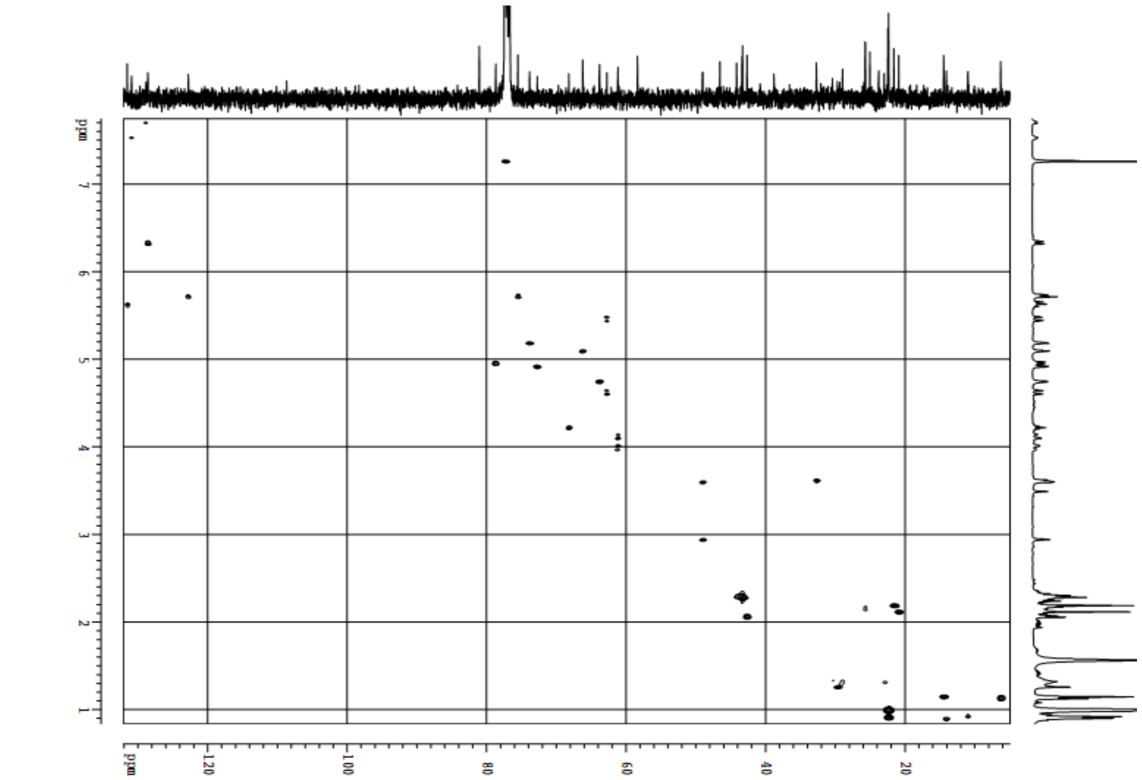

Figure S118. ^{1}H-^{1}H COSY spectrum of the new compound **15**.

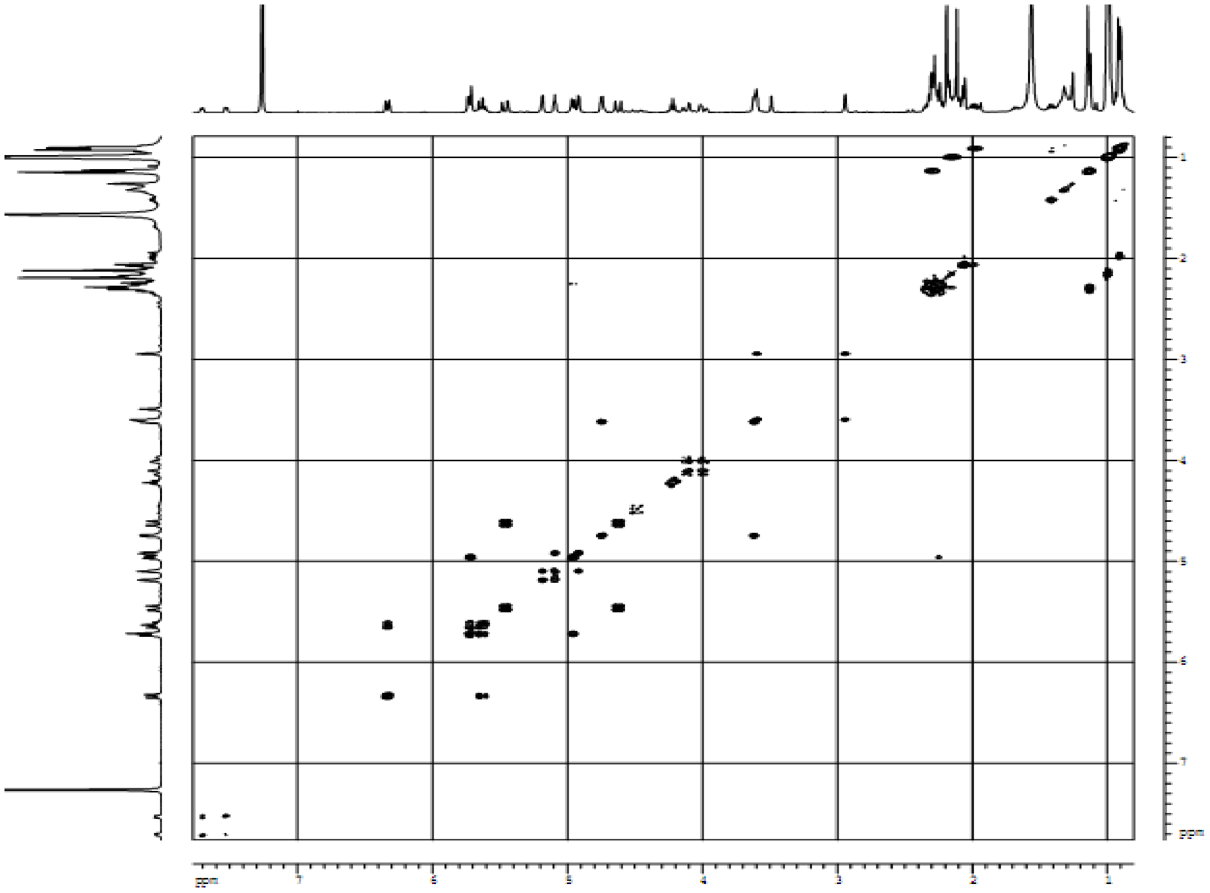

Figure S119. HMBC spectrum of the new compound **15**.

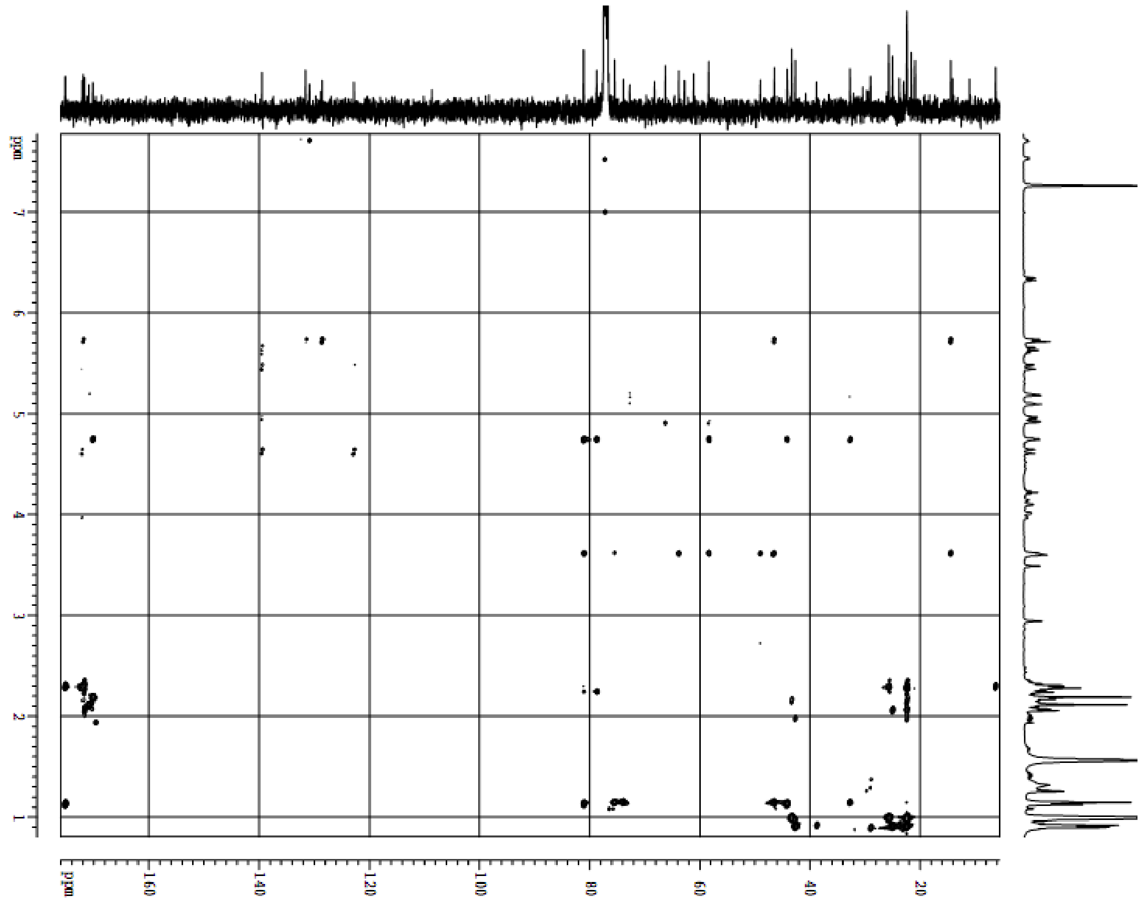

Figure S120. NOESY spectrum of the new compound **15**.

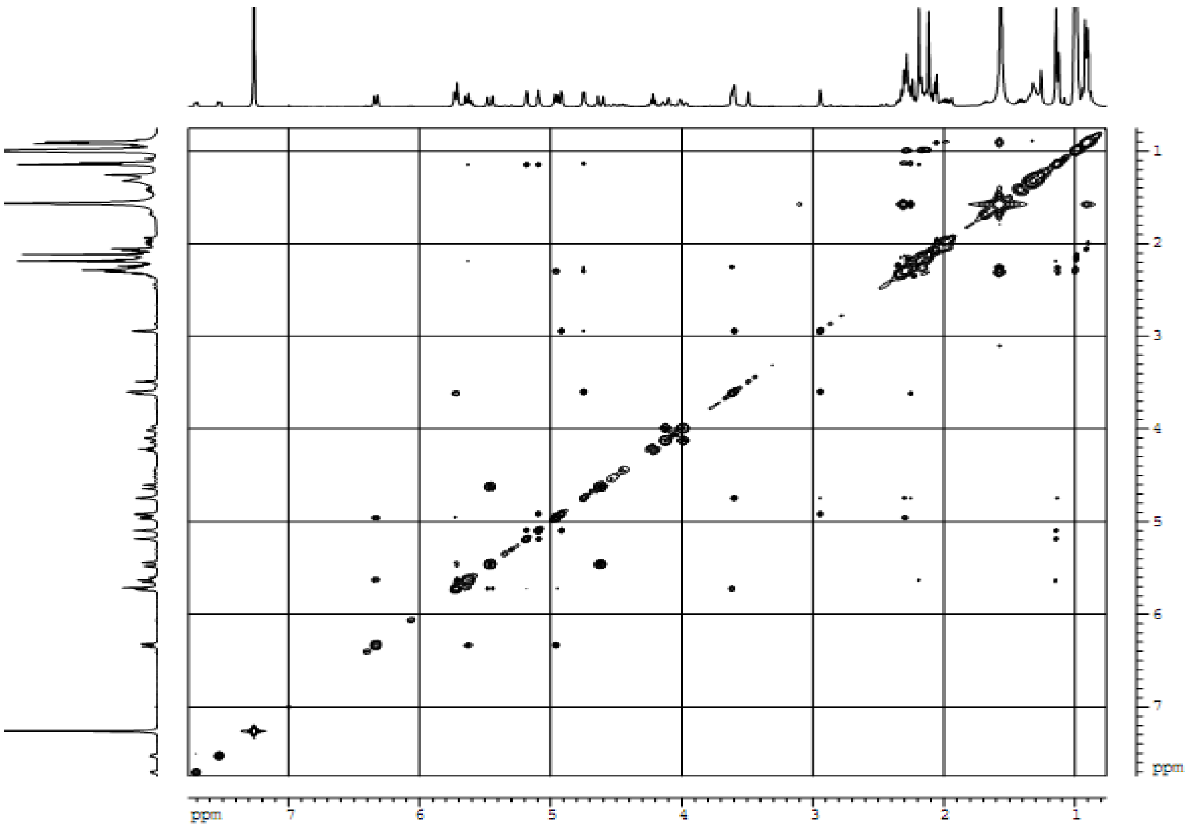

Figure S121. HRESIMS spectrum of compound **16**.

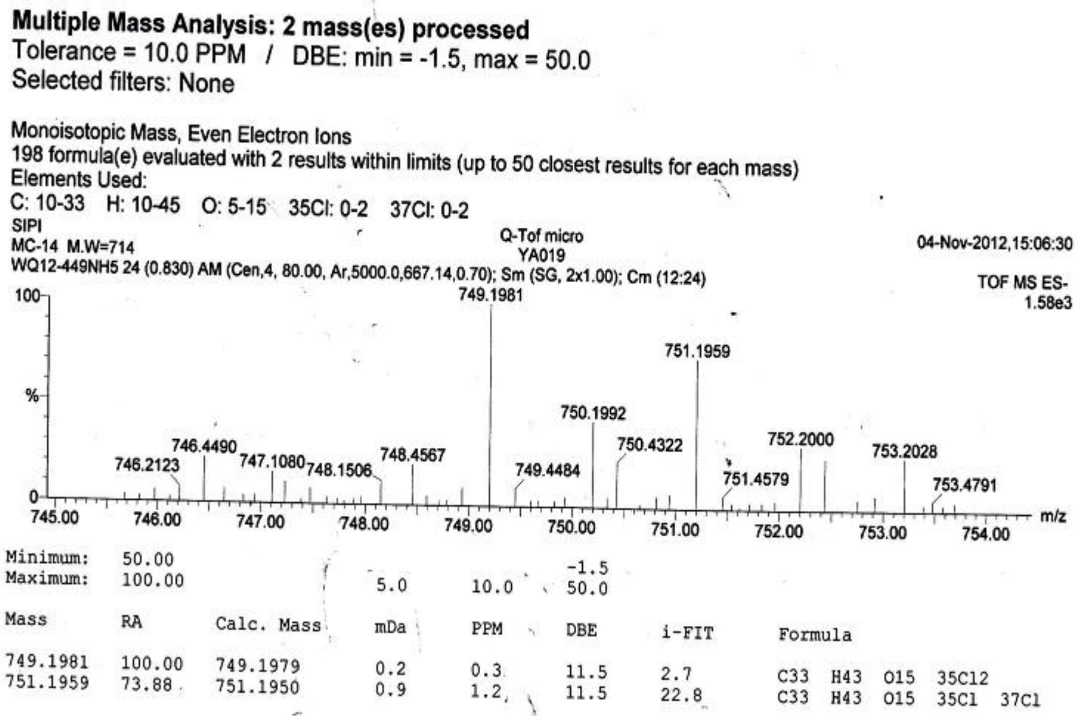

Figure S122. ^1H NMR spectrum of compound **16** in $CDCl_3$.

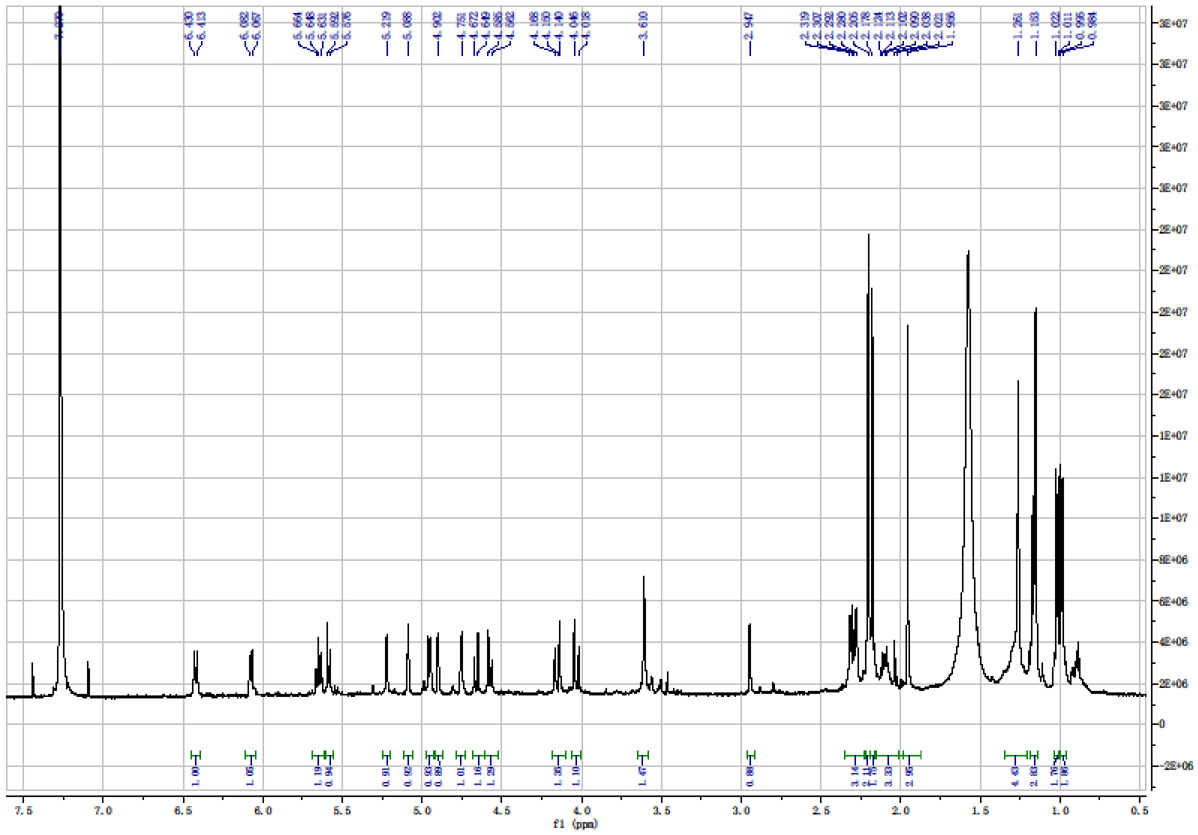

Figure S123. ^{13}C NMR spectrum of compound **16** in $CDCl_3$.

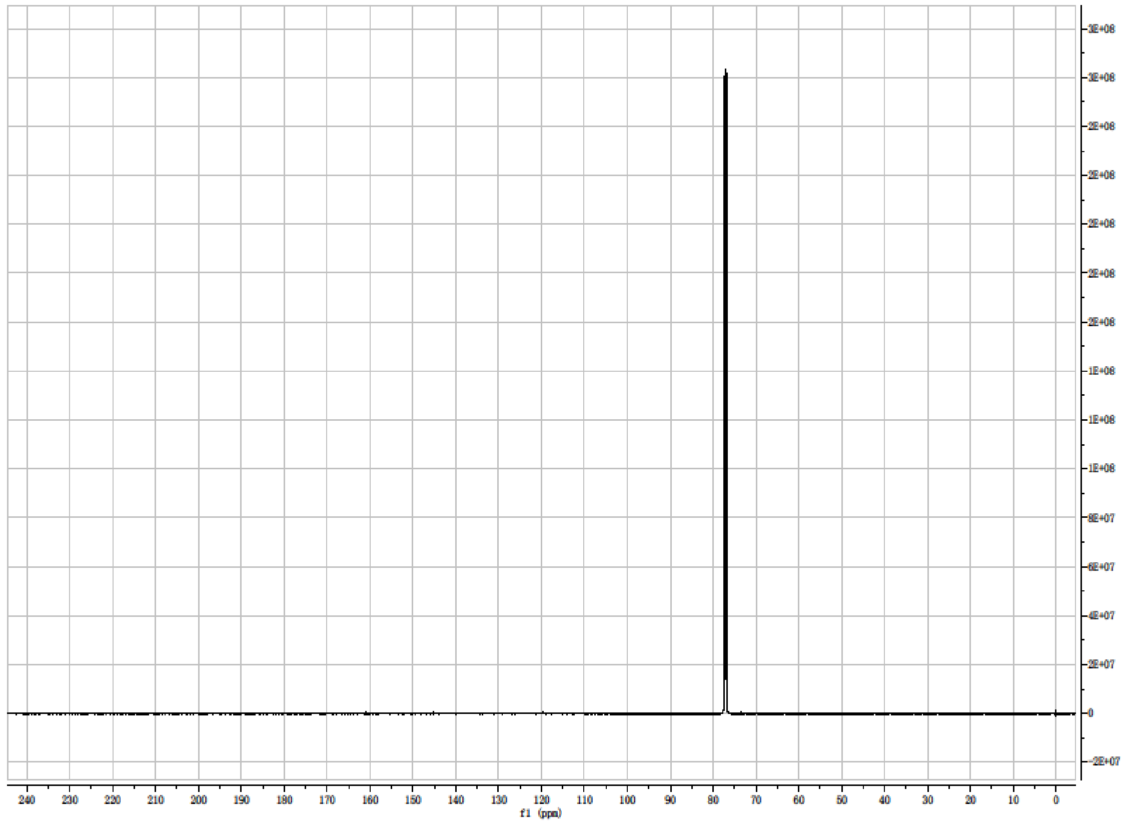

Figure S124. DEPT spectrum of compound **16** in $CDCl_3$.

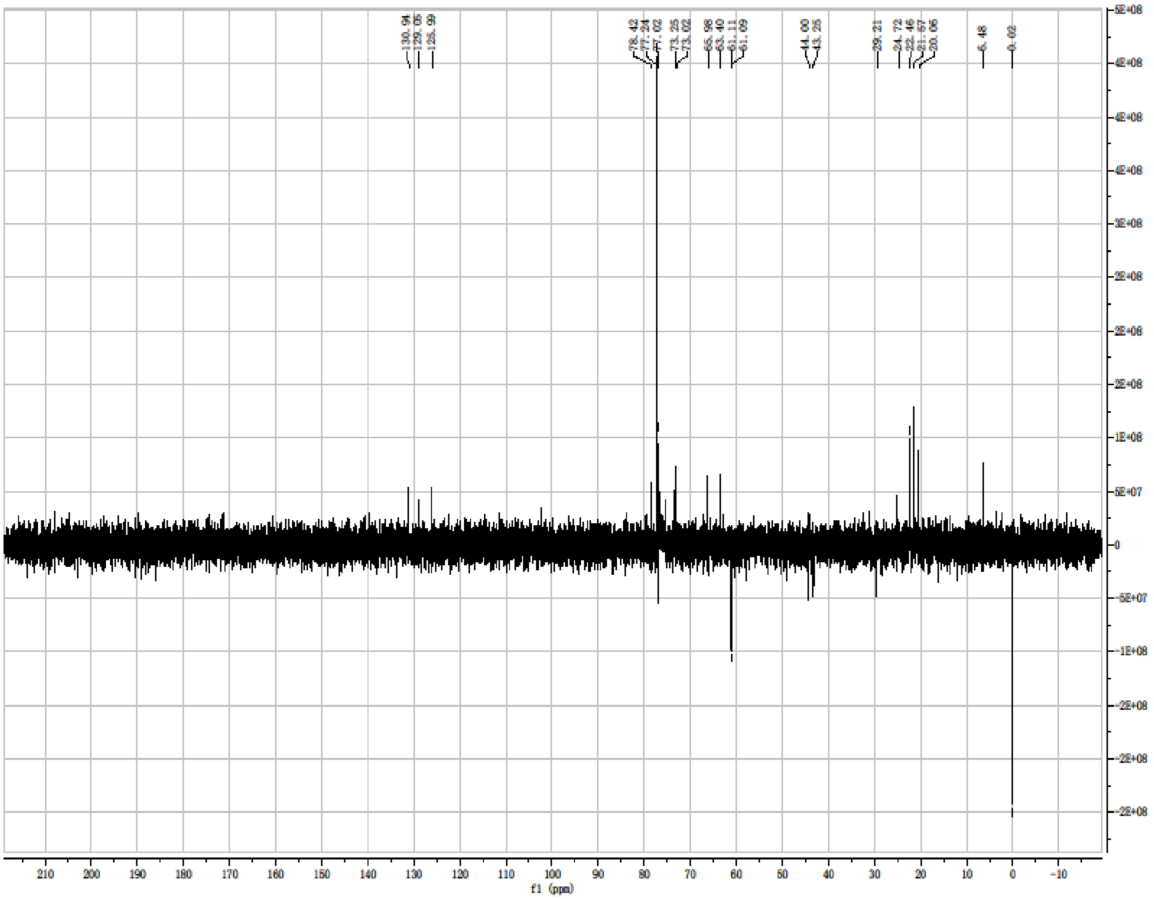

Figure S125. HSQC spectrum of compound **16** in $CDCl_3$.

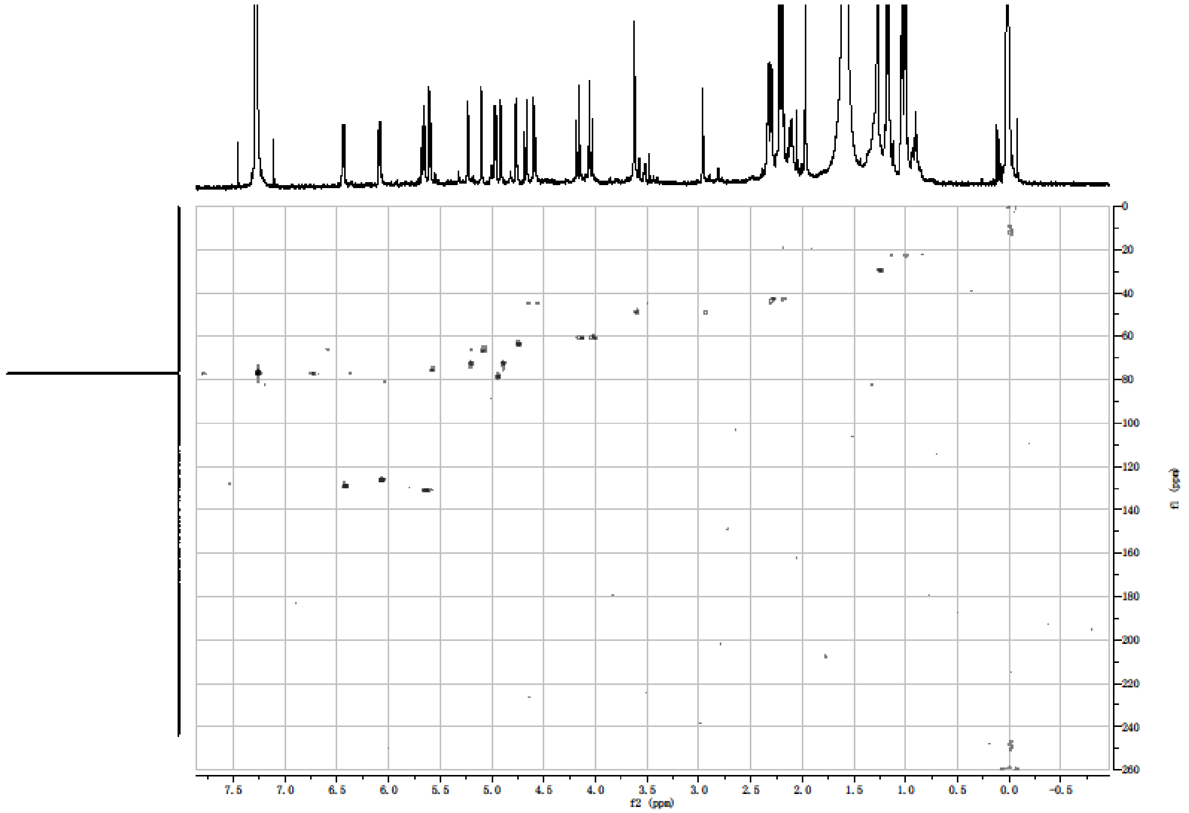

Figure S126. ^1H-^1H COSY spectrum of compound **16** in $CDCl_3$.

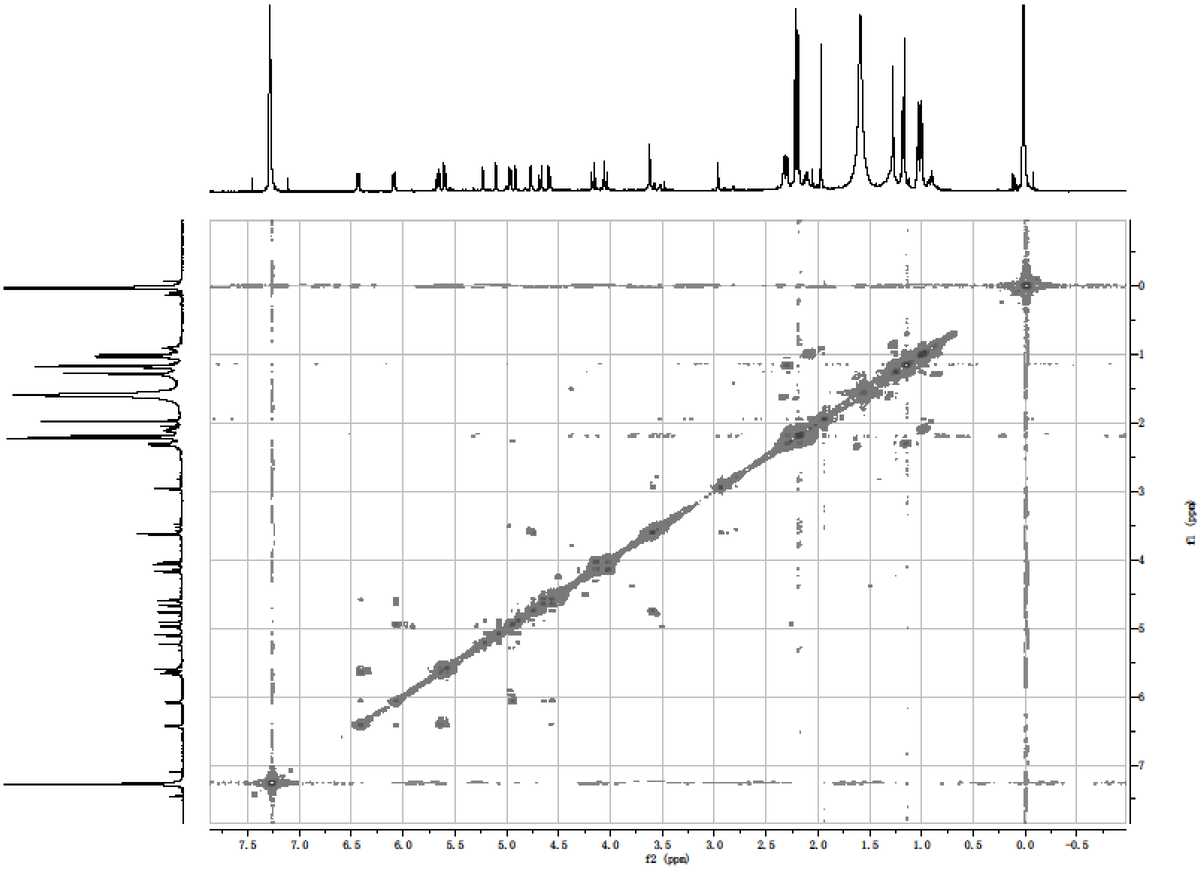

Figure S127. HMBC spectrum of compound **16** in $CDCl_3$.

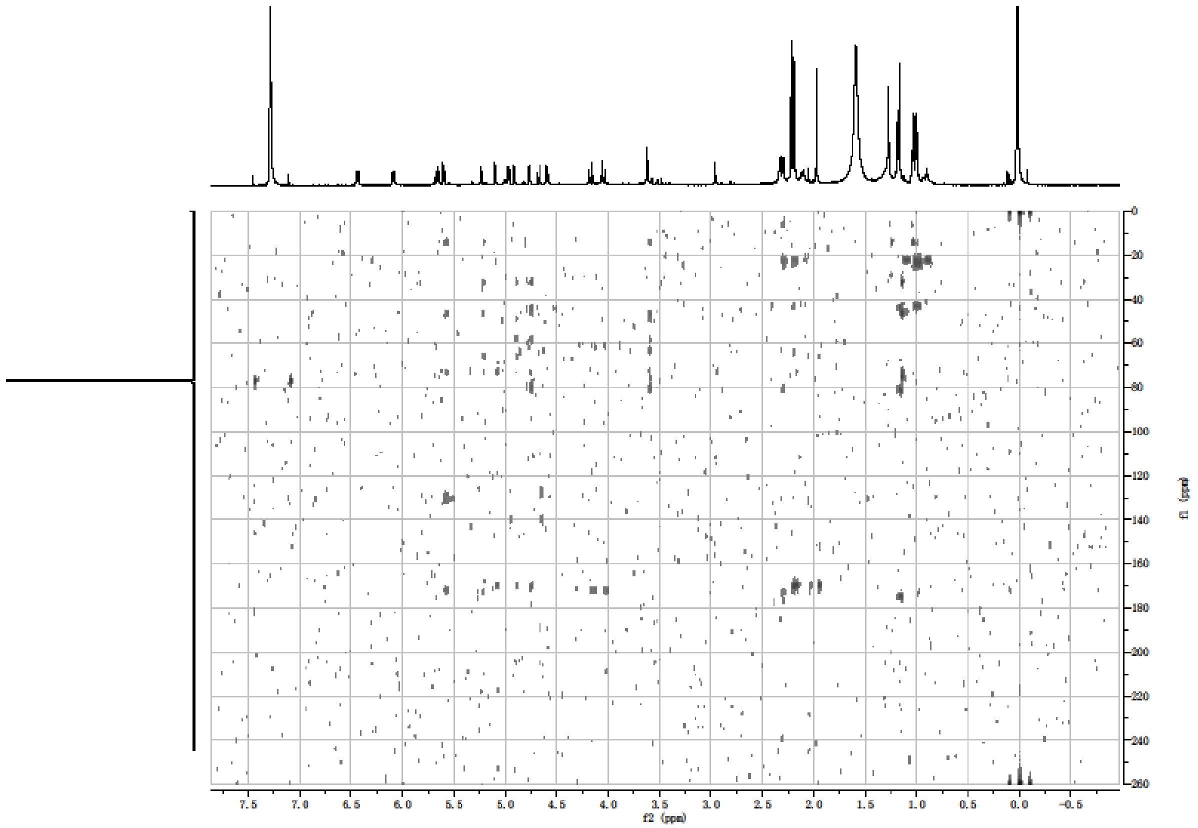

Figure S128. NOESY spectrum of compound **16** in $CDCl_3$.

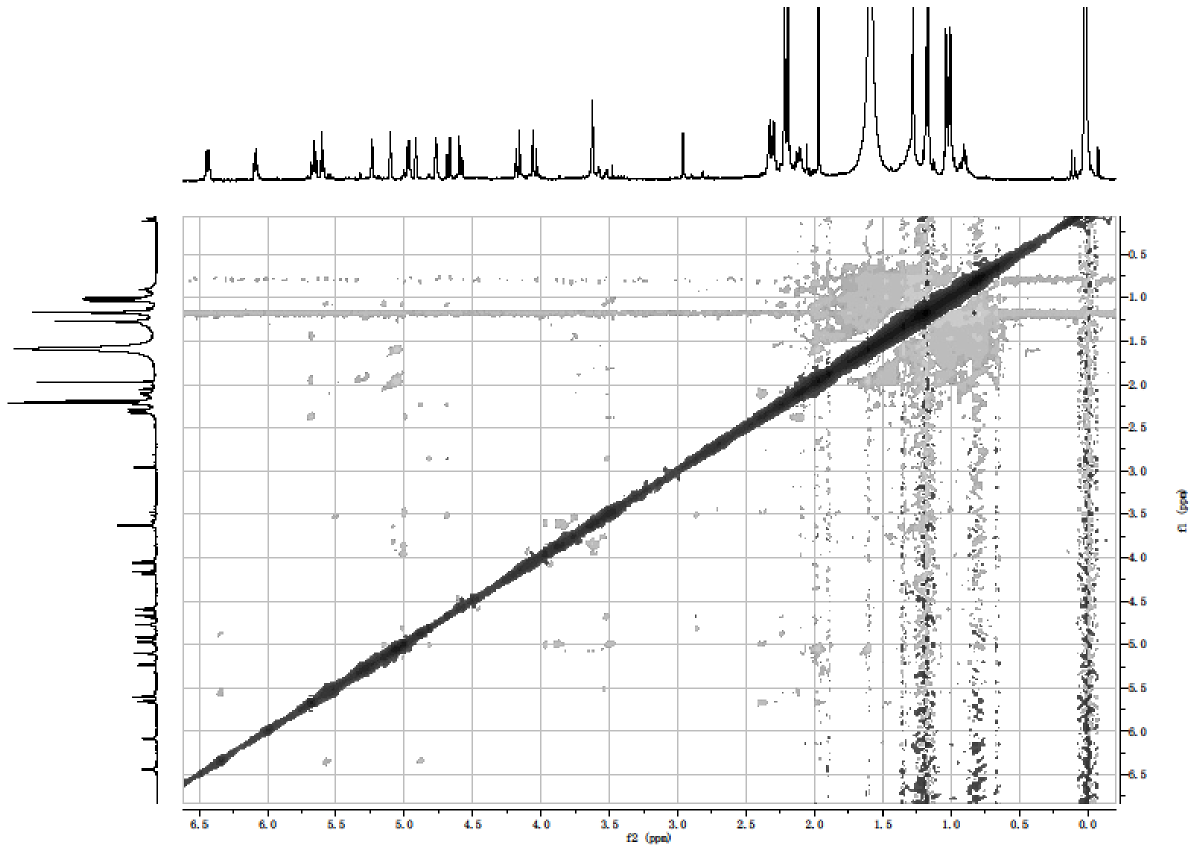

Figure S129. HR-ESIMS spectrum of the new compound **17**.

Multiple Mass Analysis: 2 mass(es) processed
Tolerance = 10.0 PPM / DBE: min = -1.5, max = 50.0
Selected filters: None

Monoisotopic Mass, Even Electron Ions
15 formula(e) evaluated with 1 results within limits (up to 50 closest results for each mass)
Elements Used:
C: 5-35 H: 1-50 O: 2-15 Na: 1-1

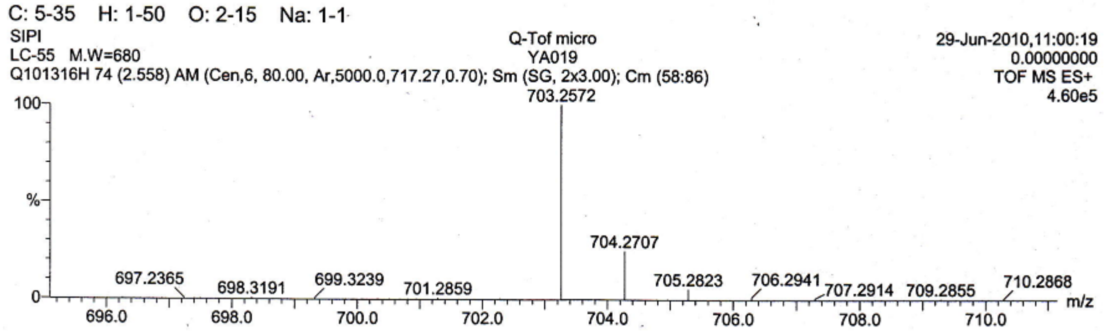

Minimum:	30.00				-1.5		
Maximum:	100.00		5.0	10.0	50.0		
Mass	RA	Calc. Mass	mDa	PPM	DBE	i-FIT	Formula
703.2572	100.00	703.2578	-0.6	-0.9	11.5	14762.5	C33 H44 O15 Na

Figure S130. ^{1}H MNR (400 MHz, $CDCl_3$) spectrum of the new compound **17**.

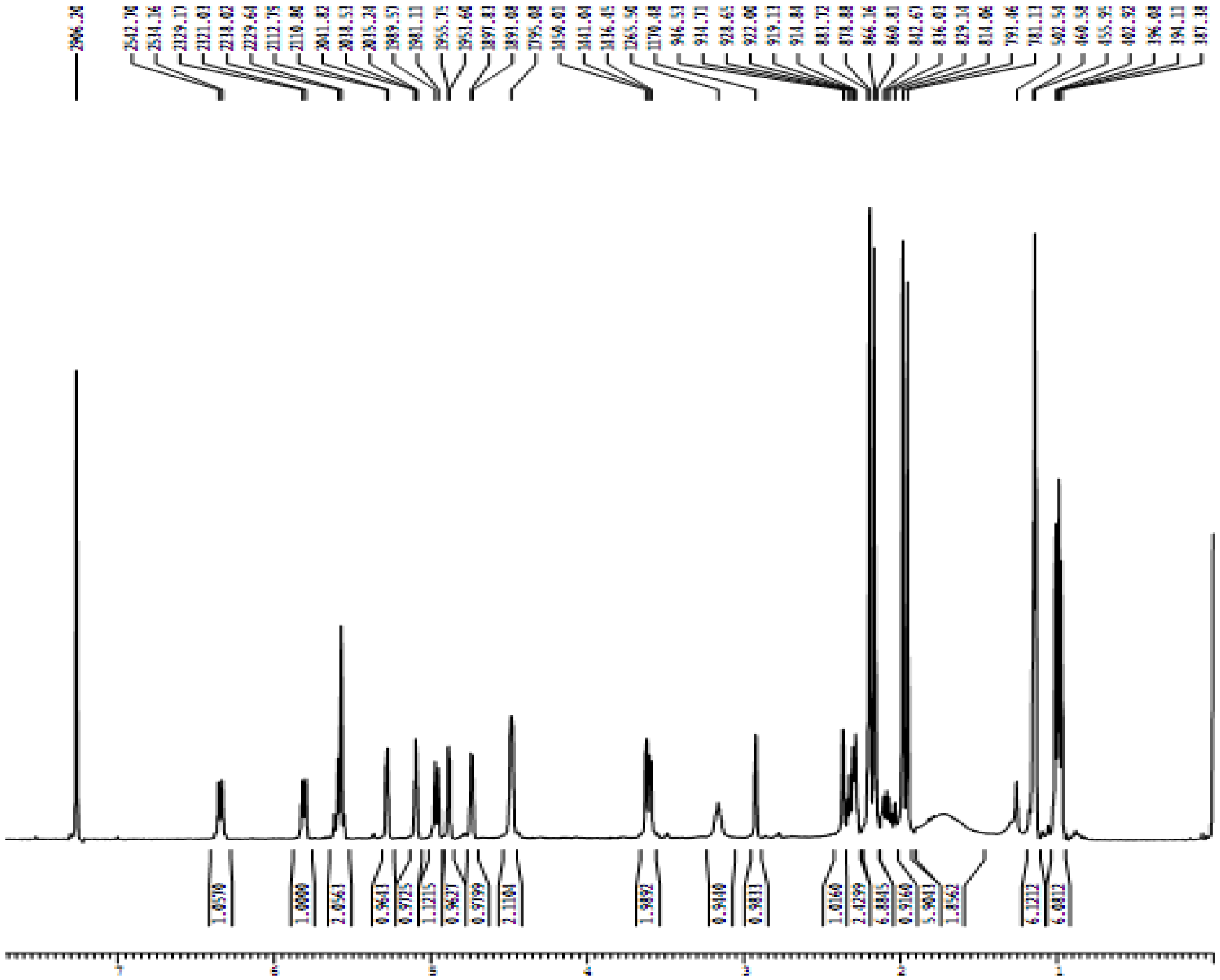

Figure S131. ^{13}C MNR (100 MHz, $CDCl_3$) spectrum of the new compound **17**.

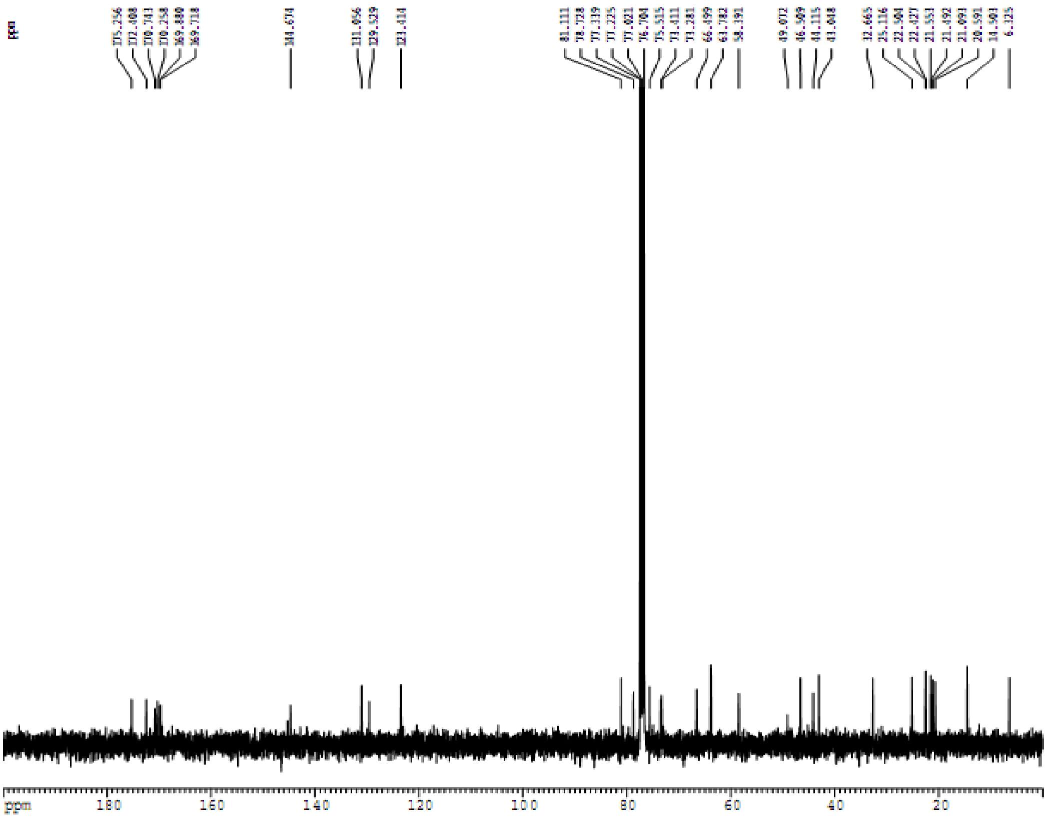

Figure S132. DEPT spectrum of the new compound **17**.

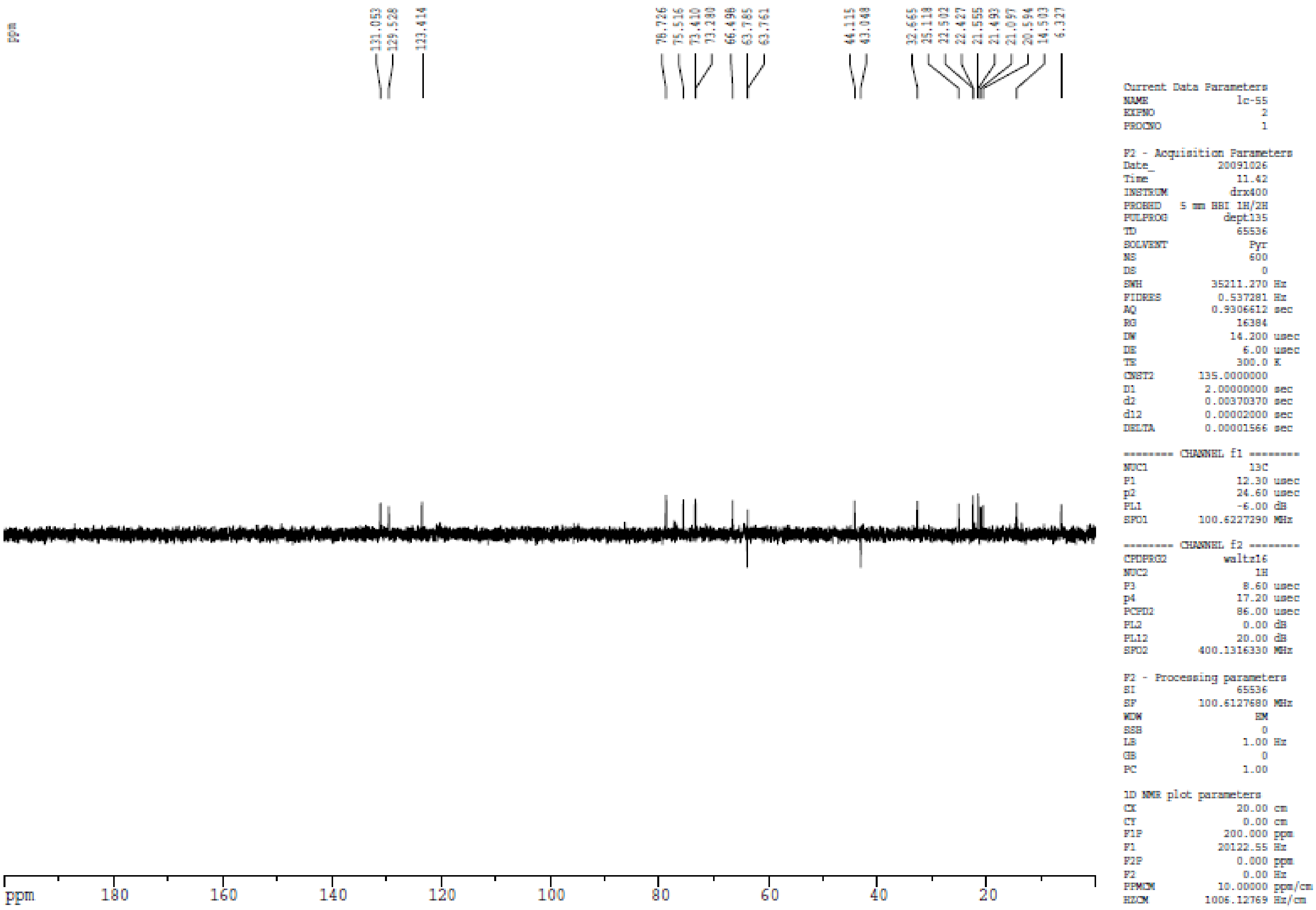

Figure S133. HSQC spectrum of the new compound **17**.

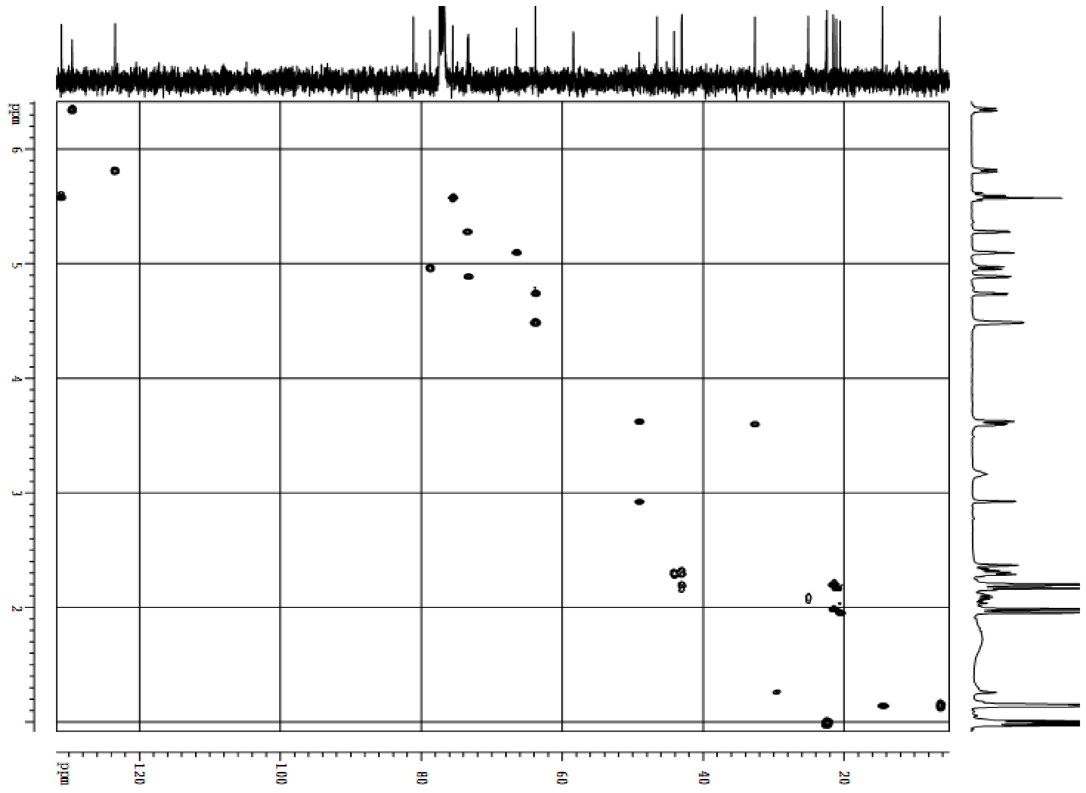

Figure S134. ^{1}H-^{1}H COSY spectrum of the new compound **17**.

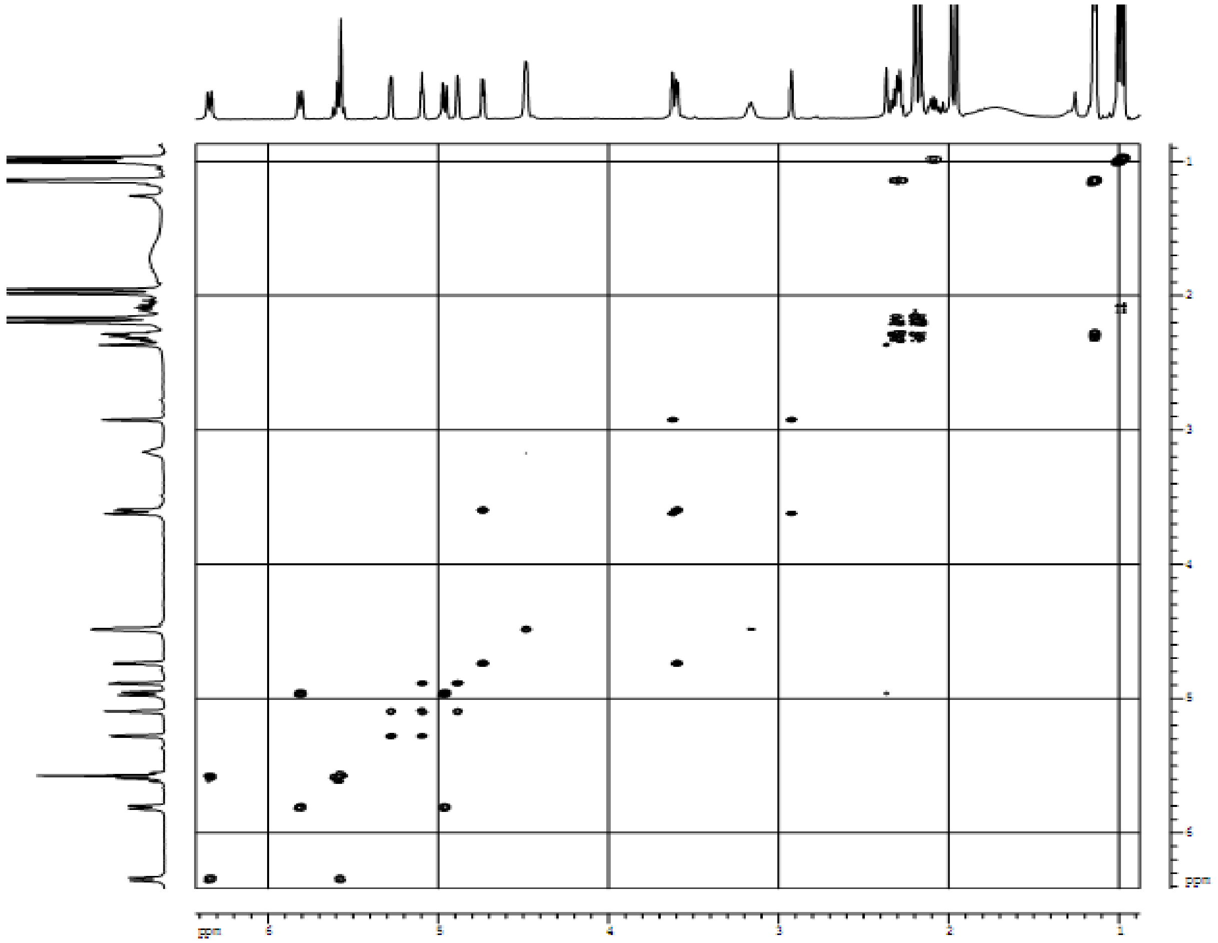

Figure S135. HMBC spectrum of the new compound **17**.

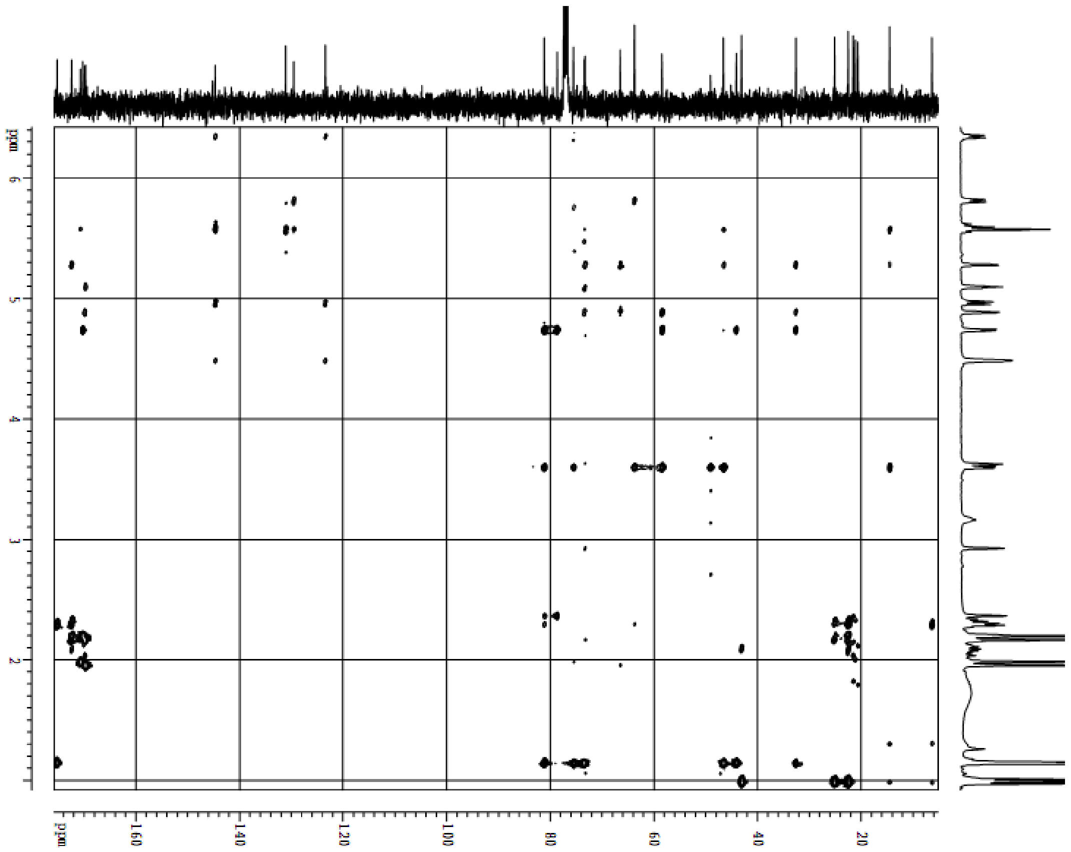

Figure S136. NOESY spectrum of the new compound **17**.

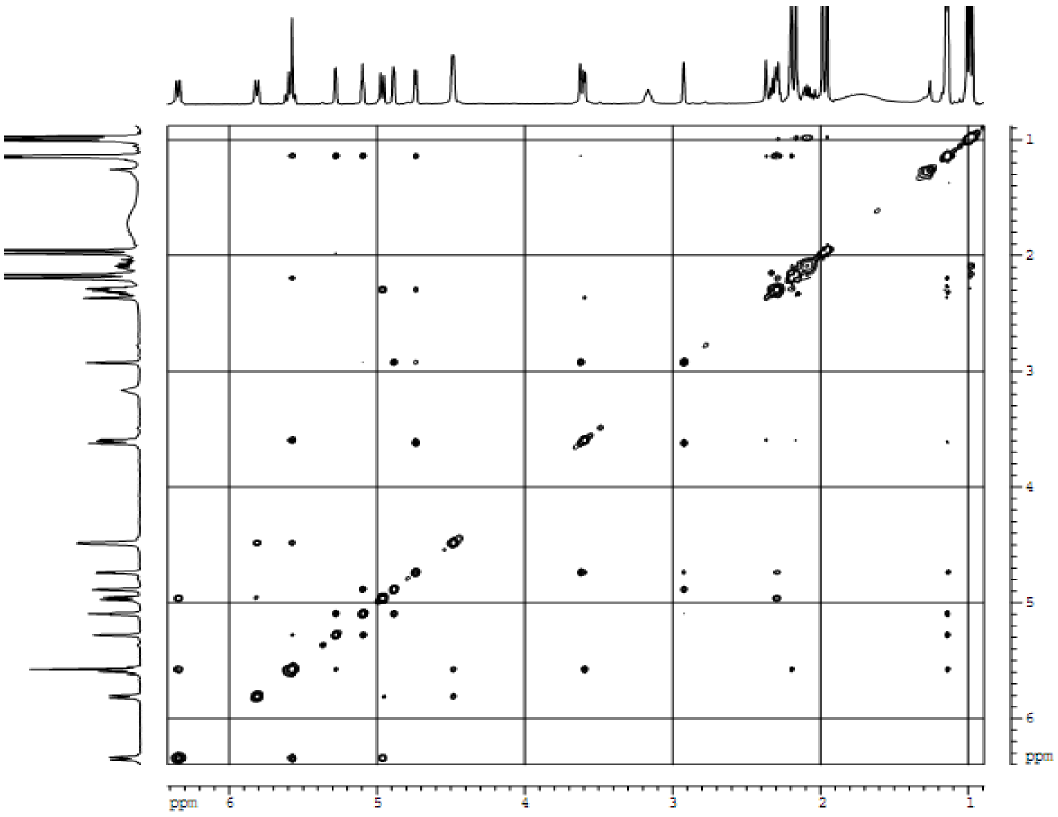

Figure S137. HR-ESIMS spectrum of the new compound **18**.

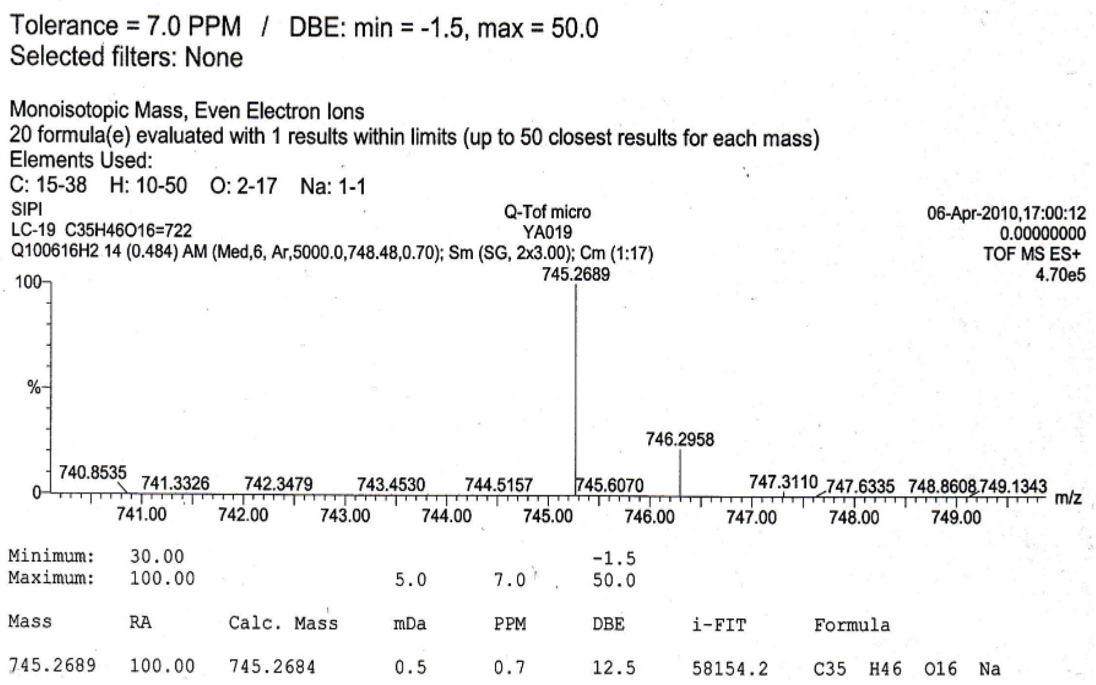

Figure S138. ^{1}H NMR spectrum of the new compound **18**.

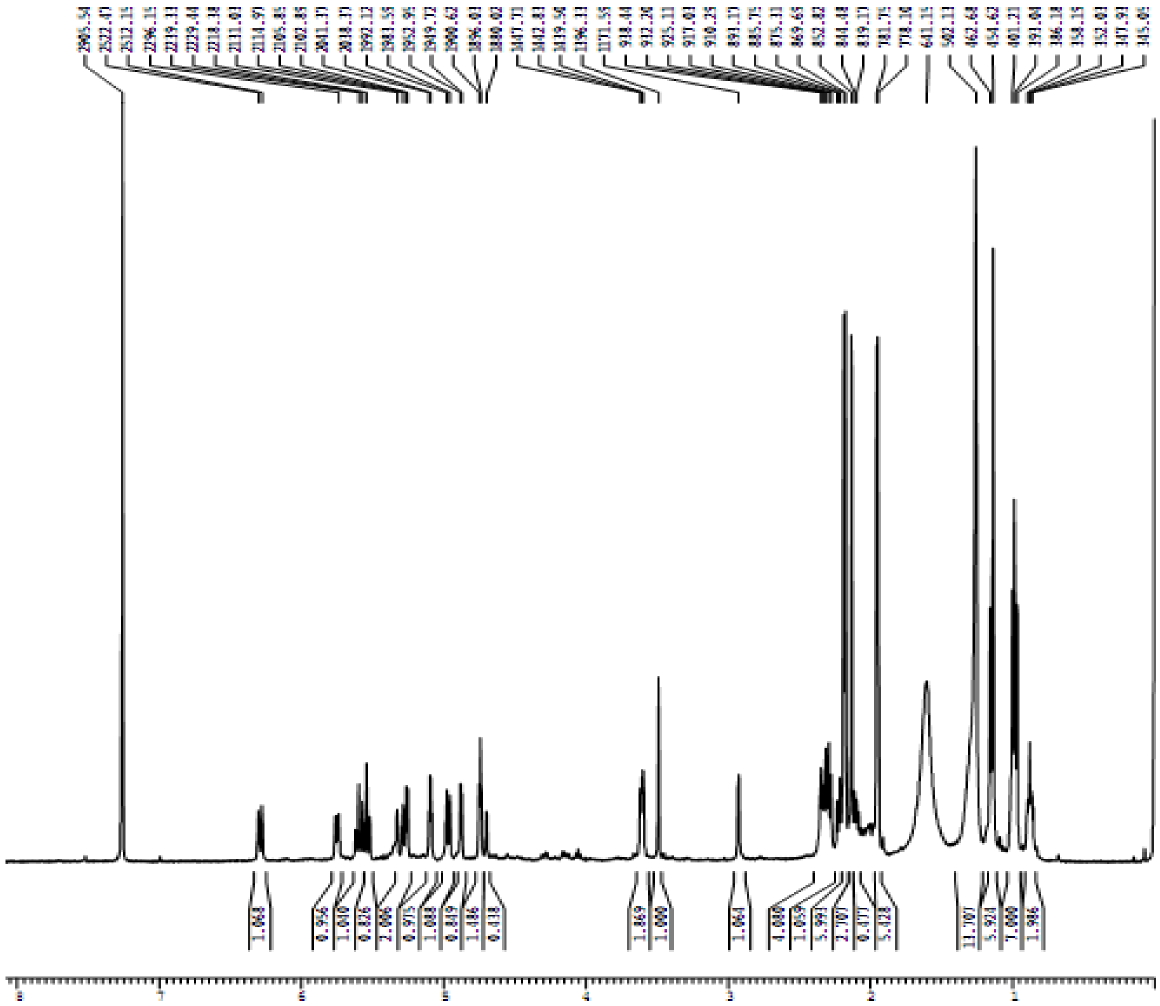

Figure S139. ^{13}C NMR spectrum of the new compound **18**.

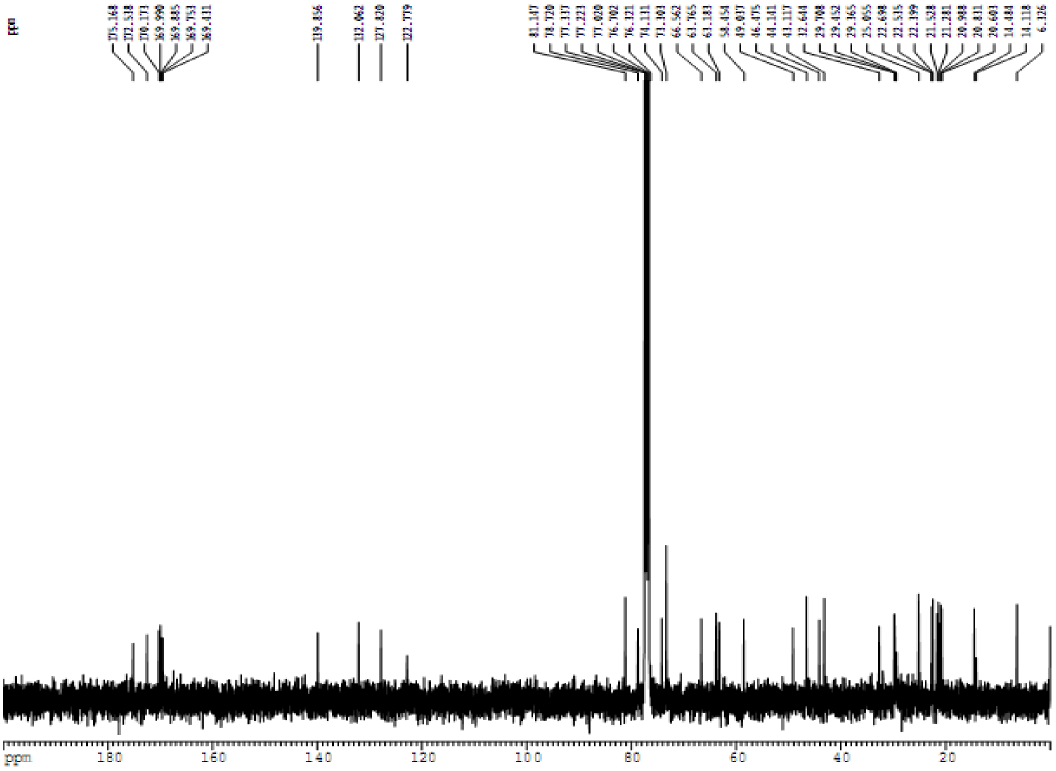

Figure S140. DEPT spectrum of the new compound **18**.

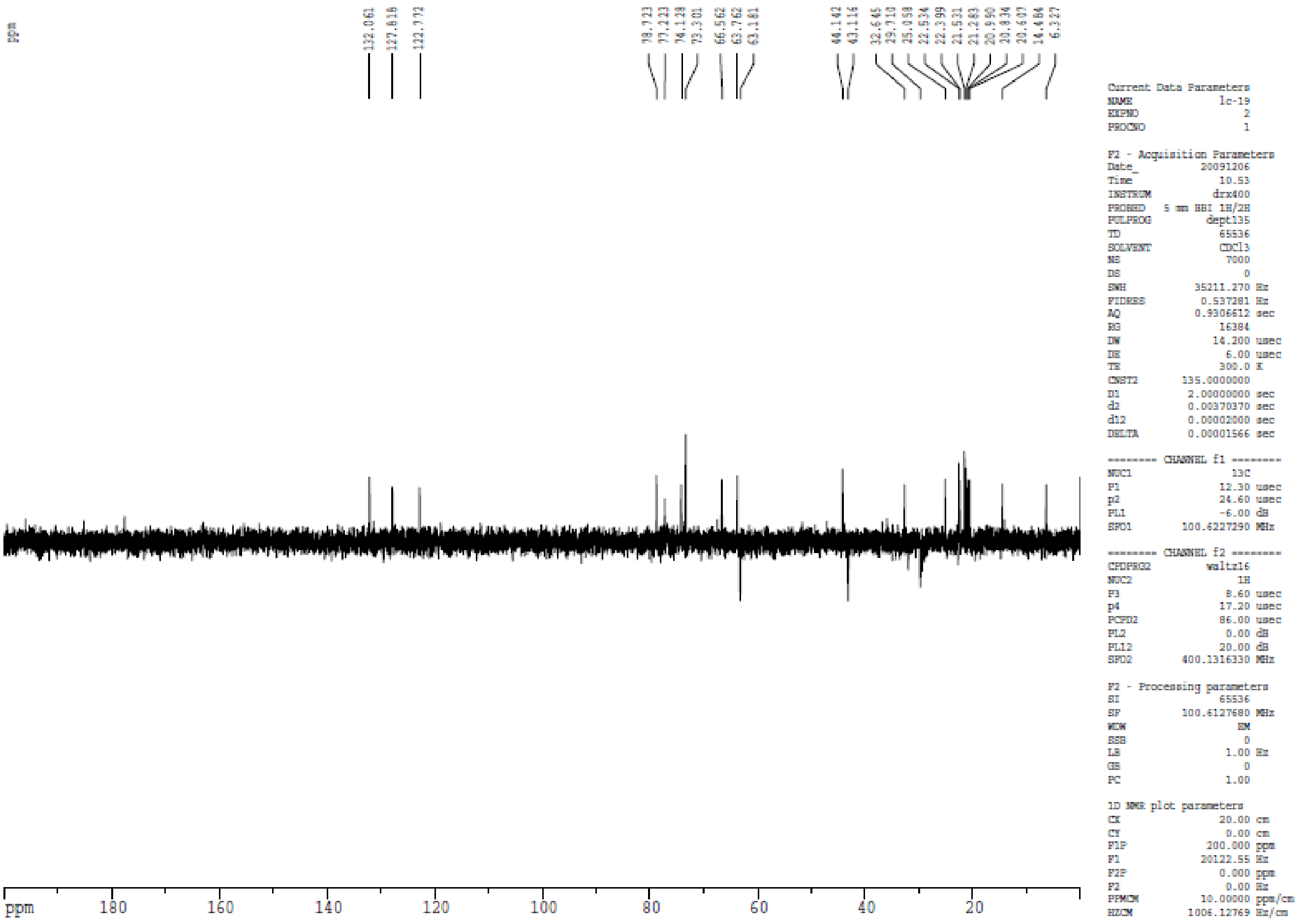

Figure S141. HSQC spectrum of the new compound **18**.

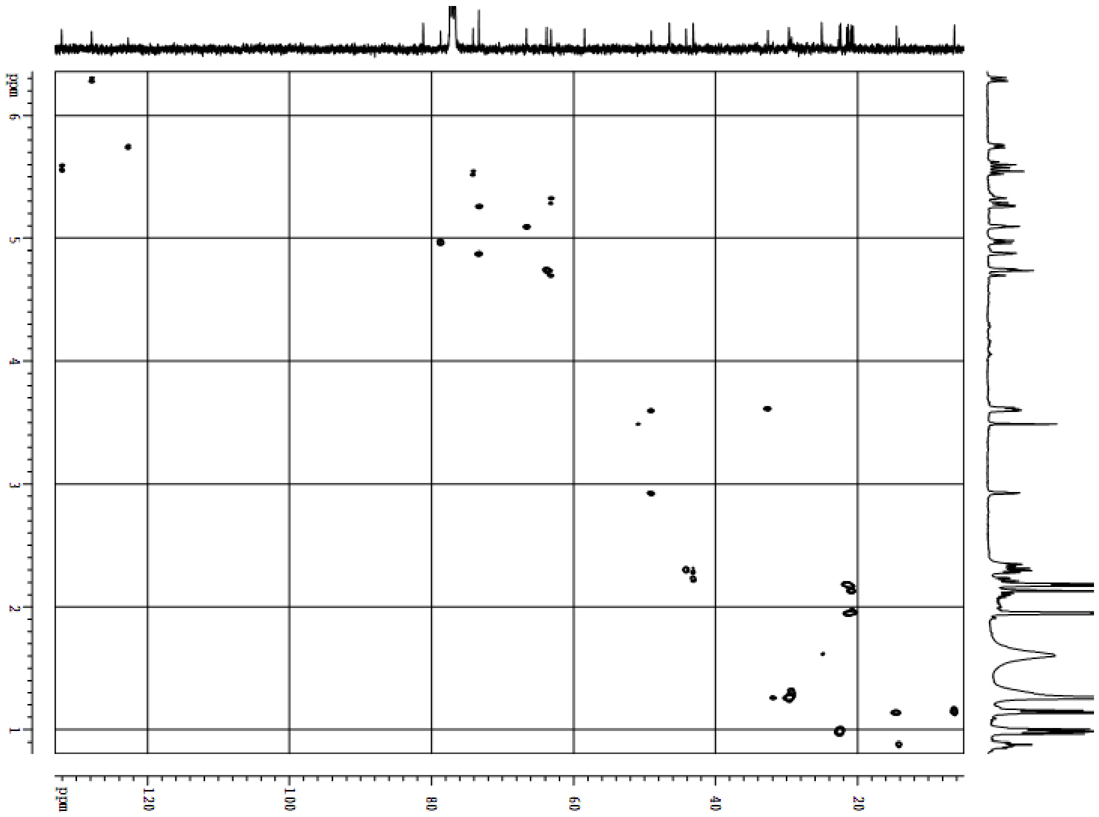

Figure S142. ^{1}H-^{1}H COSY spectrum of the new compound **18**.

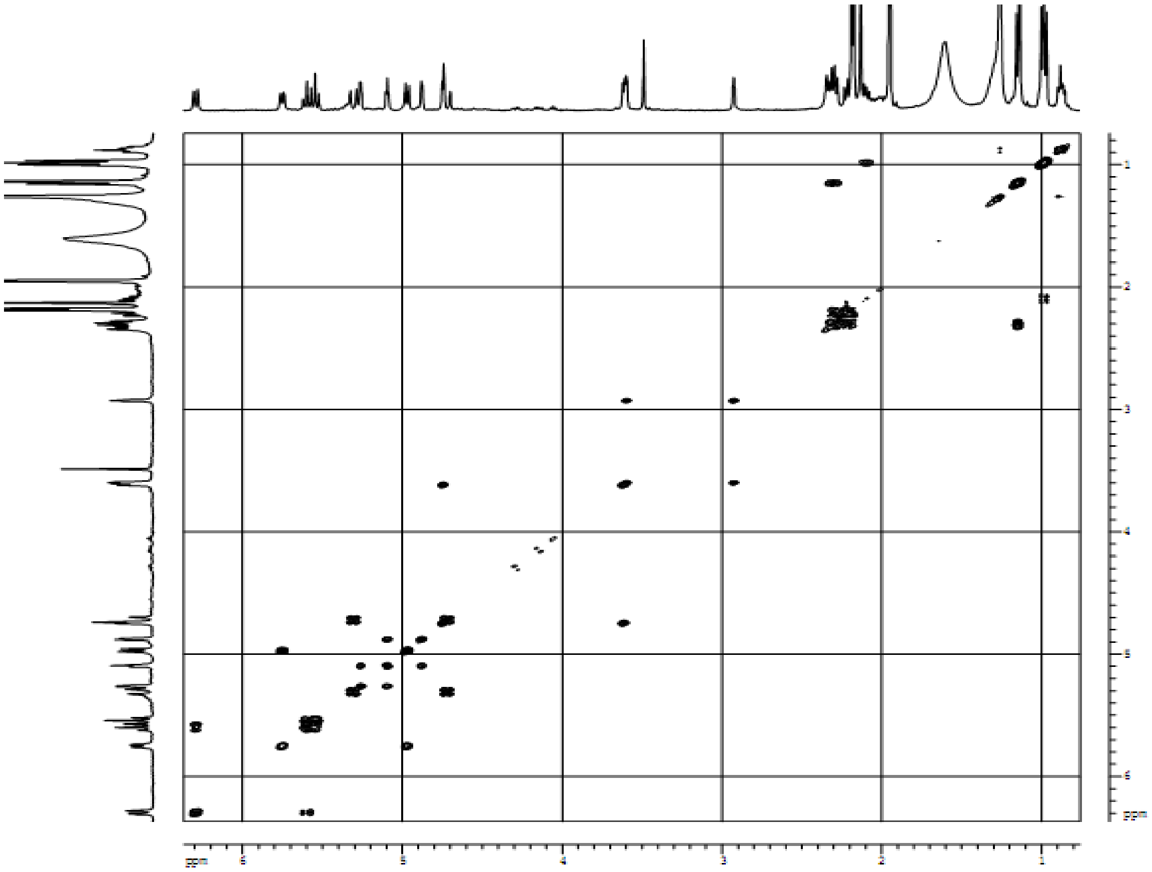

Figure S143. HMBC spectrum of the new compound **18**.

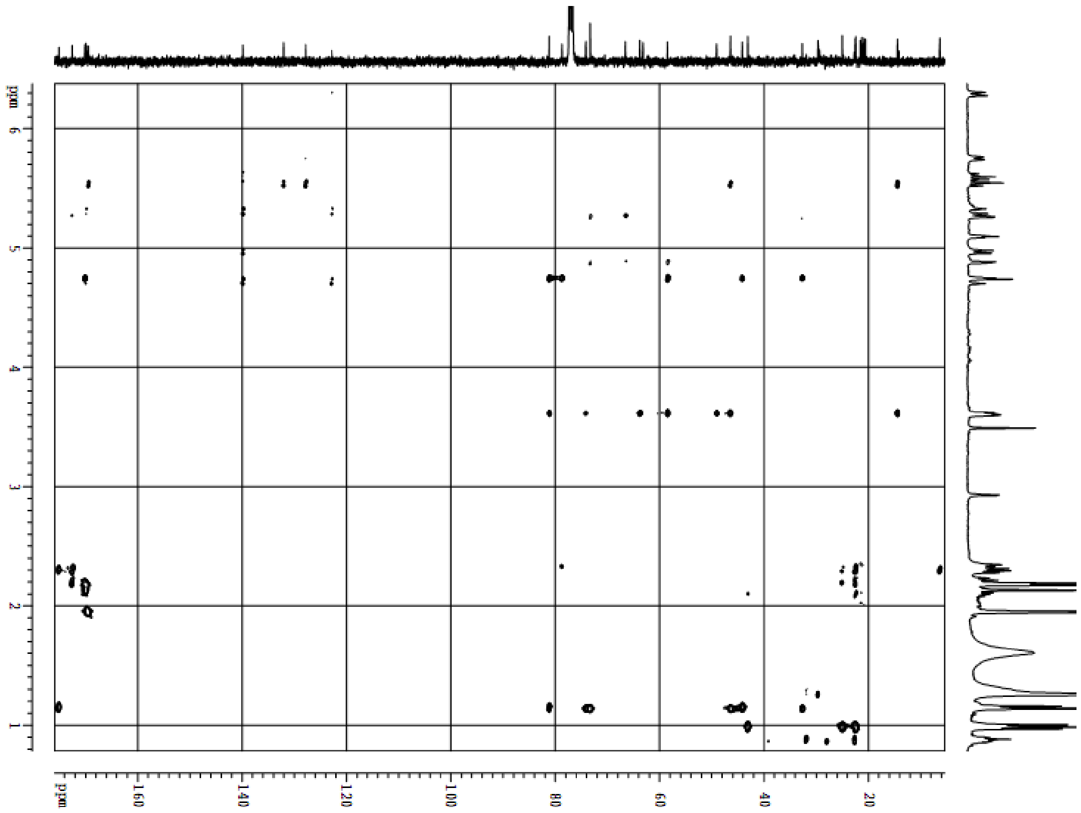

Figure S144. NOESY spectrum of the new compound **18**.

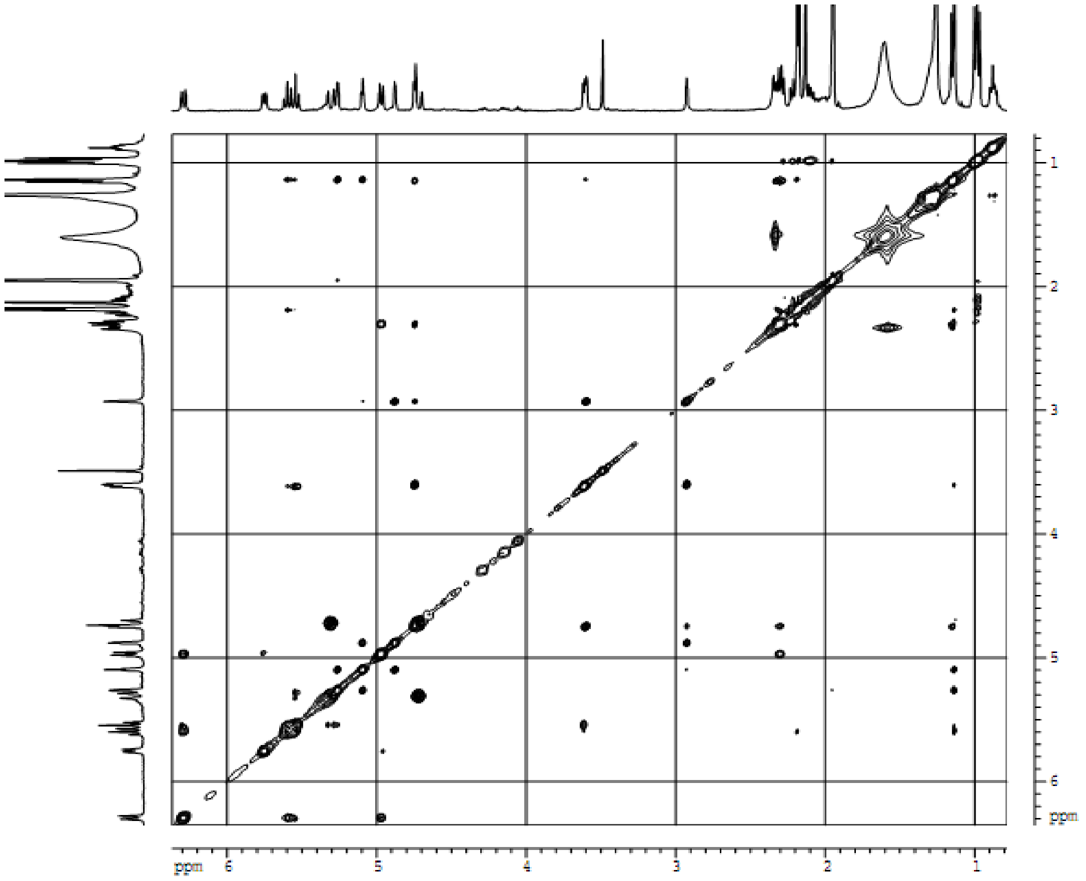

Supplement: Supplementary File 1 — Supplementary Information (PDF, 5867 KB) [file marinedrugs-11-01565-s001.pdf]
